# An age-based, RNA expression paradigm for survival biomarker identification for pediatric neuroblastoma and acute lymphoblastic leukemia

**DOI:** 10.1186/s12935-019-0790-5

**Published:** 2019-03-27

**Authors:** Andrea Diviney, Boris I. Chobrutskiy, Saif Zaman, George Blanck

**Affiliations:** 1 Department of Molecular Medicine, Morsani College of Medicine, University of South Florida, 12901 Bruce B. Downs Bd. MDC7, Tampa, USA; 2 Immunology Program, H. Lee Moffitt Cancer Center and Research Institute, Tampa, FL 33612 USA

**Keywords:** Diagnostic age, Pediatric cancer, Neuroblastoma, Acute lymphoblastic leukemia, Chromosome 17, Age of onset

## Abstract

**Background:**

Pediatric cancer survival rates overall have been improving, but neuroblastoma (NBL) and acute lymphoblastic leukemia (ALL), two of the more prevalent pediatric cancers, remain particularly challenging. One issue not yet fully addressed is distinctions attributable to age of diagnosis.

**Methods:**

In this report, we verified a survival difference based on diagnostic age for both pediatric NBL and pediatric ALL datasets, with younger patients surviving longer for both diseases. We identified several gene expression markers that correlated with age, along a continuum, and then used a series of age-independent survival metrics to filter these initial correlations.

**Results:**

For pediatric NBL, we identified 2 genes that are expressed at a higher level in lower surviving patients with an older diagnostic age; and 4 genes that are expressed at a higher level in longer surviving patients with a younger diagnostic age. For pediatric ALL, we identified 3 genes expressed at a higher level in lower surviving patients with an older diagnostic age; and 17 genes expressed at a higher level in longer surviving patients with a younger diagnostic age.

**Conclusions:**

This process implicated pan-chromosome effects for chromosomes 11 and 17 in NBL; and for the X chromosome in ALL.

**Electronic supplementary material:**

The online version of this article (10.1186/s12935-019-0790-5) contains supplementary material, which is available to authorized users.

## Background

Age of diagnosis may be particularly important in pediatric cancers due to the significant developmental changes that occur in humans from birth to age 18. Put another way, a few years in the life of a child represents a substantial percentage change in overall lifespan, not the case in later stages of adulthood.

Several studies have indicated that age of onset for pediatric neuroblastoma (NBL) and pediatric acute lymphoblastic leukemia (ALL) is reflective of disease course. Since the 1970s, a survival difference in pediatric NBL has been noted between older and younger diagnostic age, with a diagnostic age of 12 months or older reflective of significantly poorer survival [1]. In a 2005 study, pediatric NBL patients diagnosed between ages 12 and 18 months were found to have a higher 6-year event-free survival ate than those diagnosed later in life [2]. A study in 2011 found similar results, suggesting that while the impact of diagnostic age on prognosis has decreased since it was first detected in the 1970s, it remains a strong indication of survival rates for pediatric NBL patients [1].

This stark difference in survival rates based on age at diagnosis would suggest developmental, gene expression differences representing possibly unknown or as yet uncharacterized subdivisions of NBL, and indeed, certain gene expression related distinctions have been associated with pediatric NBL progression and prognoses distinctions. Lack of amplification of the MYCN gene, in addition to general hyperploidy, have been found to represent improved prognoses for pediatric NBL patients ages 12 to 18 months [3]. ATRX mutations have also been found to be increased in pediatric NBL patients with an older age at diagnosis, suggesting that expression of the wild-type version of this gene contributes to survival in patients diagnosed at a younger age [4].

Pediatric ALL also indicates elevated survival rates for patients diagnosed at a younger age. A 2014 study indicated that survival of pediatric ALL patients decreased with age at diagnosis, excluding those diagnosed within the first year of life, where there was the worst prognosis [5]. While the mutations of several genes have been correlated with survival in this cancer, none have also been assessed in a context of age at diagnosis.

Keeping in mind that many gene expression scenarios, particularly associated with development [6, 7], involve a gradient of expression and signal pathway activation gradients, and that signal pathway activation gradients have also been reported to represent distinct outputs in the cancer setting [8–10], we took an approach to biomarker discovery for NBL and ALL that emphasized a continuum of expression levels, with the expectation that, for certain genes, the higher the expression level, the greater the probability of a discreet effect, in this case a discreet effect leading to a survival distinction. Thus, in this study, we used RNA expression data from the TARGET database to first identify genes whereby a continuum of expression could be established as having a correlation with age, and then to additionally, independently filter such genes for an association of expression levels with distinct survival rates.

## Methods

### Clinical information for pediatric NBL

Of the 1076 pediatric NBL patients, 227 were age 1 or younger (21.1%); 825 were between age 1 and 10 (76.7%); 21 were between age 10 and 18 (1.95%); and 3 were over the age of 18 (0.28%). There were 463 females (43%) and 613 males (57%). 792 patients were white (73.6%); 127 were black or African American (11.8%); 29 were Asian (2.7%); 11 were Native Hawaiian or Pacific Islander (1.02%); 3 were American Indian or Alaskan Native (0.28%); and 114 did not report race or were of unknown race (10.6%). Clinical reports classified 89 patients as stage 1 (8.27%); 25 as stage 2a (2.32%); 36 as stage 2b (3.35%); 92 as stage 3 (8.55%); 777 as stage 4 (72.2%); and 55 as stage 4s (5.11%), with 2 patients having unknown staging (0.19%).

### Clinical information for pediatric ALL

For the 1550 pediatric ALL patients, five did not have any clinical information available and were therefore not used for survival analysis in this report. Of the 1545 remaining patients, 904 were age 10 or younger (58.5%); 584 were between age 10 and 18 (37.8%); and 57 were over age 18 (3.69%). There were 642 females (42%) and 903 males (58%). 1158 of the patients were white (75.0%); 109 were black or African American (7.06%), 67 were Asian (4.34%); 7 were Native Hawaiian or Pacific Islander (0.45%); 4 were American Indian or Alaskan Native (0.26%); and 200 were of unknown race (12.9%). CNS staging had been recorded for all patients, with 1255 staged as CNS 1 (81.2%), 124 as CNS 2 (8.03%), 73 as CNS 2a (4.72%), 26 as CNS 2b (1.68%), 19 as CNS 2c (1.23%), 20 as CNS 3 (1.29%), 12 as CNS 3a (0.78%), 7 as CNS 3b (0.45%), and 7 as CNS 3c (0.45%). Only 2 patients did not have CNS staging information available (0.13%).

### Gene expression correlation with diagnostic age

Survival and RNA microarray data [11, 12] were obtained from http://www.cbioportal.org. A Pearson’s correlation coefficient was calculated using an automated script (available upon email request to the corresponding author) between diagnostic age (in days) and gene expression values for each gene individually. The expression of genes with a positive correlation coefficient and a p-value < 0.05, for the correlation, were categorized as “upregulated with age”. The expression of genes with a negative correlation coefficient and a p-value < 0.05, for the correlation, were categorized as “downregulated with age”. In other words, in this latter case, we identified genes that were upregulated in younger patients.

### Identification of individual survival markers

Individual survival markers were identified using a Kaplan–Meier survival analysis for each individual gene. An automated script (available upon email request to the corresponding author) calculated survival for each gene individually as follows: For each gene, barcodes were organized by expression value, then the top 20% of expressers and bottom 20% of expressers were compared using a Kaplan–Meier survival analysis. Genes with a p-value < 0.05 and a larger median survival value in the top 20% of expressers were categorized as “upregulated, high survival markers”. Genes with a p-value < 0.05 and a smaller median survival value in the top 20% of expressers were categorized as “upregulated, low survival markers”. Figures representing Kaplan–Meier survival analyses were generated using GraphPad Prism software (version 7).

Chromosome location data was obtained from NCBI (https://www.ncbi.nlm.nih.gov/) and GeneCards (https://www.genecards.org/). Chromosome locations of genes and the figures representing these data were generated using Microsoft Excel.

## Results

### Identification of pediatric NBL survival markers

**Fig. 1:**
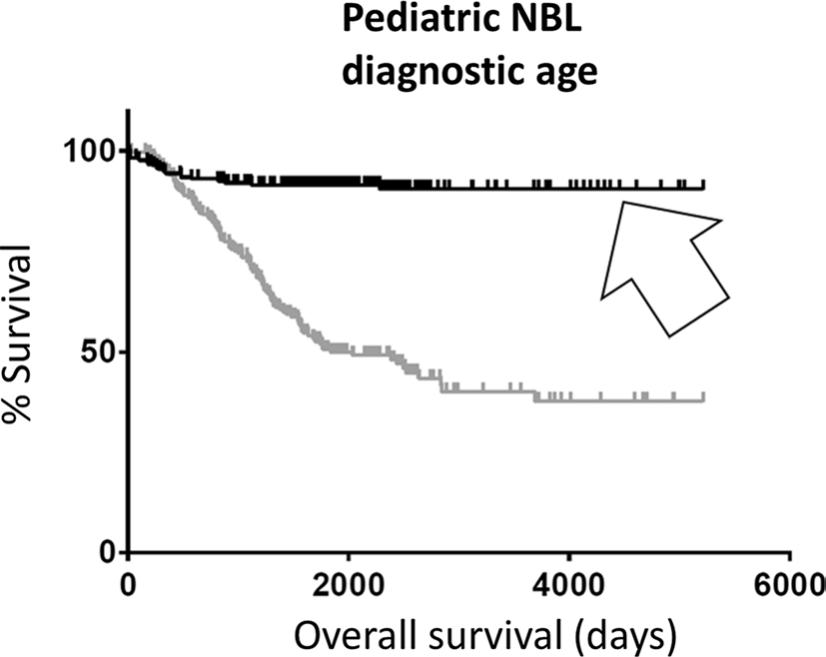
Kaplan-Meier (KM) overall survival (OS) curve for pediatric NBL barcodes representing the oldest 20% (black) of patients compared to barcodes representing the youngest 20% (gray) of patients (Additional file 1: Table S1). Youngest 20% of patients were found to have significantly better survival than the oldest 20% (arrow). Median OS for oldest 20% barcodes, 1836 days; median OS for youngest 20% barcodes, undefined. Log rank comparison p-value < 0.0001

Using a novel scripted algorithm (“Methods” section), clinical information for 1076 pediatric neuroblastoma (NBL) patients was sorted by diagnostic age in days, and an automated Kaplan–Meier (KM) analysis of the oldest 20% and the youngest 20% of the patients revealed a significant difference in survival (KM log rank p-value < 0.0001), with the oldest 20% having lower survival (Fig. 1; Additional file 1: Table S1). This analysis was repeated for the upper and lower fiftieth percentiles, for age, with results being consistent with the initial results using the twentieth percentiles (Additional file 1: Table S2).

Considering the patient group representing the twentieth percentiles above, 247 had microarray data available through the TARGET database [11, 12], representing 23,434 genes. Again, using an automated process (“Methods” section), we determined which of these genes represented RNA expression levels that differed significantly with age, based on the statistical significance of a Pearson’s correlation coefficient (“Methods” section).

With the above processing, 623 genes were found to be significantly correlated with age (upregulated in older pediatric NBL patients), and 1334 genes were found to be significantly, inversely correlated with age (upregulated in younger pediatric NBL patients) (Additional file 1: Tables S3, S4).

We next identified which of the 23,434 genes were independent survival markers (i.e., without regard to age-defined patients representing survival distinctions described in the above paragraph.) Of the 623 genes significantly correlated with older age (above paragraph), 95 (Additional file 1: Table S5) were also, independently, markers of low survival, i.e., when upregulated (with “upregulated” referring to a significant difference in the top 50% and bottom 50% of microarray levels, as determined by log-transformed t-test p-value < 0.05.) That is, the top expressers had significantly worse survival compared to the bottom expressers (with the survival distinction represented by a KM log rank p-value < 0.05.)

**Fig. 2:**
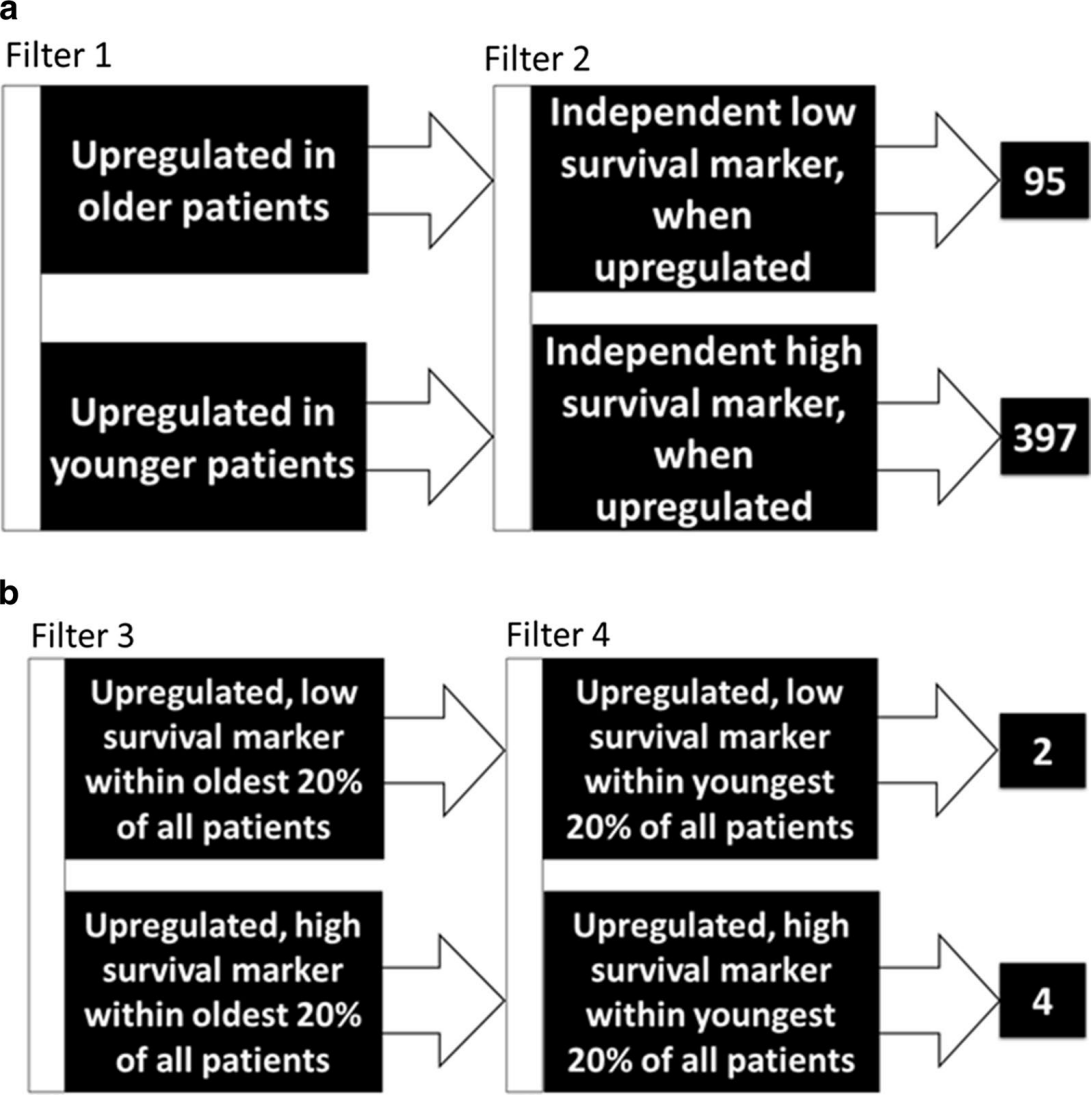
Diagrams depicting the sorting process of genes to identify gene-expression based survival markers based on diagnostic age. **a** Filter 1 indicates genes that were significantly upregulated in either older (top) or younger (bottom) patients, Pearson Correlation Coefficient, p-value < 0.05. Filter 2 indicates genes that were significantly correlated with either low (top) or high (bottom) survival, log-transformed t-test p-value < 0.05. **b** Filter 3 indicates genes that were also low (top) or high (bottom) survival markers, when upregulated, within the oldest 20% of patients. Finally, Filter 4 indicates genes that were also low (top) or high (bottom) survival markers, when upregulated, within the youngest 20% of patients. The right-most boxes indicate the number of genes that cleared all four filters, indicating the upregulated genes that are indicative of older age and lower survival (2 genes) as well as the upregulated genes indicative of younger age and higher survival (4 genes)

Of the 1334 genes significantly correlated with younger age, 397 (Additional file 1: Table S6) were also identified, independently, as high survival markers, i.e., when upregulated (with “upregulated” referring to a significant difference in the top and bottom microarray levels, as determined by log-transformed t-test p-value < 0.05). That is, the top expressers had significantly better survival compared to the bottom expressers (with the survival distinction represented by a KM log rank p-value < 0.05) (Fig. 2a).

**Fig. 3:**
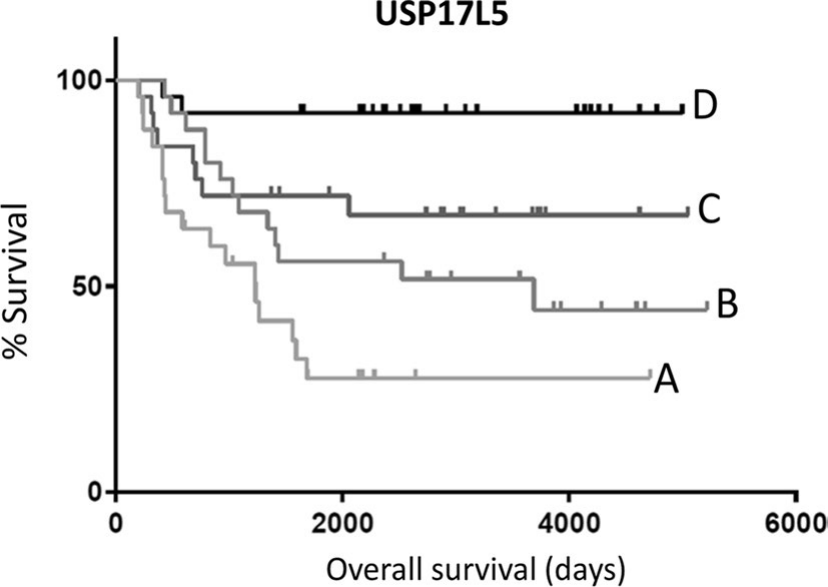
Kaplan-Meier (KM) overall survival (OS) curve for pediatric NBL barcodes comparing the oldest 20% of patients and high USP17L5 microarray-based expression (A), oldest 20% of patients and low USP17L5 microarray-based expression (B), youngest 20% of patients and high USP17L5 microarray-based expression (C), and youngest 20% of patients and low USP17L5 microarray-based expression (D). Median OS for (A), 1235 days; median OS for (B), 3691 days; median OS for (C), undefined; median OS for (D), undefined. Log rank comparison p-value < 0.0001

Of the 95 genes that were upregulated with age, and were independently correlated with low survival when upregulated, we identified 7 genes that, when upregulated, were also correlated with low survival solely within the oldest 20% of pediatric NBL patients (p-value < 0.05). Of these 7 genes, we identified 2 genes that, when upregulated, were independently correlated with low survival (p-value < 0.05) solely within the youngest 20% of pediatric NBL patients: USP17L5 and SLC25A5 (Fig. 2b; Additional file 1: Table S7). To be clear, these two genes, USP17L5 and SLC25A5, have withstood 4 filters with regard to upregulation and low survival. First, the two genes are upregulated in older patients, known to have a poor survival rate. Second, the two genes are upregulated in low surviving patients regardless of age. Third, within the older group, the genes are upregulated with poor survival. And, within the younger group, USP17L5 and SLC25A5 are upregulated with relatively poor survival. A KM curve of the USP17L5 survival marker illustrates the point that this marker is associated with decreased survival within both the oldest 20% and the youngest 20% of pediatric NBL patients (Fig. 3; for SLC25A5 KM curve, see Additional file 1: Figure S1).

**Fig. 4:**
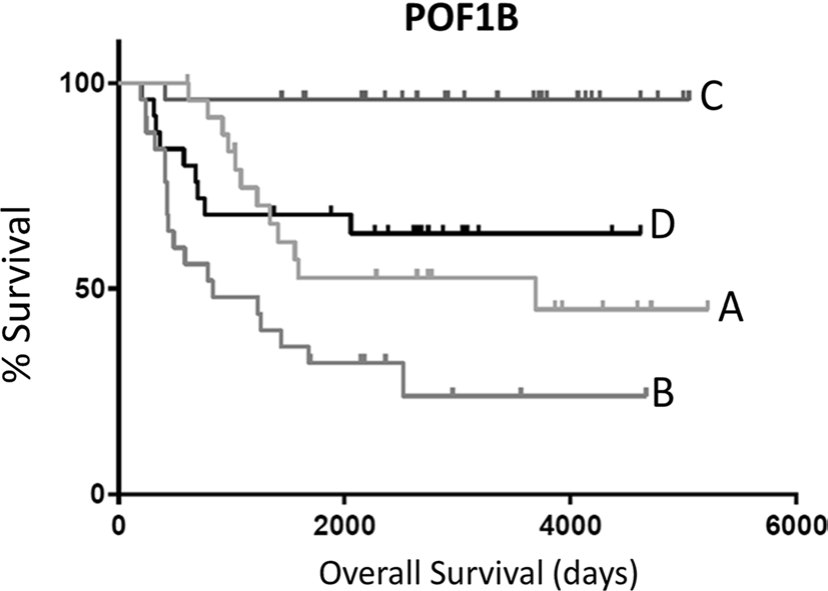
Kaplan–Meier (KM) overall survival (OS) curve for pediatric NBL barcodes comparing the oldest 20% of patients and high POF1B Microarray expression (A), oldest 20% of patients and low POF1B microarray-based expression (B), youngest 20% of patients and high POF1B microarray-based expression (C), and youngest 20% of patients and low POF1B microarray-based expression. Median OS for (A), 3691 days; median OS for (B), 833 days; median OS for (C), undefined; median OS for (D), undefined. Log rank comparison p-value < 0.0001

We used a similar filtering process for genes that were upregulated with younger age, and, independently of age, also high survival markers, when upregulated. Of these resulting 397 genes, we identified 15 genes that were also independent, high survival markers (p-value < 0.05) within the oldest 20% of pediatric NBL patients. Of these 15 genes, we identified 4 genes that were also independent high survival markers (p-value < 0.05) within the youngest 20% of pediatric NBL patients: POF1B, RND3, KLC4, and SLC12A1 (Fig. 2b; Additional file 1: Table S8). That is, these 4 genes were upregulated among high survivors within the younger patient set. A KM curve of the POF1B survival marker illustrates that upregulation of this gene is associated with increased survival within both the oldest 20% and with the youngest 20% of pediatric NBL patients (Fig. 4; for RND3, KLC4, and SLC12A1 see Additional file 1: Figure S1).

All of the final NBL results have been confirmed with three replicative datasets (Table 1) (https://hgserver1.amc.nl/) [13].

**Table 1:** Evaluation of age-based, NBL survival biomarkers (Fig. 2b) using a replicative dataset (https://hgserver1.amc.nl/)

Gene	Kocak (n = 649)	Oberthuer (n = 251)	SEQC (n = 498)
(Upregulation associated with older age and worse survival)			
USP17L5	N/A	N/A	1.20E − 08
SLC25A5	1.00E−26	1.30E−32	1.00E−33
(Upregulation associated with younger age and better survival)			
POF1B	4.50E−16	N/A	1.10E−17
RND3	1.60E−08	5.30E−06	2.80E−05
KLC4	4.80E−03	0.099	6.70E−10
SLC12A1	8.40E−05	N/A	1.00E−21

p-values for separate datasets indicated in right three columns

### Gene chromosome distribution for pediatric NBL

**Fig. 5:**
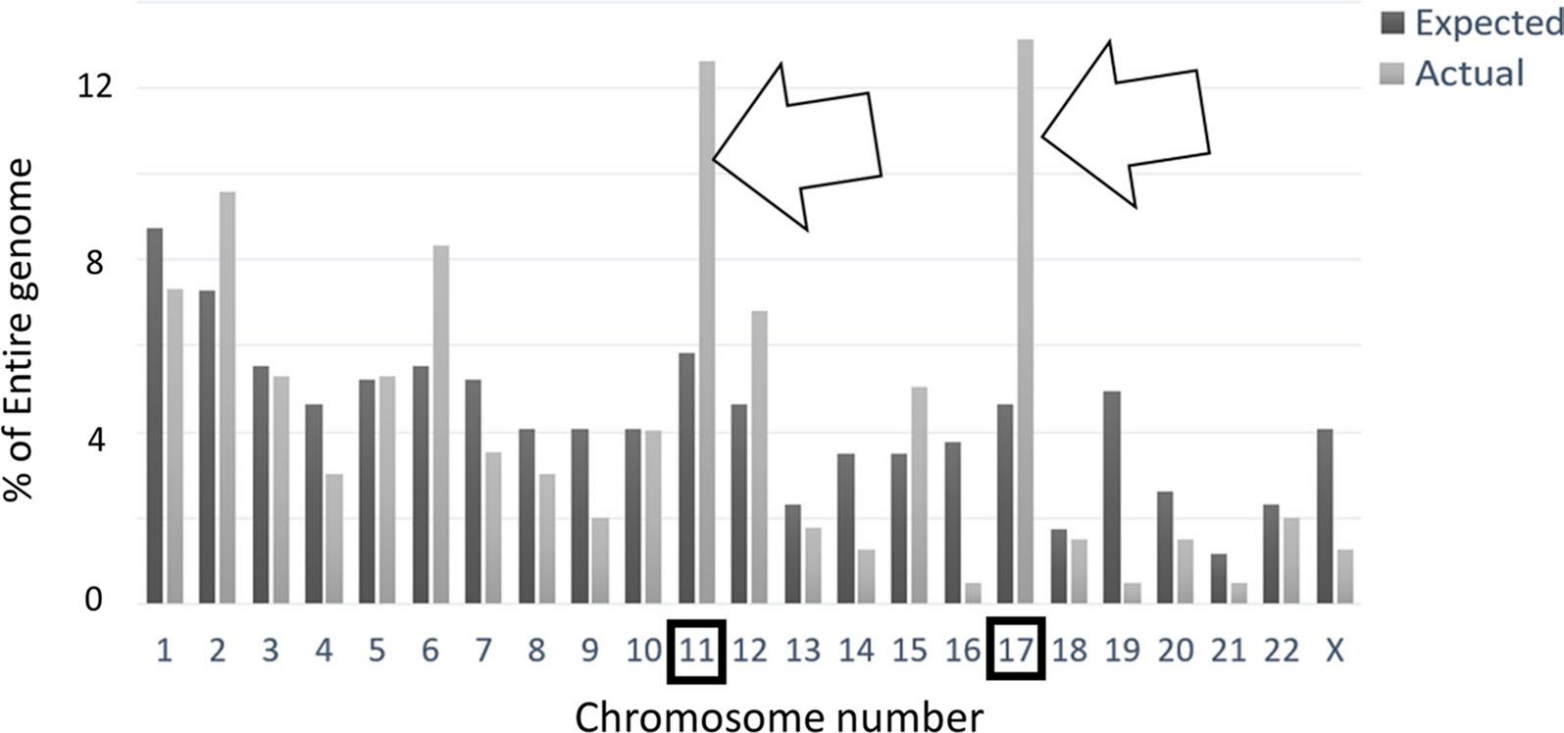
Actual (light gray) chromosome distribution of the 397 genes upregulated in younger, higher surviving pediatric NBL patients compared to expected (dark gray) chromosome distribution (Additional file 1: Table S9). Chromosome 17 was found to be the location of 13.13% of these genes (right-side arrow), compared to its expected representation of 4.65% (p-value < 0.0001, Additional file 1: Table S9). Chromosome 11 was found to be the location of 12.63% of these genes (left-side arrow), compared to its expected representation of 5.81% (p-value < 0.0001, Additional file 1: Table S9)

To identify any potential linkage among the indicated, gene expression survival markers, we did a chromosomal location analysis of the genes that were correlated with both age and survival. Among the 397 genes upregulated among younger patients that were also independently indicated as upregulated among high survivors (Fig. 2a), both chromosome 11 and 17 locations were overrepresented. Chromosome 11 was expected to contain 5.81% of these genes, based on random chance, but contained 12.63%, while chromosome 17 was expected to contain 4.65% but contained 13.13% (Fig. 5: p-value < 0.0001, for expected versus observed, for both 11 and 17; Additional file 1: Table S9).

### Identification of pediatric ALL survival markers

**Fig. 6:**
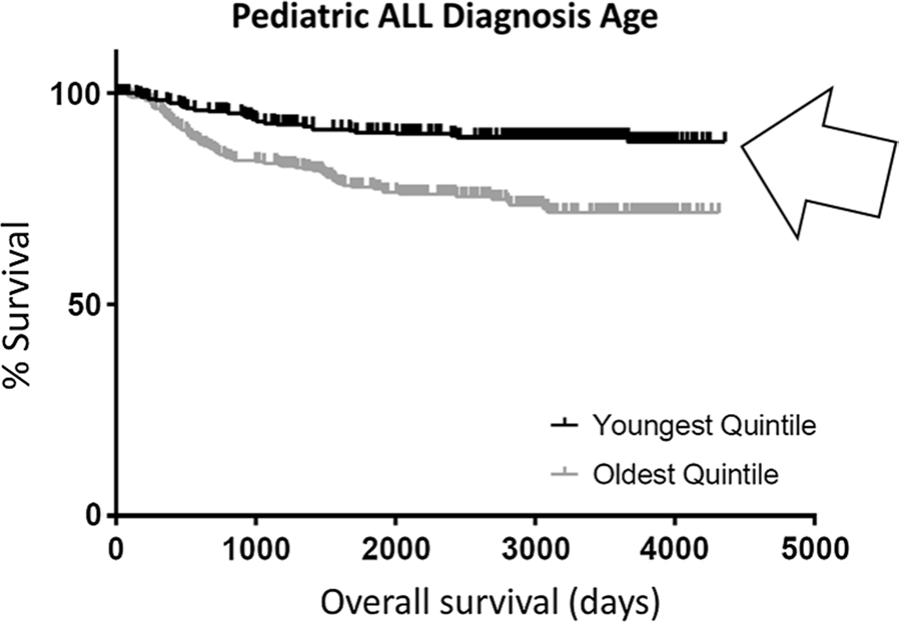
Kaplan-Meier (KM) overall survival (OS) curve for pediatric ALL barcodes representing the oldest 20% (black) of patients compared to barcodes representing the youngest 20% (gray) of patients (Additional file 1: Table S1). Youngest 20% of patients were found to have significantly better survival than the oldest 20% (arrow). Median OS for oldest 20% barcodes, undefined; median OS for youngest 20% barcodes, undefined. Log rank comparison p-value < 0.0001

Using clinical data available for 1550 pediatric ALL patients [11, 12], a KM curve of the oldest 20% and the youngest 20% of pediatric ALL patients also revealed a significant difference in survival between the two groups (p-value < 0.0001), with the oldest 20% having lower survival (Fig. 6).

Of these patients, 203 had microarray data available through the TARGET database, representing 23,434 genes. We then used a similar automated process as in the case of pediatric NBL above (“Methods” section) to identify survival markers in ALL. 1316 genes were upregulated with older age, and 471 of those were also independent low survival markers, i.e., regardless of age, when upregulated. 1366 genes were upregulated in younger patients, and 1057 of those were also independent, high survival markers, when upregulated.

Of the 471 genes upregulated with age that were also, independently, low survival markers, 21 were indicative of low survival within the oldest 20% of pediatric ALL patients. Three of these genes were also low survival markers, when upregulated, within the youngest 20% of pediatric ALL patients: THAP4, ZNHIT2, and SF3B2 (Additional file 1: Figure S1) of the 1057 genes upregulated in younger patients, and that were independently high survival markers when upregulated, 77 were indicative of higher survival within the oldest 20% of pediatric ALL patients. Seventeen of these genes were also high survival markers within the youngest 20% of pediatric patients: COL5A1, GABBR1, HACE1, RPS6KA5, LAMB1, BMP3, MAML3, SLX4IP, EPHA7, OR52H1, DDX60L, SNORA19, SNORA2A, ENTHD2, TRIP11, ZNF81, and ZNF514 (Additional file 1: Figure S1).

### Gene chromosome distribution in pediatric ALL

**Fig. 7:**
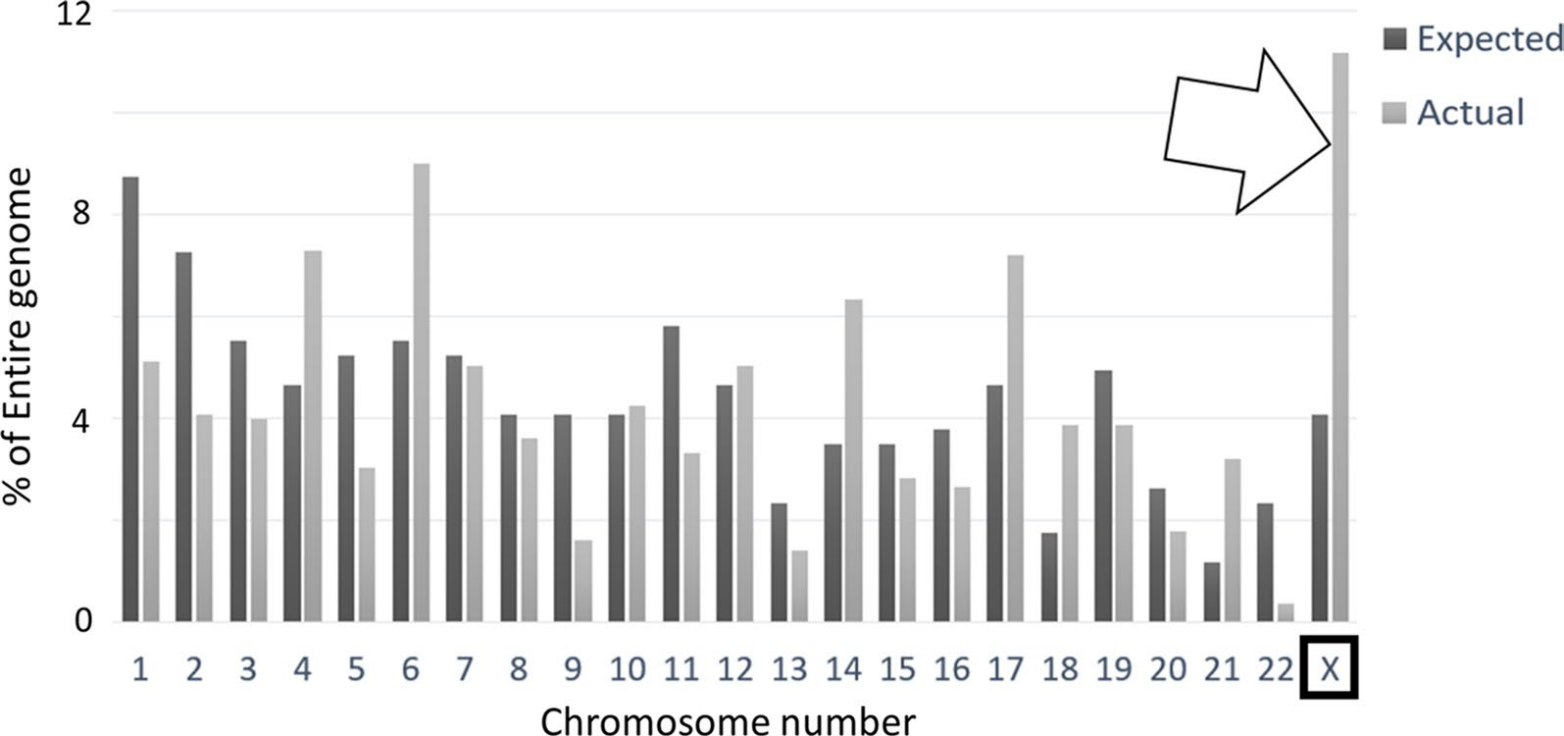
Actual (light gray) chromosome distribution of the 1057 genes upregulated in younger, higher surviving pediatric ALL patients compared to expected (dark gray) chromosome distribution (Additional file 1: Table S12). Chromosome X was found to be the location of 11.16% of these genes (arrow), compared to its expected representation of 4.07% (p-value < 0.0001)

Because chromosomes 11 and 17 were found to be common locations for genes upregulated in young, high-surviving pediatric NBL patients, a similar chromosomal distribution analysis was performed for the 1057 genes that were upregulated in younger patients and independently indicative of increased survival in pediatric ALL patients. Of these genes, 4.07% were expected to be located on chromosome X, but in fact, 11.16% were located on this chromosome (p-value < 0.0001, for expected versus observed, for X; Fig. 7; Additional file 1: Table S12).

### Diagnostic age and survival for pediatric Wilms tumor, AML, and osteosarcoma

After finding survival markers based on diagnostic age in both pediatric NBL and pediatric ALL, we performed similar analyses for pediatric Wilms tumor, pediatric acute myeloid leukemia (AML), and pediatric osteosarcoma. While a KM analysis of the oldest 20% and the youngest 20% of pediatric Wilms patients did reveal a significant difference in survival between the two groups, with the oldest 20% having lower survival (p-value = 0.0237), no genes were identified using all four filters (Fig. 2) used for identifying consistent survival markers for NBL and ALL, as described above. KM analyses of the oldest 20% and youngest 20% of patients for both pediatric AML (p-value = 0.4128) and pediatric osteosarcoma (p-value = 0.7524) found no significant difference in survival based on age.

## Discussion

The above data provided two basic indications. First, the upregulation or downregulation of a particular set of genes associated with a continuum of age can be used as a starting point to identify gene expression levels associated with survival rates, in this case where the survival rates are, in turn, associated with patient age. The approach above (Fig. 2) provides new candidate biomarkers of survival, and new candidate mediators of tumor development, based on an approach that represents a continuum of expression levels with the presumption (not directly addressed here) that such a continuum would reflect probabilistic impacts on cellular or physiological events impacting survival. From this base of candidates, further filters were applied to identify and validate the gene expression-level, survival associations. This approach represents an important, distinct starting point, in comparison to many common approaches to identifying biomarkers, and drivers of tumorigenesis, motivated by evidence that indicates that amplification of signaling pathways, rather than potential on/off switches, can ultimately have highly discreet phenotypic results, not only in tumorigenesis [10, 14] but in normal development [6, 7]. Unlike a starting point for many survival biomarkers, the empirical approach, such as transfection of an oncoprotein and assaying increased tissue culture cell division, may not be possible for certain biomarkers or facilitators of tumorigenesis. And indeed, as discussed further below, several of the genes outputted above have little previous connection to tumorigenesis, perhaps genes not easily identified in empirical approaches that require essentially, but unnaturally, on/off switches in signaling or other effects for a detectable output. Other paradigms, with a component of continuity and correlation, in the absence of empirical approaches have revealed similar successes, for example, the correlation of mutation burdens with cancer immune responses and responses to immunotherapy [15–18]; and the correlation of mutation burden in haematopoietic stem cells with subsequent development of acute myeloid leukemia [19].

Second, the data above are consistent with anomalies impacting large regions of single chromosomes, i.e., chromosomes, 11 and 17 in pediatric NBL; and the X chromosome in pediatric ALL.

In terms of the functional impacts of potential tumor drivers, or the expression of proteins that might limit tumorigenesis, it does need to be kept in mind that age of diagnosis can represent a lot of variation in terms of age of onset of the tumor, which would presumably start with one tumorigenic cell at an undetermined age. Nevertheless, correlative studies that indicate a value of gene expression level assessments based on age do likely provide at a minimum new prognoses biomarker opportunities and new candidates for assessing specific tumor functions.

As for the two genes upregulated with lower survival, USP17L5 represents an apparent, relatively poorly studied member of a family of ubiquitin peptidases; and SLC25A5 represents a carrier for ADP to the mitochondria, and a carrier of ATP from the mitochondria to the cytoplasm [20]. The ubiquitin peptidases, including the USP17 sub-family, have been variously associated with cancer progression and cancer growth inhibition (and apoptosis), apparently dependent on the type of cancer [21–23] or other factors not yet fully appreciated. SLC25A5 specifically has been reported to be down-regulated with metastasis in hepatocellular carcinoma [24], with no information available for NBL. As in the case of ubiquitin peptidases, as a family, the solute carrier proteins have a complicated association with cancer progression, or lack of cancer progression, dependent on very specific situations.

As for the four genes that are upregulated with youth and better NBL survival, only RND3 has a detailed research history with cancer. That cancer history is contradictory, as with other genes, with reports indicating a potential for high RND3 expression representing both pro- and anti-cancer results [25–27]. A recent review regarding RND3 specifically evaluated the pro- and anti-cancer functions and concluded that indeed, the overall impact of RND3 is context dependent [28]. Mutation of SLC12A1 has been associated with a short survival in NBL [29]. POF1B has no known, previous connection to NBL and little connection to cancer in general.

Pediatric ALL also reflects decreased survival with older age of diagnosis, although this correlation has not been extensively investigated [5]. We found that the pediatric ALL patients in the TARGET data set had lower survival with higher diagnostic age, confirming this risk factor for this dataset (Fig. 6). Employing the above discussed paradigm (for NBL), the upregulation of 3 genes was found to be associated with poor survival and high diagnostic age in pediatric ALL (Additional file 1: Figure S1), none of which have any previous connection to cancer; and the upregulation of 17 genes was found to be associated with high survival and low diagnostic age in this cancer (Additional file 1: Figure S1).

Of the 17 genes that, when upregulated, were associated with high survival and low diagnostic age in pediatric ALL patients, only ZNF81 is located on the X chromosome, discussed below. Of the other 16 genes, COL5A1, GABBR1, HACE1, EPHA7, and TRIP11 have well-documented associations with cancer. Inhibition of GABBR1 (gamma-amino-butyric acid type B receptor 1) has been associated with progression of colorectal cancer, whereas overexpression of this gene served as an inhibitor of miRNAs that would otherwise lead to proliferation of this cancer [30]. It is possible that this gene serves a similar role when upregulated in younger, higher-surviving pediatric ALL patients. EPHA7 may also be sequestering a microRNA, namely miR-944, which, when expressed at a high level, has been shown to facilitate proliferation of non-small cell lung cancer cells. Thus, high levels of EPHA7 may have the effect of sequestering microRNAs and reducing proliferation in other cancers [31]. HACE1 is an E3 ligase downregulated in several cancers, including gastric cancer and breast cancer, and was found to inhibit the Wnt/β-catenin pathway, thereby playing a role in suppressing tumorigenesis [32, 33]. The pathway involving TRIP11 and triiodothyronine is necessary for localization of TRIP11 to the nucleus and was found to be disrupted in renal cell cancer, leading to progression [34]. Finally, COL5A1 has been found to have associations with gastric cancer, non-small cell lung cancer, and renal cancer [35–37]. Overall, these overlapping, previous studies are consistent with the upregulation of these genes in the younger patients and in the longer surviving patients. Additional gene ontology information for both NBL and ALL is provided in Additional file 1: Table S13.

While the lack of an opportunity to confirm newly identified biomarkers consistently and firmly with either a pro-cancer or anti-cancer phenotype based on a history of gene expression functions in other cancers can be limiting, it is in fact the expectation, based on decades of previous research. First, as noted in specific cases above, there are disparities of gene expression function related to context. Second, it is clear that many cancer hallmarks are dependent on signal pathway amplification rather than a molecular on/off switch. This is exemplified by feed forward apoptosis, whereby transcription factors that activate pro-proliferative genes, such as histone genes, also activate apoptosis-effector genes, i.e., when these transcription factors are expressed at high levels [8–10, 38–42]. Third, even outside of the cancer setting, different tissues can have opposite functions for the same signaling pathway; FGFR3 activating mutations stimulate spermatocyte cell division but inhibit chondrocyte cell division, leading to achondroplasia [43, 44].

In pediatric ALL, there was a disproportionate increase in the number of genes expressed at a higher level in younger, longer surviving patients located on the X chromosome. There is very little in the literature regarding X chromosome loss and worse ALL survival or X chromosome gain and better survival. However, there has been one report with a small amount of data indicating loss of X chromosome in older patients with presumably poorer survival rates but where specific, relative survival data was lacking [45]. As for pediatric NBL and chromosomes 17 and 11, our data clearly indicated an overrepresentation of genes on these two chromosomes that were upregulated with better survival, suggesting chromosome loss in older, worse surviving patients. Again, there are no data now available regarding chromosome copy number variations (CNV) in very young NBL patients, the subject of this study. (For example, the youngest 20% of NBL patients in this study were all diagnosed under 1 year of age.) However, there have been reports of worse survival among older cohorts of patients with loss of 11q [46, 47]. The above data do not distinguish between CNV of either chromosome 11 or 17, respectively, versus loss or gain of heterochromatic regions that would affect gene expression. However, the previous reports of loss of chromosome 11q and poorer survival are consistent with chromosome loss in poorer surviving patients. 17q gain in NBL has been linked to lower survival in older patients. This is an apparent contradiction, however, these 17q data do not represent a significant overlap of our data, due to the lack 17q information for the younger patients in this study.

## Conclusion

A novel, age-based biomarker identification algorithm led to identification of several genes, where for the first time, expression levels either directly or inversely correlated with NBL and ALL survival, respectively; and led to the likely identification of a role for specific chromosome CNVs in NBL and ALL development. While the impact of these findings on clinical management is a longer term issue, the indicated survival markers are potentially useful prognostic tools. In addition, the genes at issue may suggest potential therapy targets.

## Additional file

**Additional file 1: Table S1.** Kaplan-Meier output for Fig. 1, with case barcodes at end of output. **Table S2.** Kaplan-Meier output summary for pediatric NBL diagnosis age survival curve, halves. **Table S3.** 623 genes upregulated in older pediatric NBL patients (Pearson Correlation Coefficients, p-values). **Table S4.** 1334 genes upregulated in younger pediatric NBL patients (Pearson Correlation Coefficients, p-values). **Table S5.** 95 genes upregulated in older pediatric NBL patients that are also, independently, correlated with low survival (p-values). **Table S6.** 397 genes upregulated in younger pediatric NBL patients that are also, independently, correlated with high survival (p-values). **Table S7.** Microarray values of every pediatric NBL patient for USP17L5 and SLC25A5. Table S8. Microarray values of every pediatric NBL patient for POF1B, RND3, KLC4, and SLC12A1. **Table S9.** Chromosome distribution of 397 genes upregulated in younger pediatric NBL patients that are also correlated with high survival. **Table S10.** Microarray values of every pediatric ALL patient for THAP, ZNHIT2, and SF3B2. **Table S11.** Microarray values of every pediatric ALL patient for COL5A1, GABBR1, HACE1, RPS6KA5, LAMB1, BMP3, MAML3, SLX4IP, EPHA7, OR52H1, DDX60L, SNORA19, SNORA2A, ENTHD2, TRIP11, ZNF81, and ZNF514. **Table S12.** Chromosome distribution of 1057 genes upregulated in younger pediatric ALL patients that are also correlated with high survival. **Table S13.** Gene ontology information, added in the revision. **Table S14.** KM curve median values for pediatric NBL and ALL genes, added in the revision. **Figure S1.** KM curve panels for all genes in Additional file 1: Table S14, added in the revision.

## Abbreviations

NBL: neuroblastoma
ALL: acute lymphoblastic leukemia
AML: acute myeloid leukemia
KM: Kaplan–Meier
TARGET: Therapeutically Applicable Research To Generate Effective Treatments
