# Supplementary material for: An age-based, RNA expression paradigm for survival biomarker identification for pediatric neuroblastoma and acute lymphoblastic leukemia
Source: Cancer Cell Int. 2019 Mar 27;19:73. doi: 10.1186/s12935-019-0790-5 (PMC6438000; doi:10.1186/s12935-019-0790-5)
Supplement: Supplementary file 1 — Additional file 1: Table S1. Kaplan-Meier output for Fig. 1, with case barcodes at end of output. Table S2. Kaplan-Meier output summary for pediatric NBL diagnosis age survival curve, halves. Table S3. 623 genes upregulated in older pediatric NBL patients (Pearson Correlation Coefficients, p-values). Table S4. 1334 genes upregulated in younger pediatric NBL patients (Pearson Correlation Coefficients, p-values). Table S5. 95 genes upregulated in older pediatric NBL patients that are also, independently, correlated with low survival (p-values). Table S6. 397 genes upregulated in younger pediatric NBL patients that are also, independently, correlated with high survival (p-values). Table S7. Microarray values of every pediatric NBL patient for USP17L5 and SLC25A5. Table S8. Microarray values of every pediatric NBL patient for POF1B, RND3, KLC4, and SLC12A1. Table S9. Chromosome distribution of 397 genes upregulated in younger pediatric NBL patients that are also correlated with high survival. Table S10. Microarray values of every pediatric ALL patient for THAP, ZNHIT2, and SF3B2. Table S11. Microarray values of every pediatric ALL patient for COL5A1, GABBR1, HACE1, RPS6KA5, LAMB1, BMP3, MAML3, SLX4IP, EPHA7, OR52H1, DDX60L, SNORA19, SNORA2A, ENTHD2, TRIP11, ZNF81, and ZNF514. Table S12. Chromosome distribution of 1057 genes upregulated in younger pediatric ALL patients that are also correlated with high survival. Table S13. Gene ontology information, added in the revision. Table S14. KM curve median values for pediatric NBL and ALL genes, added in the revision. Figure S1. KM curve panels for all genes in Additional file 1: Table S14, added in the revision. [file 12935_2019_790_MOESM1_ESM.pdf]

Supporting online material

Diviney et al., 2019

Table S1.	Kaplan-Meier output for Figure 1, with case barcodes at end of output.
Table S2.	Kaplan-Meier output summary for pediatric NBL diagnosis age survival curve, halves.
Table S3.	623 genes upregulated in older pediatric NBL patients (Pearson Correlation Coefficients, p-values).
Table S4.	1334 genes upregulated in younger pediatric NBL patients (Pearson Correlation Coefficients, p-values).
Table S5.	95 genes upregulated in older pediatric NBL patients that are also, independently, correlated with low survival (p-values).
Table S6.	397 genes upregulated in younger pediatric NBL patients that are also, independently, correlated with high survival (p-values).
Table S7.	Microarray values of every pediatric NBL patient for USP17L5 and SLC25A5
Table S8.	Microarray values of every pediatric NBL patient for POF1B, RND3, KLC4, and SLC12A1.
Table S9.	Chromosome distribution of 397 genes upregulated in younger pediatric NBL patients that are also correlated with high survival.
Table S10.	Microarray values of every pediatric ALL patient for THAP, ZNHIT2, and SF3B2.
Table S11.	Microarray values of every pediatric ALL patient for COL5A1, GABBR1, HACE1, RPS6KA5, LAMB1, BMP3, MAML3, SLX4IP, EPHA7, OR52H1, DDX60L, SNORA19, SNORA2A, ENTHD2, TRIP11, ZNF81, and ZNF514.
Table S12.	Chromosome distribution of 1057 genes upregulated in younger pediatric ALL patients that are also correlated with high survival.
Table S13.	Gene ontology information, added in the revision.
Table S14.	KM curve median values for pediatric NBL and ALL genes, added in the revision.
Figure S1.	KM curve panels for all genes in Table S14, added in the revision.

Table S1. Kaplan-Meier output for Figure 1

Overall Survival (Days)	Youngest 20%	Oldest 20%
0	100	100
8	99.53488372	
10	99.06976744	
15	98.60465116	
20	98.13953488	
29		99.53488372
76	98.13953488	
100	97.67220377	
162		99.53488372
172	97.20487265	
183	96.73754153	
197		99.0675838
199		98.60028387
205	96.73754153	
232		98.13298395
234	96.26794181	
240		97.66568403
245		97.1983841
257	95.7983421	
276		96.73108418
282	95.32874238	
287		96.26378426
322		95.79648433
323	94.85914266	95.32918441
339	94.38954295	
345		94.86188449
369		94.39458456
376		93.92728464
411		93.45998471
416		92.52538487
427		92.05808494
430		91.59078502
431		91.59078502
433		91.12108869
440		90.65139235
456		90.18169602
475	93.91994323	
481	93.45034352	
487		89.71199969
500		89.24230335
508		88.77260702
550		88.30291069
580	92.9807438	
586		87.83321435
588		87.36351802
593		86.89382169
603		86.42412535
609		86.42412535
617		85.95186237
618		85.47959939
637	92.9807438	
649		85.00733641
650		84.53507343
679		84.06281045
724		83.59054747
732		83.11828449
747		83.11828449

Comparison of Survival Curves		
Log-rank (Mantel-Cox) test		
Chi square	81.19	
df	1	
P value	<0.0001	
P value summary	****	
Are the survival curves sig different?	Yes	
Gehan-Breslow-Wilcoxon test		
Chi square	62.9	
df	1	
P value	<0.0001	
P value summary	****	
Are the survival curves sig different?	Yes	
Median survival		
Youngest 20%	Undefined	
Oldest 20%	1836	
Hazard Ratio (Mantel-Haenszel)	A/B	B/A
Ratio (and its reciprocal)	0.1977	5.059
95% CI of ratio	0.1389 to 0.2813	3.555 to 7.198
Hazard Ratio (logrank)	A/B	B/A
Ratio (and its reciprocal)	0.1463	6.835
95% CI of ratio	0.103 to 0.2078	4.812 to 9.708

761		82.64332287
767		82.16836124
780		82.16836124
790		81.69063821
791		81.21291518
803		80.73519215
817		80.25746912
819	92.50876033	
822		80.25746912
826		79.77688547
833		79.29630182
836		78.81571818
837		78.33513453
843		77.85455088
851	92.50876033	
864	92.50876033	
875		77.37396723
885	92.03191105	
903	92.03191105	
906	92.03191105	
916		76.89338359
919		76.41279994
922		75.93221629
934		75.45163264
953		75.45163264
959	92.03191105	
972		74.96796833
997	92.03191105	
1001		74.48430402
1008		74.48430402
1013		74.48430402
1030		74.48430402
1033		73.49775695
1060	92.03191105	
1063	92.03191105	
1077		73.49775695
1079		73.00115048
1083		72.50454402
1087		72.50454402
1095	92.03191105	
1098	92.03191105	
1102		72.00451268
1106		72.00451268
1107		71.50098462
1111	92.03191105	71.50098462
1112		71.50098462
1114	92.03191105	70.9902633
1116		70.47954198
1119	91.52900443	
1131		69.96882066
1140		69.96882066
1160		69.45434404
1168		68.93986742
1169		68.42152255
1180		68.42152255
1193		67.89922085
1195	91.52900443	
1203		67.89922085

1211		67.3728703
1212		66.84651975
1214		66.84651975
1216		66.84651975
1219	91.52900443	
1223		66.31174759
1227	91.52900443	
1229	91.52900443	
1230		65.77697544
1232	91.52900443	
1235		65.24220328
1261		64.70743112
1281		64.17265896
1294	91.52900443	
1297		63.10311465
1301	91.52900443	
1306		62.56834249
1319		62.03357033
1321		61.49879817
1324	91.52900443	
1337		61.49879817
1342		60.95933503
1350		60.95933503
1354		60.95933503
1365		60.95933503
1372		60.95933503
1378		60.40007507
1379		60.40007507
1392	91.52900443	
1409		59.83558839
1414	91.52900443	
1418		59.83558839
1438		59.26572565
1446	91.52900443	
1452	91.52900443	
1469		59.26572565
1479		58.69033025
1485	91.52900443	
1488		58.69033025
1512		58.69033025
1513	91.52900443	
1517	91.52900443	
1540		58.10342695
1544		57.51652365
1549	91.52900443	56.92962034
1561		56.34271704
1570		55.75581374
1572		55.16891044
1577	91.52900443	
1578		55.16891044
1590		54.56924837
1592		54.56924837
1593	91.52900443	
1596		54.56924837
1607		54.56924837
1608		54.56924837
1609	91.52900443	
1611		54.56924837

1628	91.52900443	54.56924837
1636		53.92725721
1638	91.52900443	
1652	91.52900443	
1659	91.52900443	
1666	91.52900443	
1668	91.52900443	
1682		53.28526605
1683	91.52900443	
1696		52.64327489
1706	91.52900443	
1725	91.52900443	
1728		52.64327489
1732	91.52900443	
1739		52.64327489
1740	91.52900443	
1743		51.97690433
1744	91.52900443	
1765		51.31053376
1766		50.64416319
1769	91.52900443	
1777	91.52900443	
1783	91.52900443	
1794	91.52900443	
1796		50.64416319
1807		50.64416319
1811		50.64416319
1812	91.52900443	50.64416319
1823	91.52900443	
1836		49.94077203
1841	91.52900443	
1857		49.94077203
1861	91.52900443	
1863		49.94077203
1867	91.52900443	
1870	91.52900443	
1871	91.52900443	
1877	91.52900443	
1880	91.52900443	
1883	91.52900443	
1886		49.94077203
1888	91.52900443	
1905	91.52900443	
1906	91.52900443	
1918		49.94077203
1919	91.52900443	
1921	91.52900443	
1935	91.52900443	
1936		49.94077203
1939	91.52900443	
1940		49.94077203
1945		49.94077203
1952		49.94077203
1955	91.52900443	
1958	91.52900443	
1959	91.52900443	
1977	91.52900443	
1980		49.94077203

1998	91.52900443	
2010	91.52900443	
2026	91.52900443	
2031	91.52900443	
2037	91.52900443	
2039		49.13527571
2041		49.13527571
2047	91.52900443	
2058	91.52900443	
2064	91.52900443	
2065	91.52900443	
2067	91.52900443	
2083	91.52900443	
2084	91.52900443	
2089	91.52900443	
2091	91.52900443	
2095	91.52900443	
2144	91.52900443	
2147		49.13527571
2152	91.52900443	
2155	91.52900443	
2176		49.13527571
2178		49.13527571
2183	91.52900443	
2185	91.52900443	
2187	91.52900443	
2191	91.52900443	
2192		49.13527571
2199	91.52900443	
2200	91.52900443	
2207	91.52900443	
2208	91.52900443	
2222	91.52900443	
2226	91.52900443	
2227		49.13527571
2230		49.13527571
2233	91.52900443	
2240	91.52900443	
2241	91.52900443	
2248	91.52900443	
2252		49.13527571
2255	91.52900443	
2272	91.52900443	
2274	91.52900443	
2277	91.52900443	
2278	91.52900443	
2281	91.52900443	
2282		49.13527571
2283	91.52900443	
2285	90.38489187	
2290	90.38489187	
2291	90.38489187	
2304	90.38489187	
2306	90.38489187	
2319	90.38489187	
2325	90.38489187	
2332	90.38489187	
2333	90.38489187	

2336	90.38489187	
2343	90.38489187	
2344	90.38489187	
2347	90.38489187	
2351		49.13527571
2357	90.38489187	
2359	90.38489187	
2365		49.13527571
2373	90.38489187	
2376	90.38489187	
2381	90.38489187	
2384		48.1525702
2387	90.38489187	
2388		48.1525702
2394	90.38489187	
2408	90.38489187	
2413		48.1525702
2415		48.1525702
2416	90.38489187	
2423	90.38489187	
2433		47.10577519
2435	90.38489187	
2437	90.38489187	
2438	90.38489187	
2444		47.10577519
2449	90.38489187	
2451	90.38489187	
2459		47.10577519
2484		47.10577519
2489	90.38489187	
2491		45.98420912
2493	90.38489187	
2499	90.38489187	
2510	90.38489187	
2511		45.98420912
2522	90.38489187	
2523		44.83460389
2526	90.38489187	
2529		44.83460389
2551	90.38489187	
2552	90.38489187	
2558		44.83460389
2586		44.83460389
2597		44.83460389
2604	90.38489187	
2606		44.83460389
2611	90.38489187	
2613	90.38489187	
2616	90.38489187	
2617	90.38489187	
2620		44.83460389
2636		43.47597953
2642		43.47597953
2650	90.38489187	
2659	90.38489187	
2664	90.38489187	
2679	90.38489187	
2691	90.38489187	

2711	90.38489187	
2725	90.38489187	
2727	90.38489187	
2744	90.38489187	43.47597953
2758		43.47597953
2772		43.47597953
2776		43.47597953
2810	90.38489187	
2812	90.38489187	
2829		43.47597953
2836		41.80382647
2846		40.13167341
2873	90.38489187	
2889		40.13167341
2916	90.38489187	
2958		40.13167341
2959		40.13167341
2962		40.13167341
2991		40.13167341
3122	90.38489187	
3220		40.13167341
3264	90.38489187	
3334	90.38489187	
3341	90.38489187	
3434	90.38489187	
3467		40.13167341
3563		40.13167341
3678	90.38489187	
3691		37.62344382
3722		37.62344382
3723	90.38489187	
3726		37.62344382
3792	90.38489187	
3824		37.62344382
3828	90.38489187	
3866		37.62344382
3929		37.62344382
4011	90.38489187	
4012		37.62344382
4067	90.38489187	
4136	90.38489187	
4191	90.38489187	
4263	90.38489187	
4287		37.62344382
4318	90.38489187	
4368	90.38489187	
4454	90.38489187	
4596		37.62344382
4614	90.38489187	
4670		37.62344382
4679		37.62344382
4683		37.62344382
4707		37.62344382
4715		37.62344382
4835	90.38489187	
4947		37.62344382
4998	90.38489187	
5048	90.38489187	

5216	90.38489187	37.62344382

Barcodes for Youngest 20% of pediatric NBL patients

TARGET-30-PATPXF
TARGET-30-PASTIJ
TARGET-30-PASJUU
TARGET-30-PATXTF
TARGET-30-PARZHA
TARGET-30-PASUML
TARGET-30-PAMLNB
TARGET-30-PASSWW
TARGET-30-PATHKB
TARGET-30-PATJXV
TARGET-30-PASLMN
TARGET-30-PASNML
TARGET-30-PAURYJ
TARGET-30-PASAVJ
TARGET-30-PALKXJ
TARGET-30-PATMAW
TARGET-30-PAPTDH
TARGET-30-PALWSJ
TARGET-30-PASGGI
TARGET-30-PAUHIK
TARGET-30-PALZHF
TARGET-30-PASCFC
TARGET-30-PALEBK
TARGET-30-PALVDR
TARGET-30-PASUEZ
TARGET-30-PALXUM
TARGET-30-PASWKI
TARGET-30-PAKZRH
TARGET-30-PASTXV
TARGET-30-PAVDYS
TARGET-30-PASMDM
TARGET-30-PASZTV
TARGET-30-PAKGGW
TARGET-30-PASZKE
TARGET-30-PASCFA
TARGET-30-PASBZV
TARGET-30-PARBAJ
TARGET-30-PAIXHH
TARGET-30-PAMKBM
TARGET-30-PARNNC
TARGET-30-PASFDV
TARGET-30-PARCET
TARGET-30-PASLGM
TARGET-30-PALXTE
TARGET-30-PASRIB
TARGET-30-PALPIN
TARGET-30-PASBDN
TARGET-30-PASFNF
TARGET-30-PATWED
TARGET-30-PASMRC
TARGET-30-PASFIC

TARGET-30-PATAYJ
TARGET-30-PARJVP
TARGET-30-PARWBC
TARGET-30-PAUZTF
TARGET-30-PAPICY
TARGET-30-PASNMJ
TARGET-30-PATGJU
TARGET-30-PARFRE
TARGET-30-PASCTR
TARGET-30-PAIXFZ
TARGET-30-PASPBZ
TARGET-30-PATAFE
TARGET-30-PARTCE
TARGET-30-PALEAC
TARGET-30-PALWXP
TARGET-30-PASBJY
TARGET-30-PATUNK
TARGET-30-PANZRV
TARGET-30-PASJZC
TARGET-30-PASREY
TARGET-30-PAKRGC
TARGET-30-PAKPNF
TARGET-30-PASMUB
TARGET-30-PARXVA
TARGET-30-PASEAR
TARGET-30-PAMVCL
TARGET-30-PATBJI
TARGET-30-PASPSE
TARGET-30-PASSUU
TARGET-30-PARPUF
TARGET-30-PASWVE
TARGET-30-PASPBY
TARGET-30-PASKCS
TARGET-30-PASKYH
TARGET-30-PAKYRJ
TARGET-30-PASEVK
TARGET-30-PAMAJT
TARGET-30-PASTSY
TARGET-30-PATSXC
TARGET-30-PASTWY
TARGET-30-PARWTY
TARGET-30-PALGFF
TARGET-30-PARTYI
TARGET-30-PAMZRP
TARGET-30-PARVMU
TARGET-30-PASZJB
TARGET-30-PATCJF
TARGET-30-PASPTF
TARGET-30-PASCWD
TARGET-30-PATUNX
TARGET-30-PASATF
TARGET-30-PASLSS

TARGET-30-PATJPI
TARGET-30-PAUNST
TARGET-30-PALWRG
TARGET-30-PASFXC
TARGET-30-PAUWXY
TARGET-30-PAUVVE
TARGET-30-PASFCG
TARGET-30-PAUWEV
TARGET-30-PASAJY
TARGET-30-PASMDG
TARGET-30-PASFDJ
TARGET-30-PAKHAV
TARGET-30-PATRXC
TARGET-30-PAKSWB
TARGET-30-PASGNT
TARGET-30-PASVKE
TARGET-30-PAUXFZ
TARGET-30-PASCRK
TARGET-30-PAUBRR
TARGET-30-PATFTY
TARGET-30-PASFEV
TARGET-30-PATKGB
TARGET-30-PATJET
TARGET-30-PATBRX
TARGET-30-PASEWX
TARGET-30-PARVIH
TARGET-30-PATAKH
TARGET-30-PASCFD
TARGET-30-PASFWL
TARGET-30-PAUZMG
TARGET-30-PATFMU
TARGET-30-PAMXYU
TARGET-30-PATZRF
TARGET-30-PASRLC
TARGET-30-PAUGWT
TARGET-30-PATFTR
TARGET-30-PAUIHH
TARGET-30-PALVUC
TARGET-30-PAUZRC
TARGET-30-PAMZMJ
TARGET-30-PAUGRP
TARGET-30-PATDVF
TARGET-30-PARYEH
TARGET-30-PATDCJ
TARGET-30-PALFSE
TARGET-30-PAIVMJ
TARGET-30-PATEWM
TARGET-30-PATNXK
TARGET-30-PASTTX
TARGET-30-PARXLN
TARGET-30-PALVVH
TARGET-30-PATTEF

TARGET-30-PAURCG
TARGET-30-PASUCU
TARGET-30-PASSZI
TARGET-30-PATWZB
TARGET-30-PARTPF
TARGET-30-PARZCJ
TARGET-30-PAIWHU
TARGET-30-PASSJK
TARGET-30-PASXCG
TARGET-30-PARXMH
TARGET-30-PASKRA
TARGET-30-PASPIK
TARGET-30-PARUGX
TARGET-30-PATHYK
TARGET-30-PATBKX
TARGET-30-PARZBH
TARGET-30-PASJWU
TARGET-30-PASTMW
TARGET-30-PASFKX
TARGET-30-PAIXLC
TARGET-30-PATUPR
TARGET-30-PASAAB
TARGET-30-PASSII
TARGET-30-PATTMM
TARGET-30-PASBPN
TARGET-30-PARXAX
TARGET-30-PASDDP
TARGET-30-PASLYF
TARGET-30-PALKKV
TARGET-30-PARSZV
TARGET-30-PASYJF
TARGET-30-PASNVM
TARGET-30-PARXPD
TARGET-30-PASWNG
TARGET-30-PATMTX
TARGET-30-PATVMF
TARGET-30-PARXLL
TARGET-30-PAIWZA
TARGET-30-PAIDJS
TARGET-30-PAKGCI
TARGET-30-PATPNR
TARGET-30-PAMAFV
TARGET-30-PAULVH
TARGET-30-PASVYV
TARGET-30-PALPSY
TARGET-30-PAMKHK
TARGET-30-PAMZSH
TARGET-30-PATUNZ
TARGET-30-PASNPG
TARGET-30-PATBHY
TARGET-30-PAUWED
TARGET-30-PATJZC

TARGET-30-PAUGJI
TARGET-30-PATYWM
TARGET-30-PATYIL
TARGET-30-PASYYM
TARGET-30-PASKSX
TARGET-30-PARYUK
TARGET-30-PAUBYW
TARGET-30-PATAFI

Barcodes for Oldest 20% of pediatric NBL patients

TARGET-30-PALXRL
TARGET-30-PAUDVA
TARGET-30-PAMNLH
TARGET-30-PATVWA
TARGET-30-PALTYB
TARGET-30-PAIFCS
TARGET-30-PASBEN
TARGET-30-PALWVJ
TARGET-30-PAMMYR
TARGET-30-PAMVRA
TARGET-30-PARFWB
TARGET-30-PATCJP
TARGET-30-PALZSL
TARGET-30-PASLIH
TARGET-30-PADKFS
TARGET-30-PAPRTD
TARGET-30-PATPJD
TARGET-30-PATMXC
TARGET-30-PARGZK
TARGET-30-PATEUC
TARGET-30-PADPMU
TARGET-30-PATJHU
TARGET-30-PATYMS
TARGET-30-PAPUEB
TARGET-30-PARLMK
TARGET-30-PATXHC
TARGET-30-PATWTW
TARGET-30-PAINRC
TARGET-30-PARASL
TARGET-30-PALNMX
TARGET-30-PACVNB
TARGET-30-PAUGVZ
TARGET-30-PARKGJ
TARGET-30-PASCUF
TARGET-30-PAMFBR
TARGET-30-PAUPDY
TARGET-30-PAUELT
TARGET-30-PALHVD
TARGET-30-PALJVX

TARGET-30-PANGTS
TARGET-30-PARNCW
TARGET-30-PASANE
TARGET-30-PASBMW
TARGET-30-PANCYF
TARGET-30-PASGDB
TARGET-30-PATDXC
TARGET-30-PASCIX
TARGET-30-PADUKP
TARGET-30-PAMMXF
TARGET-30-PASZFX
TARGET-30-PAMSXU
TARGET-30-PAVCJZ
TARGET-30-PADUWR
TARGET-30-PASALE
TARGET-30-PAILNU
TARGET-30-PAUXUP
TARGET-30-PALZRB
TARGET-30-PAITZV
TARGET-30-PARZMY
TARGET-30-PARGUX
TARGET-30-PANXFT
TARGET-30-PAPBZI
TARGET-30-PAIEIF
TARGET-30-PANZVU
TARGET-30-PAPVEB
TARGET-30-PADKYP
TARGET-30-PARXXC
TARGET-30-PAUMUC
TARGET-30-PANRRW
TARGET-30-PAPPKJ
TARGET-30-PAKIAP
TARGET-30-PADKNE
TARGET-30-PANZPV
TARGET-30-PALAKM
TARGET-30-PATYMK
TARGET-30-PAAPFA
TARGET-30-PASAZJ
TARGET-30-PASMJG
TARGET-30-PARUXY
TARGET-30-PAUBGW
TARGET-30-PADKFU
TARGET-30-PARSEA
TARGET-30-PADLES
TARGET-30-PATNWL
TARGET-30-PAPBFP
TARGET-30-PALJZV
TARGET-30-PAKPAL
TARGET-30-PALVKK
TARGET-30-PADIEY
TARGET-30-PASGEE
TARGET-30-PADMGA

TARGET-30-PADLIC
TARGET-30-PAUUHD
TARGET-30-PAPBJE
TARGET-30-PATAAV
TARGET-30-PASLDM
TARGET-30-PASTCN
TARGET-30-PARSRJ
TARGET-30-PASAZZ
TARGET-30-PAUCKF
TARGET-30-PADIHC
TARGET-30-PATYDC
TARGET-30-PAMNYX
TARGET-30-PARDYU
TARGET-30-PAPUWY
TARGET-30-PAPVFD
TARGET-30-PARUCL
TARGET-30-PASGPY
TARGET-30-PANLET
TARGET-30-PAKANZ
TARGET-30-PAMVLG
TARGET-30-PADVWW
TARGET-30-PAPTJB
TARGET-30-PADXBB
TARGET-30-PADPCI
TARGET-30-PAIFXV
TARGET-30-PAPCTS
TARGET-30-PAUGZD
TARGET-30-PASYLD
TARGET-30-PATNCI
TARGET-30-PATCKU
TARGET-30-PADWEN
TARGET-30-PASPVZ
TARGET-30-PATLCM
TARGET-30-PARDIW
TARGET-30-PAVEKN
TARGET-30-PARDCK
TARGET-30-PAUYDE
TARGET-30-PAREAG
TARGET-30-PASUXH
TARGET-30-PATHVK
TARGET-30-PASAJU
TARGET-30-PACSNL
TARGET-30-PADWVR
TARGET-30-PAUEYW
TARGET-30-PARURB
TARGET-30-PARYXW
TARGET-30-PAUHYY
TARGET-30-PANUIF
TARGET-30-PARGKK
TARGET-30-PARZIP
TARGET-30-PADXAS
TARGET-30-PARYNK

TARGET-30-PAPVRN
TARGET-30-PASAFG
TARGET-30-PANSBN
TARGET-30-PATYMZ
TARGET-30-PATNRI
TARGET-30-PARSBI
TARGET-30-PADWIM
TARGET-30-PARLTG
TARGET-30-PASWVY
TARGET-30-PARVNT
TARGET-30-PAUFVW
TARGET-30-PASSRS
TARGET-30-PAVCKK
TARGET-30-PASWVD
TARGET-30-PARMLF
TARGET-30-PAVEZM
TARGET-30-PATRHD
TARGET-30-PASWIJ
TARGET-30-PAPKXS
TARGET-30-PADLPZ
TARGET-30-PAUDDZ
TARGET-30-PATVDP
TARGET-30-PATTPL
TARGET-30-PAUIFL
TARGET-30-PATYPH
TARGET-30-PALRSD
TARGET-30-PAUHHW
TARGET-30-PATTPW
TARGET-30-PASYMX
TARGET-30-PANWRR
TARGET-30-PADWSM
TARGET-30-PAVAYF
TARGET-30-PATVDI
TARGET-30-PATMFL
TARGET-30-PASTKC
TARGET-30-PASGKP
TARGET-30-PADUYJ
TARGET-30-PARKNP
TARGET-30-PAJZUU
TARGET-30-PAPUNH
TARGET-30-PATKSX
TARGET-30-PATNKP
TARGET-30-PAURPL
TARGET-30-PAUUZU
TARGET-30-PASXNN
TARGET-30-PARBGP
TARGET-30-PARUPN
TARGET-30-PAUATG
TARGET-30-PACRZM
TARGET-30-PAUUGT
TARGET-30-PATXXI
TARGET-30-PAPREJ

TARGET-30-PAVCLI
TARGET-30-PATSKE
TARGET-30-PATRUL
TARGET-30-PATMJV
TARGET-30-PAUBEC
TARGET-30-PADLDA
TARGET-30-PATZBH
TARGET-30-PANRVJ
TARGET-30-PAMTUV
TARGET-30-PASMNT
TARGET-30-PATLNM
TARGET-30-PASPXU
TARGET-30-PASSRN
TARGET-30-PANXJL
TARGET-30-PAUXSZ
TARGET-30-PARVLK
TARGET-30-PATMSI
TARGET-30-PASTGH
TARGET-30-PASKJX
TARGET-30-PAUBHV

Table S2. Kaplan-Meier output summary for pediatric NBL diagnosis age survival curve, halves

Comparison of Survival Curves		
Log-rank (Mantel-Cox) test		
Chi square	30.17	
df	1	
P value	<0.0001	
P value summary	****	
Are the survival curves sig different?	Yes	
Gehan-Breslow-Wilcoxon test		
Chi square	13.98	
df	1	
P value	0.0002	
P value summary	***	
Are the survival curves sig different?	Yes	
Median survival		
Youngest 50%	Undefined	
Oldest 50%	2522	
Hazard Ratio (Mantel-Haenszel)	A/B	B/A
Ratio (and its reciprocal)	0.5756	1.737
95% CI of ratio	0.4726 to 0.701	1.427 to 2.116
Hazard Ratio (logrank)	A/B	B/A
Ratio (and its reciprocal)	0.5698	1.755
95% CI of ratio	0.468 to 0.6937	1.442 to 2.137

Table S3. 623 genes upregulated in older pediatric NBL patients (Pearson Correlation Coefficients, p-values)

gene	correlation	pvalue
POTED	0.434023595	7.34037E-13
ANKRD20A2	0.427043196	1.85446E-12
POTEC	0.418422157	5.65878E-12
ANKRD20A1	0.414394054	9.428E-12
POTEB	0.402057911	4.31758E-11
MET	0.391806554	1.45911E-10
NXT2	0.387474148	2.41091E-10
PPP4R4	0.385344388	3.07774E-10
ODF2	0.383562052	3.77051E-10
POTEM	0.380112376	5.56604E-10
CXorf49	0.37880624	6.44281E-10
OR52N1	0.378549996	6.62988E-10
MAGEA10	0.371251195	1.48274E-09
MAGEA9	0.367614988	2.19781E-09
MAGEC1	0.359615954	5.13545E-09
POTEG	0.357743681	6.243E-09
XAGE1A	0.353758956	9.4207E-09
IGHG1	0.352612107	1.05939E-08
SLCO1A2	0.350171198	1.3579E-08
POTEKP	0.348144682	1.66603E-08
SLC6A10P	0.338305088	4.40609E-08
OR4M1	0.336811747	5.09195E-08
IGLL5	0.331958925	8.10546E-08
MKRN3	0.331395955	8.55015E-08
OR4M2	0.32982916	9.91485E-08
OR4Q3	0.325265321	1.51906E-07
OR52E6	0.325232681	1.52366E-07
OR52E8	0.322145319	2.02537E-07
MUC15	0.321474502	2.15368E-07
POTEF	0.321315034	2.18531E-07
POTEH	0.314504494	4.04162E-07
IGKC	0.313765397	4.31657E-07
POTEE	0.311202196	5.41587E-07
FAM182B	0.310831873	5.59535E-07
TPTE	0.310757174	5.63224E-07
OR4N4	0.308824713	6.67165E-07
TEKT4	0.306670973	8.04622E-07
AP1G2	0.303827704	1.02804E-06
MAGEA11	0.303010234	1.10255E-06
1-Mar	0.302147005	1.18683E-06
OR56A1	0.301261343	1.27968E-06
SLC5A12	0.295839557	2.01846E-06
TRDN	0.295364117	2.09983E-06
ZNF101	0.29510903	2.14477E-06
LOC100508538	0.29497189	2.2746E-06
GNGT1	0.294210675	2.31045E-06

MBIP	0.293941558	2.36242E-06
OR52H1	0.292367296	2.68947E-06
FAM58A	0.291768174	2.82494E-06
TUBA3C	0.291019566	3.00336E-06
HCCS	0.290831191	3.04992E-06
MKRN9P	0.287883915	3.87437E-06
NAA11	0.287202845	4.09306E-06
CCNB3	0.287183313	4.0995E-06
SLC35F3	0.284334697	5.14982E-06
TP53TG3	0.284321665	5.15517E-06
FSIP1	0.284175059	5.21569E-06
CHMP4A	0.283077696	5.69064E-06
SPATA17	0.279907599	7.30475E-06
IGFL2	0.27823707	8.32181E-06
STK33	0.276031558	9.87186E-06
WWOX	0.276011631	9.88704E-06
CXADRP3	0.275059811	1.06385E-05
OR52N5	0.274537683	1.10734E-05
TRAPPC6B	0.273295635	1.21769E-05
PAGE5	0.272847096	1.26004E-05
IGHM	0.271055651	1.44353E-05
FAM117A	0.270186004	1.54148E-05
ZNF467	0.269177019	1.66301E-05
PNMA5	0.267891883	1.83098E-05
OR56A4	0.267566761	1.87595E-05
USP6	0.267337822	1.90824E-05
XCL1	0.266806882	1.98517E-05
VSIG1	0.265691562	2.15643E-05
OR4N2	0.26501381	2.26723E-05
OR52L1	0.264963846	2.2756E-05
TRIM5	0.264307072	2.38849E-05
DNAH2	0.263427452	2.54799E-05
LIN52	0.263168658	2.5968E-05
CGRRF1	0.260856513	3.07388E-05
IGJ	0.259153939	3.47688E-05
NRN1	0.258625956	3.61166E-05
YOD1	0.258406575	3.6691E-05
PAGE2B	0.257057279	4.04178E-05
VMA21	0.256817524	4.11163E-05
SLC7A6OS	0.256386962	4.23993E-05
KATNAL2	0.256247675	4.28224E-05
PPP6C	0.255928282	4.38076E-05
FAM182A	0.255510426	4.51288E-05
DNAH6	0.255372588	4.55729E-05
PPP1CA	0.255242835	4.59946E-05
NUP62CL	0.255233569	4.60248E-05
IQCH	0.254568644	4.82457E-05

TOX4	0.253494148	5.20492E-05
TGIF2LX	0.253335651	5.26336E-05
TPI1P2	0.253128035	5.34085E-05
NEK2	0.252969438	5.40076E-05
LAGE3	0.252931559	5.41517E-05
FRG1B	0.252861785	5.44179E-05
FBXW7	0.252726687	5.49369E-05
ELAC1	0.252166075	5.7141E-05
LOC256021	0.251623137	6.14405E-05
PIM2	0.251268348	6.08445E-05
NUCB2	0.250294719	6.5116E-05
TAF9B	0.249821953	6.72904E-05
AAGAB	0.248303808	7.47436E-05
IER3IP1	0.247966548	7.65015E-05
MX2	0.247932947	7.66787E-05
ARPC5	0.247309955	8.00358E-05
C18orf25	0.246797309	8.29014E-05
FAM104A	0.246752967	8.31537E-05
DDX5	0.246723784	8.33202E-05
TMEM55B	0.246543244	8.4357E-05
MAGEA8	0.245641686	8.97183E-05
CDH7	0.245264136	9.20572E-05
STK4	0.245026233	9.35602E-05
FBXO33	0.244912134	9.42892E-05
LOC646864	0.244748377	9.85153E-05
EFCAB6	0.244543745	9.66795E-05
KIAA0125	0.242976766	0.000107497
GCC1	0.242925801	0.000107867
FAM153C	0.242596884	0.000110285
C11orf82	0.242428683	0.00011518
WFDC2	0.241908299	0.000115512
LGSN	0.241319802	0.000120163
ZBTB6	0.241036455	0.000122465
NAA10	0.24032911	0.000128391
COL22A1	0.239865555	0.00013242
TOR1A	0.239473193	0.000135922
TAS2R38	0.23850137	0.000144973
CXCL13	0.237984957	0.000150009
FAM153A	0.237601045	0.000153858
SURF1	0.2373876	0.000156038
FAM153B	0.23709501	0.000159074
ATP6V1G1	0.236574763	0.000164608
SDAD1	0.236102264	0.000169789
CAPZA2	0.236017403	0.000170736
C9orf100	0.235821328	0.000178284
TCEB3B	0.235781929	0.000173388
FUT8	0.235632013	0.000175097

AKIRIN1	0.234900828	0.000183659
TIFA	0.234651415	0.000186668
GTSF1	0.234462918	0.000188973
SDR39U1	0.234332336	0.000190585
BCL7B	0.233559373	0.000200393
ARL6IP1	0.233506806	0.000201077
ARF6	0.233149666	0.000205781
TRUB2	0.231945302	0.000222414
KBTBD8	0.231367322	0.000230833
TMEM229B	0.231335532	0.000231305
TSPAN33	0.231230692	0.000232867
VAMP7	0.231121801	0.000234499
PRPS1	0.230868446	0.000238339
TRAT1	0.230801184	0.000239368
TCEAL2	0.230793444	0.000239487
TRIM37	0.230616406	0.000242217
MAGEA4	0.230414503	0.000245366
KLK1	0.230064843	0.000250909
THAP5	0.228621788	0.000275046
HLA-DPB2	0.227914837	0.000287645
UBLCP1	0.227118486	0.000302479
CCDC144NL	0.227065996	0.000303481
GAL3ST4	0.226986504	0.000305005
TMED10	0.22678685	0.000308864
EXOC5	0.226451151	0.000315455
C17orf47	0.226322309	0.000318019
TYRP1	0.225855612	0.000327471
GLE1	0.225683869	0.000331014
ADAT1	0.225398584	0.000336979
COPE	0.225291524	0.000339243
DRAP1	0.224724698	0.000351468
CCNA1	0.224175524	0.000363701
FEV	0.224145673	0.000364377
TCEAL1	0.224142563	0.000364448
CSAG2	0.222523811	0.000414025
STX17	0.222523257	0.000402935
POLR2C	0.222195589	0.000411169
PER3	0.222194427	0.000411198
PDCD6	0.222097144	0.000413672
RPL28	0.221609684	0.000426281
IMPA1	0.221409676	0.000431557
DHX40	0.221215336	0.000436741
ZNF812	0.221083936	0.000440279
CCL28	0.220950373	0.000443903
NUBPL	0.220564608	0.000454524
PDK3	0.220530709	0.000455468
CECR7	0.220395638	0.00045925

ANKRD30B	0.220276136	0.00046262
ADCK1	0.220222059	0.000464152
ABHD3	0.220126086	0.000466884
ZNF81	0.219289864	0.000491321
SP140	0.218966903	0.000501072
CD3G	0.218952208	0.00050152
TCEAL5	0.218845523	0.000504784
FKBP3	0.218761144	0.000507378
RSPO4	0.21867553	0.000510024
ZNF572	0.218651299	0.000510775
POP4	0.218420313	0.000517986
DYRK1A	0.218418179	0.000518053
TCEB3C	0.218332463	0.000520753
RAP2C	0.218304582	0.000521634
LOC170425	0.217887224	0.000549138
LOC646853	0.217679913	0.000556041
FAM95B1	0.217539714	0.00054635
C7orf49	0.217475691	0.000548467
TPTE2	0.21747266	0.000548568
CD2	0.217137322	0.000559784
FAM120AOS	0.216774977	0.000572142
RUNX2	0.216567646	0.000579327
PSME1	0.216358601	0.000586655
PLRG1	0.216306141	0.000588507
SPAG1	0.216300399	0.00058871
STYX	0.215725163	0.000609387
MAEL	0.215717231	0.000609677
IL17RA	0.215708178	0.000610008
PEPD	0.215616222	0.000613378
CDC42SE2	0.215381512	0.000622058
H2AFB1	0.215287907	0.000625551
RUNDC1	0.215057376	0.000634232
PGRMC1	0.215039262	0.000634919
ZBTB25	0.215014201	0.00063587
LOC440288	0.21469062	0.000664931
UBQLNL	0.214590307	0.000652163
SLAMF6	0.214388888	0.000660039
SLC6A8	0.214048502	0.000673549
SEPHS2	0.214044791	0.000673697
LOC283911	0.214002735	0.000692632
AFF2	0.213606304	0.000691481
RIBC2	0.213460786	0.000697478
MYOM2	0.213386472	0.000700559
C17orf67	0.213230625	0.000707062
LAX1	0.212838406	0.000723674
ING1	0.212535198	0.000736762
CXCL9	0.212414203	0.000742045

RAB19	0.212141883	0.000754065
SMU1	0.212111949	0.000755397
PALB2	0.212072318	0.000757164
NXF2	0.211943572	0.000762931
CTDP1	0.211660146	0.000775769
HLA-DOA	0.211523849	0.000782013
CD27	0.210732825	0.000819179
THAP9	0.21072218	0.00081969
PSME2	0.210679734	0.000821731
TEX14	0.210653175	0.00082301
VAC14	0.210603146	0.000825425
MST4	0.210357891	0.000858041
DCAF11	0.209834297	0.000863368
RASGRP1	0.20960814	0.000874831
TPST2	0.209535342	0.000878551
UBE2W	0.209510773	0.000879809
NFATC3	0.20893444	0.000909815
TNFRSF10C	0.208697489	0.000922422
NARS	0.208691577	0.000922739
RCBTB1	0.208636257	0.000925706
GBP5	0.208527018	0.000931592
NXF5	0.208466737	0.000934854
MRPS22	0.208424	0.000937174
DNAJC2	0.208122727	0.000953675
FYB	0.208083647	0.000955835
PDP2	0.207921608	0.000964839
CLDN15	0.207136729	0.001009568
NDUFA2	0.206883514	0.001024401
C6orf120	0.206741346	0.001032816
LOC642006	0.206598409	0.00106618
MBD2	0.206569824	0.001043054
PTPRC	0.206447209	0.001050429
TESK2	0.206315385	0.001058412
LUZP6	0.206290032	0.001059954
TFPT	0.205791309	0.001090704
SOCS4	0.2056669	0.001098502
ZNF717	0.205640759	0.001100146
CXXC1	0.205479188	0.001110364
USP17L5	0.205392399	0.001115888
JKAMP	0.205270807	0.00112367
ZKSCAN5	0.205229253	0.001126341
NAA38	0.205102985	0.001134492
GVINP1	0.205022703	0.001139703
EIF3G	0.204978826	0.001142561
H3F3C	0.204827552	0.001152462
PPP6R1	0.204639777	0.001164862
GK	0.204543363	0.001171277

TCEAL3	0.2045233	0.001172616
NUDT4	0.204492893	0.001174648
CXCL11	0.204439947	0.001178193
NBPF6	0.204384771	0.001181899
DNTTIP1	0.204350895	0.001184179
LOC441454	0.204306172	0.001214911
GTF3C4	0.203397092	0.001250066
CCDC113	0.202945486	0.001282421
TNFSF8	0.202880182	0.001287162
FAM46C	0.202741035	0.00129732
DHRS4L2	0.20264167	0.001304618
ALG2	0.202583685	0.001308894
UBXN11	0.202120197	0.00134354
PTPN1	0.202005673	0.001352229
OTUD5	0.201957514	0.001355899
ACTN2	0.201950878	0.001356405
TRMT6	0.201860306	0.001363333
CCDC40	0.201642683	0.001380112
SETX	0.201621499	0.001381756
BEX5	0.201276859	0.001408745
UBXN10	0.201161723	0.001417869
TMEM209	0.201084175	0.001424045
TACO1	0.201076927	0.001424623
CD96	0.200983551	0.001432095
RUNX3	0.200981871	0.001432229
RHOG	0.200973545	0.001432897
ZNF354A	0.200971155	0.001433089
NEDD8	0.200964726	0.001433605
POLR2B	0.200885987	0.00143994
CNDP2	0.200824896	0.001444872
CCDC47	0.20079031	0.001447671
TAGAP	0.200762885	0.001449894
POU2AF1	0.200591393	0.001463867
MAGEF1	0.20046785	0.001474009
GPR18	0.200441929	0.001476145
CARD11	0.200426433	0.001477424
LRRIQ1	0.200250257	0.001492029
YIPF6	0.200139785	0.001501255
NAA30	0.200132148	0.001501895
BRMS1L	0.199978804	0.001514792
FAM199X	0.199854367	0.001525333
ANKRD26P1	0.199835909	0.001526902
WDR45	0.199636676	0.001543935
MID2	0.199539243	0.001552328
MOCS3	0.199420442	0.001562618
ZNF567	0.199176224	0.001583967
SURF4	0.198974799	0.001601775

FAM7A1	0.198838709	0.001649673
RRS1	0.198834676	0.001614272
GZMK	0.198832186	0.001614495
GLUD2	0.19859742	0.001635635
POU6F2	0.198594457	0.001635903
POTEA	0.198491048	0.001681632
ZNF79	0.198481989	0.001646122
SNX20	0.198318709	0.00166106
FGF10	0.198266614	0.001665853
SLFN12L	0.198075978	0.001683497
EVI2B	0.198038	0.001687033
SRRM5	0.197923029	0.001697777
SYNJ2BP	0.197766934	0.001712465
REPIN1	0.197658429	0.001722743
LOC100288637	0.197429208	0.001782777
CCDC41	0.197348821	0.001790656
ERP44	0.197165204	0.001770179
C2orf14	0.197124842	0.001812778
ZNF92	0.197116186	0.001774957
AK7	0.196796906	0.001806374
AIM2	0.196782598	0.001807794
MAPKAPK2	0.196764148	0.001809626
STK17B	0.196436231	0.001842474
RPS6KA3	0.196326282	0.00185361
PPP2R4	0.19617595	0.001868934
THUMPD1	0.196150002	0.001871591
GAGE1	0.195902899	0.001897065
HSF5	0.195859178	0.001901605
SIRT7	0.195846845	0.001902887
FAM183A	0.195788795	0.001908934
LOC283710	0.195681527	0.001961417
RBM20	0.195668896	0.001921479
SMARCD3	0.195654122	0.00192303
TCEAL4	0.195627527	0.001925825
SMNDC1	0.195523727	0.001936769
DUOXA1	0.195432361	0.001946449
ZNF655	0.195327039	0.001957662
BMP7	0.195289662	0.001961656
SERP1	0.195190532	0.001972284
TRAF3IP3	0.195117731	0.001980122
ST6GALNAC6	0.194786833	0.002016108
TAF10	0.194761095	0.002018932
BLNK	0.194651788	0.002030966
MLYCD	0.194361115	0.002063284
RBM41	0.194200275	0.002081368
FXYD5	0.194152153	0.002086807
PRKAR1A	0.194040689	0.002099454

TRIM22	0.193894108	0.002116192
BOLA2	0.193845089	0.002121816
F8A1	0.193685028	0.002140277
FAM25A	0.193670461	0.002141964
SUPT16H	0.193507048	0.002160976
PSMA6	0.193320417	0.002182876
ASTE1	0.193272336	0.002188551
MUC1	0.193269867	0.002188843
CETN2	0.193234603	0.002193014
ZDHHC23	0.193177261	0.002199812
CPEB1	0.193152206	0.002202789
LOC100506934	0.193146893	0.002249606
TC2N	0.193114141	0.002207318
TIGD6	0.193106482	0.00220823
RAB4B	0.193012397	0.002219465
MAGEC2	0.192996151	0.00222141
CYP4X1	0.19296651	0.002224963
ICOS	0.192932253	0.002229076
CSTA	0.192682142	0.002259314
IL7R	0.192623572	0.002266449
SH2D1A	0.192596201	0.00226979
TOB1	0.192553501	0.002275012
LRRC18	0.192542104	0.002276407
TUBA3E	0.192432461	0.002289872
G6PD	0.192421884	0.002291175
SLAMF7	0.192409546	0.002292696
RRM2B	0.192293872	0.002306996
ADAM28	0.192194631	0.00231933
HVCN1	0.192037985	0.00233892
RCAN3	0.19196453	0.002348158
MALT1	0.191956934	0.002349115
ERAP2	0.19176692	0.002373175
SSX1	0.191696193	0.002382188
IKZF3	0.191675843	0.002384787
SPRR3	0.191642933	0.002388995
ABHD13	0.191539246	0.002402299
CTLA4	0.191457347	0.002412855
SSX2	0.191398349	0.002420485
WDR67	0.191363916	0.002474886
POM121L1P	0.191357888	0.00242573
SDK2	0.191299743	0.002433287
CD3E	0.191228393	0.002442588
CTAG1A	0.191066197	0.002463853
CBLL1	0.191062801	0.0024643
GAGE12F	0.190979332	0.002475311
UBQLN1	0.190850049	0.002492453
C11orf49	0.190787728	0.002500755

TMED2	0.190740426	0.002507074
TMSB4X	0.190565652	0.002530544
KPNA2	0.190499924	0.002539422
FAM107B	0.190499637	0.002539461
ENOPH1	0.190350581	0.0025597
CCDC74A	0.190302349	0.00256628
ZFAT	0.190187795	0.002581971
RAP2A	0.190179028	0.002583175
INSM2	0.190085381	0.002596072
PIK3CG	0.190052439	0.002600623
RASSF5	0.189921754	0.002618747
LCP2	0.189849125	0.00262887
C12orf48	0.189782077	0.002691724
SIT1	0.189524631	0.00267453
GPR114	0.189487167	0.002679848
CDYL	0.189482572	0.0026805
LOC100506971	0.18934478	0.002754639
PAXIP1	0.189209677	0.002719535
RPGR	0.189156513	0.0027272
C2orf27A	0.189022089	0.002746667
BBS12	0.188735019	0.002788662
ZBTB41	0.188722822	0.002790459
THAP6	0.188631812	0.002803902
TLR10	0.188614312	0.002806494
HPRT1	0.188453722	0.002830378
ARHGEF18	0.188400946	0.002838267
STRN3	0.188296818	0.002853891
OR4H12P	0.188278683	0.002913656
C15orf42	0.1882764	0.002914006
CCR5	0.188208607	0.002867189
LSM4	0.188197437	0.002868876
HIST1H2BK	0.188164049	0.002873927
PVRIG	0.188163471	0.002874014
ARHGAP5	0.188057554	0.002890089
ADAM6	0.188053103	0.002890766
STRBP	0.188042719	0.002892347
YWHAB	0.187989609	0.002900444
ARC	0.1879844	0.002901239
SLC30A8	0.187743376	0.002938253
SLAMF1	0.187732897	0.002939872
CRB1	0.18756005	0.002966692
NAG18	0.187518461	0.003032087
E2F4	0.187486949	0.002978102
C19orf23	0.187477426	0.003038602
VNN2	0.187217862	0.003020444
GMFB	0.186896981	0.00307165
RTP4	0.186702824	0.003103013

FOXN4	0.186588876	0.003121556
DNAJA1	0.186575812	0.003123688
FLT3LG	0.186531163	0.003130985
CCR2	0.18650171	0.003135807
ITK	0.186417943	0.003149559
UEVLD	0.186397297	0.003152956
IKZF1	0.186371184	0.003157258
LOC440910	0.186365101	0.003220108
RPS6KA1	0.186313672	0.003166752
C16orf93	0.186312316	0.003166976
TOMM70A	0.186270885	0.003173832
ABCF2	0.186131342	0.003197022
ARL5C	0.186097501	0.003202669
COPB1	0.186083519	0.003205005
UFM1	0.186056028	0.003209602
HIST1H2AK	0.185843496	0.003245343
KIAA1468	0.185762064	0.003259132
BTN3A1	0.185717141	0.003266762
HPSE	0.18560983	0.003285054
ZNF746	0.185568938	0.003292048
CHMP7	0.185512354	0.003301749
PSMD7	0.185396983	0.003321609
RHEB	0.185238778	0.003349018
ANKRD22	0.185219259	0.003352413
SPATA18	0.185216437	0.003352905
ZNF574	0.18515762	0.003363157
ZNF800	0.185113741	0.003370825
C1orf56	0.185076151	0.003377406
HOMEZ	0.185017911	0.003387625
NKD1	0.184979448	0.003394389
TMEM71	0.184950737	0.003399446
ZNF236	0.184877824	0.00341232
SH3BGRL3	0.184864091	0.00341475
UBE2A	0.184766447	0.00343207
VTI1B	0.184761811	0.003432895
TMEM104	0.184733875	0.003437866
PAK4	0.184620497	0.003458108
CD6	0.184264297	0.003522406
ZNF174	0.184223537	0.003529832
ELK4	0.184182302	0.00353736
SLC22A17	0.184145882	0.00354402
MORF4L2	0.183970599	0.003576233
ZNF747	0.183913895	0.003586711
ORC5	0.183842147	0.003600008
ZAP70	0.18377134	0.003613174
GLYR1	0.183766619	0.003614054
GTPBP10	0.183714237	0.003623824

PDCL	0.183498255	0.003664363
MAP4K1	0.183487785	0.003666338
MAGEA1	0.18347264	0.003669197
IQSEC2	0.183438571	0.003675637
MPV17L2	0.18341937	0.003679271
LOC723972	0.183394541	0.003753954
MDH1B	0.183385331	0.00368572
CYB5A	0.183365279	0.003689525
ZNF396	0.183238871	0.003713589
RLTPR	0.183121023	0.00373615
DAZAP2	0.182826941	0.003792994
GAPT	0.182804849	0.003797295
FAM96A	0.182785191	0.003801127
HS3ST3B1	0.182763347	0.003805388
TFAP2D	0.182738182	0.003810303
PAIP2	0.182700906	0.003817593
PARN	0.182696087	0.003818537
C3orf70	0.182502741	0.003856562
HINT2	0.182391346	0.003878626
TMX1	0.182332017	0.003890423
RFWD3	0.182186764	0.003919443
PLEKHH1	0.182161124	0.003924586
SLC25A5	0.182088464	0.003939193
LOC399815	0.182082281	0.00401428
BCL2A1	0.182049335	0.003947079
LOC441666	0.182001796	0.004030764
NME5	0.181921476	0.003972949
PPL	0.181903358	0.003976628
WDR53	0.181733501	0.00401126
HSP90AA1	0.181685855	0.004021024
INTS2	0.18149596	0.00406015
DCUN1D1	0.181431867	0.004073433
ACN9	0.181352494	0.004089937
CD48	0.181275413	0.004106023
ZNF597	0.181188877	0.004124149
RGS14	0.181154064	0.004131462
TMEM165	0.181133753	0.004135734
C14orf178	0.181054371	0.004152467
SDHAF2	0.180987641	0.004166581
FCRL5	0.180911469	0.004182745
PBX4	0.180869394	0.004191697
NCF1	0.1807525	0.00421666
LAIR2	0.180622477	0.004244584
CCL5	0.180535035	0.004263457
C1QL4	0.180488142	0.004273609
DDX42	0.180443242	0.00428335
LCK	0.180355247	0.004302498

USP14	0.180352839	0.004303023
SS18	0.180331467	0.004307686
ATAD1	0.180321078	0.004309955
CD5	0.180286741	0.004317459
HSBP1	0.180279531	0.004319037
C17orf80	0.180271418	0.004320812
AGBL2	0.180169054	0.004343271
BRCC3	0.18008379	0.004362057
HSPH1	0.180066712	0.004365829
HMGN2	0.180037256	0.004372341
BAX	0.179973174	0.004386539
STAG3	0.17991882	0.004398615
LEF1	0.179875084	0.004408353
NXPH3	0.179866846	0.004410189
RHOH	0.179823526	0.004419857
PNMAL1	0.179794117	0.004426431
LRMP	0.17976414	0.004433142
IGHA1	0.179707462	0.004527129
CRLF3	0.179584652	0.004473513
PTPN6	0.179477431	0.004497787
CSAG3	0.179434091	0.0045898
GOLGA5	0.179427474	0.004509137
PSMC2	0.179379436	0.004520076
CD69	0.179367176	0.004522871
BIRC3	0.179349734	0.004526851
PRAME	0.17933623	0.004529934
C22orf34	0.179312924	0.00453526
FICD	0.179301995	0.00453776
TAG	0.17915602	0.004654348
PPP1R2	0.17906089	0.004593218
LEKR1	0.179019969	0.004602691
RAB2B	0.178997917	0.004607803
TCF7	0.178912487	0.004627655
SRP54	0.17889432	0.004631887
ME2	0.178781458	0.004658254
PDE6D	0.178702381	0.004676809
MLF2	0.178675588	0.00468311
NBPF22P	0.178605098	0.004699726
BANK1	0.178544708	0.004714004
ARRDC1	0.178393355	0.004749958
ICAM2	0.178379119	0.004753352
NUB1	0.178321496	0.004767114
LOC440905	0.178291592	0.004860258
MAPKAP1	0.17821755	0.00479203
TLX1NB	0.178192489	0.004798055
TSEN54	0.178186743	0.004799437
MOSPD2	0.178129924	0.004813125

DENND2D	0.178048596	0.004832779
C7orf43	0.177879248	0.004873934
SNORA7A	0.177818472	0.004888781
ZNF570	0.177814659	0.004889714
MAGEA12	0.177772393	0.004900065
DUOX1	0.177635647	0.004933688
LOC100290481	0.177622608	0.005025195
PIP4K2C	0.17759835	0.004942895
NR1D2	0.177567432	0.004950539
C2orf81	0.177552063	0.004954342
RAB39B	0.177525873	0.00496083
FMR1NB	0.177471169	0.004974405
RASGEF1C	0.177428395	0.004985044

Table S4. 1334 genes upregulated in younger pediatric NBL patients (Pearson Correlation Coefficients, p-values).

gene	correlation	pvalue
PXDN	-0.3325347	7.67371E-08
DIXDC1	-0.31184414	5.11775E-07
ABR	-0.309505126	6.2863E-07
ADAMTS10	-0.308573062	6.81982E-07
NAALAD2	-0.306947203	7.85585E-07
KIF13A	-0.306777224	7.97248E-07
ATP11A	-0.302947862	1.10844E-06
SHANK2	-0.295566395	2.06483E-06
LRRC7	-0.29186997	2.80147E-06
NCAM1	-0.291581845	2.86838E-06
BCAT1	-0.289097281	3.51207E-06
CPNE8	-0.285284046	4.77418E-06
BACE1	-0.284058826	5.26415E-06
AGRN	-0.284055795	5.26542E-06
CACNG7	-0.282783116	5.82498E-06
PDE10A	-0.281905685	6.24322E-06
HBG1	-0.280717012	6.85556E-06
CDR1	-0.280508752	6.96856E-06
TMEM25	-0.279862036	7.33085E-06
DRG2	-0.278171064	8.36464E-06
MAGI3	-0.277292212	8.95521E-06
DUSP16	-0.277119287	9.07598E-06
KCNK10	-0.276572415	9.46818E-06
PTCHD1	-0.276053563	9.85512E-06
CREB5	-0.274224693	1.13422E-05
AMOTL1	-0.273191038	1.22744E-05
EPB41L3	-0.270925266	1.45783E-05
ARHGAP32	-0.270299993	1.52829E-05
PIRT	-0.266310429	2.05974E-05
FOXRED1	-0.26454768	2.34653E-05
EYA4	-0.264427997	2.36731E-05
HIST1H1A	-0.262292588	2.76868E-05
TECTA	-0.260423525	3.17197E-05
GRAMD1B	-0.260034012	3.26272E-05
TRIO	-0.259845757	3.30746E-05
CDON	-0.258835002	3.55772E-05
EPHA6	-0.258364556	3.6802E-05
TTC28	-0.258123423	3.74451E-05
ME3	-0.255620284	4.47779E-05
FAR2	-0.254600279	4.81378E-05
NFRKB	-0.25443319	4.87103E-05
ARHGEF12	-0.252882722	5.43379E-05
FIGN	-0.252621956	5.53425E-05
RAI1	-0.250904519	6.24089E-05
PTPRR	-0.249810344	6.73446E-05
CD101	-0.249473385	6.89371E-05

NEURL4	-0.247875371	7.69833E-05
FXYD6	-0.247150484	8.09171E-05
HRK	-0.246388898	8.5253E-05
KSR1	-0.24636241	8.54076E-05
SYNE1	-0.246265252	8.59772E-05
SLITRK3	-0.246140249	8.67152E-05
THSD7A	-0.246090535	8.70104E-05
TRPM3	-0.245787913	8.88275E-05
ADCY6	-0.245541891	9.0331E-05
GCNT2	-0.243963093	0.000100563
SPOCK3	-0.243540671	0.000103479
AMACR	-0.243381812	0.000104597
C17orf107	-0.241567868	0.000118182
PRICKLE1	-0.240892709	0.000123648
CDRT4	-0.240604869	0.000126049
NES	-0.240569946	0.000126344
FGD4	-0.240539408	0.000126602
PRPF40B	-0.239976431	0.000131445
NICN1	-0.2399007	0.00013211
GAB2	-0.239561834	0.000135123
MICAL3	-0.238964008	0.000140596
ANK2	-0.2386782	0.000143285
GSTM2P1	-0.238213817	0.000147757
CACNA1E	-0.237666881	0.000153192
LRFN2	-0.237482271	0.000155068
ARHGEF25	-0.237229721	0.000157669
VPS26B	-0.237111979	0.000158896
HINFP	-0.236883101	0.000161306
RPL39L	-0.236625354	0.000164062
CNR1	-0.236167174	0.000169068
ALG9	-0.236077997	0.000170059
VGLL4	-0.23548748	0.000176759
DKK1	-0.235181385	0.000180329
ALDH1L2	-0.233757061	0.000197841
CEP164	-0.233703948	0.000198524
FMN1	-0.233346827	0.000203172
SV2C	-0.232648794	0.000212552
ADRBK2	-0.232180767	0.000219066
KLHL4	-0.232033247	0.000221158
MYO18A	-0.231935903	0.000222548
PITPNM2	-0.231846411	0.000223834
FRY	-0.23066495	0.000241465
SNRPN	-0.230588592	0.000242648
DIP2C	-0.230554855	0.000243173
NRSN1	-0.230032232	0.000251432
AGPAT6	-0.229762506	0.000255797
DEFB106A	-0.229378828	0.000262127

IFT172	-0.228239283	0.000281798
DIRAS1	-0.227441327	0.000296381
ZNF516	-0.226951042	0.000305687
LOC641467	-0.22693185	0.000314822
BMP5	-0.225959342	0.000325348
ACAP3	-0.225434384	0.000336225
LOC150622	-0.225254074	0.000349635
CADM1	-0.224889556	0.000347871
GRAMD4	-0.224479216	0.000356888
SCARA3	-0.223311152	0.00038376
NEU3	-0.222847617	0.000394936
XYLB	-0.222688452	0.000398843
GTDC1	-0.222554293	0.000402163
MRPS25	-0.222136786	0.000412663
KCNJ3	-0.221497658	0.000429229
FAM189A2	-0.221088598	0.000440153
GOLM1	-0.221011226	0.000442248
OBSCN	-0.220968004	0.000443423
TSPAN2	-0.220851509	0.000446602
SRRM4	-0.220596463	0.000453638
ANO4	-0.220543015	0.000455125
MCF2L	-0.220478349	0.000456931
SLC35E2B	-0.220474049	0.000457051
PCBP4	-0.220329918	0.0004611
GOLGA6L9	-0.219999903	0.000470498
CDRT1	-0.219971368	0.000471318
TNP2	-0.219959438	0.000471662
ARHGAP26	-0.219510888	0.000484749
PDXP	-0.219283268	0.000491518
SPTB	-0.219112139	0.000496665
DGKB	-0.21909153	0.000497288
FOXO3	-0.218792652	0.000506408
PHLDB1	-0.218493145	0.000515702
DYNC2H1	-0.217941905	0.000533222
TMEM44	-0.217433047	0.000549882
LMBR1L	-0.21739294	0.000551215
CSAD	-0.216913406	0.000567392
SPAG11B	-0.21682622	0.000570379
TRAK1	-0.216353316	0.000586841
CAMK1	-0.216304343	0.000588571
RAB30	-0.215901338	0.000602984
KIF1B	-0.215735612	0.000609006
SLC22A10	-0.215295144	0.000625281
ADCY1	-0.214808788	0.000643718
UCN3	-0.214403622	0.00065946
ST6GALNAC5	-0.21400756	0.000675191
STARD9	-0.21364679	0.000689821

HSD17B3	-0.21357396	0.00069281
CAMK1D	-0.213317547	0.000703428
C7orf41	-0.21283168	0.000742254
ARHGEF17	-0.212366426	0.000744141
CCDC149	-0.21212907	0.000754635
RNF144A	-0.212071849	0.000757185
CNTFR	-0.21188033	0.000765778
ARHGAP28	-0.211729928	0.00077259
DDAH1	-0.211670557	0.000775294
ASTN1	-0.211622171	0.000777504
SLC35E2	-0.211592857	0.000778846
POF1B	-0.211285517	0.000793044
KCTD21	-0.211174295	0.000798241
DPH1	-0.21048498	0.000831155
CHRM3	-0.210467032	0.000832028
NUDT3	-0.210290192	0.00084068
C11orf55	-0.210237337	0.000864088
AGPAT4	-0.210116435	0.000849262
HIST1H4F	-0.209954485	0.000857333
KIAA0649	-0.209942652	0.000879035
SNORD23	-0.209731213	0.000868576
RERE	-0.209473785	0.000881707
PRSS12	-0.20894505	0.000909254
LOC100128651	-0.208905859	0.000933537
ALDH3A2	-0.208784633	0.000917767
GSTM2	-0.208279123	0.000945076
TMPRSS5	-0.208152547	0.00095203
RGN	-0.208126308	0.000953477
DBC1	-0.207880898	0.00099046
RBFOX2	-0.207499446	0.000988666
LOC729723	-0.207269803	0.001025901
ANKZF1	-0.207197469	0.00100604
NANOG	-0.206646303	0.001038478
MLH1	-0.206425166	0.00105176
B4GALT2	-0.205533009	0.001106951
IGSF9B	-0.205526217	0.001107381
TBX1	-0.205504854	0.001108735
PARD3	-0.205472714	0.001110775
FEZ1	-0.20542614	0.001113737
IGSF3	-0.2051378	0.001132239
FRK	-0.204611517	0.001166739
PTPRO	-0.2044037	0.001180626
B4GALNT4	-0.204327435	0.001185761
FBN2	-0.204301038	0.001187543
ZNF445	-0.204283277	0.001188743
FBN3	-0.204109141	0.00120057
TMTC2	-0.204048783	0.001204695

PCDHGA11	-0.20382765	0.001219918
LRP5L	-0.203467993	0.001245055
SHC2	-0.203292368	0.001257501
CUL7	-0.203207595	0.00126355
BCO2	-0.203140802	0.001268334
BIN1	-0.203103757	0.001270995
SLC25A23	-0.202979017	0.001279992
PRKAR1B	-0.202771788	0.001295068
C11orf54	-0.202570538	0.001309865
DUSP26	-0.202544575	0.001311786
ACAD8	-0.202538398	0.001312243
PAR1	-0.202271272	0.001362661
PLCXD2	-0.202125223	0.00134316
RSF1	-0.201983528	0.001353916
C7orf59	-0.201652409	0.001410759
CCDC12	-0.201569818	0.001385773
CNTNAP5	-0.201340389	0.001403734
KIF21A	-0.201249886	0.001410878
VN1R5	-0.201007652	0.00146252
ANKRD13B	-0.200714054	0.00145386
LOC100131864	-0.20057861	0.001497921
KCNQ3	-0.200545915	0.001467593
TMEM136	-0.200429356	0.001477182
RNF207	-0.200336777	0.00148484
DNAJC12	-0.200274388	0.001490021
RPUSD4	-0.200161295	0.001499455
STT3A	-0.200021471	0.001511194
GOLGA6L10	-0.200000109	0.001512994
POLR2A	-0.199880906	0.00152308
ABCG4	-0.199689847	0.001539372
PAK7	-0.199261179	0.00157651
INPP1	-0.199208037	0.001581171
IGSF10	-0.199125241	0.001588457
LOC100506019	-0.199108964	0.001625215
RBMS3	-0.198564845	0.001638588
ACAT1	-0.198473655	0.001646881
CPSF3L	-0.197814588	0.001707969
ACVR2B	-0.197794244	0.001709887
RNF122	-0.197772691	0.001711921
MFSD10	-0.197572161	0.001730955
FAT4	-0.197408537	0.001746629
TRIB2	-0.197263699	0.001760612
MMP16	-0.196665052	0.001819496
GSTM1	-0.196563725	0.001829639
HRH1	-0.196510865	0.001834951
BAZ2B	-0.196143149	0.001872293
PAK1	-0.196072248	0.001879573

MGAT5	-0.195973747	0.001889729
ARAP3	-0.195803934	0.001907355
TMEM218	-0.19537428	0.001952625
TMEM45A	-0.195319601	0.001958456
STARD10	-0.195304336	0.001960087
XRRA1	-0.19523978	0.001966997
ND6	-0.194917625	0.002001813
ALDH7A1	-0.194875209	0.002006439
ULK2	-0.194860449	0.002008051
PCDHGB6	-0.194838253	0.002010477
GOLGA6L4	-0.19474528	0.002020669
OR51T1	-0.194673471	0.002028574
OVCA2	-0.194672847	0.002028642
HFM1	-0.194233697	0.002077598
LOC100507145	-0.194209667	0.002124372
NCKAP5	-0.194195209	0.00208194
SCN3B	-0.194110914	0.002091478
LAMA1	-0.19406325	0.002096888
C20orf3	-0.193987095	0.002150061
DOK6	-0.193891487	0.002116492
CATSPER3	-0.193819311	0.002124779
MINK1	-0.193749788	0.00213279
ASAP2	-0.193675156	0.00214142
ANKS1A	-0.193624718	0.00214727
IGF2BP3	-0.193442702	0.002168504
CELSR3	-0.193407369	0.002172648
APLP2	-0.193388558	0.002174857
SCN5A	-0.193371216	0.002176895
POLR3H	-0.193312395	0.002183822
CWC15	-0.193194538	0.002197762
AKR1C1	-0.193118427	0.002206807
SULT4A1	-0.192924994	0.002229948
LOC642311	-0.192693234	0.002305082
ILVBL	-0.192556992	0.002274584
ZZEF1	-0.192325154	0.002303121
RDH13	-0.192315709	0.00230429
CUX2	-0.192149713	0.002324932
KIF5C	-0.192056516	0.002336595
SETD5	-0.192022409	0.002340876
TFDP2	-0.191996113	0.002344182
USP35	-0.191974497	0.002346902
KIAA1211	-0.191575094	0.002397692
MAP4K4	-0.191449658	0.002413848
ETFB	-0.191251633	0.002439555
TRIM16	-0.191248117	0.002440014
FAT3	-0.191213265	0.002444564
DGKD	-0.191206831	0.002445405

AKR1C2	-0.190986858	0.002474316
HS6ST3	-0.190913614	0.002484011
CKMT1B	-0.190691371	0.002513641
AMMECR1L	-0.190661299	0.002517675
HTR4	-0.19036077	0.002558312
MYO15A	-0.190003324	0.002607421
LOC100144602	-0.189977872	0.002663979
SNAP91	-0.189907078	0.00262079
LOC147670	-0.18968439	0.002705664
PCDHB18	-0.189611379	0.002662253
TMEM175	-0.189534357	0.002673151
RAD52	-0.18945676	0.002684171
C11orf65	-0.189261355	0.002712104
DLG2	-0.189166819	0.002725713
EEF2K	-0.188665222	0.002798961
NPAS3	-0.188425856	0.002834541
CCDC81	-0.188398605	0.002838618
STH	-0.188255937	0.002860047
ENOSF1	-0.18816425	0.002873896
EPB41L5	-0.188132399	0.002878722
SFRP1	-0.188099432	0.002883724
KLHL13	-0.188026071	0.002894883
FN3K	-0.187910737	0.002912506
ARRB1	-0.187905066	0.002913375
SLIT1	-0.187654021	0.002952083
DOPEY1	-0.187644522	0.002953557
USP28	-0.18739593	0.002992363
SLC1A1	-0.186856097	0.00307823
PLEKHG3	-0.186553087	0.0031274
SLC4A2	-0.186296529	0.003169587
PDIA2	-0.186162667	0.003191803
MRPL14	-0.186059712	0.003208985
TMEFF2	-0.185951849	0.003227076
FAM168B	-0.185879213	0.003239311
TRIM16L	-0.185812656	0.003250559
SMAD9	-0.185488031	0.003305927
C6orf225	-0.185396789	0.003386049
FLRT2	-0.185253021	0.003346541
MAN2C1	-0.185129593	0.003368053
TRIM61	-0.185017338	0.003387726
MTL5	-0.184816243	0.003423227
PPAPDC1A	-0.184340249	0.003508606
PYGO1	-0.183994531	0.00357182
SLC45A2	-0.183922258	0.003585164
CAMTA2	-0.183891795	0.003590802
FAM13C	-0.183573698	0.003650156
FAT1	-0.183537766	0.003656916

SYTL4	-0.183417974	0.003679535
GSTA4	-0.183378994	0.003686922
BTBD9	-0.183267014	0.003708219
CXXC4	-0.183194391	0.00372209
ACVR2A	-0.183125314	0.003735327
C3orf35	-0.182945881	0.00376991
PAR5	-0.182823355	0.003865331
C1QTNF3	-0.182756061	0.00380681
RNF214	-0.182495515	0.00385799
ARHGEF10L	-0.182444021	0.003868179
PRTG	-0.182415409	0.00387385
PHACTR1	-0.182347395	0.003887362
LOC344595	-0.182103961	0.00400985
SSH2	-0.181950323	0.003967099
LOC285771	-0.181848137	0.004062406
C9orf4	-0.181514799	0.004131818
LRRFIP2	-0.181428388	0.004074155
LOC439914	-0.181354616	0.004165553
KLK9	-0.18120375	0.004121029
RAD21L1	-0.181200809	0.004121646
CYFIP1	-0.18111947	0.00413874
LEUTX	-0.181105902	0.004141597
HTR3A	-0.181097652	0.004143336
MIR125B1	-0.181072163	0.004148711
GDPD3	-0.181066832	0.004149836
LOC643650	-0.180845858	0.004274351
RAPH1	-0.180833524	0.004199343
CAP2	-0.180799905	0.00420652
CCDC127	-0.180770033	0.004212907
JMY	-0.180644958	0.004239744
SUN1	-0.180348523	0.004303965
FAM165B	-0.180318795	0.004389759
EPN2	-0.179902721	0.004402197
NINL	-0.179654064	0.004457861
LOC100128977	-0.17947714	0.004579879
FJX1	-0.179327849	0.004531849
LOC100508125	-0.179208341	0.004642141
SLC26A10	-0.179194476	0.004562416
C6orf27	-0.179185099	0.00464756
STOX2	-0.179040776	0.004597872
TP53AIP1	-0.178996711	0.004608082
C5orf4	-0.178768548	0.004745654
C1orf96	-0.178723756	0.004756313
PSD3	-0.178681646	0.004681685
WDR6	-0.17865773	0.004687315
LOC157562	-0.178648653	0.004774232
SARM1	-0.17855886	0.004710654

CST8	-0.178055582	0.004831087
DLGAP4	-0.177945008	0.004857916
IL1RAPL1	-0.177919697	0.004864076
HECW2	-0.177854029	0.00488009
NCRNA00254	-0.177810989	0.004978248
ANO8	-0.177724824	0.004911738
WDSUB1	-0.177607872	0.004940543
MIR16-2	-0.177605026	0.004941246
ACADL	-0.177516786	0.004963083
THSD4	-0.177338028	0.005007586
C9orf84	-0.177206712	0.005040506
ACVR1B	-0.177001665	0.005092299
SLC46A1	-0.176783095	0.005148035
LONRF2	-0.176669024	0.00517734
SNORD113-4	-0.176662692	0.005178971
MIR98	-0.17666179	0.005179203
ZNF512B	-0.176580996	0.005200056
CNNM4	-0.17657437	0.00520177
CHRNA7	-0.176565059	0.005204179
LOC100505888	-0.176258434	0.005377204
MYO1B	-0.17610254	0.005325102
C9orf7	-0.176075355	0.005426092
OR6K3	-0.176050078	0.005338976
NEUROD2	-0.176042951	0.005340863
GREB1	-0.176037007	0.005342437
NCRNA00294	-0.176033734	0.005437262
CHD3	-0.175895045	0.005380165
TBC1D3F	-0.175843815	0.005393838
KRT2	-0.175812472	0.005402219
PLCB1	-0.175661368	0.005442787
PPFIA2	-0.17551499	0.005482347
SLITRK1	-0.175451094	0.005499697
ARHGDIG	-0.175398039	0.005514139
ST6GALNAC3	-0.175288624	0.005544033
LOC100128003	-0.175211437	0.005662211
ZC3H7B	-0.175154019	0.005581007
HSFY1P1	-0.174958955	0.005634982
PLXNA4	-0.174929873	0.005643069
DCLK2	-0.174869027	0.005660022
GPR161	-0.174846336	0.005666356
C21orf74	-0.174806044	0.005776153
TUBGCP6	-0.17474614	0.005694402
TMEM163	-0.174716101	0.005702834
LOC100302640	-0.174433316	0.005882722
LOC284379	-0.174400011	0.005892329
FBXO31	-0.174369868	0.005800836
FAM123A	-0.174344996	0.005908231

GDF1	-0.174318008	0.005916045
SIDT2	-0.174249777	0.005835179
LRP1B	-0.174119118	0.005872751
MPDZ	-0.17400816	0.005904827
ST3GAL3	-0.173863046	0.005947015
RPH3A	-0.173800255	0.005965353
RGMB	-0.173592193	0.00602648
MGC42105	-0.173500776	0.006157106
FGF14	-0.173403869	0.006082292
RNF152	-0.173343359	0.006100322
LOC153811	-0.1732861	0.006221871
SHPK	-0.173041502	0.006190985
PCSK5	-0.172813183	0.006260361
DST	-0.172760172	0.006276567
OSBPL1A	-0.172669596	0.006304345
MYT1L	-0.172655081	0.006308807
PAQR7	-0.172555338	0.006339542
CNTROB	-0.172491956	0.006359143
CLVS2	-0.172489467	0.006359914
SLC23A2	-0.172426307	0.006379502
SCCPDH	-0.1724209	0.006381181
SYN3	-0.172364999	0.006398568
LOC100507063	-0.172357573	0.006509068
LOC100288594	-0.172329962	0.006517787
PTPRD	-0.17232204	0.006411957
SLC6A9	-0.172239291	0.006437819
TMEM39B	-0.172236753	0.006438614
LOC283140	-0.172222873	0.006551702
TIMP2	-0.172192443	0.006452502
RFTN1	-0.172015136	0.006508345
SHISA9	-0.171929659	0.00653542
TNFRSF19	-0.171886911	0.006548998
VHL	-0.171762171	0.006588764
SLC22A13	-0.171757501	0.006590257
IFT46	-0.171725139	0.006600611
NUMA1	-0.171687564	0.006612651
SH3PXD2A	-0.171655475	0.006622949
UPB1	-0.171629448	0.006631311
EPB49	-0.171576482	0.00675978
CHD5	-0.171557544	0.006654465
ZC3H12C	-0.17143681	0.006693503
NFASC	-0.171197037	0.00677164
NCRNA00086	-0.171147352	0.006901161
PRDM10	-0.171111101	0.006799842
HS6ST2	-0.1710138	0.006831901
SEMA4C	-0.170977584	0.006843868
IRS2	-0.170919939	0.006862954

ZBTB16	-0.170882607	0.00687534
FAM59A	-0.170880521	0.006990397
GARNL3	-0.170862954	0.006881868
ODZ2	-0.170839419	0.007004233
HIP1R	-0.170559796	0.006983277
RBMY2FP	-0.170557032	0.006984208
ATPAF2	-0.170502325	0.007002651
RMND5A	-0.170488366	0.007007364
KRTAP2-4	-0.17029084	0.007074359
REP15	-0.170166639	0.007116777
CDK5R1	-0.169736222	0.007265536
BSN	-0.169688578	0.007282171
EIF2B3	-0.169649925	0.007295693
FMNL2	-0.169528882	0.007338181
PISD	-0.169499401	0.007348562
BUD13	-0.169483546	0.00735415
PCDHGB7	-0.16944409	0.007368075
RPL24	-0.169195104	0.007456486
PCDH18	-0.168722315	0.007626972
TBC1D30	-0.168692613	0.007637798
SRGAP1	-0.168498819	0.007708766
EXOSC7	-0.16848799	0.007712749
PGAM2	-0.168436615	0.00773167
PREP	-0.168336873	0.007768522
EXTL3	-0.168248	0.00780149
AP4B1	-0.168143365	0.007840464
LOC100507194	-0.168016209	0.008015185
DCC	-0.167875645	0.007940975
NLGN2	-0.167837704	0.007955312
LOC648149	-0.167688678	0.008140498
C3orf14	-0.167653253	0.008025339
R3HDM1	-0.167620349	0.008037888
LPHN2	-0.167327469	0.008150364
SIK2	-0.167297381	0.008161998
KCTD16	-0.167257553	0.00817742
CRAMP1L	-0.1672362	0.008185699
ATXN7L2	-0.167227382	0.00818912
PAR-SN	-0.166950015	0.008429528
JAM3	-0.166945217	0.008299266
STOML1	-0.16674562	0.008377975
C8orf79	-0.166716207	0.008522896
SNORD55	-0.166662431	0.008410975
ZFYVE20	-0.166214561	0.008590632
ST8SIA1	-0.166127161	0.008626086
GABRG1	-0.166043311	0.008660223
GRID2	-0.165895184	0.00872082
PCDH11Y	-0.165869568	0.008731337

GRID1	-0.165860473	0.008735074
CLASP1	-0.16585553	0.008737106
KLKB1	-0.165745952	0.00878225
ZEB2	-0.16574403	0.008783044
PROKR2	-0.165732335	0.008787875
NF2	-0.165676568	0.008810942
LOC100506881	-0.165631594	0.008968148
KLHDC5	-0.165515608	0.009016963
TUBB2B	-0.165469956	0.008896875
DMC1	-0.165378906	0.00893498
SLC6A15	-0.165293899	0.008970686
GK5	-0.165255777	0.00898674
HSFY2	-0.16508102	0.00906066
MLLT4	-0.165012525	0.00908978
CCDC82	-0.164808026	0.009177214
KRT20	-0.164786432	0.009186489
MTNR1A	-0.164782745	0.009188074
PRKCZ	-0.164753384	0.009200702
CNNM1	-0.164636725	0.009251027
FBXW12	-0.164551011	0.009288158
TMCO3	-0.16452164	0.009300912
PVRL1	-0.164420398	0.009344994
SRY	-0.164397218	0.009355113
CRELD1	-0.163884257	0.009581538
RND3	-0.163879101	0.009583838
SAT2	-0.163790286	0.009623539
LPPR2	-0.163778874	0.009776632
LOC100288144	-0.163739864	0.009794328
LOC285889	-0.163615867	0.009850764
SLC25A12	-0.163613381	0.009703053
SNURF	-0.163569659	0.009871868
C4orf50	-0.163542858	0.009734913
CNNM3	-0.163482731	0.009762149
SRRD	-0.163454081	0.009775151
KCNQ5	-0.163383752	0.009807132
LPAR4	-0.163269525	0.009859273
FAM18B2	-0.163199462	0.010042398
LPCAT4	-0.163129653	0.009923454
ATP7B	-0.163027578	0.009970524
ABCB1	-0.162975332	0.009994694
CNTNAP1	-0.162967739	0.00999821
USP22	-0.162941141	0.010010538
LAMB1	-0.162871577	0.010042844
PLD2	-0.162764005	0.010092984
MIR181C	-0.16272499	0.010111223
OBSL1	-0.162647424	0.010147572
IKZF4	-0.162614879	0.010162857

MYH10	-0.16259062	0.010174264
LRRC63	-0.162334781	0.010295256
TMOD2	-0.162332494	0.010296344
TOX2	-0.162320954	0.010301832
BACH2	-0.162115303	0.010400065
RADIL	-0.161985427	0.010462529
LOC400662	-0.161860895	0.010680966
ENTPD3	-0.161832985	0.01053627
C1orf173	-0.161724131	0.010748188
RGS12	-0.161649131	0.010625816
PLAGL1	-0.161613392	0.0106433
DCUN1D5	-0.16156724	0.010665916
ZNF609	-0.161411571	0.010742514
PRELID2	-0.16133355	0.010781088
INPP5J	-0.161270562	0.010812318
IGDCC4	-0.161237178	0.010828903
ZNF586	-0.161223033	0.010835937
C1orf66	-0.160914396	0.0111539
MIR29B1	-0.160912361	0.010991443
NEIL1	-0.160825035	0.011035508
MAST2	-0.160706875	0.011095379
KIAA0408	-0.160644635	0.011127031
PTPRG	-0.160611954	0.011143683
RIMBP2	-0.160559752	0.011170327
WASH2P	-0.160430361	0.011236611
IMPDH2	-0.16024996	0.011329605
MC4R	-0.160192534	0.011359349
R3HDM2	-0.160067189	0.011424512
LOC100505679	-0.159822335	0.011722455
C16orf47	-0.159597828	0.011842456
KIAA0100	-0.159539271	0.011702595
UBE2Q2P2	-0.159450904	0.011749721
WDR27	-0.159307229	0.0118267
IGLON5	-0.15927869	0.011842044
IGF1R	-0.159191608	0.011888971
ZBED3	-0.158951569	0.012019174
ZNF154	-0.15894103	0.012024919
COLQ	-0.158830341	0.012085407
LOC284080	-0.158795893	0.012279984
MIR573	-0.158753837	0.01212737
GPR75	-0.158642447	0.012188699
ATP6AP1L	-0.158596925	0.01221384
LOC284688	-0.158595871	0.012391308
ACOT2	-0.158513587	0.012259985
MAP7	-0.158511784	0.012260986
B3GALT6	-0.158497065	0.012269152
LOC100509111	-0.158399652	0.012501377

ANKDD1A	-0.158322987	0.012366102
DKFZP686I15217	-0.158296476	0.012559598
KRTAP5-2	-0.158292874	0.012382941
ZNF428	-0.158114118	0.012483317
DEPDC5	-0.158109902	0.012485692
LOC100129995	-0.158016552	0.01271876
LRP5	-0.157939603	0.012582001
KLC4	-0.157883818	0.01261369
PLXNB1	-0.157864363	0.012624759
HCRTR2	-0.157863421	0.012625295
LOC100507185	-0.157799601	0.012843334
MIAT	-0.157473265	0.012849081
GALNT3	-0.157391321	0.012896523
OR5T3	-0.157296689	0.012951503
POLN	-0.1572029	0.013006196
RBM5	-0.157084968	0.013075257
UNC93B5	-0.157016004	0.013302258
LOC100272217	-0.156965415	0.013332376
ABCD2	-0.156920863	0.013171892
PCDH7	-0.15691868	0.013173182
OR52E2	-0.156879378	0.01319642
SRGAP3	-0.156876144	0.013198334
EED	-0.156839234	0.013220193
SLC30A10	-0.156801351	0.013242663
POU6F1	-0.156776046	0.01325769
CADM4	-0.1567081	0.013298114
CCDC158	-0.156706727	0.013298932
SCARNA9	-0.156695327	0.013305726
COL21A1	-0.156586548	0.013370701
CAMKK1	-0.156521022	0.013409976
C2orf80	-0.156490718	0.013428173
CSRNP3	-0.156473243	0.013438677
C1orf175	-0.156467236	0.013632172
SYT2	-0.156460593	0.013446284
PFKL	-0.156371053	0.013500244
SMAD1	-0.156173244	0.013620124
HIST1H4E	-0.156161593	0.013627214
TNRC6B	-0.156147445	0.013635827
C1orf51	-0.156061603	0.01388063
DBX2	-0.155993527	0.013729848
MIR32	-0.155910011	0.013781102
CYB5RL	-0.155888299	0.013794454
RAPGEF4	-0.15585502	0.013814942
IP6K1	-0.155709783	0.013904666
XKR6	-0.155709476	0.013904856
HMGCLL1	-0.155649821	0.013941858
SCAPER	-0.155634845	0.013951161

HHIP	-0.155572151	0.013990163
CRYBB2P1	-0.155554377	0.014001238
ABLIM2	-0.155544703	0.014007269
PAAF1	-0.155478327	0.014048711
FRMD3	-0.155403826	0.014095354
KCTD6	-0.155385474	0.014106865
WASH3P	-0.155361479	0.014121927
HMP19	-0.155228286	0.01440355
CTNNA2	-0.15517844	0.01423729
PIGL	-0.155077458	0.014301289
MEP1A	-0.1550505	0.014318416
HERC2	-0.154932222	0.014393776
MGC23284	-0.154886025	0.014623278
CITED2	-0.154691574	0.014548177
MBD5	-0.154615569	0.014597242
DLGAP1	-0.154597518	0.014608917
LOC728622	-0.154474139	0.014891593
LOC643988	-0.154450965	0.014906817
MIR134	-0.154442203	0.014709703
DDX23	-0.154437676	0.01471265
PTS	-0.154350691	0.014769372
HOXC9	-0.15419299	0.014872695
DEFB1	-0.154142829	0.014905692
ANKS1B	-0.15413412	0.014911428
OR5AN1	-0.153908184	0.015060901
NOMO3	-0.15385484	0.015096382
TOM1L2	-0.153850125	0.015099521
C6orf114	-0.153663771	0.015432102
FOXK1	-0.153625455	0.015249792
PAPOLB	-0.153537586	0.015308918
TRIM67	-0.153531841	0.015312791
FBRSL1	-0.153524118	0.015317998
HS3ST5	-0.153497994	0.015335625
C19orf63	-0.153479953	0.015557069
KCNE2	-0.153431031	0.015380887
KIAA1244	-0.153384921	0.015412122
TNRC18	-0.153336414	0.015445041
CHL1	-0.15322201	0.015522923
C21orf56	-0.153187032	0.015758037
METTL7B	-0.153154104	0.015569314
SCN3A	-0.153105765	0.01560241
MSTN	-0.153007464	0.015669905
AARS2	-0.15300366	0.015672522
TMEM135	-0.152948028	0.015710839
PVR	-0.152895969	0.015746768
MST1	-0.152855177	0.015774972
SAA3P	-0.152847287	0.015780432

SPATS2L	-0.152825431	0.015795566
RBMS1	-0.15280101	0.015812491
CLASP2	-0.152734641	0.015858569
ABCC6P1	-0.152638325	0.015925647
CATSPER2	-0.15262457	0.015935246
VARS2	-0.152560682	0.0159799
TLE3	-0.152526047	0.016004153
CEP57	-0.152494654	0.016026164
AKR1E2	-0.152423855	0.016075901
CWF19L2	-0.152381418	0.016105778
SLC22A4	-0.152224502	0.016216674
PCDHGA6	-0.152165511	0.016258537
LOC440297	-0.152161965	0.016479295
LOC154761	-0.152053545	0.016557241
TAGLN3	-0.152051673	0.016339588
LOC100126584	-0.152020499	0.016581063
PLEKHM3	-0.151969804	0.016398096
SYBU	-0.151969203	0.016398526
GRIP1	-0.151879815	0.016462617
UBE2Q2P1	-0.151845929	0.01648697
PDE2A	-0.151305801	0.01687942
LOC100130713	-0.151280969	0.017122044
SNORD116-27	-0.151271942	0.016904291
C7orf51	-0.151253434	0.01714248
ANKRD52	-0.151178156	0.016973347
KCNIP2	-0.151134429	0.017005628
MEX3B	-0.151043487	0.017072937
CACNB3	-0.151007075	0.017099951
WBSCR17	-0.150910227	0.017171985
INSR	-0.150890207	0.017186908
NCRNA00260	-0.150885974	0.017417247
NCAM2	-0.150605218	0.017400578
KCNH8	-0.15057768	0.017421347
XPNPEP1	-0.150555537	0.017438062
HS6ST1	-0.150409662	0.017548532
VASH1	-0.15039776	0.017557571
NDUFA10	-0.15039401	0.017560421
TRANK1	-0.150274167	0.017651685
UBIAD1	-0.150264427	0.017659121
RPUSD3	-0.150226598	0.017688026
DLG5	-0.150181479	0.017722555
AMIGO2	-0.150149298	0.017747217
PCDH9	-0.150103317	0.017782509
TRPV2	-0.150090176	0.017792606
NLGN1	-0.15004057	0.017830767
ST8SIA2	-0.150020563	0.017846178
ANKRD28	-0.150017797	0.017848309

2-Mar	-0.150001648	0.017860759
C1orf134	-0.149960576	0.018126278
ARHGEF7	-0.149872388	0.017960677
FBXO39	-0.149863888	0.017967265
BPHL	-0.149722222	0.018077367
OXCT1	-0.149710626	0.018086405
ARHGAP23	-0.149698806	0.018095622
TTTY6	-0.149669816	0.018118245
ADCY9	-0.149625439	0.018152923
RXFP2	-0.149616246	0.018160115
GABARAPL1	-0.149563736	0.018201237
PLEKHA9	-0.149472617	0.018510165
GABARAPL3	-0.149430765	0.018543418
DRP2	-0.149388577	0.018338997
LOC100130921	-0.149374084	0.018588535
AMPH	-0.149368124	0.018355142
HIVEP3	-0.149349273	0.018370034
BRSK1	-0.14929272	0.018414771
PTGFRN	-0.149239338	0.018457086
PRKCE	-0.149180798	0.018503588
CNTN5	-0.149102141	0.01856623
CROCCP3	-0.14903353	0.018621022
FAM19A1	-0.148972812	0.018669628
ZNF704	-0.148970181	0.018671736
CAMSAP1L1	-0.148897573	0.018971624
PTPN23	-0.148866348	0.018755121
OR4B1	-0.148841616	0.01877503
C5orf40	-0.14869816	0.019133967
NR2C2	-0.148680637	0.018905066
TBCEL	-0.14866673	0.018916336
SLC47A1	-0.148650102	0.018929819
LOC100506870	-0.148646887	0.019175902
CPNE9	-0.148554042	0.019007876
GTPBP2	-0.148529619	0.019027766
HAS2-AS	-0.148497853	0.01929825
LOC643454	-0.148482284	0.019311071
KLHL1	-0.148481754	0.0190668
DTX4	-0.148448809	0.019093707
SGSM2	-0.148426996	0.019111541
UQCRC1	-0.148392193	0.019140024
HYDIN2	-0.148355237	0.019415964
ARSE	-0.148238824	0.019265984
C1orf213	-0.148162089	0.01957638
FILIP1	-0.148157778	0.019332837
PCDHA6	-0.148042615	0.019428178
LOC729867	-0.147954453	0.01975011
TTC3P1	-0.147943119	0.019759632

RNF169	-0.147936273	0.019516578
ICK	-0.147710428	0.01970548
OSBPL5	-0.147648608	0.019757463
NLN	-0.147643006	0.019762179
LMO7	-0.147572691	0.019821463
HPS4	-0.147447247	0.019927609
PCGEM1	-0.147439148	0.01993448
LOC100505551	-0.147313518	0.020294852
ZNF630	-0.147279364	0.020070439
BNC2	-0.147208111	0.020131326
SNORD95	-0.147199657	0.02013856
TARBP2	-0.147194933	0.020142604
MIR206	-0.147055512	0.020262264
ING4	-0.147011853	0.020299862
C2orf84	-0.146898447	0.020654537
MIR3120	-0.146863424	0.020685138
DKFZp547J222	-0.14675767	0.020777778
MFSD9	-0.146650869	0.020613053
CACNB4	-0.14660132	0.020656366
CENPV	-0.146593431	0.02066327
ZFHX2	-0.146567119	0.02068631
C2orf83	-0.146397595	0.020835286
RHOBTB3	-0.14634844	0.020878656
RDX	-0.146275172	0.020943447
SLC44A5	-0.146259305	0.020957501
ZDHHC11	-0.146232822	0.020980976
LOC100507303	-0.146185483	0.021285267
MTUS1	-0.146169825	0.02103691
EPM2AIP1	-0.146109313	0.021090759
VN1R4	-0.146098706	0.021100211
DRD2	-0.146057356	0.021137091
OR51Q1	-0.146024423	0.021166504
FXYD2	-0.146021557	0.021169065
LOC100288974	-0.145959647	0.021488501
PDE4D	-0.145953273	0.021230169
OSTalpha	-0.145876654	0.021563609
OR5B3	-0.145811128	0.021357859
FBXL2	-0.145810853	0.021358106
LRRTM3	-0.145803153	0.021365043
MIR184	-0.145760096	0.021403863
ANKFN1	-0.145756594	0.021407023
UNC5C	-0.145752723	0.021410516
FLOT2	-0.145749107	0.02141378
TMEM196	-0.145692647	0.021464798
SLC43A3	-0.145640412	0.021512092
P4HA3	-0.14559335	0.021554779
LOC100506312	-0.145584442	0.021829868

LOC729970	-0.145498742	0.021908494
SNTG2	-0.145495312	0.021643939
CYP2G1P	-0.145449594	0.021685625
PFKP	-0.1453894	0.021740618
FOXB1	-0.145360624	0.02176695
FAM109B	-0.145237765	0.021879684
DZIP1	-0.145043333	0.022059126
LOC100131091	-0.145015688	0.02235627
LOC284798	-0.14497413	0.02239516
ZNF662	-0.14485465	0.022234477
ZDHHC8P1	-0.144802225	0.022283411
SACS	-0.144792238	0.022292743
MDN1	-0.144767668	0.022315717
ABCC5	-0.144765809	0.022317456
DPYD	-0.144740196	0.02234143
SPTBN1	-0.144725643	0.022355061
SMG6	-0.144702723	0.022376542
LOC100509093	-0.144679928	0.022672149
AFAP1	-0.144549386	0.022520722
C6orf35	-0.144496811	0.022846042
IL1F5	-0.144468304	0.022873216
PPIP5K1	-0.144357149	0.022702611
PCDHGA9	-0.1443246	0.022733533
MYRIP	-0.144310567	0.022746877
SORBS1	-0.144294153	0.022762492
CTBP2	-0.144191301	0.02286055
NCALD	-0.144138	0.02291151
P4HTM	-0.144041923	0.023003616
XKR3	-0.144008393	0.023035834
RNF217	-0.143990144	0.023053387
TRIOBP	-0.143925862	0.023115305
ITGA9	-0.143817503	0.023220004
ZNF815	-0.143737926	0.023579031
OR5D16	-0.143682611	0.023350913
LOC100510659	-0.143619869	0.023694863
LOC339468	-0.143581425	0.023732689
LHFPL4	-0.143579237	0.023451665
ABCA11P	-0.143552388	0.023477893
DKFZp566F0947	-0.143540123	0.023773384
KCNAB1	-0.143507662	0.023521643
SDHA	-0.143411272	0.023616168
LOC100508645	-0.143303553	0.024007641
ELP2P	-0.143265584	0.024045423
NISCH	-0.143214786	0.023809867
PCDH11X	-0.143176723	0.023847548
PGAP2	-0.143174211	0.023850036
CGREF1	-0.143172382	0.023851849

GSTM5	-0.143101255	0.023922411
CGNL1	-0.143069043	0.023954426
GLIPR1L1	-0.143005844	0.024017346
DARC	-0.142742911	0.024570758
SLC2A13	-0.142735259	0.024288352
SULT1C4	-0.142705004	0.024318816
ARCN1	-0.14265611	0.02436812
C9orf3	-0.142640929	0.024383445
TRIM48	-0.142565095	0.024460126
LOC285501	-0.14254277	0.024774519
OR5D13	-0.142500247	0.024525863
C11orf1	-0.142469431	0.024557153
GIPC2	-0.142461336	0.024565379
DAZAP1	-0.142403792	0.024623921
KRT25	-0.142335665	0.024693382
VAMP2	-0.142247346	0.024783684
PDLIM3	-0.142194268	0.02483809
ZFAND2B	-0.142179927	0.024852807
ASCL4	-0.142121832	0.024912504
OR5B2	-0.141998895	0.025039238
ZFYVE9	-0.141985247	0.025053341
WNT5B	-0.141977999	0.025060833
PARP11	-0.141970886	0.025068189
SPRN	-0.141963648	0.025075675
ABP1	-0.141949577	0.025387008
FLJ27255	-0.141932967	0.025404344
NF1	-0.14192803	0.025112543
TECTB	-0.141927865	0.025112713
LETMD1	-0.141901895	0.025139624
LRRC2	-0.141857928	0.02518524
ITFG2	-0.141829625	0.025214643
H19	-0.141770553	0.025276104
LOC121456	-0.141707224	0.025640967
MATN2	-0.141677586	0.025373092
FAM155A	-0.141584412	0.025470617
CCBE1	-0.141535007	0.02552246
CTAGE1	-0.14137506	0.025690922
PLEKHG5	-0.141354017	0.025713157
SAMD13	-0.141255818	0.025817134
MAN2A2	-0.141170088	0.025908203
LDLRAD3	-0.141167467	0.025910991
LOC100509213	-0.141143052	0.026240629
SULT1E1	-0.141116655	0.025965103
MEG8	-0.141063845	0.026021446
MIR377	-0.141046676	0.026039787
OR4A15	-0.140966065	0.026126045
TMEM192	-0.140953722	0.026139274

SEMA6D	-0.140949846	0.02614343
C11orf95	-0.140914872	0.026180951
C6orf163	-0.140907825	0.026188518
ASTN2	-0.140856213	0.026243987
LOC255130	-0.140850527	0.026556271
SGK2	-0.140841251	0.026260087
GRIA4	-0.140789551	0.026315782
TMEM63B	-0.140781973	0.026323954
LOC100131907	-0.140770995	0.02664265
SIPA1L2	-0.140755898	0.026352089
LRRC48	-0.140497986	0.02663178
PVT1	-0.140424967	0.026711427
ARPP21	-0.140413531	0.026723919
TMEM177	-0.14039773	0.026741189
FANCE	-0.14035692	0.026785834
NCF1C	-0.140338778	0.026805701
LOC100507201	-0.140294484	0.027165248
APC2	-0.14026968	0.026881488
LOC221710	-0.140243255	0.027221952
CA4	-0.140130778	0.027034391
MAGI1	-0.140105337	0.027062478
ATCAY	-0.140084624	0.027085363
CDH12	-0.140003296	0.02717538
PLAC1	-0.139992411	0.027187448
ZHX3	-0.139965536	0.027217262
FBXO42	-0.139955892	0.027227967
SULT2A1	-0.139891178	0.027299898
C21orf117	-0.139784061	0.027734769
FAM18A	-0.139753374	0.027769332
PPP3CB	-0.139749354	0.027458107
C9orf62	-0.139743776	0.027464346
RNASE8	-0.13971205	0.027499851
YWHAE	-0.139674178	0.027542287
CARTPT	-0.139655514	0.027563221
CSTT	-0.139578576	0.027966917
MRFAP1L1	-0.139538432	0.027694851
PIP5K1C	-0.139501627	0.027736341
PPCDC	-0.139493749	0.027745229
SOCS7	-0.139485477	0.027754564
D4S234E	-0.139475875	0.028083568
RNF112	-0.139470361	0.027771629
GLIPR1L2	-0.13946303	0.027779909
DIRAS2	-0.139415818	0.02783328
FSD1	-0.139386972	0.027865932
SMYD4	-0.13935299	0.02790444
PFKM	-0.139351098	0.027906586
NT5M	-0.139299307	0.027965365

TRYX3	-0.139295213	0.02828978
CLK2P	-0.139255596	0.028335172
C2orf24	-0.139166197	0.028437834
NRIP1	-0.139099657	0.028192954
PCGF3	-0.139063682	0.028234132
FLJ13773	-0.138929842	0.028710788
BCL7A	-0.138923211	0.028395411
C17orf85	-0.138904511	0.02841694
AKIRIN2	-0.138898923	0.028423377
SLITRK4	-0.138753703	0.028591077
PLCZ1	-0.138743271	0.028603158
PGM2L1	-0.13857286	0.0288011
PCDHB5	-0.138570813	0.028803485
ENPP3	-0.138529303	0.028851881
MEG3	-0.138322114	0.029094488
MED7	-0.1381885	0.029251865
SYNGR1	-0.138171142	0.029272364
FLJ44896	-0.138170886	0.029602477
CLPX	-0.138107957	0.029347084
KIF26A	-0.138106718	0.029348551
LOC100499467	-0.138061894	0.029732455
OR5A1	-0.138012069	0.02946079
COL7A1	-0.137932593	0.02955532
GRM5	-0.137930974	0.029557247
DUSP5P	-0.137900723	0.029925551
ARHGAP10	-0.137819551	0.029690219
MTHFD1L	-0.137748304	0.029775511
C22orf41	-0.137675672	0.030196974
LOC100499193	-0.137638148	0.030242433
CTTN	-0.137634975	0.029911615
PLAC8L1	-0.137603566	0.029949429
NDUFC2	-0.137598688	0.029955306
LOC100287428	-0.13747376	0.030442275
ALKBH8	-0.137349001	0.030257421
LOC100128063	-0.137254329	0.030710789
AKAP12	-0.13699277	0.030692955
KLHL17	-0.136985137	0.030702346
CUL9	-0.13694656	0.030749843
KIAA1908	-0.136927644	0.031114289
EDA	-0.136914262	0.030789657
TMEM225	-0.136840632	0.030880587
IFFO1	-0.136824128	0.030900999
FAM66D	-0.136770917	0.031309468
FAM5B	-0.136739098	0.03134922
LOC100130148	-0.136580698	0.031547757
C9orf25	-0.136486014	0.031666945
LOC100128508	-0.136469199	0.031688151

ZNF197	-0.136401923	0.031427131
LOC100134259	-0.136362295	0.031823258
MAP2K6	-0.136304803	0.031549233
KIAA1543	-0.136203714	0.032024581
PLB1	-0.136193871	0.031689193
GOLGA2P2Y	-0.136168213	0.03172164
DDX10	-0.136095922	0.031813212
OR4A13P	-0.136007575	0.032275083
AADAT	-0.135859504	0.032114262
TMEM117	-0.135851424	0.032124595
CXorf19	-0.135804062	0.032536765
OLFM2	-0.135785217	0.032209358
PDZK1P1	-0.13577267	0.03257729
ADPRHL1	-0.135739332	0.032268215
ALKBH5	-0.135641103	0.032394522
LOC100287482	-0.135583662	0.032822196
KALRN	-0.135515014	0.032557271
OR5T2	-0.135499273	0.032577637
L1TD1	-0.135417341	0.032683819
LOC401052	-0.135362127	0.033111243
NXPH2	-0.1353237	0.032805537
C9orf24	-0.135265079	0.03288193
WFDC6	-0.135245363	0.032907658
ASXL1	-0.135207205	0.032957498
CRIPAK	-0.135207059	0.03295769
ROBO2	-0.135062779	0.033146726
KCNT2	-0.135006907	0.033220177
PARK7	-0.135001782	0.033226922
SOHLH1	-0.134990937	0.033241198
PUS10	-0.134989012	0.033243732
KIAA0562	-0.134900488	0.033720524
MORF4L1	-0.134860114	0.033413817
MRE11A	-0.134755351	0.0335526
KRBA2	-0.134692738	0.033635778
GABRP	-0.134628248	0.033721634
PELI2	-0.134627529	0.033722592
LOC440894	-0.134580229	0.034148785
MPRIP	-0.134473701	0.03392814
BIRC8	-0.134339792	0.034107938
SLCO5A1	-0.134249468	0.034229673
PITRM1	-0.134207777	0.034285985
DHRS7B	-0.13420617	0.034288158
ADCYAP1R1	-0.133983211	0.034590668
ANKRD2	-0.133982268	0.034591952
TMEM151B	-0.133977791	0.034598051
TMEM92	-0.133876064	0.034736849
MIOS	-0.133870472	0.034744493

CA2	-0.133849858	0.034772682
QPCT	-0.133780855	0.034867184
PMCH	-0.133775148	0.034875008
ACSM3	-0.133757839	0.034898752
NADK	-0.133741287	0.03492147
ZNF806	-0.133723845	0.035316729
SLC22A2	-0.133707857	0.034967393
ProSAPiP1	-0.13369928	0.035350724
GDPD5	-0.133684281	0.034999809
IPW	-0.133669317	0.035392229
GHRLOS	-0.133660347	0.035032744
FGL1	-0.133591647	0.035127427
LOC390595	-0.133523561	0.035594714
JUP	-0.133439977	0.035337227
LRP1	-0.133425964	0.035356665
MCHR1	-0.13336682	0.035438802
PDLIM7	-0.133267536	0.035577049
NHEDC2	-0.13321379	0.036028315
SLC6A14	-0.133195717	0.035677338
KGFLP2	-0.133179649	0.036076377
VSTM2L	-0.13310018	0.035811118
HDAC9	-0.133060519	0.03586678
RAB6A	-0.132954442	0.036016013
FBXL19	-0.132945828	0.036028156
SNORD109A	-0.13291371	0.036073457
LOC100128300	-0.132887245	0.036490233
AMY1A	-0.132762658	0.036287159
AUTS2	-0.132761147	0.036289303
POLH	-0.132755374	0.036297492
LOC441268	-0.132744628	0.036693541
PPARGC1A	-0.132597463	0.036522096
TACC2	-0.132535996	0.036609842
CLDN8	-0.132490407	0.036675037
PHF13	-0.132474986	0.036697112
SYCN	-0.132432763	0.036757611
RBFOX1	-0.132416866	0.036780412
CD276	-0.132360161	0.036861837
ATF7	-0.132309637	0.036934517
BAHCC1	-0.132277323	0.036981063
NKAIN1	-0.132197201	0.03709669
NTNG1	-0.132160104	0.037150331
PMAIP1	-0.132094133	0.037245882
LOC647286	-0.132045999	0.037703386
FBLL1	-0.132035877	0.037718187
PTH1R	-0.132026963	0.037343384
LRP8	-0.131878896	0.037559075
CACHD1	-0.131860915	0.03758534

ACCN1	-0.131832757	0.038016263
ZRANB3	-0.131820859	0.037643906
ZMIZ1	-0.13181752	0.037648792
ST3GAL6	-0.131749852	0.037747915
C21orf84	-0.131693603	0.038221616
ZBED1	-0.131663881	0.037874167
GUCY1A2	-0.131595969	0.037974151
TTC27	-0.13155134	0.038039978
ZNF671	-0.131469022	0.038161649
DDX25	-0.131410246	0.038248725
ACSF3	-0.13139695	0.038268445
TDGF1	-0.131383063	0.038289051
CLSTN1	-0.131352247	0.038334814
ZFHX3	-0.13128507	0.038434729
MCAM	-0.131245579	0.038493569
LOC100131738	-0.131179126	0.038989
RPL14	-0.131075024	0.038748563
BAI3	-0.131051521	0.038783814
INPP5F	-0.131010425	0.038845517
GAS6	-0.131008669	0.038848155
5-Sep	-0.130996127	0.038867003
KCTD14	-0.13091888	0.038983262
C1orf57	-0.130910289	0.039395138
MXD4	-0.130881411	0.039039759
FAM124A	-0.130868208	0.039059684
RASIP1	-0.130839191	0.039103503
LOC152578	-0.130781197	0.039591426
GSTT1	-0.13074023	0.03925326
RPH3AL	-0.130728011	0.039271783
KIAA1984	-0.130669593	0.039761789
PUS3	-0.130596603	0.039471468
OR52D1	-0.130570181	0.039511723
LOC284242	-0.130525683	0.039982377
WHSC1L1	-0.130493103	0.039629347
SLC28A2	-0.130484568	0.039642391
SCMH1	-0.13042744	0.039729786
CNKSR3	-0.130386196	0.039792983
LOC100509475	-0.1303674	0.040226186
TAB1	-0.130289683	0.039941198
ZC3H13	-0.130266783	0.039976434
TMTC4	-0.130256591	0.039992124
NACA	-0.130184103	0.04010387
LOC100131744	-0.130180533	0.040515638
DHRS13	-0.130161428	0.040138878
TSPAN18	-0.130136872	0.04017682
COQ5	-0.1300687	0.040282314
TRIM65	-0.130001943	0.040385844

LOC100129999	-0.129921196	0.040920248
AMY2A	-0.129709774	0.040841597
TET3	-0.129635653	0.040957904
OR5P2	-0.129601851	0.041011037
AKR7A3	-0.129588084	0.041032694
MYO10	-0.129584369	0.04103854
NPRL2	-0.129548721	0.041094669
PANX1	-0.129534719	0.041116734
RAB11FIP3	-0.129510205	0.041155387
AS3MT	-0.12946122	0.041232718
NBEAL2	-0.129413135	0.041308747
C10orf85	-0.129366065	0.041797798
C12orf68	-0.129347465	0.041827473
SRCAP	-0.129347236	0.041413133
RSPH4A	-0.129325667	0.041447348
GREB1L	-0.129257629	0.04155543
MPND	-0.129230319	0.04159888
LOC100506365	-0.129197537	0.042067318
LOC647979	-0.129148143	0.042146589
RYR3	-0.129130675	0.04175774
GRK4	-0.129092151	0.041819294
SESTD1	-0.129017773	0.041938352
C10orf113	-0.128968506	0.04201737
FLJ23152	-0.12896004	0.04244962
ZSWIM7	-0.128932781	0.042074748
PTCHD2	-0.12891015	0.04211113
DPPA2	-0.128894295	0.042136634
TIMM8B	-0.128863914	0.042185541
PFAS	-0.128858394	0.042194431
LOC100505814	-0.12882289	0.042671719
METAP1D	-0.12876135	0.042350999
SLCO4C1	-0.128748207	0.042372241
VEPH1	-0.128739345	0.042386569
HEATR7A	-0.128678121	0.042907215
KCNMB2	-0.128575033	0.042652965
CYGB	-0.128538588	0.042712244
LOC100130927	-0.128509233	0.043183323
ARID1B	-0.12847566	0.04281476
LOC100130899	-0.12846168	0.043261335
C11orf20	-0.12845747	0.043268247
LEO1	-0.128454915	0.0428486
PPFIBP1	-0.128421506	0.042903148
HADHA	-0.128417519	0.042909661
MIR92A2	-0.12837019	0.042987044
LOC100130370	-0.128344096	0.043454741
CD24	-0.128283248	0.043129501
LOC100129717	-0.128206527	0.043681941

C3orf39	-0.128144502	0.043784702
ANO1	-0.128060935	0.043495571
ZSCAN23	-0.128045192	0.043521593
PRPF8	-0.12802517	0.043554706
RUNX1T1	-0.127980085	0.043629348
RUFY2	-0.127942978	0.043690862
FLJ41309	-0.12784684	0.044280692
MTG1	-0.12783536	0.043869674
RAB11FIP4	-0.12777184	0.043975506
C8orf17	-0.127762692	0.044421757
RP1	-0.127750092	0.044011789
PMM1	-0.127737339	0.044033077
PGAP1	-0.127726288	0.044051532
SGSM3	-0.127725418	0.044052985
STK31	-0.127710969	0.044077123
C19orf76	-0.127597283	0.044700147
SNX31	-0.127570049	0.044313131
SCAP	-0.127510573	0.044413057
SETDB1	-0.127505935	0.044420857
COL4A2	-0.127491588	0.044444994
ZNF589	-0.127488963	0.044449412
GSTA3	-0.127382429	0.044628996
KRTAP5-9	-0.127371129	0.044648079
LOC441204	-0.127338986	0.045137798
CCDC84	-0.127312684	0.044746894
OR1D5	-0.127301804	0.044765308
C20orf43	-0.127234862	0.045315234
FLJ38773	-0.127216085	0.045347294
VSNL1	-0.127203893	0.044931316
LOC339788	-0.127196208	0.045381252
SLC15A5	-0.127147207	0.045027664
KRTAP4-8	-0.127087164	0.045129904
PCDP1	-0.127085576	0.04557065
TRIM13	-0.127078933	0.045143935
SNORD114-26	-0.127076417	0.045148224
OR1J1	-0.126933907	0.045391751
C20orf4	-0.126923969	0.045848504
AMT	-0.126867728	0.045505215
MMP3	-0.126841165	0.045550824
SEMA3C	-0.126757884	0.045694066
PCDHB8	-0.126673032	0.045840398
F7	-0.126588015	0.045987408
SLC12A1	-0.126536869	0.046076038
C11orf10	-0.126488508	0.04660427
PPT2	-0.126478283	0.046177738
SLC36A4	-0.126466051	0.046198995
GLCCI1	-0.126443538	0.04623814

KIF3C	-0.126427002	0.046266911
SUPT6H	-0.12637583	0.046356036
LOC284661	-0.126349601	0.046847531
CCDC83	-0.126345101	0.046409626
GLB1L3	-0.126291007	0.046504091
SH3BP5	-0.126268805	0.046542908
PARP16	-0.126263469	0.046552241
TM7SF4	-0.12622126	0.047073231
ABCB9	-0.12620825	0.046648919
SH3YL1	-0.126207683	0.046649911
CXADRP2	-0.126167989	0.047167182
HSP90AB2P	-0.126120895	0.047250368
NCL	-0.126110838	0.046819877
HEPACAM2	-0.126020477	0.046978929
SLC6A4	-0.126019343	0.046980927
CALB1	-0.125962423	0.047081352
RILPL1	-0.125850939	0.047278565
LOC100287314	-0.125844926	0.047740301
NHLRC4	-0.125819754	0.047333854
THUMPD2	-0.125814138	0.047343818
OLFM3	-0.125735612	0.047483303
TFAP4	-0.125690324	0.047563905
ZNF391	-0.125678994	0.047584086
DPAGT1	-0.125653952	0.047628719
GPR22	-0.125636075	0.047660603
CEBPZ	-0.125543159	0.047826611
ARL10	-0.125361122	0.048153242
TSPAN6	-0.125331779	0.048206068
DSCR6	-0.125281845	0.048753123
IFT20	-0.125280355	0.048298759
LOC100287030	-0.125273935	0.048767478
FLJ16124	-0.125135698	0.04901891
PDE9A	-0.125135144	0.048561308
PAM	-0.125131323	0.048568233
SLC9A11	-0.125127723	0.049033449
SLC4A3	-0.125086415	0.048649678
ANO5	-0.125075422	0.048669633
SYNPO2	-0.125049983	0.048715837
ANK3	-0.125018671	0.048772757
PDE4B	-0.125012194	0.048784538
COL3A1	-0.125011616	0.048785589
GMEB1	-0.125009613	0.048789234
C1orf212	-0.124999859	0.049267034
GBF1	-0.124983728	0.048836343
KRT27	-0.124951155	0.048895679
OR2D3	-0.124910105	0.048970542
LOC158402	-0.124873775	0.049498276

OR2AJ1	-0.124850741	0.04954062
ABCA5	-0.124763736	0.049238256
SLC37A4	-0.124745722	0.049271289
IFNE	-0.124743171	0.049275968
PKD1	-0.124734192	0.049292441
CCNL2	-0.124681734	0.049388773
B3GALNT2	-0.124673207	0.049404446
C19orf10	-0.124666744	0.04941633
C7orf28B	-0.1246424	0.04992498
LOC401233	-0.124536913	0.050120534
GUSBP3	-0.124522475	0.049682194
EYA1	-0.124513008	0.049699683
PDLIM4	-0.124508701	0.04970764
NHSL2	-0.124497916	0.049727572
GIT1	-0.124493086	0.0497365
ABCC10	-0.124428099	0.049856759
C22orf9	-0.124422021	0.050334249
LOC100129198	-0.12441451	0.050348248
ZNF76	-0.124397634	0.049913219

Table S5. 95 genes upregulated in older pediatric NBL patients that are also correlated with low survival (p-values).

gene	logrank_pvalue
EIF3G	3.86209E-06
BOLA2	4.87449E-06
ZDHHC23	6.71427E-06
STK33	1.11276E-05
TRMT6	4.29669E-05
PSMD7	0.000102247
E2F4	0.000115562
LSM4	0.000158065
RPL28	0.00016337
CRB1	0.000404519
RRS1	0.000760361
LOC10029(	0.000856671
IGHG1	0.000919849
SDK2	0.000958091
VMA21	0.000984333
ZBTB25	0.001341196
CLDN15	0.001596759
BAX	0.001874591
TSEN54	0.002221953
IGFL2	0.00287664
NAA10	0.004216447
MX2	0.004258438
COPE	0.004406661
PSME1	0.004454313
SLC25A5	0.004462639
ODF2	0.004483149
NXT2	0.004806321
SIRT7	0.004926126
MLYCD	0.005495132
OR4N2	0.005960645
IGHM	0.005990895
ING1	0.006189109
UBXN10	0.006262149
CXXC1	0.007343863
G6PD	0.007479365
RBM20	0.007969073
ARC	0.009124695
TMX1	0.00963243
CCDC40	0.009781265
SUPT16H	0.010120016
RIBC2	0.010530146
PSMA6	0.012065038
TSPAN33	0.012347814
ST6GALNA	0.012816846
POLR2C	0.013691555
BMP7	0.015966397

MAPKAPK2	0.016134845
PIM2	0.017016797
PSME2	0.01715831
SURF4	0.01751735
POTEM	0.017801204
MAPKAP1	0.018523462
IGKC	0.018606301
SERP1	0.020531022
KIAA0125	0.02055746
PDP2	0.020740785
WWOX	0.021276604
RFWD3	0.023230231
RSPO4	0.023804826
OR52H1	0.023988889
LOC25602[illegible]	0.026694752
TAF10	0.027895593
NEK2	0.028969949
SLC6A8	0.029695037
LOC28371[illegible]	0.0300041
PAK4	0.030191893
PGRMC1	0.03084862
USP17L5	0.030974859
TAF9B	0.033673701
STK4	0.033714794
TOMM70A	0.034119072
OR52E8	0.035144733
IGHA1	0.035483443
C2orf81	0.035537152
CSAG3	0.037587764
TPST2	0.038334256
F8A1	0.039234204
LAGE3	0.039396062
RLTPR	0.04002201
POTEB	0.040148136
TRUB2	0.040651611
PLEKHH1	0.041591965
STRBP	0.042062318
NBPF22P	0.042444538
IGLL5	0.042470007
MRPS22	0.043223409
NAG18	0.043492122
PAGE5	0.043616428
MAGEC1	0.043680747
ZNF79	0.044239711
ADAT1	0.044549682
ERAP2	0.046402372
TCEAL4	0.046885523

PPP6R1	0.047605436
TFPT	0.049233124

Table S6. 397 genes upregulated in younger pediatric NBL patients that are also correlated with high survival (p-values)

gene	logrank_pvalue
PGM2L1	2.01603E-08
INPP1	7.25074E-08
DOPEY1	9.68449E-08
SCN3A	1.87989E-07
PLCXD2	1.11749E-06
C17orf107	1.28859E-06
ARHGAP28	1.30917E-06
FAR2	1.32814E-06
WDSUB1	3.57233E-06
KCNK10	3.9498E-06
TOM1L2	3.97876E-06
NCAM1	4.22075E-06
ICK	4.72815E-06
NCALD	5.87424E-06
CNR1	7.40349E-06
CCBE1	8.97316E-06
GDPD5	1.23257E-05
THSD7A	1.32008E-05
ABCG4	1.41754E-05
CSRNP3	1.51054E-05
TMEM196	2.92772E-05
SEMA6D	3.08856E-05
PRKCE	3.25319E-05
USP35	3.6644E-05
CLASP1	3.98531E-05
PIGL	4.26371E-05
FAM124A	4.66288E-05
ARHGAP23	5.71395E-05
CDRT4	6.0917E-05
PRICKLE1	6.46574E-05
IL1RAPL1	6.83678E-05
RAB6A	8.33428E-05
ULK2	8.68916E-05
LRRTM3	8.74174E-05
ALDH3A2	9.12096E-05
JUP	9.50086E-05
STARD10	9.56285E-05
GSTM2	0.000106554
SLC22A4	0.000111388
POF1B	0.000116995
KLHL4	0.000133271
RND3	0.000135787
TMEM44	0.000142622
LMBR1L	0.000145589
RPH3AL	0.000152729
AMIGO2	0.000157379

SCMH1	0.000157657
SOCS7	0.000204706
KIF1B	0.00021996
HOXC9	0.000230838
RUNX1T1	0.000246713
RGMB	0.000247237
NDUFA10	0.000250713
TUBB2B	0.000260545
ADCY1	0.000268919
KIAA0408	0.000295494
CHD5	0.000307951
TAGLN3	0.000316237
ANK3	0.000320301
SESTD1	0.000339794
AGPAT4	0.000341146
MAST2	0.000346383
ARHGEF10L	0.000351737
ATPAF2	0.000370029
AKR1C2	0.000373643
PTPRD	0.00038918
EPB41L3	0.000432235
AKIRIN2	0.000470174
PPP3CB	0.000479238
KLC4	0.000487472
BTBD9	0.000491811
NF1	0.000497927
SCN3B	0.000499242
THSD4	0.000503813
ARRB1	0.000509764
ANO4	0.000518191
AKR1C1	0.000557006
PTS	0.000561193
EPN2	0.000564528
CGNL1	0.000582952
ST3GAL3	0.000596931
CAMTA2	0.000630863
TMOD2	0.000636673
TRPV2	0.00064161
DST	0.000657368
RSPH4A	0.00065748
TOX2	0.000672977
CADM4	0.000691783
CPNE9	0.000729629
RAI1	0.000735642
CDH12	0.000746576
LONRF2	0.000789829
KLHL1	0.000795618

KIF13A	0.000798469
SLC12A1	0.000803578
KIF3C	0.000803866
STK31	0.000834026
CADM1	0.00084174
IGSF3	0.000876979
ZFAND2B	0.000897969
IKZF4	0.001003623
MYO18A	0.001004841
KIF5C	0.001009034
HIVEP3	0.001021862
AMPH	0.001021989
FBXO42	0.001049766
BAZ2B	0.001057297
DPYD	0.001074974
MBD5	0.001084449
ZZEF1	0.001157586
SLC35E2	0.001175229
ABCC10	0.001330573
AMACR	0.001408083
SSH2	0.001434171
HECW2	0.001471103
KCTD21	0.001477056
RBFOX2	0.001479026
FRMD3	0.001627757
SNAP91	0.001638206
EPHA6	0.001690849
DLG2	0.001851193
ARHGEF12	0.001856989
GLIPR1L1	0.001872663
GK5	0.001893327
HS3ST5	0.002044312
ANKFN1	0.002071853
SLC35E2B	0.002088377
HTR4	0.002088553
ABCA5	0.002123354
UBE2Q2P1	0.002253091
SMYD4	0.002258168
MATN2	0.002288231
LOC158402	0.002335424
ACVR2A	0.002501204
KRBA2	0.002620907
GSTA4	0.002642513
DTX4	0.00267249
PCSK5	0.002886468
ANKS1A	0.002928729
PSD3	0.00293776

CACNB3	0.002983896
LRP1B	0.002994551
RERE	0.003077077
MLLT4	0.003095683
DLG5	0.003158717
PAK1	0.003292577
CACNB4	0.00345007
ADRBK2	0.003467551
SLC22A10	0.003474563
ST8SIA2	0.003530697
GBF1	0.003724618
PRPF8	0.003768229
HSD17B3	0.003778121
PLEKHG5	0.003815244
CATSPER3	0.003900357
SRRM4	0.003964958
SHANK2	0.003970218
PYGO1	0.004315859
RUFY2	0.004373858
CARTPT	0.004462061
STOML1	0.004678585
DCC	0.004821626
AMY1A	0.00495775
TECTA	0.004999255
FBXL2	0.005072766
RSF1	0.005079918
GLIPR1L2	0.005152372
SCAPER	0.005488252
HIP1R	0.005551482
DGKD	0.005606965
GRID2	0.005666709
SIDT2	0.005784705
MYT1L	0.005825605
KALRN	0.005858193
TMEM63B	0.006172544
ZNF806	0.006217569
ZNF76	0.006298333
NUDT3	0.00630248
C17orf85	0.006343166
NDUFC2	0.006451698
PAK7	0.006637984
NPAS3	0.006762878
POLR2A	0.006870087
SPOCK3	0.006973979
KLKB1	0.007041572
DIXDC1	0.007049983
PHLDB1	0.007062256

RAPH1	0.007083943
SEMA3C	0.007091928
RNF112	0.007237033
TRIB2	0.007296769
VGLL4	0.007395607
SLCO4C1	0.007520939
GABRG1	0.007813096
SRGAP3	0.007998278
CLVS2	0.00818942
KCNMB2	0.008393146
XKR6	0.008445765
ARPP21	0.008464963
ANKZF1	0.008504633
ABLIM2	0.008648907
HIST1H1A	0.008680361
ALDH1L2	0.008688346
MAP4K4	0.00870589
SCCPDH	0.00878826
CCDC149	0.009066124
ENTPD3	0.009072107
SUN1	0.009085099
YWHAE	0.009115647
GPR161	0.009229178
SLC25A12	0.009358213
MORF4L1	0.009643051
PCDHGB6	0.009649309
ARHGAP26	0.009756512
ND6	0.009960518
DKK1	0.010048357
CAMSAP1L1	0.010382695
FOXO3	0.010480335
DRD2	0.01060738
INPP5F	0.010870327
PPFIBP1	0.010988889
USP22	0.011237521
PCDHB5	0.011240492
WHSC1L1	0.011253738
FAT3	0.01130926
REP15	0.011404472
CDK5R1	0.011493938
NEU3	0.011629056
SNRPN	0.011738758
DUSP16	0.011795334
DBX2	0.012051337
C1orf57	0.012267259
LRRFIP2	0.012281092
TMCO3	0.01239758

CDRT1	0.012477681
CLPX	0.012654945
KRTAP5-2	0.012735198
DUSP26	0.012769288
CNTFR	0.01280999
ALG9	0.01281434
PLEKHM3	0.012941766
CNTN5	0.013139509
LPCAT4	0.013143711
CLASP2	0.013518145
PRKCZ	0.013732717
NLN	0.013757242
ZBTB16	0.013800988
SIK2	0.013844623
ALKBH5	0.014308082
GALNT3	0.015303763
CSTT	0.01537558
RNF122	0.015417861
ANKDD1A	0.015468732
SAT2	0.015530361
KIAA1211	0.015544245
TMEM25	0.015651957
TMTC2	0.015722122
FLRT2	0.015945202
ABCB9	0.015967291
NHEDC2	0.015999614
CDON	0.016118819
SHPK	0.016131942
GMEB1	0.016189709
UBE2Q2P2	0.016265544
SH3BP5	0.016478477
MMP16	0.016802539
NRSN1	0.016922837
PTPRO	0.017152243
LOC152578	0.017328354
HFM1	0.017792643
CAMK1D	0.017881671
MYH10	0.017913629
PVRL1	0.018044635
SLC23A2	0.018130318
SPTBN1	0.01828676
FBXO31	0.018383805
MED7	0.018773016
FRY	0.018835362
TRIM67	0.018876774
RBMS1	0.019566723
IGF1R	0.019670897

RBFOX1	0.019742354
LOC285501	0.019817189
PIRT	0.020258632
R3HDM2	0.020586819
LRRC63	0.020773085
AUTS2	0.020847373
RAB30	0.021161656
C1orf66	0.02116902
RDX	0.021197255
UCN3	0.021354549
AGPAT6	0.021386722
DGKB	0.021775065
VSNL1	0.021843486
GCNT2	0.021958
KSR1	0.022078119
MRFAP1L1	0.022154458
SYN3	0.022180831
PISD	0.022199497
VSTM2L	0.022604286
ZMIZ1	0.022619743
TNFRSF19	0.02262542
INPP5J	0.02267259
FAM13C	0.02267787
CNTNAP5	0.023117935
GSTM2P1	0.023121844
LOC100288144	0.023584346
C9orf24	0.02373333
SGSM2	0.023824635
UPB1	0.023995762
TRIO	0.024487401
IFT20	0.02452325
PCDHB18	0.0245555
XPNPEP1	0.024823754
LEUTX	0.025380171
EXTL3	0.025999418
PCDHA6	0.026049939
OBSCN	0.026084807
OVCA2	0.026313773
DLGAP4	0.02718208
PPT2	0.027900239
ATF7	0.028282795
BACH2	0.028513503
RNF144A	0.028601724
HMGCLL1	0.028674358
PDE4D	0.028823088
NCAM2	0.028897146
PCDHGB7	0.029023322

AMMECR1L	0.029093389
CWC15	0.029256095
APLP2	0.029396739
CCDC82	0.029426238
SLC45A2	0.030012676
CHD3	0.030194948
CLK2P	0.030200233
DHRS13	0.030249606
RFTN1	0.030625029
RNF152	0.030673978
AMY2A	0.031378625
TIMP2	0.032308271
CREB5	0.032533622
PDE10A	0.032881184
ACAT1	0.032994917
C11orf10	0.033303222
CATSPER2	0.03368856
HRK	0.033865576
RBM5	0.033908
TAB1	0.033954181
PDE2A	0.034397147
SMG6	0.034442947
DIRAS2	0.034694543
TMEM135	0.03511389
SFRP1	0.035179575
GRAMD1B	0.035340138
PTCHD1	0.035645773
ZFHX2	0.035764297
GABARAPL1	0.035917288
ZC3H7B	0.036073278
TNRC18	0.03619316
CXorf19	0.036675019
B3GALT6	0.037132264
PLAGL1	0.037173981
CWF19L2	0.037420844
PTH1R	0.037425121
TSPAN2	0.037478308
QPCT	0.037481494
DCLK2	0.038113717
ST8SIA1	0.038121194
TIMM8B	0.039109292
ACAD8	0.039323608
C10orf113	0.039400674
FAM59A	0.039565862
PLXNA4	0.039680942
MINK1	0.04095235
ZFYVE20	0.041041504

SNTG2	0.041106463
NEURL4	0.041618008
GPR75	0.04235923
PPFIA2	0.043216503
PDE9A	0.043690022
LRRC48	0.04370157
ARID1B	0.043785896
GAB2	0.04393218
HRH1	0.044025721
LRP1	0.044233596
NT5M	0.04528062
SNORD116-27	0.04567768
ZSWIM7	0.045792221
CKMT1B	0.046236984
MPRIP	0.04714081
TRIM13	0.047474404
GRIA4	0.047549585
PCDHGA11	0.047948861
DHRS7B	0.048483612
PAM	0.048930267
PRKAR1B	0.048946908
RAB11FIP4	0.049753142

Table S7. Microarray values of every pediatric NBL patient for USP17L5 and SLC25A5

Barcode	USP17L5	SLC25A5
TARGET-30-PAAPFA-01	6.34369	7.49797
TARGET-30-PACLJN-01	6.85389	7.2494
TARGET-30-PACPJG-01	6.65609	7.350844
TARGET-30-PACRYY-01	6.648035	7.201079
TARGET-30-PACRZM-01	6.710275	7.446544
TARGET-30-PACSNL-01	6.83907	7.499866
TARGET-30-PACSSR-01	7.38606	6.112756
TARGET-30-PACUGP-01	6.931085	7.008871
TARGET-30-PACVNB-01	7.127225	7.259166
TARGET-30-PACYGY-01	7.144365	6.3568
TARGET-30-PACZPX-01	6.447405	6.333877
TARGET-30-PADENF-01	5.931115	7.305119
TARGET-30-PADFLI-01	7.1253	7.311108
TARGET-30-PADHWC-01	5.83292	6.4499
TARGET-30-PADIEY-01	7.418145	7.561596
TARGET-30-PADIHC-01	6.611155	6.43216
TARGET-30-PADINC-01	7.093615	7.139094
TARGET-30-PADIRB-01	6.44188	7.746661
TARGET-30-PADKFS-01	6.898475	7.607928
TARGET-30-PADKFU-01	6.44263	7.279621
TARGET-30-PADKGF-01	5.037455	7.822454
TARGET-30-PADKNE-01	6.8237	7.453961
TARGET-30-PADKRU-01	6.21478	6.837009
TARGET-30-PADKVV-01	6.756005	6.945703
TARGET-30-PADKXS-01	6.616055	7.77961
TARGET-30-PADKYP-01	5.443465	7.668076
TARGET-30-PADLDA-01	6.556135	7.672365
TARGET-30-PADLDT-01	7.41775	7.564254
TARGET-30-PADLES-01	6.206585	7.165311
TARGET-30-PADLIC-01	6.69105	6.821411
TARGET-30-PADLJN-01	6.854225	7.11099
TARGET-30-PADLKJ-01	8.037955	7.601786
TARGET-30-PADLNM-01	7.019515	8.291215
TARGET-30-PADLPR-01	6.69519	6.631788
TARGET-30-PADLPZ-01	6.503675	6.486964
TARGET-30-PADLTD-01	7.73079	7.238475
TARGET-30-PADMGA-01	6.496735	7.077119
TARGET-30-PADMTD-01	7.343985	7.863475
TARGET-30-PADMVB-01	6.07137	6.759499
TARGET-30-PADMXD-01	5.91236	8.491776
TARGET-30-PADMYD-01	6.014605	7.621761
TARGET-30-PADNFT-01	7.088425	7.618018
TARGET-30-PADNNX-01	6.9766	7.282244
TARGET-30-PADNXC-01	6.720745	7.595399
TARGET-30-PADPCI-01	7.13517	7.134739
TARGET-30-PADPEY-01	8.124025	7.351809

TARGET-30-PADPHP-01	7.00763	7.179214
TARGET-30-PADPMU-01	7.074215	7.837824
TARGET-30-PADPRJ-01	4.92305	7.339256
TARGET-30-PADRYN-01	8.911075	6.452775
TARGET-30-PADSEC-01	7.11676	7.322583
TARGET-30-PADSMF-01	5.441465	7.20456
TARGET-30-PADSXE-01	6.58295	7.275214
TARGET-30-PADSXU-01	6.11866	7.123258
TARGET-30-PADTCU-01	7.362	6.705514
TARGET-30-PADTJJ-01	7.18065	7.91286
TARGET-30-PADTRV-01	7.24927	6.151685
TARGET-30-PADUKP-01	5.92771	6.224019
TARGET-30-PADUWR-01	6.003945	7.253091
TARGET-30-PADUYJ-01	7.097785	8.030717
TARGET-30-PADVSN-01	7.199155	6.331025
TARGET-30-PADVWW-01	6.00389	7.067546
TARGET-30-PADWEN-01	8.393835	7.728039
TARGET-30-PADWIM-01	8.442215	7.017325
TARGET-30-PADWSM-01	6.96379	8.112376
TARGET-30-PADWVR-01	6.5428	6.962291
TARGET-30-PADXAS-01	6.7069	6.777609
TARGET-30-PADXBB-01	10.17287	6.743891
TARGET-30-PAIDJS-01	6.065515	6.59366
TARGET-30-PAIEIF-01	7.97943	7.799806
TARGET-30-PAILZJ-01	6.062535	7.603459
TARGET-30-PAIMDS-01	7.12947	7.12954
TARGET-30-PAIPGU-01	6.39834	7.958164
TARGET-30-PAIPUD-01	7.5537	7.035351
TARGET-30-PAIRDP-01	6.44881	7.674444
TARGET-30-PAIRIK-01	7.59401	6.503399
TARGET-30-PAIRLN-01	6.8645	7.716816
TARGET-30-PAISNS-01	6.46415	6.627454
TARGET-30-PAITCI-01	6.39482	7.073709
TARGET-30-PAITEG-01	6.63143	7.905282
TARGET-30-PAITRM-01	7.83952	7.363117
TARGET-30-PAITZV-01	6.904445	7.600076
TARGET-30-PAIUJB-01	7.057075	7.023984
TARGET-30-PAIVHE-01	7.130435	6.864655
TARGET-30-PAIVMJ-01	5.96901	6.437956
TARGET-30-PAIVZR-01	6.65207	6.91297
TARGET-30-PAIWHU-01	6.45748	6.102091
TARGET-30-PAIWVG-01	5.94227	7.002821
TARGET-30-PAIXLC-01	6.782965	6.88781
TARGET-30-PAIXNG-01	7.488195	6.699145
TARGET-30-PAJXLE-01	6.733935	7.789213
TARGET-30-PAJYHP-01	7.026615	7.06894
TARGET-30-PAJYLA-01	6.734775	7.503358

TARGET-30-PAJYVA-01	7.249445	7.446585
TARGET-30-PAJZUU-01	5.673765	7.630525
TARGET-30-PAJZWL-01	6.096885	7.065609
TARGET-30-PAKACY-01	8.1042	7.173702
TARGET-30-PAKADI-01	7.48974	6.780256
TARGET-30-PAKAFK-01	6.059355	7.217891
TARGET-30-PAKANZ-01	6.965885	6.831877
TARGET-30-PAKDHL-01	6.758955	6.844376
TARGET-30-PAKGCI-01	6.609835	6.75118
TARGET-30-PAKGKH-01	6.47334	7.746328
TARGET-30-PAKGMY-01	6.79447	7.20265
TARGET-30-PAKHAV-01	6.0483	7.45694
TARGET-30-PAKINP-01	6.06408	6.384622
TARGET-30-PAKJBA-01	6.47143	7.909049
TARGET-30-PAKSWB-01	5.55863	6.634039
TARGET-30-PAKUXT-01	7.20064	7.603824
TARGET-30-PAKWCJ-01	6.00664	6.405349
TARGET-30-PAKXDZ-01	8.159485	8.15675
TARGET-30-PAKYZS-01	6.40675	6.61969
TARGET-30-PALAWZ-01	5.95787	7.715949
TARGET-30-PALBFV-01	5.40582	7.526267
TARGET-30-PALBFW-01	7.11916	6.650119
TARGET-30-PALEAC-01	5.917625	6.501169
TARGET-30-PALEBK-01	6.544785	6.695791
TARGET-30-PALETP-01	7.339105	7.41425
TARGET-30-PALFRE-01	6.42159	7.918213
TARGET-30-PALFSE-01	6.523535	8.032241
TARGET-30-PALGFF-01	6.67675	6.869369
TARGET-30-PALHVD-01	6.478805	6.490695
TARGET-30-PALIIN-01	6.469665	6.541615
TARGET-30-PALKKV-01	6.259525	6.725175
TARGET-30-PALKUC-01	6.9378	6.83505
TARGET-30-PALLJJ-01	6.761925	7.055226
TARGET-30-PALNFE-01	6.109885	5.908267
TARGET-30-PALNHZ-01	6.89819	7.266113
TARGET-30-PALNVP-01	5.561495	6.620902
TARGET-30-PALPIN-01	5.77776	6.043119
TARGET-30-PALPSY-01	5.115245	6.491747
TARGET-30-PALSAE-01	6.59853	6.9178
TARGET-30-PALTYB-01	6.5808	7.532177
TARGET-30-PALUDH-01	6.994225	7.353242
TARGET-30-PALUYS-01	6.50663	6.951297
TARGET-30-PALVKK-01	6.238875	7.338779
TARGET-30-PALVUC-01	6.12072	6.052859
TARGET-30-PALVVH-01	5.308655	6.473895
TARGET-30-PALWBT-01	5.967935	6.417447
TARGET-30-PALWSJ-01	6.13868	7.372716

TARGET-30-PALWVJ-01	6.093795	7.684806
TARGET-30-PALWXP-01	5.51852	6.543454
TARGET-30-PALXTB-01	6.529015	6.630607
TARGET-30-PALXTE-01	5.79352	6.465086
TARGET-30-PALXUM-01	5.869585	6.727689
TARGET-30-PALYEC-01	7.2948	7.241587
TARGET-30-PALYLZ-01	6.08066	6.493287
TARGET-30-PALYPW-01	6.04211	6.563968
TARGET-30-PALZRG-01	5.60631	7.505824
TARGET-30-PALZZV-01	6.759545	7.200311
TARGET-30-PAMACS-01	6.711065	7.147029
TARGET-30-PAMAFV-01	5.5995	6.376179
TARGET-30-PAMAJT-01	6.967965	6.63859
TARGET-30-PAMBDH-01	6.63408	6.72204
TARGET-30-PAMBMJ-01	6.01711	7.208405
TARGET-30-PAMBYG-01	6.45718	6.675041
TARGET-30-PAMCXF-01	6.788955	7.613451
TARGET-30-PAMEHH-01	5.275485	6.813101
TARGET-30-PAMEZH-01	6.377495	6.819646
TARGET-30-PAMJED-01	7.048395	7.076856
TARGET-30-PAMLMY-01	7.01138	7.137171
TARGET-30-PAMLNB-01	6.29875	7.349899
TARGET-30-PAMMXF-01	7.26669	7.287143
TARGET-30-PAMMYR-01	5.92361	7.230881
TARGET-30-PAMNAL-01	5.036185	6.768901
TARGET-30-PAMNLH-01	6.86435	7.066509
TARGET-30-PAMVCL-01	6.06374	6.861504
TARGET-30-PAMXWF-01	5.538795	6.785678
TARGET-30-PAMYCE-01	7.81588	6.927754
TARGET-30-PAMYVA-01	6.77273	6.728146
TARGET-30-PAMZCE-01	8.534245	7.216435
TARGET-30-PAMZMG-01	6.70194	7.219286
TARGET-30-PAMZYM-01	7.46328	7.206721
TARGET-30-PANBJH-01	5.925175	6.76772
TARGET-30-PANBMJ-01	6.549	7.62716
TARGET-30-PANBSP-01	6.326215	7.766035
TARGET-30-PANCYF-01	8.29565	8.15089
TARGET-30-PANDHZ-01	8.41285	6.720301
TARGET-30-PANGTS-01	5.989295	6.846008
TARGET-30-PANHUA-01	6.34342	6.510969
TARGET-30-PANLSG-01	6.372515	6.35888
TARGET-30-PANNIJ-01	6.03288	7.35787
TARGET-30-PANRRW-01	9.404135	6.78093
TARGET-30-PANUKV-01	7.390175	7.392436
TARGET-30-PANUVK-01	6.18122	7.973599
TARGET-30-PANVAN-01	6.304285	6.728779
TARGET-30-PANYGR-01	6.694255	7.909897

TARGET-30-PANZHT-01	7.55097	7.420857
TARGET-30-PANZPV-01	8.610945	7.345141
TARGET-30-PAPAKS-01	6.8581	6.671717
TARGET-30-PAPBGH-01	6.30999	8.02318
TARGET-30-PAPBZI-01	7.27948	7.910346
TARGET-30-PAPEFE-01	5.889545	7.142824
TARGET-30-PAPEJW-01	6.766615	7.350337
TARGET-30-PAPHPE-01	8.627335	6.650069
TARGET-30-PAPJLD-01	6.322335	7.458382
TARGET-30-PAPKWN-01	6.08703	7.785001
TARGET-30-PAPRTD-01	7.67245	7.343833
TARGET-30-PAPSEI-01	7.18772	7.796371
TARGET-30-PAPSKM-01	7.83798	7.497421
TARGET-30-PAPTIP-01	7.64395	7.889773
TARGET-30-PAPTLD-01	7.711395	6.678393
TARGET-30-PAPTLY-01	6.39451	7.068729
TARGET-30-PAPTMM-01	5.46233	8.498962
TARGET-30-PAPUAR-01	6.553465	6.906262
TARGET-30-PAPUEB-01	6.357195	6.779582
TARGET-30-PAPUNH-01	6.651095	7.794819
TARGET-30-PAPUTN-01	6.65153	7.299754
TARGET-30-PAPWUC-01	5.87398	7.515424
TARGET-30-PAPZYP-01	6.289455	7.297241
TARGET-30-PARABN-01	7.250205	7.405711
TARGET-30-PARACM-01	6.468695	6.913749
TARGET-30-PARAHE-01	6.43339	7.771024
TARGET-30-PARCWT-01	6.09306	7.636199
TARGET-30-PARDUJ-01	8.22773	6.983005
TARGET-30-PAREGK-01	6.78726	7.03005
TARGET-30-PARFBW-01	8.68959	7.073037
TARGET-30-PARGDJ-01	7.20608	7.210153
TARGET-30-PARGUX-01	5.787375	6.956291
TARGET-30-PARHYL-01	5.90241	7.489705
TARGET-30-PARIKF-01	7.22072	7.673805
TARGET-30-PARIKT-01	6.768065	7.691194
TARGET-30-PARIRD-01	6.70351	7.225517
TARGET-30-PARJAR-01	9.293425	7.604917
TARGET-30-PARJTB-01	6.26812	7.965895
TARGET-30-PARMFA-01	7.284415	7.878589
TARGET-30-PARMTT-01	7.68143	7.445139
TARGET-30-PARNCW-01	7.000275	7.346534
TARGET-30-PARNEE-01	7.08499	7.199574
TARGET-30-PARRBU-01	6.28488	8.540715
TARGET-30-PARSEA-01	8.141835	7.108925
TARGET-30-PARSHT-01	6.583255	7.491313
TARGET-30-PARUXY-01	6.02337	6.955612
TARGET-30-PARVME-01	6.24556	7.150848

TARGET-30-PARVRR-01	7.684915	7.814415
TARGET-30-PARXLM-01	6.72809	7.416247
TARGET-30-PASATK-01	8.049195	6.597865
TARGET-30-PASAZJ-01	6.197775	6.997311
TARGET-30-PASBEN-01	7.800805	7.227166
TARGET-30-PASCKI-01	6.89507	7.453646
TARGET-30-PASCLP-01	6.2519	7.114839
TARGET-30-PASCUF-01	7.195135	7.066392
TARGET-30-PASDZJ-01	5.97962	7.190123
TARGET-30-PASFRV-01	6.22006	7.159814
TARGET-30-PASGPY-01	6.160115	6.699554
TARGET-30-PASJRT-01	7.108335	6.878801
TARGET-30-PASJYB-01	7.62113	7.167871
TARGET-30-PASKJX-01	8.261255	6.805509
TARGET-30-PASLGS-01	6.31999	7.329964

Table S8. Microarray values of every pediatric NBL patient for POF1B, RND3, KLC4, and SLC12A1.

Barcode	POF1B	RND3	KLC4	SLC12A1
TARGET-30-PAAPFA-01	3.532882	7.44919	7.231266	3.670032
TARGET-30-PACLJN-01	5.546098	7.877754	7.888136	4.586368
TARGET-30-PACPJG-01	5.184811	9.576647	7.57845	4.507737
TARGET-30-PACRYY-01	4.781669	9.926089	7.44349	4.516838
TARGET-30-PACRZM-01	4.919965	8.432804	8.037613	4.041041
TARGET-30-PACSNL-01	3.857683	8.234914	7.703652	3.601735
TARGET-30-PACSSR-01	3.748492	5.562615	7.061933	4.23875
TARGET-30-PACUGP-01	3.80228	9.315133	7.277768	4.082726
TARGET-30-PACVNB-01	3.546622	7.504144	7.403017	4.060347
TARGET-30-PACYGY-01	6.384315	8.187659	7.48207	3.93616
TARGET-30-PACZPX-01	3.442904	7.324674	7.405956	4.020055
TARGET-30-PADENF-01	7.529175	9.151557	7.585339	4.785634
TARGET-30-PADFLI-01	3.467857	9.557963	7.383132	4.330952
TARGET-30-PADHWC-01	5.245081	9.342913	6.967923	4.956584
TARGET-30-PADIEY-01	7.004307	9.056502	7.693526	4.411992
TARGET-30-PADIHC-01	6.142949	8.154263	7.709976	4.195442
TARGET-30-PADINC-01	3.610641	7.650421	7.331237	3.859949
TARGET-30-PADIRB-01	5.470049	9.57829	7.929417	4.35002
TARGET-30-PADKFS-01	6.150604	9.67092	7.61485	3.974013
TARGET-30-PADKFU-01	5.482167	7.685609	7.558222	4.046
TARGET-30-PADKGF-01	5.094602	8.910911	7.170365	3.849668
TARGET-30-PADKNE-01	3.37735	8.172694	7.717079	4.102267
TARGET-30-PADKRU-01	4.10881	6.373977	7.217171	4.112244
TARGET-30-PADKVV-01	5.671116	8.360259	7.039886	3.864293
TARGET-30-PADKXS-01	3.277421	8.354148	7.728141	4.024246
TARGET-30-PADKYP-01	5.146547	8.450772	7.325991	3.980847
TARGET-30-PADLDA-01	4.094157	7.465579	7.203041	4.107347
TARGET-30-PADLDT-01	3.829225	8.503048	7.732421	4.3396
TARGET-30-PADLES-01	4.402774	9.681926	7.765742	4.262486
TARGET-30-PADLIC-01	3.848985	8.493789	7.595875	4.27006
TARGET-30-PADLJN-01	4.628731	8.164357	7.522506	4.390002
TARGET-30-PADLKJ-01	3.990533	9.272109	7.314495	4.110451
TARGET-30-PADLNM-01	3.670476	7.565353	7.475415	4.058063
TARGET-30-PADLPR-01	3.30372	7.211474	7.523408	3.969196
TARGET-30-PADLPZ-01	3.975417	8.885461	7.470293	4.378104
TARGET-30-PADLTD-01	3.421601	7.67326	7.258608	3.892115
TARGET-30-PADMGA-01	4.730659	9.772505	7.18516	5.010912
TARGET-30-PADMTD-01	3.471046	8.094874	7.491585	4.270873
TARGET-30-PADMVB-01	3.817979	8.561742	7.539754	4.388738
TARGET-30-PADMXD-01	3.713904	8.433171	7.453373	3.886319
TARGET-30-PADMYD-01	4.147782	7.714146	7.445726	4.126973
TARGET-30-PADNFT-01	3.932413	7.81985	7.541869	4.638893
TARGET-30-PADNNX-01	4.404149	7.93518	7.524093	4.632726
TARGET-30-PADNXC-01	3.660251	8.630842	7.356274	3.928625
TARGET-30-PADPCI-01	3.849361	9.026648	7.305856	3.760449

TARGET-30-PADPEY-01	3.399243	7.291686	7.337104	4.099744
TARGET-30-PADPHP-01	3.929175	8.492896	7.462505	3.99084
TARGET-30-PADPMU-01	4.804194	9.326566	7.716524	4.519094
TARGET-30-PADPRJ-01	4.62997	9.302081	7.45058	5.318792
TARGET-30-PADRYN-01	3.36677	6.027759	7.196111	4.067534
TARGET-30-PADSEC-01	3.511054	8.147443	7.533485	4.639789
TARGET-30-PADSMF-01	5.864477	8.357147	7.636254	4.030834
TARGET-30-PADSXE-01	6.489508	9.499367	7.585556	3.985617
TARGET-30-PADSXU-01	6.2841	8.427564	7.398365	4.024434
TARGET-30-PADTCU-01	6.146331	8.761784	7.274851	4.689631
TARGET-30-PADTJJ-01	3.795023	8.313684	7.317366	3.898177
TARGET-30-PADTRV-01	6.606612	9.09467	7.551289	4.034743
TARGET-30-PADUKP-01	4.630232	6.172776	7.232959	4.127427
TARGET-30-PADUWR-01	4.001156	8.481029	7.789821	6.519313
TARGET-30-PADUYJ-01	3.557217	7.440603	7.365948	3.721637
TARGET-30-PADVSN-01	6.84667	8.422167	7.841489	4.593622
TARGET-30-PADVWW-01	3.589437	8.749705	7.247963	3.97804
TARGET-30-PADWEN-01	3.240964	8.667226	7.35084	3.819001
TARGET-30-PADWIM-01	4.887319	8.619137	7.428146	4.220327
TARGET-30-PADWSM-01	7.930311	10.0395	7.861953	4.397278
TARGET-30-PADWVR-01	4.438235	8.952856	7.684388	4.497431
TARGET-30-PADXAS-01	3.989982	7.729393	7.515972	4.015048
TARGET-30-PADXBB-01	4.12552	8.1181	6.876597	4.240487
TARGET-30-PAIDJS-01	5.810255	7.441339	7.350046	4.584193
TARGET-30-PAIEIF-01	3.830851	8.329593	7.376599	4.313585
TARGET-30-PAILZJ-01	3.997285	9.631274	7.412	3.831511
TARGET-30-PAIMDS-01	6.088134	9.269183	7.425659	4.532047
TARGET-30-PAIPGU-01	4.572189	7.804964	7.344349	3.753793
TARGET-30-PAIPUD-01	4.114964	9.512018	7.463633	4.286659
TARGET-30-PAIRDP-01	3.696244	7.12926	7.443077	4.165476
TARGET-30-PAIRIK-01	6.008242	9.239051	7.469425	4.329527
TARGET-30-PAIRLN-01	4.256131	8.769863	7.648009	3.803144
TARGET-30-PAISNS-01	6.03498	8.342478	7.836832	4.909571
TARGET-30-PAITCI-01	3.733538	9.158778	7.23977	3.934091
TARGET-30-PAITEG-01	4.148538	9.970501	7.190429	4.350534
TARGET-30-PAITRM-01	4.10344	7.199331	7.348405	4.176053
TARGET-30-PAITZV-01	3.853081	9.049038	7.426123	4.535018
TARGET-30-PAIUJB-01	3.531706	6.535953	7.52017	3.914221
TARGET-30-PAIVHE-01	4.295081	7.984226	7.390652	4.1768
TARGET-30-PAIVMJ-01	6.232197	9.631564	7.658551	4.030446
TARGET-30-PAIVZR-01	3.666197	8.228527	7.560111	4.084448
TARGET-30-PAIWHU-01	5.657421	9.285584	7.889963	5.027079
TARGET-30-PAIWVG-01	4.038968	7.847582	7.339866	4.162775
TARGET-30-PAIXLC-01	7.010078	8.937695	7.662487	4.1721
TARGET-30-PAIXNG-01	3.841515	6.660654	7.426295	4.121167
TARGET-30-PAJXLE-01	4.666078	9.743914	7.382309	3.789803
TARGET-30-PAJYHP-01	3.52183	8.27085	7.223057	3.981988

TARGET-30-PAJYLA-01	3.739859	8.936542	7.301952	4.022341
TARGET-30-PAJYVA-01	3.478586	7.328313	7.115313	3.612753
TARGET-30-PAJZUU-01	3.813835	6.789034	7.131175	4.379326
TARGET-30-PAJZWL-01	5.779253	8.232067	7.523174	4.442668
TARGET-30-PAKACY-01	3.616265	8.196345	7.194729	3.954737
TARGET-30-PAKADI-01	4.024921	9.086281	7.504738	4.450923
TARGET-30-PAKAFK-01	3.78659	8.700862	7.29155	4.067336
TARGET-30-PAKANZ-01	3.656895	5.782856	7.11687	3.979126
TARGET-30-PAKDHL-01	3.573905	8.354029	7.681215	3.895618
TARGET-30-PAKGCI-01	6.657982	8.31457	7.719827	4.804315
TARGET-30-PAKGKH-01	3.997265	9.686394	7.744685	4.630577
TARGET-30-PAKGMY-01	3.761558	7.794246	7.680998	4.255485
TARGET-30-PAKHAV-01	6.999222	9.827686	7.548256	4.871248
TARGET-30-PAKINP-01	3.945927	8.896662	7.777284	5.378725
TARGET-30-PAKJBA-01	4.142943	7.607881	7.473015	4.421635
TARGET-30-PAKSWB-01	6.744521	8.900473	7.451037	5.689163
TARGET-30-PAKUXT-01	3.59241	9.049975	7.597595	4.863055
TARGET-30-PAKWCJ-01	6.022256	8.065644	7.679924	4.693018
TARGET-30-PAKXDZ-01	3.628744	8.277843	7.426004	3.832164
TARGET-30-PAKYZS-01	4.144725	8.613255	7.574469	4.114364
TARGET-30-PALAWZ-01	4.17376	9.237461	7.179737	4.224799
TARGET-30-PALBFV-01	3.898508	6.488899	7.27098	3.973905
TARGET-30-PALBFW-01	6.37789	9.308821	7.59742	4.822062
TARGET-30-PALEAC-01	6.603545	10.39106	7.436422	4.822083
TARGET-30-PALEBK-01	7.076045	9.525848	7.710722	4.94297
TARGET-30-PALETP-01	3.897299	6.714275	7.520306	4.015185
TARGET-30-PALFRE-01	4.07648	6.566385	7.327245	4.036222
TARGET-30-PALFSE-01	4.930562	8.882529	7.584685	4.431617
TARGET-30-PALGFF-01	7.356081	8.936094	7.646701	4.771268
TARGET-30-PALHVD-01	3.802659	9.020764	7.693998	4.134276
TARGET-30-PALIIN-01	3.860674	9.572555	7.43701	4.980002
TARGET-30-PALKKV-01	4.793943	10.07148	7.732247	4.151275
TARGET-30-PALKUC-01	3.627615	8.217132	7.402778	3.966456
TARGET-30-PALLJJ-01	5.842638	10.51465	7.582187	4.95829
TARGET-30-PALNFE-01	3.849523	8.922391	7.601734	5.038914
TARGET-30-PALNHZ-01	3.670213	7.746408	7.545194	3.874885
TARGET-30-PALNVP-01	5.989553	8.829647	7.717439	4.588352
TARGET-30-PALPIN-01	5.161684	9.414156	7.864059	4.170216
TARGET-30-PALPSY-01	4.892888	8.655833	7.687659	4.332048
TARGET-30-PALSAE-01	5.182357	7.741861	7.664453	3.984781
TARGET-30-PALTYB-01	6.571439	9.915416	7.60886	5.987634
TARGET-30-PALUDH-01	6.835142	9.209423	7.817159	4.256885
TARGET-30-PALUYS-01	4.471858	8.846413	7.452557	4.347847
TARGET-30-PALVKK-01	4.735611	9.08122	7.216259	3.97588
TARGET-30-PALVUC-01	4.948305	9.069564	7.782776	4.542669
TARGET-30-PALVVH-01	6.351338	9.902263	7.562985	4.761915
TARGET-30-PALWBT-01	4.492475	9.472565	7.524821	4.823414

TARGET-30-PALWSJ-01	7.864462	8.58304	8.071064	4.453924
TARGET-30-PALWVJ-01	3.987779	9.012387	7.509056	4.003936
TARGET-30-PALWXP-01	6.972253	9.684789	7.796135	5.327816
TARGET-30-PALXTB-01	5.754335	9.531121	7.663216	4.484061
TARGET-30-PALXTE-01	6.953757	8.385019	7.672126	4.087678
TARGET-30-PALXUM-01	7.43485	8.428916	7.734476	4.326952
TARGET-30-PALYEC-01	3.658641	7.55065	7.550175	3.873409
TARGET-30-PALYLZ-01	4.389693	9.472915	7.839149	4.220529
TARGET-30-PALYPW-01	4.402747	9.045549	7.25789	4.251455
TARGET-30-PALZRG-01	3.818305	7.39815	7.729027	4.193877
TARGET-30-PALZZV-01	3.646624	6.142102	7.4062	4.144153
TARGET-30-PAMACS-01	3.823262	8.874828	7.508678	3.976167
TARGET-30-PAMAFV-01	5.928767	7.370935	7.904437	4.346819
TARGET-30-PAMAJT-01	4.890379	6.432407	7.527991	4.711262
TARGET-30-PAMBDH-01	3.783092	6.61914	7.469595	4.321574
TARGET-30-PAMBMJ-01	4.41768	7.843358	7.578928	4.064434
TARGET-30-PAMBYG-01	3.721193	8.810247	7.305932	3.913904
TARGET-30-PAMCXF-01	3.727528	8.894117	7.902532	4.230123
TARGET-30-PAMEHH-01	5.02186	9.488003	7.543782	4.492801
TARGET-30-PAMEZH-01	4.797332	9.134549	7.416974	4.65604
TARGET-30-PAMJED-01	3.748895	8.301934	7.319105	4.051006
TARGET-30-PAMLMY-01	3.729096	8.945932	7.968192	4.328607
TARGET-30-PAMLNB-01	3.787037	9.331987	7.389902	3.892825
TARGET-30-PAMMXF-01	3.676898	7.205436	7.461257	3.85843
TARGET-30-PAMMYR-01	4.227205	7.568491	7.387596	4.012325
TARGET-30-PAMNAL-01	4.313085	8.163194	7.320541	4.119297
TARGET-30-PAMNLH-01	5.442	8.40906	7.277163	4.407139
TARGET-30-PAMVCL-01	4.066717	8.262472	7.561523	4.643181
TARGET-30-PAMXWF-01	6.045256	8.987235	7.542462	5.341587
TARGET-30-PAMYCE-01	3.747629	7.655973	7.701341	4.134716
TARGET-30-PAMYVA-01	5.715089	9.875318	7.276749	4.367144
TARGET-30-PAMZCE-01	3.793577	7.533747	7.603452	4.288291
TARGET-30-PAMZMG-01	3.69556	6.655396	7.614421	3.962282
TARGET-30-PAMZYM-01	4.018599	7.47594	7.393448	4.409878
TARGET-30-PANBJH-01	4.18871	7.899541	7.569997	4.943557
TARGET-30-PANBMJ-01	3.983854	8.965163	7.440474	4.171033
TARGET-30-PANBSP-01	3.752633	8.526833	7.520635	3.748756
TARGET-30-PANCYF-01	3.748874	8.354526	7.510926	3.94892
TARGET-30-PANDHZ-01	4.586865	8.208649	7.320862	4.33043
TARGET-30-PANGTS-01	4.099896	9.173476	7.571068	4.44436
TARGET-30-PANHUA-01	4.478824	8.86997	7.359072	3.992953
TARGET-30-PANLSG-01	4.065829	6.660846	7.674311	4.125936
TARGET-30-PANNIJ-01	3.86935	7.450055	7.328302	4.144369
TARGET-30-PANRRW-01	3.920562	8.747328	7.653229	4.548729
TARGET-30-PANUKV-01	3.596396	6.721014	7.565114	3.845622
TARGET-30-PANUVK-01	4.310099	9.696565	7.40697	3.921352
TARGET-30-PANVAN-01	4.145975	8.305791	7.212438	4.1618

TARGET-30-PANYGR-01	6.507118	8.397689	7.540845	4.112624
TARGET-30-PANZHT-01	3.755653	8.85078	7.435144	4.03415
TARGET-30-PANZPV-01	3.684692	8.197433	7.620252	4.181165
TARGET-30-PAPAKS-01	4.416717	5.748505	7.919207	4.298747
TARGET-30-PAPBGH-01	3.493613	7.601711	7.285074	3.793008
TARGET-30-PAPBZI-01	3.682714	9.737988	7.368391	4.04078
TARGET-30-PAPEFE-01	3.973	7.61794	7.217666	4.091815
TARGET-30-PAPEJW-01	3.786813	8.250513	7.411256	4.262631
TARGET-30-PAPHPE-01	3.982815	7.208986	7.418337	4.597797
TARGET-30-PAPJLD-01	5.048854	9.326232	7.628565	4.540991
TARGET-30-PAPKWN-01	4.005149	7.833474	7.514759	4.175441
TARGET-30-PAPRTD-01	4.695971	9.682117	7.364547	5.224014
TARGET-30-PAPSEI-01	3.68794	7.395081	7.40512	3.818797
TARGET-30-PAPSKM-01	5.20929	8.865752	7.483765	4.046374
TARGET-30-PAPTIP-01	3.560796	8.057161	7.563575	3.897091
TARGET-30-PAPTLD-01	3.882926	6.235535	7.562925	4.213489
TARGET-30-PAPTLY-01	3.749877	6.367844	7.749175	3.878093
TARGET-30-PAPTMM-01	3.85783	8.024097	7.113701	3.712504
TARGET-30-PAPUAR-01	5.314902	9.645609	7.352604	4.739589
TARGET-30-PAPUEB-01	4.063221	8.362072	7.118334	4.615756
TARGET-30-PAPUNH-01	3.641229	8.11042	7.070609	3.812024
TARGET-30-PAPUTN-01	4.031042	6.587272	7.402867	4.274166
TARGET-30-PAPWUC-01	4.644857	7.145628	7.048489	3.563816
TARGET-30-PAPZYP-01	3.677215	8.997258	7.284356	3.66723
TARGET-30-PARABN-01	3.714768	6.864521	7.439561	3.826868
TARGET-30-PARACM-01	4.93561	9.530025	7.236509	5.223186
TARGET-30-PARAHE-01	4.796799	8.663311	7.318792	3.553707
TARGET-30-PARCWT-01	8.203767	9.077078	7.206067	3.913981
TARGET-30-PARDUJ-01	4.512672	9.047349	7.527542	4.305707
TARGET-30-PAREGK-01	4.936391	8.816002	7.332763	3.908467
TARGET-30-PARFBW-01	4.231224	8.828135	7.462015	5.517975
TARGET-30-PARGDJ-01	6.004587	9.269424	7.641684	4.298155
TARGET-30-PARGUX-01	4.143058	8.649568	7.607437	3.912526
TARGET-30-PARHYL-01	5.998327	8.684588	7.314653	3.968592
TARGET-30-PARIKF-01	3.680628	8.507618	7.50721	3.721032
TARGET-30-PARIKT-01	5.943392	7.775303	7.328575	4.875795
TARGET-30-PARIRD-01	3.976131	7.731482	7.326808	4.194858
TARGET-30-PARJAR-01	4.935064	9.705004	7.267613	4.524678
TARGET-30-PARJTB-01	3.646968	7.688374	7.254756	3.722042
TARGET-30-PARMFA-01	5.769027	9.513786	7.082566	4.758447
TARGET-30-PARMTT-01	4.792083	8.355502	7.301073	3.875561
TARGET-30-PARNCW-01	5.915791	7.268342	7.177561	3.832935
TARGET-30-PARNEE-01	3.802288	7.571516	7.168702	3.891752
TARGET-30-PARRBU-01	3.801819	8.085515	7.260736	3.681208
TARGET-30-PARSEA-01	5.988968	7.936561	7.556949	4.347356
TARGET-30-PARSHT-01	3.64247	6.238298	7.460582	3.8766
TARGET-30-PARUXY-01	3.946519	6.71562	7.61002	3.964215

TARGET-30-PARVME-01	3.965892	9.296615	7.391808	4.002516
TARGET-30-PARVRR-01	3.51115	7.974591	7.503109	3.573651
TARGET-30-PARXLM-01	6.47417	9.508162	7.777235	4.225032
TARGET-30-PASATK-01	5.866666	9.148342	7.386589	4.082573
TARGET-30-PASAZJ-01	3.97051	7.965986	7.338128	4.489648
TARGET-30-PASBEN-01	4.178736	7.880033	7.375051	3.900744
TARGET-30-PASCKI-01	6.51877	9.28344	7.3331	3.789007
TARGET-30-PASCLP-01	3.745368	7.952936	7.137927	4.034321
TARGET-30-PASCUF-01	3.687658	6.433697	7.532723	3.99668
TARGET-30-PASDZJ-01	4.239716	7.033435	7.030769	3.879637
TARGET-30-PASFRV-01	7.449633	9.240163	7.311205	4.075639
TARGET-30-PASGPY-01	3.611284	7.514235	7.423964	4.128228
TARGET-30-PASJRT-01	3.746115	6.780825	7.443037	3.967801
TARGET-30-PASJYB-01	3.602026	7.25438	7.498819	3.78745
TARGET-30-PASKJX-01	3.739696	6.315059	7.421716	4.118078
TARGET-30-PASLGS-01	3.750585	8.427546	7.227892	3.86057

Table S9. Chromosome distribution of 397 genes upregulated in younger pediatric NBL patients that are also correlated with high survival

Gene	Chromosome
C10orf113	10p12.31
CAMK1D	10p13
AKR1C1	10p15.1
AKR1C2	10p15.1
UCN3	10p15.1
DKK1	10q21.1
FAM13C	10q21.1
ANK3	10q21.2
LRRTM3	10q21.3
RUFY2	10q21.3
PPP3CB	10q22.2
DLG5	10q22.3
ZMIZ1	10q22.3
GBF1	10q24.32
XPNPEP1	10q25.1
INPP5F	10q26.11
KRTAP5-2	11p15.5
DTX4	11q12.1
C11orf10	11q12.2
SLC22A10	11q12.3
STARD10	11q13
SHANK2	11q13.3-q13.4
ARRB1	11q13.4
PDE2A	11q13.4
PGM2L1	11q13.4
RAB6A	11q13.4
USP35	11q13.4
GDPD5	11q13.4-q13.5
NEU3	11q13.5
PAK1	11q13.5-q14.1
DLG2	11q14.1
GAB2	11q14.1
KCTD21	11q14.1
NDUFC2	11q14.1
RAB30	11q14.1
RSF1	11q14.1
TMEM135	11q14.2
FAT3	11q14.3
CCDC82	11q21
CWC15	11q21
CNTN5	11q22.1
ACAT1	11q22.3
CWF19L2	11q22.3
GRIA4	11q22.3
PTS	11q22.3
RDX	11q23

ALG9	11q23.1
DIXDC1	11q23.1
SIK2	11q23.1
TIMM8B	11q23.1
DRD2	11q23.2
NCAM1	11q23.2
ZBTB16	11q23.2
ABCG4	11q23.3
ARHGEF12	11q23.3
CADM1	11q23.3
PHLDB1	11q23.3
SIDT2	11q23.3
TECTA	11q23.3
TMEM25	11q23.3
PVRL1	11q23.3
GRAMD1B	11q24.1
SCN3B	11q24.1
CDON	11q24.2
APLP2	11q24.3
ACAD8	11q25
FAR2	12p11.22
REP15	12p11.22
PPFIBP1	12p11.23-p11.22
ST8SIA1	12p12.1
PTPRO	12p12.3
DUSP16	12p13.2
GABARAPL1	12p13.2
DBX2	12q12
PRICKLE1	12q12
AMIGO2	12q13.11
CACNB3	12q13.12
LMBR1L	12q13.12
ATF7	12q13.13
HOXC9	12q13.13
IKZF4	12q13.2
LRP1	12q13.3
R3HDM2	12q13.3
GLIPR1L1	12q21.2
GLIPR1L2	12q21.2
PPFIA2	12q21.31
TMTC2	12q21.31
ANO4	12q23.1
ALDH1L2	12q23.3
HRK	12q24.22
SRRM4	12q24.23
ABCB9	12q24.31
HIP1R	12q24.31

TNFRSF19	13q12.12
FRY	13q13.1
LRRC63	13q14.13
TRIM13	13q14.2
FAM124A	13q14.3
KLHL1	13q21.33
TMCO3	13q34
ZFHX2	14q11.2
NPAS3	14q13.1
FLRT2	14q31.3
KCNK10	14q31.3
LOC100288144	14q32.33
SNORD116-27	15q11.2
SNRPN	15q11.2
LPCAT4	15q14
CATSPER2	15q15.3
CKMT1B	15q15.3
SEMA6D	15q21.1
SLC12A1	15q21.1
TMOD2	15q21.2
CGNL1	15q21.3
PYGO1	15q21.3
ANKDD1A	15q22.31
CLPX	15q22.31
THSD4	15q23
STOML1	15q24.1
SCAPER	15q24.3
MORF4L1	15q25.1
UBE2Q2P1	15q25.2
UBE2Q2P2	15q25.2
ST8SIA2	15q26.1
IGF1R	15q26.3
RBFOX1	16p13.3
FBXO31	16q24.2
ALDH3A2	17p11.2
ALKBH5	17p11.2
ATPAF2	17p11.2
DHRS7B	17p11.2
EPN2	17p11.2
MPRIP	17p11.2
NT5M	17p11.2
PIGL	17p11.2
RAI1	17p11.2
RNF112	17p11.2
TOM1L2	17p11.2
TRPV2	17p11.2
ULK2	17p11.2

USP22	17p11.2
LRRC48	17p11.2
CDRT1	17p12
CDRT4	17p12
PIRT	17p12
ZSWIM7	17p12
CHD3	17p13.1
KRBA2	17p13.1
MYH10	17p13.1
NEURL4	17p13.1
POLR2A	17p13.1
SAT2	17p13.1
C17orf107	17p13.2
CAMTA2	17p13.2
MINK1	17p13.2
SHPK	17p13.2
ZZEF1	17p13.2
C17orf85	17p13.2
OVCA2	17p13.3
PRPF8	17p13.3
RPH3AL	17p13.3
SGSM2	17p13.3
SMG6	17p13.3
SMYD4	17p13.3
YWHAE	17p13.3
CDK5R1	17q11.2
DHRS13	17q11.2
IFT20	17q11.2
KSR1	17q11.2
MYO18A	17q11.2
NF1	17q11.2
RAB11FIP4	17q11.2
SSH2	17q11.2
ARHGAP23	17q12
SOCS7	17q12
JUP	17q21.2
ANKFN1	17q22
ABCA5	17q24.3
TIMP2	17q25.3
ARHGAP28	18p11.31
EPB41L3	18p11.31
FAM59A	18q12.1
DCC	18q21.2
CCBE1	18q21.32
RNF152	18q21.33
LEUTX	19q13.2
CADM4	19q13.31

IGSF3	1p13.1
TSPAN2	1p13.2
GSTM2	1p13.3
AMY1A	1p21.1
AMY2A	1p21.1
DPYD	1p21.3
HFM1	1p22.2
MAST2	1p34.1
ST3GAL3	1p34.1
HIVEP3	1p34.2
SCMH1	1p34.2
GMEB1	1p35.3
ARHGEF10L	1p36.13
FBXO42	1p36.13
KIF1B	1p36.22
RERE	1p36.23
CHD5	1p36.31
PLEKHG5	1p36.31
B3GALT6	1p36.33
PRKCZ	1p36.33
SLC35E2B	1p36.33
SLC35E2	1p36.33
C1orf66	1q23.1
GPR161	1q24.2
CAMSAP1L1	1q32.1
OBSCN	1q42.13
TRIM67	1q42.2
C1orf57	1q42.2
SCCPDH	1q44
CSTT	20p11.21
PAK7	20p12.2
SLC23A2	20p13
DLGAP4	20q11.23
VSTM2L	20q11.23
TOX2	20q13.12
NCAM2	21q21.1
PDE9A	21q22.3
UPB1	22q11.23
ADRBK2	22q12.1
INPP5J	22q12.2
PISD	22q12.2
RBFOX2	22q12.3
SYN3	22q12.3
TAB1	22q13.1
ZC3H7B	22q13.2
GPR75	2p16.2
SPTBN1	2p16.2

PRKCE	2p21
QPCT	2p22.2
KIF3C	2p23.3
VSNL1	2p24.2
TRIB2	2p24.3
RNF144A	2p25.1
MYT1L	2p25.3
SNTG2	2p25.3
LONRF2	2q11.2
MAP4K4	2q11.2
CLASP1	2q14.2-q14.3
AMMECR1L	2q14.3
CNTNAP5	2q14.3
ZNF806	2q21.2
LRP1B	2q22.1-q22.2
ACVR2A	2q22.3-q23.1
KIF5C	2q23.1-q23.2
MBD5	2q23.2
CACNB4	2q23.3
RND3	2q23.3
BAZ2B	2q24.2
RBMS1	2q24.2
WDSUB1	2q24.2
CSRNP3	2q24.3
GALNT3	2q24.3
SCN3A	2q24.3
SLC25A12	2q31.1
SESTD1	2q31.2
INPP1	2q32.2
HECW2	2q32.3
RAPH1	2q33.2
PLEKHM3	2q33.3
ANKZF1	2q35
ZFAND2B	2q35
DGKD	2q37.1
NDUFA10	2q37.3
PTH1R	3p21.31
RBM5	3p21.31
ENTPD3	3p22.1
LRRFIP2	3p22.2
ARPP21	3p22.3
CLASP2	3p22.3
FBXL2	3p22.3
RFTN1	3p24.3
SH3BP5	3p25.1
ZFYVE20	3p25.1
CPNE9	3p25.3

HRH1	3p25.3
SRGAP3	3p25.3
VGLL4	3p25.3-p25.2
EPHA6	3q11.2
PLCXD2	3q13.2
TAGLN3	3q13.2
KALRN	3q21.1-q21.2
GK5	3q23
KCNMB2	3q26.32
TMEM44	3q29
GABRG1	4p12
CCDC149	4p15.2
ABLIM2	4p16.1
MRFAP1L1	4p16.1
KIAA1211	4q12
LOC152578	4q12
GRID2	4q22.1-q22.2
NHEDC2	4q24
DCLK2	4q31.23-q31.3
SPOCK3	4q32.3
LOC285501	4q34.3
KLKB1	4q35.2
AMACR	5p13.2
SLC45A2	5p13.2
CDH12	5p14.3
TRIO	5p15.2
PDE4D	5q11.2-q12.1
NLN	5q12.3
CARTPT	5q13.2
RGMB	5q15
PAM	5q21.1
SLCO4C1	5q21.1
PCDHGB6	5q31
CATSPER3	5q31.1
SLC22A4	5q31.1
ARHGAP26	5q31.3
PCDHA6	5q31.3
PCDHB5	5q31.3
PCDHGA11	5q31.3
PCDHGB7	5q31.3
PCDHB18	5q31.3
HTR4	5q32
MED7	5q33.3
DST	6p12.1
HMGCLL1	6p12.1
ICK	6p12.1
GSTA4	6p12.2

ABCC10	6p21.1
KLC4	6p21.1
TMEM63B	6p21.1
BTBD9	6p21.2
ANKS1A	6p21.31
NUDT3	6p21.31
ZNF76	6p21.31
PPT2	6p21.32
HIST1H1A	6p22.2
KIF13A	6p22.3
NRSN1	6p22.3
GCNT2	6p24.3-p24.2
TUBB2B	6p25.2
DOPEY1	6q14.1
SNAP91	6q14.2
AKIRIN2	6q15
BACH2	6q15
CNR1	6q15
FOXO3	6q21
GSTM2P1	6q21
HS3ST5	6q21-q22.1
RSPH4A	6q22.1
CLVS2	6q22.31
KIAA0408	6q22.33
PLAGL1	6q24.2
ARID1B	6q25.3
AGPAT4	6q26
PDE10A	6q27
MLLT4	6q27
ADCY1	7p12.3
AMPH	7p14.1
CREB5	7p15.1-p14.3
STK31	7p15.3
CLK2P	7p15.3
TMEM196	7p21.1
DGKB	7p21.2
THSD7A	7p21.3
TNRC18	7p22.1
PRKAR1B	7p22.3
SUN1	7p22.3
AUTS2	7q11.22
SEMA3C	7q21.11
PLXNA4	7q32.3
SFRP1	8p11.21
AGPAT6	8p11.21
WHSC1L1	8p11.23
DUSP26	8p12

RNF122	8p12
EXTL3	8p21.1
PSD3	8p22
XKR6	8p23.1
MMP16	8q21.3
RUNX1T1	8q21.3
MATN2	8q22.1-q22.2
NCALD	8q22.3
C9orf24	9p13.3
CNTFR	9p13.3
PTPRD	9p24.1-p23
PCSK5	9q21.13
FRMD3	9q21.32
DIRAS2	9q22.2
HSD17B3	9q22.32
ND6	MT
LOC158402	Unknown
IL1RAPL1	Xp21.3-p21.2
PTCHD1	Xp22.11
POF1B	Xq21.1
KLHL4	Xq21.31
CXorf19	Xq27.1

Table S10. Microarray values of every pediatric ALL patient for THAP4, ZNHIT2, and SF3B2.

Barcode	THAP4	ZNHIT2	SF3B2
TARGET-10-PAKSWW-03	23.657	1.4678	19.2823
TARGET-10-PAKSWW-04	35.3252	1.8751	16.5375
TARGET-10-PAMXHJ-09	13.7013	1.0392	14.0618
TARGET-10-PAMXSP-09	24.5012	0.7509	19.1507
TARGET-10-PANCVR-03	16.302	2.4834	20.2135
TARGET-10-PANCVR-04	28.7235	1.9202	29.2183
TARGET-10-PANDWE-03	6.9057	1.6935	25.5708
TARGET-10-PANEUH-09	18.3598	2.7688	27.318
TARGET-10-PANFNZ-03	21.8477	1.6861	13.5037
TARGET-10-PANGIF-03	32.7549	1.6346	25.7253
TARGET-10-PANIEU-03	25.7918	2.2864	21.4198
TARGET-10-PANJPG-09	13.8962	4.5579	19.2941
TARGET-10-PANJWJ-09	15.7102	1.3336	19.7123
TARGET-10-PANKAK-04	28.2491	1.4409	18.9668
TARGET-10-PANKAK-09	25.1778	1.524	24.9592
TARGET-10-PANKDT-03	20.5747	3.3027	18.4061
TARGET-10-PANKGK-09	32.9532	1.9348	20.1564
TARGET-10-PANKMB-09	19.0338	1.2227	22.8548
TARGET-10-PANKRG-03	17.5989	2.5385	16.0281
TARGET-10-PANLIC-09	15.5977	1.499	20.0333
TARGET-10-PANPJI-09	26.5191	1.849	20.4112
TARGET-10-PANRDC-03	12.587	2.0044	16.7548
TARGET-10-PANRWG-09	12.1346	1.1746	17.3106
TARGET-10-PANRYM-09	17.4193	1.353	16.6342
TARGET-10-PANSBR-09	15.7607	2.4287	25.5117
TARGET-10-PANSDA-04	26.1443	1.0907	22.2613
TARGET-10-PANSDA-09	17.7732	1.6406	16.2629
TARGET-10-PANSHK-04	10.9062	1.6314	16.7518
TARGET-10-PANSHK-09	11.8524	1.934	18.8286
TARGET-10-PANSIA-09	9.7546	3.6366	22.1163
TARGET-10-PANSPW-09	25.6533	2.4645	22.6411
TARGET-10-PANSUL-09	17.6699	3.865	28.305
TARGET-10-PANSXG-09	15.8384	1.2191	29.2385
TARGET-10-PANTSM-04	48.135	4.7815	29.2218
TARGET-10-PANTSM-09	37.3706	2.6774	27.6558
TARGET-10-PANUHA-09	11.5873	1.4241	29.0997
TARGET-10-PANUSN-03	20.4243	4.0185	26.9798
TARGET-10-PANUSN-04	13.7495	3.8206	18.2469
TARGET-10-PANUUF-09	14.0997	2.102	27.346
TARGET-10-PANWFB-09	48.6438	2.5614	25.4389
TARGET-10-PANWHJ-09	29.3597	3.729	23.2047
TARGET-10-PANWHW-09	87.0153	1.2744	33.9531
TARGET-10-PANWVW-09	15.298	5.5258	24.6003
TARGET-10-PANWYH-09	16.5762	3.2042	22.3377
TARGET-10-PANXDB-03	23.8377	2.7994	29.7414
TARGET-10-PANXDR-09	20.2501	3.0971	33.2722

TARGET-10-PANXPE-04	53.2073	1.6363	22.919
TARGET-10-PANYEJ-09	10.7633	0.8847	15.048
TARGET-10-PANYGB-04	46.6544	5.6657	33.7856
TARGET-10-PANYGB-09	16.6631	5.1633	18.4727
TARGET-10-PANYYV-04	18.9213	0.9075	20.9847
TARGET-10-PANYZE-09	29.5753	3.67	23.4979
TARGET-10-PANZPJ-04	26.2292	1.7925	18.766
TARGET-10-PANZPJ-09	12.3172	1.4898	24.8442
TARGET-10-PANZXZ-03	11.8367	1.2194	18.011
TARGET-10-PAPACP-04	14.8542	1.6402	21.8446
TARGET-10-PAPAGK-04	34.1236	2.0159	20.3368
TARGET-10-PAPAGK-09	37.7738	2.2957	25.261
TARGET-10-PAPAIZ-09	25.7179	1.3249	13.9822
TARGET-10-PAPBCI-09	8.1539	1.7476	22.8596
TARGET-10-PAPBES-03	17.3225	1.7338	14.5859
TARGET-10-PAPBFN-09	18.6591	2.1826	24.3249
TARGET-10-PAPBSY-09	26.5661	2.1219	22.9759
TARGET-10-PAPBZK-09	14.9229	4.0562	15.356
TARGET-10-PAPCUR-09	17.5855	2.4957	14.6176
TARGET-10-PAPCVZ-04	17.9806	1.0311	24.3896
TARGET-10-PAPDJM-04	25.7902	2.8518	18.1658
TARGET-10-PAPDKJ-09	21.1567	4.2678	20.2015
TARGET-10-PAPDUF-09	19.6011	2.7252	19.4726
TARGET-10-PAPDUV-04	14.8415	1.2945	17.0638
TARGET-10-PAPDUV-09	11.4795	2.55	14.3293
TARGET-10-PAPDWT-04	18.5224	1.0158	13.4506
TARGET-10-PAPEAB-04	19.2622	1.0936	13.0913
TARGET-10-PAPECF-04	13.7269	1.5335	17.4733
TARGET-10-PAPECF-09	35.2109	5.209	16.3139
TARGET-10-PAPEFH-04	29.0181	1.1162	19.7193
TARGET-10-PAPEFH-09	27.8023	2.8644	16.6257
TARGET-10-PAPEJN-04	16.9575	3.679	24.0453
TARGET-10-PAPEJN-09	14.2309	2.2938	22.6612
TARGET-10-PAPESW-09	15.1918	1.0712	15.2166
TARGET-10-PAPEWB-09	27.1233	1.6995	22.193
TARGET-10-PAPFWH-09	20.4691	0.9911	17.6965
TARGET-10-PAPFXN-09	14.9936	0.4294	11.3813
TARGET-10-PAPGFP-04	39.0915	2.0662	18.0081
TARGET-10-PAPGFP-09	22.4593	3.5744	15.234
TARGET-10-PAPGGT-03	19.9838	1.9776	19.1471
TARGET-10-PAPGNC-03	13.6857	1.0719	16.1174
TARGET-10-PAPGYC-09	17.7169	1.9449	21.2284
TARGET-10-PAPHEK-03	16.2579	2.997	14.3941
TARGET-10-PAPHGD-09	28.8066	2.3167	25.7006
TARGET-10-PAPHJF-04	19.4142	4.0074	21.0841
TARGET-10-PAPHJF-09	17.5517	2.3604	21.3013
TARGET-10-PAPHMH-09	34.0186	1.8555	26.8264
TARGET-10-PAPHZT-09	17.5558	1.5841	20.304

TARGET-10-PAPIGX-09	19.2278	2.0734	14.6846
TARGET-10-PAPIJB-04	25.3456	1.3469	19.5215
TARGET-10-PAPIJM-09	23.9408	1.5234	21.85
TARGET-10-PAPIRZ-09	33.8506	3.1973	17.3331
TARGET-10-PAPISG-04	44.7266	3.066	33.0258
TARGET-10-PAPJHB-04	21.0345	1.0828	13.5173
TARGET-10-PAPJHB-09	14.5685	1.2368	13.8965
TARGET-10-PAPJHR-09	25.3917	4.0166	25.158
TARGET-10-PAPJIB-04	11.9017	1.8355	7.1728
TARGET-10-PAPJIB-09	21.4107	2.5271	14.3378
TARGET-10-PAPJXI-04	16.093	1.8267	22.4631
TARGET-10-PAPJXI-09	21.7356	1.27	21.0251
TARGET-10-PAPKNC-09	27.9928	4.3057	23.4804
TARGET-10-PAPLDL-03	25.5799	2.1734	20.2182
TARGET-10-PAPLDL-04	24.8451	3.7891	18.0701
TARGET-10-PAPLDM-04	9.2945	0.6026	14.582
TARGET-10-PAPLTZ-04	12.7358	2.3355	21.5174
TARGET-10-PAPLTZ-09	14.0214	1.1625	23.0939
TARGET-10-PAPLUG-09	32.54	2.453	36.3563
TARGET-10-PAPMFI-04	8.4488	2.7471	19.0532
TARGET-10-PAPMVB-04	10.0533	0.9738	19.5788
TARGET-10-PAPMVB-09	16.3027	1.3907	15.7582
TARGET-10-PAPMYD-03	20.8803	2.8476	26.9009
TARGET-10-PAPNFY-03	11.7853	0.8546	13.9167
TARGET-10-PAPNFY-04	15.1039	0.512	10.5246
TARGET-10-PAPNMY-04	24.4562	0.9231	14.065
TARGET-10-PAPNMY-09	33.2225	1.6763	20.992
TARGET-10-PAPNNX-04	44.1175	4.7252	33.1564
TARGET-10-PAPNNX-09	53.9229	1.5102	29.7732
TARGET-10-PAPPGN-04	23.2954	1.5912	18.5071
TARGET-10-PAPRCS-04	42.0208	3.0593	23.7893
TARGET-10-PAPRCS-09	41.2036	2.7346	25.6137
TARGET-10-PAPRFE-09	31.3463	1.3231	18.575
TARGET-10-PAPSPG-04	27.2459	2.5767	26.2439
TARGET-10-PAPSPN-04	34.4578	5.718	36.0819
TARGET-10-PAPSPN-09	50.9589	2.684	27.5262
TARGET-10-PAPTLM-09	19.5454	2.3957	27.597
TARGET-10-PAPVTA-04	28.3687	1.3085	19.5098
TARGET-10-PAPVTA-09	18.1966	1.2616	14.1072
TARGET-10-PAPZNK-04	45.4853	3.3038	33.8925
TARGET-10-PAPZNK-09	49.2321	4.1748	21.7136
TARGET-10-PAPZST-04	7.0293	4.6816	8.8837
TARGET-10-PAPZST-09	22.1253	2.6366	16.6797
TARGET-10-PARACA-09	34.9521	3.7653	33.1997
TARGET-10-PARAKF-04	22.265	1.4438	24.5126
TARGET-10-PARAKF-09	24.8727	3.3176	26.8737
TARGET-10-PARARJ-09	12.9253	2.776	18.2784
TARGET-10-PARBRK-04	20.0594	0.9371	18.5888

TARGET-10-PARBRK-09	29.7582	1.8518	19.6239
TARGET-10-PARBVI-04	55.21	2.2178	19.5791
TARGET-10-PARBVI-09	27.6369	2.0026	22.2832
TARGET-10-PARCHB-03	16.1941	2.2945	18.3253
TARGET-10-PARDWE-03	22.0072	3.2627	21.5077
TARGET-10-PARFLV-04	29.8779	1.529	22.8683
TARGET-10-PARFLV-09	114.6785	4.7618	28.4539
TARGET-10-PARFTR-04	35.4291	4.562	40.4577
TARGET-10-PARFTR-09	21.2099	3.3607	27.7595
TARGET-10-PARGFV-03	60.8792	2.2793	23.2321
TARGET-10-PARGFV-04	20.4401	1.8312	20.8446
TARGET-10-PARGHW-04	23.0865	3.2333	19.4288
TARGET-10-PARGML-03	13.678	2.3717	23.4744
TARGET-10-PARGVZ-04	29.2105	0.4421	21.3608
TARGET-10-PARIAD-03	37.1165	2.8717	25.9916
TARGET-10-PARIAD-04	43.0994	2.8563	26.5021
TARGET-10-PARJSR-04	12.6222	3.1033	22.9661
TARGET-10-PARJSR-09	82.7528	3.7827	32.3711
TARGET-10-PARJZZ-04	32.7173	1.9537	21.9039
TARGET-10-PARJZZ-09	35.8858	5.4578	22.2579
TARGET-10-PARLAF-04	38.6459	2.0703	21.6471
TARGET-10-PARLAF-09	66.1896	3.7043	16.8064
TARGET-10-PARLEK-03	20.7991	6.5769	23.156
TARGET-10-PARMSP-04	27.7537	7.7822	33.3348
TARGET-10-PARMSP-09	31.7295	4.7261	24.7372
TARGET-10-PARMXF-03	20.5482	1.2905	24.4436
TARGET-10-PARMXF-04	12.5601	1.5303	19.3413
TARGET-10-PARNMF-03	13.2886	1.9056	21.3705
TARGET-10-PARNMF-04	12.619	0.8486	34.8155
TARGET-10-PARNSH-03	21.0964	2.6895	15.4271
TARGET-10-PARPNM-09	34.5894	4.4109	29.5272
TARGET-10-PARPRW-04	49.2694	3.4194	23.2089
TARGET-10-PARPRW-09	34.7326	2.598	32.7384
TARGET-10-PARPZJ-03	16.9216	3.4277	13.1564
TARGET-10-PARSGC-03	12.6102	1.8552	9.2601
TARGET-10-PARSZH-09	31.3223	3.5876	18.0507
TARGET-10-PARTJL-09	8.4635	1.5659	14.1187
TARGET-10-PARTKL-09	22.9247	1.4122	9.3937
TARGET-10-PARUBK-03	16.2548	7.2103	32.6027
TARGET-10-PARUGP-09	34.5548	3.3048	24.8643
TARGET-10-PARVWD-09	31.8372	3.7159	29.8568
TARGET-10-PARWVN-03	11.7707	2.0292	19.1491
TARGET-10-PARXMC-04	11.2144	1.1748	17.8892
TARGET-10-PARXMC-09	11.9611	2.3037	18.1149
TARGET-10-PASCIU-03	51.4059	1.2125	23.4329
TARGET-10-PASDYK-03	44.7369	0.8273	11.2998
TARGET-10-PASEVJ-09	20.4057	2.1959	13.9886
TARGET-10-PASFXA-03	23.1487	3.1606	22.9147

TARGET-10-PASFXA-04	43.4908	4.2878	29.6561
TARGET-10-PASHUI-04	10.4954	0.5496	21.7179
TARGET-10-PASHUI-09	13.4559	2.3727	16.3882
TARGET-10-PASKAY-04	37.0673	4.9782	33.0016
TARGET-10-PASKAY-09	18.7523	5.7888	20.9364
TARGET-10-PASKHT-03	14.3267	2.8705	17.3181
TARGET-10-PASLZM-40	29.7482	3.3911	22.7943
TARGET-10-PASSXJ-03	44.6797	10.8963	13.8298
TARGET-10-PASUBW-09	42.0977	9.0247	22.9592
TARGET-10-PASYGM-03	12.8519	1.6405	22.2289
TARGET-10-PATCTI-04	38.9625	3.3405	11.5932
TARGET-10-PATCTI-09	16.4416	1.4922	26.8883
TARGET-10-PATTEE-03	12.4646	4.4222	19.9023

Table S11. Microarray values of every pediatric ALL patient for COL5A1, GABBR1, HACE1, RPS6KA5, LAMB1, BMP3, MAML3, SLX4IP, EPHA7, OR52H1, DDX60L, SNORA19, SNORA2A, ENTHD2, TRIP11, ZNF81, and ZNF514.

Barcode	COL5A1	GABBR1	HACE1	RPS6KA5	LAMB1	BMP3	MAML3	SLX4IP	EPHA7	OR52H1	DDX60L	SNORA19	SNORA2A	ENTHD2	TRIP11	ZNF81	ZNF514
TARGET-10-PAKSWW-03	23.0486	17.0556	2.4427	1.089	0.0451	1.5682	10.2768	0.5324	7.2587	0.0521	3.9105	8.6997	5.3349	9.3469	1.646	1.6187	1.7701
TARGET-10-PAKSWW-04	0.2188	3.5093	0.357	0.539	0.0153	0.0297	3.5738	0.4257	0	0	5.3681	3.1822	0.3743	3.5859	0.9927	0.2916	0.1461
TARGET-10-PAMXHJ-09	21.7222	38.0206	4.7593	2.6582	0	0.1098	24.2517	3.3442	8.668	0.054	7.2699	2.7821	1.2602	14.0768	9.0754	4.4668	3.5735
TARGET-10-PAMXSP-09	17.074	16.4124	3.8127	2.2064	0.0098	0.487	19.708	1.9111	15.0745	0	17.2666	3.9072	0.6675	9.0934	3.0191	2.0805	3.3857
TARGET-10-PANCVR-03	0.598	11.3128	1.2732	0.896	0	0.3378	4.0338	1.2627	1.4805	0.0692	2.3454	3.4821	1.158	8.9858	1.2223	0.694	0.7886
TARGET-10-PANCVR-04	0.8218	3.3224	2.4315	1.3719	0.0752	0.1633	3.7266	1.25	0.3425	0.0078	2.1001	4.3289	3.0112	13.5374	1.839	0.526	0.5121
TARGET-10-PANDWE-03	5.5431	12.146	0.8769	2.3535	0.0938	0.0638	7.1683	0.7963	0.5588	0.0766	3.051	4.1522	1.3076	8.3605	2.4229	0.989	2.2321
TARGET-10-PANEUH-09	9.7087	9.9554	5.7413	1.6764	0.0072	1.9361	19.3197	0.177	1.2547	0.0612	0.9942	5.0438	1.0153	14.4501	3.8821	0.5227	1.6412
TARGET-10-PANFNZ-03	11.6735	7.5505	1.7336	1.798	0.0164	1.3536	5.8283	1.0605	1.4987	0	2.4416	2.0476	0.1959	4.0819	2.6029	0.5566	1.8844
TARGET-10-PANGIF-03	29.6072	10.7342	0.64	1.0748	0.0364	0.0194	1.1411	0.4135	0.0025	0.0698	0.8408	1.5301	2.1604	12.0508	1.4378	0.4985	0.5784
TARGET-10-PANIEU-03	21.7301	6.75	4.8655	1.888	0.1645	0.2378	4.0205	2.2467	0.0179	0.0644	4.8623	2.0477	1.6745	17.2354	5.9401	2.3498	3.7032
TARGET-10-PANJPG-09	23.6736	14.1462	3.4733	2.2918	0.015	1.8405	7.8469	1.5903	2.3484	0.0461	9.4182	1.4376	5.0219	13.5578	5.8169	1.0696	4.2134
TARGET-10-PANJWJ-09	23.9135	3.0094	2.4636	1.7644	1.0648	1.4365	5.5486	0.8943	0.145	0.0124	4.2089	1.7002	0.9396	12.1055	1.5493	1.5344	5.0213
TARGET-10-PANKAK-04	10.7732	2.4324	11.3734	2.2039	0.1751	0.9948	2.7918	1.1915	0.008	0.0342	0.6733	1.5217	1.7896	9.1054	1.4409	1.9217	2.7924
TARGET-10-PANKAK-09	35.0786	11.641	12.076	1.3127	0.0957	25.677	4.5371	1.8902	0.2893	0.0153	6.1372	5.5007	5.2192	10.2376	4.0856	2.3475	6.1385
TARGET-10-PANKDT-03	1.7251	19.3673	3.1629	2.3575	0.0138	4.9958	5.3935	1.7897	1.8398	0.0588	4.6229	2.6298	2.2962	20.9846	6.8903	2.1442	4.3333
TARGET-10-PANKGK-09	12.1013	6.2734	0.9236	1.1678	0	1.1355	9.3561	0.7295	1.2706	0	2.5055	2.4037	1.6562	6.097	2.8738	0.334	0.4196
TARGET-10-PANKMB-09	31.0769	27.1335	6.1919	1.6325	0.0508	0.7371	6.3349	4.1765	7.9066	0.1028	6.1681	2.1391	1.3502	31.631	8.002	3.6985	9.8998
TARGET-10-PANKRG-03	65.3705	57.8465	6.772	2.6962	0.1581	0.1425	16.2163	3.0063	35.1572	0.3195	7.3488	4.7037	3.591	19.9292	7.3803	5.8014	8.9403
TARGET-10-PANLIC-09	18.9113	16.7046	4.3408	2.1248	0.1075	3.1863	22.102	2.9532	12.0323	0.2693	5.0172	2.082	3.4651	15.4877	7.2501	3.8056	6.4095
TARGET-10-PANPJI-09	8.2976	4.245	2.219	1.8705	0.042	7.6781	11.2841	0.9229	0.9614	0.0971	3.7682	4.0495	2.0622	8.9203	2.9302	0.7627	2.0156
TARGET-10-PANRDC-03	25.9366	9.9342	1.8006	2.549	0.0361	11.8916	12.701	0.8386	1.6595	0.2045	5.6646	6.3781	3.6102	12.6665	3.3599	1.2257	2.2252
TARGET-10-PANRWG-09	19.8244	4.7452	4.078	2.7314	0.2658	0.9783	22.2226	2.5893	0.1753	0.214	3.7466	13.2887	4.2678	9.8229	2.8452	2.1931	1.2514
TARGET-10-PANRYM-09	23.4852	27.4104	3.7399	1.3387	0.0082	0.0876	32.8789	0.7911	7.5998	0.1152	8.6419	1.3496	1.538	15.659	5.7391	1.7909	3.6234
TARGET-10-PANSBR-09	47.3484	15.2835	2.7922	1.8378	0.0077	0.347	4.25	1.1755	2.869	0.0376	5.2853	1.42	1.6432	16.862	2.572	0.975	1.1205
TARGET-10-PANSDA-04	21.1834	3.0193	3.1967	1.2034	0.3354	0.0434	8.7688	0.9312	1.1855	0.0213	0.8093	2.1089	0.6023	5.0029	2.1253	1.3117	1.61
TARGET-10-PANSDA-09	61.8679	13.6945	4.4245	1.6226	0.0727	0.1856	9.3005	2.0769	3.9885	0.1517	4.0982	6.0343	2.3674	8.0516	4.9078	2.1988	3.1727
TARGET-10-PANSHK-04	17.7077	22.2729	5.8649	3.4782	0.6422	0.8738	7.1727	4.0637	21.6993	0.5873	8.0784	7.5044	6.0937	12.9794	9.8285	3.3405	7.6145
TARGET-10-PANSHK-09	39.768	42.9082	7.7647	2.2142	0	0.335	6.983	5.4185	18.5692	0.8343	6.2268	6.4998	5.3253	18.3408	8.4622	4.3235	15.8974
TARGET-10-PANSIA-09	20.9704	30.5474	6.9231	3.108	0.3474	7.4571	25.6591	3.0402	11.787	0.1663	7.6461	8.3146	20.0951	13.195	2.9449	1.8513	1.8146
TARGET-10-PANSPW-09	26.5215	19.5944	2.8249	1.662	0.0653	0	4.8536	1.0842	0.5034	0.043	4.1426	3.9383	1.7969	11.485	1.83	1.4795	3.68
TARGET-10-PANSUL-09	17.3954	15.3833	2.2515	1.825	0.4347	1.6661	3.6515	0.3649	1.5745	0	1.7727	4.3063	3.2979	16.0048	2.5532	0.5296	0.8681
TARGET-10-PANSXG-09	9.3152	11.9055	2.7236	2.3833	0.0814	0.0564	12.4899	0.8422	2.9506	0.0305	3.7893	5.4079	2.8518	7.6804	3.3434	1.0457	0.7961
TARGET-10-PANTSM-04	7.0781	8.1197	1.0371	1.3418	0.3453	0.3323	7.6724	1.0127	0	0.0405	0.6125	0.4907	0.4125	6.3962	1.0662	0.4214	1.0462
TARGET-10-PANTSM-09	7.2668	3.1602	0.9627	1.6164	0.041	0.4608	8.3648	0.2599	0.0088	0.0295	0.5096	3.6172	3.0346	14.1821	1.5753	0.7333	0.1739
TARGET-10-PANUHA-09	30.0235	23.5721	3.4541	2.6743	2.2431	2.9264	12.611	1.2429	4.5801	0.0691	5.2309	2.8825	2.2602	13.3966	5.5495	2.2264	1.9552
TARGET-10-PANUSN-03	9.67	10.1629	1.0891	4.7977	0.0196	1.8049	3.436	0.5236	3.4869	0.033	1.6564	3.0853	0.916	11.4142	3.5772	0.3923	0.5154
TARGET-10-PANUSN-04	3.049	1.5585	1.8678	1.0091	0.0052	0.8155	2.245	0.9468	0	0	2.1389	2.5879	3.3238	6.9847	3.8422	0.384	0.5082
TARGET-10-PANUUF-09	21.0955	4.2534	1.5073	2.8793	0.0395	3.4151	8.0198	0.3501	1.722	0.1123	1.1849	3.426	3.6189	7.0558	5.2796	1.1897	0.2564
TARGET-10-PANWFB-09	14.2581	1.4504	0.7104	1.2058	0.0305	0.4485	7.9501	0.2727	0.2994	0	0.8819	1.5853	1.216	10.8459	1.4524	0.4654	0.0677
TARGET-10-PANWHJ-09	15.9746	4.2904	1.4978	0.7441	0.0012	20.652	56.8784	0.2127	1.9774	0	1.3389	0.178	0.2279	6.5324	1.8002	1.1672	0.2047
TARGET-10-PANWHW-09	0.2422	3.9591	0.9649	4.0911	0.0502	0.2733	3.9637	0.421	0.4686	0.0986	0.7702	2.4856	2.4054	12.1954	4.7101	1.4949	0.2582
TARGET-10-PANWVW-09	18.8938	7.4603	3.3103	2.1955	0.0279	23.7614	16.113	0.8508	0.9158	0.0267	0.9229	2.1284	0.8107	8.9902	2.3159	0.5976	0.8415
TARGET-10-PANWYH-09	29.5712	10.8832	5.1955	2.8383	0.3161	0.5635	6.5101	2.4613	5.1996	0.239	5.3505	1.7107	2.086	10.4435	10.6439	3.2134	2.2628
TARGET-10-PANXDB-03	6.1624	1.7497	2.0557	2.9703	0.1569	0.8217	4.8698	0.8925	13.2685	0.4628	4.5797	9.0889	2.0845	5.457	5.2407	0.8183	1.2102
TARGET-10-PANXDR-09	31.0912	9.0979	3.5994	2.8037	0.0105	0.0149	7.7069	0.4803	1.0447	0.0646	2.1134	1.912	1.399	13.8288	2.5927	2.2183	1.3667
TARGET-10-PANXPE-04	8.3297	5.6234	0.9797	0.743	0.0094	0.2936	7.1773	0.3952	2.8943	0	0.714	2.1163	2.864	6.709	1.5246	0.2118	0.8044
TARGET-10-PANYEJ-09	25.9318	11.59	6.9006	2.7019	0.3342	3.8587	18.5363	1.5806	8.1409	0.6235	4.3638	5.3162	8.1896	6.9291	4.1392	1.0827	1.8472
TARGET-10-PANYGB-04	2.0779	0.7546	1.345	1.2325	0.4663	0.0028	3.0056	0.3165	0.0052	0	0.8246	2.7723	3.4132	7.0968	1.7555	0.2796	0.1852
TARGET-10-PANYGB-09	0.1313	0.5263	1.0254	1.3532	0.0588	0	13.158	0.9572	0	0.1815	0.4513	3.7339	0.2548	4.7131	5.6809	1.2827	0.6379
TARGET-10-PANYYV-04	33.8209	10.0651	5.3086	1.9594	0.0057	0.9544	17.2508	1.6424	5.0883	0	3.4844	3.7024	3.8508	7.6571	2.5702	1.96	2.7021
TARGET-10-PANYZE-09	12.4218	6.4415	4.5466	1.9485	0.0055	0.9788	13.6039	0.6412	13.7713	0.0549	3.584	3.5314	1.7557	7.6228	5.2656	4.1597	4.1361
TARGET-10-PANZPJ-04	2.93	3.8769	0.5821	1.2508	0.6102	0.0277	3.6456	0	0.201	0	0.5713	0.2989	0.8679	6.6314	1.3641	0.4518	0.234
TARGET-10-PANZPJ-09	10.1435	14.9304	3.4021	3.1648	0.0557	0.0702	11.4877	0.2736	2.3789	0.2204	8.1194	10.3689	3.6248	14.5393	3.8653	2.9132	1.535
TARGET-10-PANZXZ-03	9.9104	11.1323	1.1387	2.938	0.1722	0.4687	7.6892	1.4712	1.9094	0.0725	6.1479	2.5815	0.7707	9.272	4.7531	1.3954	2.3258
TARGET-10-PAPACP-04	45.3968	19.2416	7.4353	4.2249	0.02	4.6792	3.087	1.4163	5.8006	0.4389	3.7908	1.9692	3.0938	19.2192	8.6831	2.144	5.691
TARGET-10-PAPAGK-04	2.6712	0.8497	0.4797	0.6092	0.0248	0.0068	4.0429	0.5494	0.269	0	0.6133	0.5897	0.5815	7.1204	0.718	0.183	0.123
TARGET-10-PAPAGK-09	36.3689	1.8272	0.6969	0.2768	0.0398	0.0917	8.4229	0.7392	1.8055	0.0381	0.6791	0	1.749	13.371	0.5237	0.2077	0.4204
TARGET-10-PAPAIZ-09	66.8495	18.7368	2.5774	0.7881	0.0089	1.1768	18.6929	0.6256	2.1481	0.0114	1.0905	1.4352	1.3715	7.5841	1.1605	0.567	2.7637
TARGET-10-PAPBCI-09	8.9313	7.9827	2.3668	1.3297	0.9613	0.2238	6.8547	0.872	6.4854	0.0232	1.8679	5.3477	3.2944	10.7401	1.449	0.7707	1.7027
TARGET-10-PAPBES-03	22.2484	28.249	2.5392	1.1949	0.0186	0.4532	11.7022	1.5127	6.6972	0.0522	4.1229	3.7452	0.9367	12.6186	4.1661	1.7449	3.0747
TARGET-10-PAPBFN-09	17.1245	14.9531	3.2728	1.8937	0	0.0451	17.1687	1.1428	2.8351	0.1996	7.3836	1.1618	1.6438	6.3019	4.9824	2.3203	1.6903
TARGET-10-PAPBSY-09	14.2051	2.9104	2.1936	0.7451	0.0026	0.0206	4.3972	0.7267	0.0245	0	0.9759	2.4777	1.2922	5.0146	0.4382	0.2351	0.2172
TARGET-10-PAPBZK-09	17.26	10.7887	2.4314	1.5342	0	0.0894	2.7242	1.2412	3.6229	0.1558	4.7478	0.155	1.1865	7.1625	3.3853	0.7198	1.8289
TARGET-10-PAPCUR-09	8.9563	3.4476	4.653	1.3643	0.0259	12.7309	5.0364	0.9768	0.4325	0	2.8679	0.2494	0.078	5.0066	1.5371	0.6496	1.4719
TARGET-10-PAPCVZ-04	22.7125	16.4887	4.766	2.4524	0.0087	0.4804	19.1077	1.7762	4.0331	0.0838	6.904	6.2871	5.8754	11.178	2.5391	1.9787	2.0077
TARGET-10-PAPDJM-04	58.7932	10.7965	0.8596	0.4682	0	1.9966	10.5938	0.8446	0.9494	0	0.7099	0.3195	2.3514	21.4767	1.3119	0.2593	2.2025
TARGET-10-PAPDKJ-09	64.2227	26.251	2.1729	1.6446	2.7881	9.4816	12.138	1.7143	5.1355	0.4521	10.4693	5.7766	7.5491	12.2684	5.6273	1.423	3.2653
TARGET-10-PAPDUF-09	25.6694	16.4829	1.9756	1.106	0.0087	11.4911	36.7403	0.8538	1.7306	0.0664	2.9324	0.7066	0.1567	11.7432	3.1808	1.4015	2.2976
TARGET-10-PAPDUV-04	2.2267	17.6266	3.8957	1.1711	0.3615	0.6291	24.9174	3.7838	15.3797	0.2621	2.7633	3.8638	2.9564	12.9421	5.6721	3.8535	4.8173
TARGET-10-PAPDUV-09	7.2317	26.7962	4.0768	1.7742	0.0514	3.9741	22.3918	3.4636	16.3689	0.2723	2.4058	3.469	4.8399	11.5372	8.0128	3.1996	4.5973
TARGET-10-PAPDWT-04	63.5368	7.9902	4.1106	1.4921	0.0512	0.02	6.103	0.9825	2.3494	0.0154	3.6527	2.0549	1.0779	4.522	1.8391	0.7452	3.0182
TARGET-10-PAPEAB-04	48.7966	12.0329	3.7548	1.9263	0	0.092	22.2475	0.6627	3.7264	0.0183	2.515	4.7479	1.9614	5.8832	2.4329	1.1302	1.2029
TARGET-10-PAPECF-04	43.3133	17.7405	6.0174	2.9216	3.9958	3.9104	15.4806	2.0425	4.842	0.3945	21.7164	6.7351	6.6227	13.5266	6.1473	3.7499	7.9692
TARGET-10-PAPECF-09	19.0805	8.1342	1.0298	0.5714	0.381	1.177	10.8528	0.4216	0.4414	0	1.1366	0.1095	1.2964	11.6754	1.8328	0.4273	2.292
TARGET-10-PAPEFH-04	49.5218	5.4233	1.4358	0.4515	0.0939	0.6496	5.3631	1.9599	1.1714	0	5.3906	0.387	1.2989	4.3759	0.6367	0.8959	1.0366
TARGET-10-PAPEFH-09	86.712	6.1847	1.4312	0.2761	0.0095	1.2493	10.3404	0.8214	1.896	0.1462	1.0385	0.4259	0.426	7.5984	1.05	0.9822	1.3104
TARGET-10-PAPEJN-04	33.4564	17.1849	6.3254	1.9853	0.5514	0.4243	10.4003	2.9387	16.4532	0.0367	9.0475	2.8997	5.5874	9.0664	4.0696	2.0267	3.8575
TARGET-10-PAPEJN-09	44.3786	26.6262	10.9157	2.8825	8.2069	1.4033	19.9035	3.9798	14.1758	0.83	17.9156	7.113	4.7759	9.1499	6.4357	4.5334	8.0659
TARGET-10-PAPESW-09	52.2856	14.8945	6.0182	3.3292	0.0127	3.2555	21.566	2.0796	8.4926	0.4755	5.8969	5.5787	3.7047	11.0089	5.7944	3.0095	4.4199
TARGET-10-PAPEWB-09	18.2846	0.8624	2.8513	1.1433	0.1346	0.0546	11.5345	0.598	0.0555	0.041	4.4457	3.338	2.3611	7.7087	1.2585	1.1324	0.9387
TARGET-10-PAPFWH-09	24.3357	13.1449	4.9912	0.8855	0.0526	0.0792	5.7269	2.178	0.6059	0.0526	2.8061	1.1831	0.5929	9.5872	5.27	1.7436	3.2468
TARGET-10-PAPFXN-09	15.4856	14.6227	1.969	1.3093	0.0584	0.8146	12.9258	1.2796	5.5358	0.0003	3.7498	3.738	2.2619	3.8674	2.1088	0.5748	1.2259
TARGET-10-PAPGFP-04	3.8838	3.4496	3.6089	0.4526	0.0049	0.1504	4.0871	1.0714	1.1309	0	1.5643	0.2569	0.1767	5.7659	0.7703	0.4248	1.6573
TARGET-10-PAPGFP-09	13.3885	4.8728	5.0536	0.9098	0.1937	0.2073	3.6803	1.557	0.6457	0.1854	3.4722	2.1808	3.249	12.8036	1.82	1.2646	1.6637
TARGET-10-PAPGGT-03	13.1672	16.0588	2.974	1.4325	0.0116	0.5298	8.0558	1.3778	8.4502	0.0754	5.3852	3.2307	1.2528	9.0911	5.1674	2.3216	2.3768
TARGET-10-PAPGNC-03	7.4589	10.8614	2.8292	3.5646	0.1503	0.1983	6.8504	1.4894	1.6838	0.2047	6.3249	7.3047	1.1468	15.3037	3.6423	3.2484	4.4387
TARGET-10-PAPGYC-09	40.102	17.6936	4.6068	1.8256	0.0035	0.091	14.6593	1.8792	15.0981	0.1117	6.2888	3.9841	4.1783	9.5197	5.07	3.6113	4.0492
TARGET-10-PAPHEK-03	1.3241	0.7219	1.3953	1.1074	0.6304	0.0387	3.9149	0.9	0.4409	0	2.6111	0.2471	0.2559	6.2315	1.2955	0.4902	1.4341
TARGET-10-PAPHGD-09	19.6457	13.3859	4.8115	1.7992	0.1031	0.0028	7.267	1.6829	7.142	0.0151	8.0687	0.9529	2.7681	11.9381	5.8661	2.1664	2.6739
TARGET-10-PAPHJF-04	2.4796	6.2008	4.296	3.7606	0.12	0.0077	3.4358	3.6115	0.034	0.1958	12.5482	1.9628	1.7647	10.732	10.9614	3.4113	9.8412
TARGET-10-PAPHJF-09	30.6164	19.1433	4.097	1.8327	0.0104	0.7307	5.5144	2.4261	0.0614	0.0679	7.7913	0.8427	0.8053	11.7394	8.4584	3.5571	7.8661
TARGET-10-PAPHMH-09	0.0189	1.8678	0.5686	0.3838	0.0356	0.0455	3.5255	0.3197	0.0233	0.0234	0.5927	3.2452	3.8085	6.1432	1.5676	0.2473	0.4648
TARGET-10-PAPHZT-09	30.6321	16.0853	4.4233	2.0546	0.0468	1.4691	9.7823	3.2399	1.4526	0.1807	13.301	4.0033	2.8343	13.4544	4.5893	2.6771	4.563
TARGET-10-PAPIGX-09	7.3024	4.1301	3.863	2.0151	0	7.4917	4.0729	0.9822	0.0579	0.0955	4.9356	0.4356	0.9549	15.357	3.9352	1.2879	0.9761
TARGET-10-PAPIJB-04	37.8069	14.3778	2.6425	0.6503	0.0318	0.2825	10.1996	1.3651	1.3256	0	1.2019	0.6739	0.3573	10.0749	1.0852	0.8314	2.7694
TARGET-10-PAPIJM-09	46.4212	16.4529	3.6099	2.1154	0.0556	0.0049	11.4711	1.3911	19.8804	0.0491	4.0715	1.9379	1.0888	7.948	3.2548	2.2738	2.0907
TARGET-10-PAPIRZ-09	0.1738	7.2967	1.1238	0.6994	0.0066	0.0094	4.5718	0.7201	0.1955	0	1.8812	0.6706	0.9358	6.7097	1.3424	0.4993	0.4708
TARGET-10-PAPISG-04	27.7206	7.3405	2.948	1.1716	0.0563	1.3839	3.7032	0.8412	1.0863	0.054	1.1728	0.3575	0.1804	7.1575	1.747	0.5742	2.2099
TARGET-10-PAPJHB-04	14.3875	18.3623	2.7217	1.4509	0.1173	0.3675	14.8406	2.2175	8.7329	0.4338	3.5023	8.0967	5.8489	9.9457	2.5505	2.2011	2.883
TARGET-10-PAPJHB-09	17.8146	19.7246	4.1245	2.1203	0.0682	0.9023	11.5329	3.3232	9.5281	0.0684	4.0061	7.8924	5.4267	9.3192	5.4374	3.1462	5.9959
TARGET-10-PAPJHR-09	21.3661	10.5309	2.6957	1.6236	0.1062	0.0861	5.0988	0.6806	4.6819	0.055	1.3966	3.1929	3.401	11.9202	2.0094	0.4342	2.1625
TARGET-10-PAPJIB-04	17.6761	4.8786	2.0225	1.0726	0.0334	0.5129	7.9853	0.4499	0.4361	0	1.3043	0.2568	0.7084	4.3867	1.6093	0.5121	1.3473
TARGET-10-PAPJIB-09	37.5606	8.9044	2.2601	1.1629	0.0097	2.8884	20.0161	1.2164	5.3596	0.0747	3.3961	1.9318	0.7015	8.6252	1.7405	0.6867	1.8192

TARGET-10-PAPJXI-04	23.8203	9.1773	11.8185	2.3743	0.057	1.4452	19.1059	3.6574	11.3695	0.4191	7.4743	5.6591	6.6696	10.2704	6.4758	3.439	4.6005
TARGET-10-PAPJXI-09	15.8885	9.3074	7.4568	1.0109	0.2853	1.847	11.8832	2.7705	4.7023	0.049	2.3817	3.1161	2.7744	7.3913	1.9215	1.4972	3.2489
TARGET-10-PAPKNC-09	12.0128	3.6731	0.6599	1.4653	0.0209	0	3.6345	0.4481	0.0421	0.037	2.217	8.8082	2.6207	6.5508	2.0988	0.6892	0.4683
TARGET-10-PAPLDL-03	25.386	14.42	3.7012	1.1663	0	1.6316	9.7605	1.6393	8.7018	0.0574	2.3808	3.5564	2.9334	13.9398	1.8778	1.0871	1.7341
TARGET-10-PAPLDL-04	26.7925	3.2759	1.556	0.5025	0.1296	4.5721	13.2191	1.2819	1.0344	0	0.7825	0.0127	0.6129	13.302	1.8764	0.7345	1.4853
TARGET-10-PAPLDM-04	47.5044	24.3807	7.6113	2.4425	0.0411	18.7152	44.4617	3.1832	6.5739	0.1352	6.5774	3.5659	3.1414	15.4605	4.4066	2.7398	4.667
TARGET-10-PAPLTZ-04	13.9357	22.4088	2.5845	1.4716	2.5813	19.2851	10.0383	3.4457	10.3112	0.1332	8.2686	3.8635	4.7041	13.2149	10.5466	2.9818	8.7298
TARGET-10-PAPLTZ-09	18.216	23.0827	2.9994	1.3712	1.8269	5.653	8.7258	2.1528	7.0589	0	3.2245	3.0937	1.7429	5.636	1.7344	0.9823	7.4274
TARGET-10-PAPLUG-09	11.7612	10.8252	4.4563	1.7229	0.1079	14.2501	7.4854	0.7477	1.9494	0	7.9996	7.6354	4.9861	11.0285	3.1177	0.8111	0.488
TARGET-10-PAPMFI-04	6.6829	18.5642	4.0595	1.8688	3.654	0.4458	7.1717	1.0294	2.3724	0.0183	2.359	11.3855	7.6391	12.6811	2.3945	0.9908	2.9788
TARGET-10-PAPMVB-04	16.3176	10.4533	3.0049	1.8931	3.3592	2.4883	9.2682	1.6038	2.6436	0.056	1.6604	4.0679	2.8368	9.0531	1.427	1.1331	1.189
TARGET-10-PAPMVB-09	20.6843	18.1183	3.7755	1.4063	4.5146	8.8974	7.6153	1.7279	2.1409	0.2854	2.9748	4.5298	2.7178	10.6782	1.3639	1.1233	1.6652
TARGET-10-PAPMYD-03	14.87	3.8724	1.9059	1.9032	0.0058	0.0082	0.9174	0.4564	0	0.5303	2.6587	4.3368	0.9583	8.5323	4.8954	1.3496	0.7157
TARGET-10-PAPNFY-03	18.4201	11.1316	3.4013	2.835	0.1844	1.3442	31.9203	1.6435	3.4285	0.1311	8.8196	4.5745	1.7571	9.9157	6.4098	2.8049	2.7764
TARGET-10-PAPNFY-04	15.4328	5.0649	2.216	2.6706	0.1052	0.6309	19.1411	0.0932	0.8805	0.1398	4.2699	2.9383	0.9878	5.0396	3.9914	1.7771	0.6207
TARGET-10-PAPNMY-04	0.4496	8.6004	2.593	0.9269	0.0825	0.5178	23.7449	1.6289	6.8111	0.0573	2.6662	4.5937	2.4023	8.145	1.5395	0.7342	1.5303
TARGET-10-PAPNMY-09	12.1789	16.9548	4.281	1.4862	0.0326	2.0403	22.7583	1.7402	6.0276	0.1242	5.3221	1.6095	1.515	10.1623	3.161	1.3594	2.6741
TARGET-10-PAPNNX-04	0.0134	1.5421	0.8899	0.6452	0.1724	4.0337	5.5619	0.5721	0.0213	0	0.1672	0.3169	0.6762	4.5776	0.367	0.1981	0.178
TARGET-10-PAPNNX-09	0.2173	3.0597	1.1724	1.7098	0.1682	3.2343	6.9272	0.3891	0.0753	0.3001	0.8884	1.0532	1.112	7.075	3.2925	1.1086	0.2875
TARGET-10-PAPPGN-04	13.3759	3.4548	2.5109	1.4044	0.0327	0.1523	6.0954	1.1536	0.2453	0.1379	2.4802	1.6955	1.3485	7.2494	2.6608	0.7305	1.5571
TARGET-10-PAPRCS-04	1.0707	1.9849	1.3248	0.4238	0.0156	0.0025	3.4306	0.3009	0	0	0.6729	0.3951	0.3344	3.6257	0.7657	0.2802	0.5858
TARGET-10-PAPRCS-09	1.1046	6.9094	2.2045	1.0395	0.0153	0.0081	5.882	0.6633	0.5364	0.0146	1.6276	1.156	0.4367	5.3887	2.4355	0.6728	1.806
TARGET-10-PAPRFE-09	6.8859	4.8987	2.6731	1.3988	0.0108	0.0639	9.7384	0.6158	0.0067	0.6729	1.7022	3.24	1.9577	7.8999	5.3267	1.2848	1.76
TARGET-10-PAPSPG-04	16.8266	2.2746	1.777	0.6059	0.0251	0.0443	6.0497	1.5596	0.0587	0	0.6595	0.5862	0.728	4.0489	0.8048	0.5495	1.469
TARGET-10-PAPSPN-04	5.4296	2.4868	1.5789	0.6995	0.1774	0.1453	8.59	1.1712	0.036	0	0.7541	0.4273	0.4358	4.1938	0.7917	0.2738	0.9604
TARGET-10-PAPSPN-09	11.3537	5.608	0.5161	0.3055	0.0679	0.0031	7.2245	1.0745	0.02	0.0168	0.4795	0.0035	0.1322	5.9169	0.4697	0.2992	1.2029
TARGET-10-PAPTLM-09	12.8729	5.7541	2.3414	1.4181	0.0174	0.0597	5.986	1.1884	0.0302	0.3889	2.2081	10.5255	3.9186	6.9121	1.797	0.519	0.5022
TARGET-10-PAPVTA-04	28.6541	3.6601	1.9341	1.5786	0.0113	5.7288	19.4869	0.5372	0.9301	0.0173	1.7035	0.2837	0.8693	6.0332	1.1732	0.9771	0.2561
TARGET-10-PAPVTA-09	69.07	25.5861	8.7782	2.7823	0.0177	3.6746	30.206	5.0932	12.4953	1.5649	8.6558	10.5316	9.3295	13.2934	5.2742	4.7303	4.4308
TARGET-10-PAPZNK-04	0.0297	0.5226	0.8145	0.2284	0.2378	0.0722	5.0907	0.936	0.5692	0	0.6029	0.325	0.1461	3.0953	0.3788	0.2193	0.4877
TARGET-10-PAPZNK-09	0.0124	3.4188	0.413	0.2294	0	0.0122	5.7933	0.4459	0.1695	0	0.8012	0.2459	2.1296	17.0091	0.8622	0.1877	0.312
TARGET-10-PAPZST-04	0.408	0.1049	0.2594	0.3782	0	0.3617	0.5688	0.1637	0	0	0.5276	0	3.0367	2.4824	0.7366	0.136	0.2306
TARGET-10-PAPZST-09	9.5827	6.128	1.9176	0.7063	0.0449	0.4374	7.1294	2.0318	1.1556	0.0108	2.0195	0.5448	0	9.1513	2.3821	0.978	1.8995
TARGET-10-PARACA-09	1.0608	1.0807	1.2537	0.6226	0	0	1.8192	1.0614	0.008	0	1.3029	0	0.5179	6.4103	1.6567	0.5379	0.9538
TARGET-10-PARAKF-04	14.2868	7.9922	1.2189	0.8777	0.8544	0.1495	8.6553	1.3823	0.8355	0	0.5073	0.5544	1.4238	9.8725	0.5211	0.2929	0.9566
TARGET-10-PARAKF-09	15.1124	17.3401	5.0688	1.2299	1.4943	1.5844	16.6762	2.3202	4.5454	0.1484	4.3431	4.0277	7.3963	12.5726	2.9791	1.6086	3.1091
TARGET-10-PARARJ-09	40.6252	6.6221	6.4685	3.3068	0.0161	0.0164	15.5042	3.4309	27.261	0.4123	6.429	5.481	3.5108	8.9263	7.464	2.8553	2.2611
TARGET-10-PARBRK-04	44.9488	10.5458	2.3809	0.9703	0.0218	5.9455	10.434	1.6919	0.93	0.0167	2.1198	2.2139	1.4141	9.1051	1.7028	1.5915	2.9268
TARGET-10-PARBRK-09	43.1424	7.5805	1.3183	0.6387	0.0167	0.3482	25.1332	0.5736	1.5204	0	0.7113	0.3426	0.2825	6.9115	1.2232	0.4809	1.2472
TARGET-10-PARBVI-04	3.317	3.873	0.3699	0.4254	0.0731	0.0066	12.2771	0.6472	0.1428	0	0.5796	0.1827	0.1171	3.924	0.3899	0.2134	0.5888
TARGET-10-PARBVI-09	16.0781	5.9438	1.3873	1.7083	0.0072	0.0972	17.071	1.0666	3.3782	0	1.7832	4.5547	1.2194	4.769	4.944	2.7498	1.3984
TARGET-10-PARCHB-03	28.198	16.2365	3.0626	1.6292	0.0773	2.1838	17.3166	2.035	2.1588	0.3645	12.6839	4.4866	3.5364	12.9937	8.557	2.8088	4.3741
TARGET-10-PARDWE-03	0.7745	18.4934	2.8844	1.4751	0.0214	0.086	8.875	5.0533	2.6082	0.2675	5.2259	1.338	0.7589	18.3378	7.5406	3.6947	7.4639
TARGET-10-PARFLV-04	14.4619	9.1853	3.2667	1.944	0.0965	0.0054	7.7606	0.8331	0.0951	0.0872	2.0985	4.8673	2.9316	7.1225	2.0045	0.6463	0.8133
TARGET-10-PARFLV-09	15.2435	3.1928	0.7427	0.9432	0.0018	0	5.9041	0.1703	0.0977	0	0.4971	0	0.278	4.6424	1.1945	0.1928	0.1306
TARGET-10-PARFTR-04	0.0713	4.5533	1.452	0.3442	0.0058	0	0.4842	1.5074	0.0072	0	1.1471	0.4363	0.5129	5.2159	0.9587	0.4605	2.1498
TARGET-10-PARFTR-09	0.3474	2.6552	1.266	0.8227	0.0532	0.2115	0.8409	0.3083	0.0293	0.0376	1.9589	3.0728	1.3217	9.3073	1.6173	0.3288	0.6449
TARGET-10-PARGFV-03	17.2036	4.9671	0.9803	0.4384	0.034	0.0027	6.498	0.6273	0.0389	0	0.4668	0.4409	0.8005	4.8332	0.6771	0.1839	0.3843
TARGET-10-PARGFV-04	52.2207	17.8579	2.5415	1.3008	0.0903	0.0726	19.9803	1.5953	3.12	0	1.9383	0.826	0.8911	7.8251	1.8517	0.42	0.8365
TARGET-10-PARGHW-04	2.3933	12.6013	0.7174	0.311	0.0191	0.363	6.6743	0.863	0.3985	0	0.2552	0.5369	0	6.5004	0.7236	0.3557	0.6953
TARGET-10-PARGML-03	0.1355	3.7504	1.0527	4.1902	0.0054	0.0268	1.4713	1.1081	0	0	4.0009	0.99	0.7467	7.4155	6.195	0.9177	0.2585
TARGET-10-PARGVZ-04	54.0308	0.7158	7.8188	1.3891	0.014	9.8882	13.7251	2.3401	4.537	0.0212	4.825	0.1361	0.5892	9.3776	6.0756	2.8154	3.7971
TARGET-10-PARIAD-03	23.0047	2.2134	0.7453	0.669	0.0502	0.1258	3.7931	0.233	0	0.0227	1.0628	0.2264	0.653	9.8982	1.7767	0.6032	0.4177
TARGET-10-PARIAD-04	25.877	6.4123	1.4269	1.4562	0.0859	0.0086	2.9841	0.8351	0.0243	0	1.2471	0.4442	0.5747	5.9793	1.7401	0.13	0.4436
TARGET-10-PARJSR-04	12.4843	8.4761	4.0394	2.0133	0.1625	0.1076	11.0663	1.5709	14.1452	0.1224	1.2162	1.7793	1.7363	16.7838	5.4904	2.087	3.3448
TARGET-10-PARJSR-09	1.5657	4.5648	1.3007	1.3995	0.3157	0.0065	7.1954	0.4119	2.224	0	0.13	0.2592	0.549	7.542	1.0663	0.4008	0.3024
TARGET-10-PARJZZ-04	8.8775	0.4753	0.8631	1.3313	0.018	0.0041	1.5164	0.695	0.4785	0.0173	0.5719	0.6185	0.8678	9.0007	1.0675	0.1857	0.5025
TARGET-10-PARJZZ-09	10.166	1.1791	3.0229	0.9964	0.0066	0.03	2.5441	1.7693	0.7191	0	2.4385	0.2725	0.52	8.1449	2.8011	0.6502	2.2794
TARGET-10-PARLAF-04	5.1521	1.4108	1.6002	0.8268	1.6011	2.9329	7.1235	0.2353	0.3063	0	0.2919	0.8841	0.6199	5.8558	2.4769	0.1625	0.3481
TARGET-10-PARLAF-09	8.6359	6.0344	0.2646	0.579	0.0713	1.1405	12.1348	0.3295	0.1221	0	0.139	0	0.634	6.2093	1.1489	0.1097	0.1666
TARGET-10-PARLEK-03	0.0177	2.1446	0.8621	0.776	0.0061	0.1452	15.7382	0.5597	0.0417	0	0.5219	0.6355	0.0822	4.3023	1.7623	0.3049	0.5774
TARGET-10-PARMSP-04	6.7074	3.5417	0.8724	0.5113	0.0276	0.0886	8.6772	0.4791	1.3603	0.0424	2.2139	0.0626	0.5818	10.8778	1.1169	0.4506	0.8047
TARGET-10-PARMSP-09	13.4926	2.4148	0.9403	0.7915	0.0072	0.6754	15.7461	0.1744	1.0074	0.0183	0.6271	1.8258	2.0282	6.8216	0.859	0.4774	0.2216
TARGET-10-PARMXF-03	1.1387	5.7069	1.5842	2.2078	0.0307	0.0897	4.1084	0.6027	0.003	0.0527	1.4799	10.7186	2.8551	4.6123	2.1695	0.7209	0.571
TARGET-10-PARMXF-04	0.0495	11.1801	1.6921	2.48	0	0	4.6187	1.6121	0.0025	0	10.5061	2.4819	0.9545	13.3905	1.8645	0.983	0.5884
TARGET-10-PARNMF-03	21.2168	32.8912	5.6032	2.0546	3.9727	1.3374	9.4245	3.8647	7.8448	0.2491	11.356	5.2491	6.3119	18.7885	6.9215	3.9644	7.9218
TARGET-10-PARNMF-04	12.9644	5.68	3.5076	0.9009	2.7838	2.6912	6.2373	0.6788	1.7152	0	2.7143	2.6028	2.8242	10.7504	1.5192	0.5957	0.9588
TARGET-10-PARNSH-03	11.9612	19.3803	1.7972	1.1095	0	2.17	8.6583	1.4387	2.4319	0.1345	11.1542	2.2414	3.1622	11.2296	3.7444	1.7247	4.8443
TARGET-10-PARPNM-09	1.1626	0.3048	1.3412	0.4799	0.005	0	6.5542	1.0164	0	0	1.0869	0.4338	0.2597	7.2261	1.481	0.3858	0.8673
TARGET-10-PARPRW-04	13.3168	1.8349	5.5414	0.6067	0	0.0746	4.4356	0.8039	0.5746	0	0.7654	0.0924	0.7674	5.6072	0.9284	0.2418	1.1265
TARGET-10-PARPRW-09	3.2502	3.659	1.5868	0.5417	0	0.717	4.77	0.5668	0.1515	0.0134	1.98	0.4421	0.6241	6.7482	0.9938	0.3668	1.7904
TARGET-10-PARPZJ-03	11.3428	13.782	2.7967	2.7747	1.0568	1.1576	9.8224	1.1884	1.5885	0.25	4.8414	8.2122	12.3581	11.8183	4.7082	0.9833	2.9836
TARGET-10-PARSGC-03	9.9551	9.9164	4.023	1.4976	0.0824	0.8347	13.3217	1.0687	3.5194	0.4483	6.1506	3.2885	7.5661	2.3101	2.1083	0.5104	0.8275
TARGET-10-PARSZH-09	25.1984	3.1212	1.0112	0.3244	0	2.4402	4.816	0.6277	1.5913	0.0209	1.3724	0.0622	1.3564	4.2905	0.7882	0.3336	0.673
TARGET-10-PARTJL-09	18.9733	23.2651	3.7578	3.5014	0.2701	2.7057	12.8417	1.1954	4.8012	0	4.4867	6.0577	0.9333	12.2327	6.102	1.8617	2.4958
TARGET-10-PARTKL-09	44.4874	8.2896	1.3777	0.5547	0	0.6705	4.7234	0.4046	0.9619	0	0.5687	0.3643	1.8133	13.3898	0.3903	0.1871	0.7433
TARGET-10-PARUBK-03	0.0332	1.7762	2.7631	1.6859	0	0.0602	11.4292	0.8862	0.2919	0	4.6427	1.5632	0.9994	9.6466	2.4578	0.8428	2.3406
TARGET-10-PARUGP-09	15.4504	2.1226	1.5286	0.6613	0.0088	0	5.0548	0.6483	0.0618	0	1.8896	0.8428	1.0712	7.0476	0.7332	0.3027	1.7121
TARGET-10-PARVWD-09	7.1655	3.9775	2.5686	0.7504	0.0109	0.0059	3.909	0.6074	5.4308	0.013	1.7779	3.3512	3.1017	9.5176	1.1025	0.4283	0.6837
TARGET-10-PARWVN-03	8.0127	20.0872	3.3815	2.1281	0.1886	4.0848	1.2421	1.8965	0.4727	0	4.4835	3.3852	2.9759	18.3519	2.116	1.2653	1.1917
TARGET-10-PARXMC-04	37.4131	15.1502	4.6327	1.6231	1.6457	1.8448	6.3356	1.7511	4.9351	0.1435	2.4691	4.173	9.7422	9.3149	3.0898	1.6975	4.4016
TARGET-10-PARXMC-09	27.0664	13.0723	2.768	1.4058	0.6141	0.6205	5.6911	1.3118	5.6539	0.0294	2.6361	2.9147	2.1954	5.4927	1.6355	0.8691	3.9941
TARGET-10-PASCIU-03	1.8764	4.1756	0.7824	1.789	0.0115	0.0383	2.3029	0.8101	0.828	0.0487	1.9155	1.0256	0.2889	6.1069	2.2155	0.7405	0.4365
TARGET-10-PASDYK-03	16.111	25.2466	4.4503	1.9704	0.1718	3.9586	19.9864	1.6491	3.3525	0.2165	3.7334	3.3626	6.1495	7.86	2.5814	1.7068	1.4283
TARGET-10-PASEVJ-09	5.4704	18.5288	5.6152	1.3232	0.0194	0.7224	13.3287	2.9232	25.9992	0.0489	4.1701	3.0804	4.2503	12.6799	7.1584	3.5284	5.4106
TARGET-10-PASFXA-03	2.1803	2.9986	0.6011	0.6962	0.0042	0.0239	4.7286	1.0385	0.0046	0	0.9016	0.4758	0	6.8259	1.3785	0.3394	1.0365
TARGET-10-PASFXA-04	18.4973	3.2918	0.6463	0.7543	0.0475	0.0062	4.7361	0.7228	0.0016	0	0.8481	0.1586	0.2399	5.7225	1.1314	0.4292	1.2767
TARGET-10-PASHUI-04	17.9303	2.8577	3.3046	2.5392	0.256	5.4359	10.4894	0.8946	0.9386	0.0266	2.2578	1.6102	3.2822	9.9046	5.7567	2.5814	0.9454
TARGET-10-PASHUI-09	51.8768	23.5204	5.0971	1.6472	0.1318	7.0984	16.9404	2.1983	6.8302	0.052	6.8388	1.1621	1.885	24.1559	6.7012	4.2039	7.0043
TARGET-10-PASKAY-04	14.3327	2.9512	0.6023	0.4622	0.0152	0	2.0848	0.7941	0	0.0388	0.776	0.0587	0.8326	7.5059	0.9718	0.3331	0.8198
TARGET-10-PASKAY-09	21.2472	6.1349	1.984	1.1508	0.021	0.04	4.1603	1.9715	0.0069	0.0322	2.128	0.4595	1.1135	10.3053	2.8995	0.6774	1.1252
TARGET-10-PASKHT-03	0.9031	16.7762	4.1502	3.221	0.2219	0.2413	1.1824	1.803	0.0917	0.2318	8.414	6.0721	19.3397	11.5204	6.3472	1.7947	2.8645
TARGET-10-PASLZM-40	1.0469	4.0536	2.2565	0.5892	0.0415	0.0529	4.9839	0.9786	0.1886	0	1.6579	0.9705	0.4938	4.6461	1.2574	0.3195	0.7858
TARGET-10-PASSXJ-03	10.8684	5.6183	0.2158	0.1423	0.0794	0.0002	2.6778	0.2009	0.1052	0	0.2513	0	1.0206	9.7165	0.9483	0.0872	0.295
TARGET-10-PASUBW-09	0.4264	2.3648	0.2015	0.1561	0	0.0501	3.6016	0.4966	0.0086	0	0.2748	0.6259	0.1044	4.4436	0.758	0.0076	0.3722
TARGET-10-PASYGM-03	1.5948	3.6843	1.6763	1.5615	0.1665	0.2547	1.468	0.797	0.121	0.1222	4.4934	4.2113	2.71	12.7744	3.4027	0.9249	1.159
TARGET-10-PATCTI-04	31.1409	2.1326	0.8484	0.0657	0.1795	0	1.9208	0.7955	0	0	0.3922	0	0.2615	5.6355	0.551	0.1225	0.5266
TARGET-10-PATCTI-09	6.5336	7.4305	2.1927	1.1118	0.0412	0.1319	1.0727	1.0243	0.0163	0	2.8953	6.3315	1.2868	9.0859	2.2671	0.8133	1.441
TARGET-10-PATTEE-03	0.832	0.173	0.2278	0.3325	0	0.0141	15.6873	0.1972	0.0036	0	0.3739	0	0.0807	5.5347	0.83	0.2402	0.1167

Table S12. Chromosome distribution of 1057 genes upregulated in younger pediatric ALL patients that are also correlated with high survival

Gene	Chromosome
CCNY	10p11.21
CCDC7	10p11.22
SVIL	10p11.23
C10orf126	10p12.1
ARMC3	10p12.2
OTUD1	10p12.2
C10orf111	10p13
PPIAP30	10p13
SFMBT2	10p14
LINC00700	10p15.3
OR13A1	10q11.21
C10orf53	10q11.23
ERCC6	10q11.23
IPMK	10q21.1
ARID5B	10q21.2
REEP3	10q21.3
SNORD98	10q21.3
TET1	10q21.3
DUPD1	10q22.2
KAT6B	10q22.2
C10orf99	10q23.1
CCSER2	10q23.1
LINC00858	10q23.1
RGR	10q23.1
NUTM2A	10q23.2
LINC00865	10q23.31
SNORA12	10q24.31
BTRC	10q24.32
FBXW4	10q24.32
SUFU	10q24.32
CALHM1	10q24.33
GUCY2GP	10q25.2
TCF7L2	10q25.2-q25.3
CCDC186	10q25.3
MIR3663HG	10q25.3
PNLIPRP1	10q25.3
PDZD8	10q25.3-q26.11
CACUL1	10q26.11
GRK5-IT1	10q26.11
SNORA19	10q26.11
IKZF5	10q26.13
PLEKHA1	10q26.13
ZRANB1	10q26.13
PTPRE	10q26.2
JAKMIP3	10q26.3
ALX4	11p11.2

BBOX1-AS1	11p14.2-p14.1
MRGPRX1	11p15.1
PTPN5	11p15.1
TPH1	11p15.1
PDE3B	11p15.2
ART1	11p15.4
CYB5R2	11p15.4
DCHS1	11p15.4
KCNQ1DN	11p15.4
MRVI1	11p15.4
MRVI1-AS1	11p15.4
NLRP10	11p15.4
OR52H1	11p15.4
OR5P2	11p15.4
OR5P3	11p15.4
OVCH2	11p15.4
TRPM5	11p15.5
OR5A2	11q12.1
TRIM51	11q12.1
MS4A8	11q12.2
ANKRD13D	11q13.2
FOLR2	11q13.4
KCTD21	11q14.1
TRIM49C	11q14.3
MAML2	11q21
USP28	11q23.2
TMPRSS13	11q23.3
TMPRSS4-AS1	11q23.3
CLMP	11q24.1
CDON	11q24.2
HEPACAM	11q24.2
OR10G8	11q24.2
LINC00167	11q24.3
PRDM10	11q24.3
BICD1	12p11.21
H3F3C	12p11.21
SLCO1C1	12p12.2
ERP27	12p12.3
RERGL	12p12.3
TAS2R13	12p13
LRP6	12p13.2
TAS2R31	12p13.2
AICDA	12p13.31
ATN1	12p13.31
CLEC2A	12p13.31
CLEC4E	12p13.31
FAM66C	12p13.31

NANOG	12p13.31
PHC1	12p13.31
SCARNA11	12p13.31
CNTN1	12q12
SNORA2A	12q13.11
SNORA2B	12q13.11
MIR1293	12q13.12
GRASP	12q13.13
KRT2	12q13.13
POU6F1	12q13.13
LACRT	12q13.2
OR6C2	12q13.2
IL23A	12q13.3
R3HDM2	12q13.3
MON2	12q14.1
KCNMB4	12q15
MDM2	12q15
SLC35E3	12q15
RAB21	12q21.1
VEZT	12q22
DEPDC4	12q23.1
ELK3	12q23.1
RMST	12q23.1
APPL2	12q23.3
CRY1	12q23.3
EID3	12q23.3
GLT8D2	12q23.3
NT5DC3	12q23.3
RFX4	12q23.3
LINC01486	12q24.11
SSH1	12q24.11
TBX3	12q24.21
HRK	12q24.22
SRRM4	12q24.23
HCAR3	12q24.31
RILPL1	12q24.31
SIRT4	12q24.31
LINC00939	12q24.32
PIWIL1	12q24.33
ZNF891	12q24.33
LINC00544	13q12.3
MTUS2	13q12.3
RNU6-53P	13q12.3
TEX26-AS1	13q12.3
UBL3	13q12.3
FOXO1	13q14.11
LINC01055	13q14.13

RNF219-AS1	13q22.3-q31.1
POU4F1	13q31.1
SLITRK5	13q31.2
LINC00559	13q31.3
MIR3170	13q32.2
SLC15A1	13q32.2-q32.3
DAOA	13q33.2
LINC00676	13q34
OR4Q3	14p13
ACIN1	14q11.2
CEBPE	14q11.2
IL25	14q11.2
OR4K1	14q11.2
OR4K14	14q11.2
OR4K2	14q11.2
OR4M1	14q11.2
OR4N2	14q11.2
OSGEP	14q11.2
POTEM	14q11.2
RAB2B	14q11.2
RPGRIP1	14q11.2
LINC01296	14q11.2
HECTD1	14q12
NOVA1	14q12
STRN3	14q12
C14orf23	14q12
BAZ1A	14q13.1-q13.2
BRMS1L	14q13.2
MIA2	14q13.2
PPP2R3C	14q13.2
RALGAPA1	14q13.2
MBIP	14q13.3
LRFN5	14q21.1
C14orf28	14q21.2
FANCM	14q21.2
FAM179B	14q21.2
SOS2	14q21.3
C14orf182	14q21.3
FRMD6-AS2	14q22.1
GPR137C	14q22.1
PELI2	14q22.3
C14orf105	14q22.3
SLC35F4	14q22.3-q23.1
PRKCH	14q23.1
WDR89	14q23.2
FNTB	14q23.3
FUT8	14q23.3

MIR4706	14q23.3
MPP5	14q23.3
PLEKHD1	14q24.1
RBM25	14q24.2
PCNX	14q24.2
ADCK1	14q24.3
C14orf178	14q24.3
NUMB	14q24.3
ZDHHC22	14q24.3
FOXN3	14q31.3-q32.11
FOXN3-AS2	14q32.11
RPS6KA5	14q32.11
ATXN3	14q32.12
BTBD7	14q32.12
COX8C	14q32.12
TRIP11	14q32.12
UNC79	14q32.12
SMEK1	14q32.12
BDKRB1	14q32.2
CYP46A1	14q32.2
EVL	14q32.2
PAPOLA	14q32.2
SETD3	14q32.2
DIO3	14q32.31
DIO3OS	14q32.31
MEG8	14q32.31
MIR4710	14q32.33
PPP1R13B	14q32.33
SNORD108	15q11.2
SNORD116-23	15q11.2
SNORD116-24	15q11.2
SNORD116-29	15q11.2
GOLGA8M	15q13.1
NUTM1	15q14
MGA	15q15
INO80	15q15.1
PLA2G4E	15q15.1
PPP1R14D	15q15.1
DUOX1	15q21.1
LINC01413	15q21.3
RNF111	15q22.1-q22.2
CILP	15q22.31
DAPK2	15q22.31
LCTL	15q22.31
LINC01169	15q22.31
SCARNA14	15q22.31
ITGA11	15q23

MIR629	15q23
PAQR5	15q23
LINC00277	15q23
MIR4513	15q24.1
PEAK1	15q24.3
SCAPER	15q24.3
ADAMTSL3	15q25.2
ASB9P1	15q26.1
FAM174B	15q26.1
LINC00930	15q26.1
FAM169B	15q26.3
FBXL19	16p11.2
GDPD3	16p11.2
NPIPB9	16p11.2
ZG16	16p11.2
ZNF843	16p11.2
CACNG3	16p12.1
IL21R-AS1	16p12.1
KIAA0430	16p13.11
C16orf72	16p13.2
C16orf96	16p13.3
LUC7L	16p13.3
MEIOB	16p13.3
OR1F2P	16p13.3
OR2C1	16p13.3
PTX4	16p13.3
RGS11	16p13.3
RPL3L	16p13.3
CASC16	16q12.1-q12.2
FTO-IT1	16q12.2
NLRC5	16q13
ESRP2	16q22.1
TERF2	16q22.1
TMED6	16q22.1
ZFP90	16q22.1
PHLPP2	16q22.2
PKD1L2	16q23.2
HSD17B2	16q23.3
CBFA2T3	16q24.3
AKAP10	17p11.2
CCDC144NL	17p11.2
EPN2-AS1	17p11.2
GRAPL	17p11.2
PIGL	17p11.2
RNF112	17p11.2
ZNF624	17p11.2
LINC00670	17p12

MYOCD	17p12
NLRP1	17p13
FBXO39	17p13.1
KCNAB3	17p13.1
KRBA2	17p13.1
MIR497HG	17p13.1
SLC16A11	17p13.1
SLC16A13	17p13.1
TMEM88	17p13.1
ANKFY1	17p13.2
ARRB2	17p13.2
CAMTA2	17p13.2
MIS12	17p13.2
SLC52A1	17p13.2
ZZEF1	17p13.2
OR1G1	17p13.3
SGSM2	17p13.3
SMG6	17p13.3
FAM101B	17p13.3
SNORD4B	17q11
WSB1	17q11.1
EFCAB5	17q11.2
FAM222B	17q11.2
NOS2	17q11.2
SH3GL1P1	17q11.2
SNORD42B	17q11.2
SSH2	17q11.2
TAOK1	17q11.2
TBC1D29	17q11.2
FBXL20	17q12
LRRC37A11P	17q12
LRRC3C	17q21.1
MSL1	17q21.1
CCR10	17q21.2
EIF1	17q21.2
KRTAP16-1	17q21.2
KRTAP17-1	17q21.2
KRTAP29-1	17q21.2
KRTAP4-1	17q21.2
KRTAP4-11	17q21.2
KRTAP4-12	17q21.2
LINC00974	17q21.2
TUBG2	17q21.2
GPATCH8	17q21.31
KANSL1	17q21.31
LRRC37A	17q21.31
LRRC37A2	17q21.31

C17orf105	17q21.31
LINC00854	17q21.31
IGF2BP1	17q21.32
ZNF652	17q21.32
APPBP2	17q23.2
MED13	17q23.2
KCNH6	17q23.3
MAP3K3	17q23.3
SNORA38B	17q24.2
ACOX1	17q25.1
BTBD17	17q25.1
CPSF4L	17q25.1
GPR142	17q25.1
ITGB4	17q25.1
MYO15B	17q25.1
SAP30BP	17q25.1
RNF213	17q25.3
SGSH	17q25.3
SLC26A11	17q25.3
TNRC6C-AS1	17q25.3
ENTHD2	17q25.3
ANKRD62	18p11.21
CEP192	18p11.21
GNAL	18p11.21
LINC01255	18p11.21
LINC01254	18p11.22
NDUFV2-AS1	18p11.22
DLGAP1	18p11.31
LAMA1	18p11.31
LINC00667	18p11.31
LINC00668	18p11.31
CLUL1	18p11.32
MYO5B	18q
ABHD3	18q11.2
AQP4-AS1	18q11.2
MIB1	18q11.2
ASXL3	18q12.1
B4GALT6	18q12.1
DSC3	18q12.1
DSG1-AS1	18q12.1
TRAPPC8	18q12.1
TTR	18q12.1
KIAA1328	18q12.2
ZNF397	18q12.2
LINC01478	18q12.3
PIK3C3	18q12.3
SETBP1	18q12.3

CTIF	18q21.1
KATNAL2	18q21.1
LOXHD1	18q21.1
MIR4744	18q21.1
SIGLEC15	18q21.1
DCC	18q21.2
MEX3C	18q21.2
SMAD4	18q21.2
SNORA37	18q21.2
TCF4	18q21.2
CDH20	18q21.33
C18orf63	18q22.3
FAM69C	18q22.3
ZNF236	18q22-q23
ZNF407	18q23
ZNF430	19p12
ZNF737	19p12
OR10H2	19p13.1
ARRDC2	19p13.11
INSL3	19p13.11
LINC00663	19p13.11
ZNF14	19p13.11
KIAA1683	19p13.11
PGLYRP2	19p13.12
AP1M2	19p13.2
KANK3	19p13.2
MIR4748	19p13.2
SNORD12B	19p13.2
ZNF491	19p13.2
ZNF699	19p13.2
C19orf71	19p13.3
MIR4747	19p13.3
HPN	19q13.11
PRODH2	19q13.12
ZNF570	19q13.12
ZNF571-AS1	19q13.12
ZNF793	19q13.12
ZNF540	19q13.13
LGALS7B	19q13.2
NTF4	19q13.3
TRPM4	19q13.3
ZNF283	19q13.31
CEACAM16	19q13.31-q13.32
ADM5	19q13.33
BSPH1	19q13.33
DKKL1	19q13.33
FAM83E	19q13.33

MIR4324	19q13.33
RCN3	19q13.33
SLC6A16	19q13.33
TULP2	19q13.33
LILRA2	19q13.4
ZNF460	19q13.4
FAM90A27P	19q13.42
NLRP9	19q13.43
ZNF471	19q13.43
C1orf137	1p13.1
KCNA2	1p13.3
OLFM3	1p21.1
NEGR1-IT1	1p31.1
FAM73A	1p31.1
NFIA-AS2	1p31.3
SGIP1	1p31.3
MIR5095	1p32.3
SLC5A9	1p33
ELAVL4	1p33-p32.3
CCDC17	1p34.1
ERI3-IT1	1p34.1
AGO4	1p34.3
CSMD2	1p35.1
HCRTR1	1p35.2
SCARNA1	1p35.3
CD164L2	1p36.11
FUCA1	1p36.11
SFN	1p36.11
WNT4	1p36.12
AADACL4	1p36.21
SLC25A34	1p36.21
C1orf127	1p36.22
MIR4689	1p36.31
MEGF6	1p36.32
DRD5P2	1q21.1
RNVU1-17	1q21.2
ATP8B2	1q21.3
DCST2	1q21.3
SLC27A3	1q21.3
SNORA80E	1q22
SPTA1	1q23.1
ATP1A4	1q23.2
CADM3	1q23.2
IVNS1ABP	1q25.3
OVAAL	1q25.3
RGL1	1q25.3
SHCBP1L	1q25.3

OCLM	1q31.1
MIR1278	1q31.2
CRB1	1q31.3
CHIT1	1q32.1
DYRK3	1q32.1
LAX1	1q32.1
LRRN2	1q32.1
NR5A2	1q32.1
ZBED6	1q32.1
FAIM3	1q32.1
HSD11B1	1q32.2
KCNH1	1q32.2
SERTAD4-AS1	1q32.2
GJC2	1q42.13
OBSCN	1q42.13
SIPA1L2	1q42.2
BANF2	20p12.1
FLRT3	20p12.1
KIF16B	20p12.1
SLX4IP	20p12.2
BMP2	20p12.3
HAO1	20p12.3
EBF4	20p13
SUN5	20q11.21
MIR4755	20q11.22
PHF20	20q11.22-q11.23
GHRH	20q11.23
PLCG1-AS1	20q12
RBPJL	20q13.12
SULF2	20q13.12
PREX1	20q13.13
SNORD12	20q13.13
SNORD12C	20q13.13
CTCFL	20q13.31
ANKRD60	20q13.32
HSPA13	21q11.2
SAMSN1-AS1	21q11.2
NRIP1	21q11.2-q21.1
LINC00320	21q21.1
NCAM2	21q21.1
USP25	21q21.1
BACH1	21q21.3
GABPA	21q21.3
GRIK1	21q21.3
LTN1	21q21.3
ITSN1	21q22.11
LINC00159	21q22.11

LINC00649	21q22.11
PAXBP1	21q22.11
PAXBP1-AS1	21q22.11
SCAF4	21q22.11
SON	21q22.11
SYNJ1	21q22.11
CBR3-AS1	21q22.12
SETD4	21q22.12
DOPEY2	21q22.12
CLDN14	21q22.13
DSCR9	21q22.13
DSCAM-IT1	21q22.2
ERG	21q22.2
ABCG1	21q22.3
C21orf2	21q22.3
DIP2A	21q22.3
DIP2A-IT1	21q22.3
ITGB2-AS1	21q22.3
LINC00322	21q22.3
MCM3AP	21q22.3
PCNT	21q22.3
ZBTB21	21q22.3
CCT8L2	22q11.1
IL17RA	22q11.1
ZNF280A	22q11.22
TNRC6B	22q13.1
TSPYL6	2p16.2
SLC3A1	2p21
CDKL4	2p22.1
ATL2	2p22.2-p22.1
MYADML	2p22.3
XDH	2p23.1
YPEL5	2p23.1
NCOA1	2p23.3
TCF23	2p23.3
MYCNOS	2p24.3
MIR7515HG	2p25.2
ZNF514	2q11.1
CNGA3	2q11.2
MAP4K4	2q11.2
VWA3B	2q11.2
SLC5A7	2q12.3
EDAR	2q13
CBWD2	2q14.1
DPP10-AS1	2q14.1
FOXD4L1	2q14.1
HTR5BP	2q14.1

ZC3H6	2q14.1
INHBB	2q14.2
MIR128-1	2q21.3
RAB3GAP1	2q21.3
ACVR1C	2q24.1
ABCB11	2q31.1
DHRS9	2q31.1
TLK1	2q31.1
MLK7-AS1	2q31.1
PLEKHA3	2q31.2
ALS2CR11	2q33.1
CYP20A1	2q33.2
FAM117B	2q33.2
LINC00607	2q35
MIR26B	2q35
TMEM169	2q35
MIR4439	2q36.2
C2orf83	2q36.3
SLC16A14	2q36.3
CHRNG	2q37.1
UGT1A9	2q37.1
ACKR3	2q37.3
FOXP1	3p13
FRMD4B	3p14.1
LMOD3	3p14.1
CADPS	3p14.2
LINC00698	3p14.2
SYNPR	3p14.2
ERC2	3p14.3
FAM208A	3p14.3
GRM2	3p21.2
CDHR4	3p21.31
MIR566	3p21.31
SEMA3F	3p21.31
SLC6A20	3p21.31
SNRK	3p22.1
VIPR1	3p22.1
TTC21A	3p22.2
LRRC3B	3p24.1
RBMS3-AS3	3p24.1
LINC00852	3p25.3
SLC6A11	3p25.3
EDEM1	3p26.1
ITPR1	3p26.1
CNTN4	3p26.3-p26.2
RNU6-26P	3q12.1
BBX	3q13.12

TMPRSS7	3q13.2
KIAA2018	3q13.2
FBXO40	3q13.33
POPDC2	3q13.33
RYK	3q22.2
A4GNT	3q22.3
ZIC1	3q24
CLRN1	3q25.1
TSC22D2	3q25.1
WWTR1-AS1	3q25.1
C3orf55	3q25.32
HTR3D	3q27.1
C3orf65	3q27.2
FETUB	3q27.3
KNG1	3q27.3
UTS2B	3q28
SLC51A	3q29
CWH43	4p11
SLAIN2	4p11
ZAR1	4p11
GABRA4	4p12
GABRB1	4p12
NSUN7	4p14
TLR1	4p14
GBA3	4p15.2
PI4K2B	4p15.2
RBPJ	4p15.2
CLNK	4p16.1
KIAA0232	4p16.1
STK32B	4p16.2
C4orf6	4p16.2
FAM193A	4p16.3
GAK	4p16.3
PCGF3	4p16.3
CLOCK	4q12
SNORA26	4q12
TMPRSS11E	4q13.2
AFM	4q13.3
SMR3B	4q13.3
PPEF2	4q21.1
SOWAHB	4q21.1
C4orf26	4q21.1
BMP2K	4q21.21
BMP3	4q21.21
HNRNPDL	4q21.22
CDS1	4q21.23
NKX6-1	4q21.23

WDFY3	4q21.23
WDFY3-AS2	4q21.23
MAPK10	4q21.3
ABCG2	4q22.1
CCSER1	4q22.1
HERC3	4q22.1
PKD2	4q22.1
PPM1K	4q22.1
ADH4	4q23
ADH7	4q23
MIR3684	4q23
MTTP	4q23
ARHGEF38	4q24
DDIT4L	4q24
LINC01216	4q24
TET2	4q24
DKK2	4q25
ZGRF1	4q25
CEP170P1	4q26
USP53	4q26
ANKRD50	4q28.1
LINC01091	4q28.1
LARP1B	4q28.2
ELMOD2	4q31.1
MAML3	4q31.1
MGARP	4q31.1
RAB33B	4q31.1
SCOC-AS1	4q31.1
SMAD1	4q31.21
TBC1D9	4q31.21
ARFIP1	4q31.3
FGA	4q31.3
TIGD4	4q31.3
ETFDH	4q32.1
LRAT	4q32.1
RAPGEF2	4q32.1
FSTL5	4q32.2
NAF1	4q32.2
TKTL2	4q32.2
DDX60L	4q32.3
HAND2-AS1	4q34.1
SPATA4	4q34.2
ENPP6	4q35.1
FAM92A1P2	4q35.1
TRAPPC11	4q35.1
LINC01262	4q35.2
ZFP42	4q35.2

FGF10-AS1	5p12
C9	5p13.1
DAB2	5p13.1
FYB	5p13.1
FAM134B	5p15.1
IRX1	5p15.33
ZNF366	5q13.1
SCARNA18	5q14.2
MEF2C	5q14.3
LINC01340	5q15
POU5F2	5q15
KCNN2	5q22.3
ADAMTS19	5q23.3
SLC12A2	5q23.3
SLC27A6	5q23.3
RAPGEF6	5q31.1
ARHGAP26-IT1	5q31.3
PCDHB10	5q31.3
PCDHB11	5q31.3
PCDHB14	5q31.3
PCDHB15	5q31.3
PCDHB19P	5q31.3
C5orf46	5q32
PDE6A	5q32
NMUR2	5q33.1
PANK3	5q34
FOXI1	5q35.1
SNORA74B	5q35.1
FAM193B	5q35.3
MIR340	5q35.3
PROP1	5q35.3
ZNF454	5q35.3
MIR548U	6p11.2
GSTA4	6p12.2
IL17F	6p12.2
CD2AP	6p12.3
TFAP2D	6p12.3
GPR111	6p12.3
GPR115	6p12.3
GNMT	6p21.1
KLC4	6p21.1
NCR2	6p21.1
OARD1	6p21.1
PRPH2	6p21.1
SLC22A7	6p21.1
TREML2	6p21.1
TREML4	6p21.1

UNC5CL	6p21.1
ZNF318	6p21.1
LAP3P2	6p21.2
TBC1D22B	6p21.2
C6orf222	6p21.31
CLPSL1	6p21.31
DEF6	6p21.31
IP6K3	6p21.31
STK38	6p21.31
AGER	6p21.32
BRD2	6p21.32
C6orf10	6p21.32
PBX2	6p21.32
RXRB	6p21.32
DDX39B	6p21.33
GNL1	6p21.33
HLA-C	6p21.33
IER3	6p21.33
SNORA38	6p21.33
SNORD117	6p21.33
SNORD84	6p21.33
GABBR1	6p22.1
TRIM39	6p22.1
BTN2A1	6p22.2
HIST1H1A	6p22.2
HIST1H2BC	6p22.2
SCGN	6p22.2
MIR4639	6p22.3
NRSN1	6p22.3
STMND1	6p22.3
HIVEP1	6p24.1
CDYL	6p25.1
RIPK1	6p25.2
EYS	6q12
COL19A1	6q13
FAM135A	6q13
RIMS1	6q13
COL12A1	6q13-q14.1
FILIP1	6q14.1
PHIP	6q14.1
SENP6	6q14.1
RARS2	6q15
SLC35A1	6q15
C6orf164	6q15-q16.1
EPHA7	6q16.1
FAXC	6q16.2
ASCC3	6q16.3

HACE1	6q16.3
MICAL1	6q21
TRAF3IP2-AS1	6q21
ZBTB24	6q21
FRK	6q22.1
NT5DC1	6q22.1
ROS1	6q22.1
RSPH4A	6q22.1
TSPYL1	6q22.1
MCM9	6q22.31
C6orf58	6q22.33
LINC01013	6q23.2
SNORA33	6q23.2
SNORD100	6q23.2
SNORD101	6q23.2
PBOV1	6q23.3
PDE7B	6q23.3
KIAA1244	6q23.3-q24.1
HECA	6q24.1
NMBR	6q24.1
EPM2A	6q24.3
GRM1	6q24.3
ZC3H12D	6q25.1
IPCEF1	6q25.2
SCAF8	6q25.2
ARID1B	6q25.3
CLDN20	6q25.3
SOD2	6q25.3
SYNJ2-IT1	6q25.3
TAGAP	6q25.3
TATDN2P2	6q25.3
PHF10	6q27
T	6q27
FKBP9P1	7p11.2
ABCA13	7p12.3
SEPT7P2	7p12.3
STK17A	7p13
HERPUD2	7p14.2
DPY19L1P1	7p14.3
KBTBD2	7p14.3
NEUROD6	7p14.3
RP9	7p14.3
WIPF3	7p14.3
CCDC129	7p14.3
IGF2BP3	7p15.3
COL28A1	7p21.3
DAGLB	7p22.1

RNF216-IT1	7p22.1
RSPH10B	7p22.1
C1GALT1	7p22.1-p21.3
CCT6P3	7q11.21
ERV3-1	7q11.21
ZNF117	7q11.21
RSBN1L	7q11.23
SPDYE5	7q11.23
UPK3B	7q11.23
PCLO	7q21.11
SEMA3D	7q21.11
GRM3	7q21.11-q21.12
CYP51A1-AS1	7q21.2
GNG11	7q21.3
SGCE	7q21.3
AZGP1P1	7q22.1
EPHB4	7q22.1
GPC2	7q22.1
LRRC17	7q22.1
MIR4653	7q22.1
NYAP1	7q22.1
GATS	7q22.1
GPR22	7q22.3
LAMB1	7q31.1
LAMB4	7q31.1
C7orf60	7q31.1
ASZ1	7q31.2
CFTR	7q31.2
KCND2	7q31.31
HYAL4	7q31.32
FAM71F1	7q32.1
OR6W1P	7q34
PRSS1	7q34
WEE2	7q34
WEE2-AS1	7q34
C7orf34	7q34
OR2A12	7q35
OR2A20P	7q35
SHH	7q36.3
KAT6A	8p11.21
AGPAT6	8p11.21
C8orf4	8p11.21
HGSNAT	8p11.21-p11.1
CSGALNACT1	8p21.3
ENTPD4	8p21.3
FAM160B2	8p21.3
PEBP4	8p21.3

PIWIL2	8p21.3
PPP3CC	8p21.3
SORBS3	8p21.3
PSD3	8p22
SGCZ	8p22
TUSC3	8p22
FAM90A2P	8p23.1
ARHGEF10	8p23.3
LINC01301	8q12.1
PLAG1	8q12.1
TOX	8q12.1
PDE7A	8q13.1
NCOA2	8q13.3
KCNB2	8q21.11
PAG1	8q21.13
ZBTB10	8q21.13
ZNF704	8q21.13
CNGB3	8q21.3
PSKH2	8q21.3
WWP1	8q21.3
MIR1273A	8q22.2
TBC1D31	8q24.13
CASC8	8q24.21
FAM84B	8q24.21
PCAT1	8q24.21
ASAP1	8q24.21-q24.22
MROH5	8q24.3
PLEC	8q24.3
PTK2	8q24.3
SPATC1	8q24.3
FAM221B	9p13.3
GNE	9p13.3
RECK	9p13.3
TUSC1	9p21.2
TTC39B	9p22.3
KIAA2026	9p24.1
LINC01410	9q13
MIR4477B	9q13
CBWD3	9q21.11
ABHD17B	9q21.13
GNA14	9q21.2
KIF27	9q21.32
SPIN1	9q22.1
DIRAS2	9q22.2
TGFBR1	9q22.33
OR1L1	9q33.2
COL5A1	9q34.3

FAAH2	Xp11.21
RRAGB	Xp11.21
UBQLN2	Xp11.21
ZXDB	Xp11.21
ACTG1P10	Xp11.22
FGD1	Xp11.22
HUWE1	Xp11.22
KANTR	Xp11.22
KDM5C	Xp11.22
PHF8	Xp11.22
SSX7	Xp11.22
WNK3	Xp11.22
CCDC120	Xp11.23
GRIPAP1	Xp11.23
HDAC6	Xp11.23
KCND1	Xp11.23
OTUD5	Xp11.23
PORCN	Xp11.23
SLC35A2	Xp11.23
SYP	Xp11.23
TBC1D25	Xp11.23
TFE3	Xp11.23
ZNF182	Xp11.23
ZNF630	Xp11.23
ZNF81	Xp11.23
INE1	Xp11.3
KDM6A	Xp11.3
RGN	Xp11.3
SLC9A7	Xp11.3
UBA1	Xp11.3
ZNF674	Xp11.3
CASK	Xp11.4
CXorf38	Xp11.4
DDX3X	Xp11.4
RPGR	Xp11.4
USP9X	Xp11.4
CXorf22	Xp21.1
CXorf30	Xp21.1
TAB3	Xp21.2
SMEK3P	Xp21.3
ACOT9	Xp22.11
CDKL5	Xp22.13
SCML1	Xp22.13
SCML2	Xp22.13
TRAPPC2	Xp22.2
FAM9B	Xp22.31
PRKX-AS1	Xp22.33

LINC00102	Xp22.33 and Yp11.31
AWAT2	Xq13.1
CXorf65	Xq13.1
DLG3	Xq13.1
EFNB1	Xq13.1
FOXO4	Xq13.1
ITGB1BP2	Xq13.1
MED12	Xq13.1
NONO	Xq13.1
P2RY4	Xq13.1
TAF1	Xq13.1
RGAG4	Xq13.1
CHIC1	Xq13.2
FTX	Xq13.2
MIR374B	Xq13.2
MIR421	Xq13.2
ABCB7	Xq13.3
ATRX	Xq21.1
BRWD3	Xq21.1
HDX	Xq21.1
MAGT1	Xq21.1
RPS6KA6	Xq21.1
CHM	Xq21.2
TGIF2LX	Xq21.31
DIAPH2-AS1	Xq21.33
ARMCX4	Xq22.1
ARMCX5	Xq22.1
GPRASP1	Xq22.1
HNRNPH2	Xq22.1
LINC00630	Xq22.1
RAB40AL	Xq22.1
ZMAT1	Xq22.1
TCEAL3	Xq22.2
CLDN2	Xq22.3
RBM41	Xq22.3
RIPPLY1	Xq22.3
TBC1D8B	Xq22.3
DCX	Xq23
TRPC5	Xq23
CUL4B	Xq24
DOCK11	Xq24
LINC01285	Xq24
NKAP	Xq24
UPF3B	Xq24
WDR44	Xq24
IGSF1	Xq25
TENM1	Xq25

ZIK1P1	Xq25
OCRL	Xq26.1
ZNF280C	Xq26.1
MBNL3	Xq26.2
RAP2C	Xq26.2
RAP2C-AS1	Xq26.2
FAM122B	Xq26.3
MIR503HG	Xq26.3
MMGT1	Xq26.3
SLC9A6	Xq26.3
SNORD61	Xq26.3
ZNF75D	Xq26.3
DDX26B	Xq26.3
ATP11C	Xq27.1
CDR1	Xq27.1
F9	Xq27.1
LINC00632	Xq27.1
MIR505	Xq27.1
SOX3	Xq27.1
SPANXD	Xq27.2
FMR1	Xq27.3
CD99L2	Xq28
GABRE	Xq28
MTMR1	Xq28

Table S13a. Geneontology output for genes upregulated in low surviving, older pediatric NBL patients

Analysis Type:	PANTHER Overrepresentation Test (Released 20181113)
Annotation Version and Release Date:	GO Ontology database Released 2019-01-01
Analyzed List:	upload_1 (Homo sapiens)
Reference List:	Homo sapiens (all genes in database)
Test Type:	FISHER
Correction:	BONFERRONI
Bonferroni count:	8749

GO biological process complete	Homo sapiens - REFLIST (20996)	upload_1 (88)	upload_1 (expected)	upload_1 (over/under)	upload_1 (fold Enrichment)	upload_1 (P-value)
antigen receptor-mediated signaling pathway (GO:0050851)	292	10	1.22	+	8.17	4.62E-03
negative regulation of mitotic cell cycle (GO:0045930)	306	10	1.28	+	7.8	6.97E-03
negative regulation of cell cycle process (GO:0010948)	327	10	1.37	+	7.3	1.25E-02
Unclassified (UNCLASSIFIED)	3224	6	13.51	-	0.44	0.00E+00

Table S13b. Geneontology output for genes upregulated in high surviving, younger pediatric NBL patients

Analysis Type:	PANTHER Overrepresentation Test (Released 20181113)						
Annotation Version and Release Date:	GO Ontology database Released 2019-01-01						
Analyzed List:	upload_1 (Homo sapiens)						
Reference List:	Homo sapiens (all genes in database)						
Test Type:	FISHER						
Correction:	BONFERRONI						
Bonferroni count:		8749					
GO biological process complete	Homo sapiens - REFLIST (20996)		upload_1 (389)	upload_1 (expected)	upload_1 (over/unc	upload_1 (fold Enric	upload_1 (P-value)
neuron projection morphogenesis (GO:00488		475	31	8.8	+	3.52	3.81E-05
axon development (GO:0061564)		401	26	7.43	+	3.5	7.90E-04
plasma membrane bounded cell projection m		479	31	8.87	+	3.49	4.58E-05
regulation of small GTPase mediated signal tr		340	22	6.3	+	3.49	8.11E-03
cell projection morphogenesis (GO:0048858)		481	31	8.91	+	3.48	5.02E-05
cell morphogenesis involved in neuron differe		424	27	7.86	+	3.44	6.27E-04
axonogenesis (GO:0007409)		366	23	6.78	+	3.39	7.37E-03
cell part morphogenesis (GO:0032990)		502	31	9.3	+	3.33	1.28E-04
regulation of neuron projection development		483	28	8.95	+	3.13	2.23E-03
neuron projection development (GO:0031175		659	38	12.21	+	3.11	1.68E-05
regulation of neuron differentiation (GO:0045		643	35	11.91	+	2.94	2.97E-04
cell morphogenesis involved in differentiatior		540	29	10	+	2.9	6.04E-03
neuron development (GO:0048666)		802	42	14.86	+	2.83	3.38E-05
cell morphogenesis (GO:0000902)		696	36	12.9	+	2.79	6.31E-04
neuron differentiation (GO:0030182)		989	48	18.32	+	2.62	2.52E-05
regulation of plasma membrane bounded cell		649	31	12.02	+	2.58	2.58E-02
cellular component morphogenesis (GO:0032		796	38	14.75	+	2.58	1.90E-03
regulation of neurogenesis (GO:0050767)		796	38	14.75	+	2.58	1.90E-03
regulation of nervous system development (G		902	43	16.71	+	2.57	4.67E-04
regulation of cell projection organization (GO		658	31	12.19	+	2.54	3.37E-02
generation of neurons (GO:0048699)		1502	68	27.83	+	2.44	1.74E-07
neurogenesis (GO:0022008)		1600	70	29.64	+	2.36	3.76E-07
regulation of cell development (GO:0060284)		916	39	16.97	+	2.3	2.26E-02
plasma membrane bounded cell projection or		1082	46	20.05	+	2.29	3.70E-03
cell projection organization (GO:0030030)		1122	46	20.79	+	2.21	6.93E-03
nervous system development (GO:0007399)		2309	94	42.78	+	2.2	3.98E-09
cell development (GO:0048468)		1597	59	29.59	+	1.99	5.95E-03
multicellular organism development (GO:000		4940	133	91.53	+	1.45	2.13E-02
anatomical structure development (GO:0048{		5320	143	98.57	+	1.45	8.40E-03
developmental process (GO:0032502)		5664	149	104.94	+	1.42	1.39E-02
multicellular organismal process (GO:003250		6866	173	127.21	+	1.36	1.69E-02
biological_process (GO:0008150)		17772	360	329.27	+	1.09	3.83E-02
Unclassified (UNCLASSIFIED)		3224	29	59.73	-	0.49	0.00E+00

Table S13c. Geneontology output for genes upregulated in low surviving, older pediatric ALL patients

Analysis Type:	PANTHER Overrepresentation Test (Released 20181113)					
Annotation Version and Release Date:	GO Ontology database Released 2019-01-01					
Analyzed List:	upload_1 (Homo sapiens)					
Reference List:	Homo sapiens (all genes in database)					
Test Type:	FISHER					
Correction:	BONFERRONI					
Bonferroni count:	8749					
GO biological process complete	Homo sapiens - REFLIST (20996)	upload_1 (461)	upload_1 (expected)	upload_1 (over/under)	upload_1 (fold Enrichment)	upload_1 (P-value)
mitochondrial translational elongation (GO:0070125)	88	25	1.93	+	12.94	1.25E-14
mitochondrial translational termination (GO:0070126)	89	24	1.95	+	12.28	1.69E-13
translational termination (GO:0006415)	97	24	2.13	+	11.27	8.98E-13
mitochondrial translation (GO:0032543)	112	25	2.46	+	10.17	1.64E-12
translational elongation (GO:0006414)	125	26	2.74	+	9.47	1.80E-12
cellular protein complex disassembly (GO:0043624)	138	27	3.03	+	8.91	1.79E-12
mitochondrial gene expression (GO:0140053)	137	26	3.01	+	8.64	1.24E-11
respiratory electron transport chain (GO:0022904)	112	20	2.46	+	8.13	6.48E-08
regulation of cellular amino acid metabolic process (GO:000	62	11	1.36	+	8.08	3.75E-03
mitochondrial ATP synthesis coupled electron transport (GO	91	16	2	+	8.01	1.01E-05
ATP synthesis coupled electron transport (GO:0042773)	92	16	2.02	+	7.92	1.16E-05
aerobic respiration (GO:0009060)	79	13	1.73	+	7.49	7.34E-04
ribosomal small subunit biogenesis (GO:0042274)	67	11	1.47	+	7.48	7.45E-03
cellular respiration (GO:0045333)	168	26	3.69	+	7.05	8.82E-10
oxidative phosphorylation (GO:0006119)	117	18	2.57	+	7.01	6.29E-06
ribosome assembly (GO:0042255)	66	10	1.45	+	6.9	4.47E-02
regulation of cellular amine metabolic process (GO:003323	83	12	1.82	+	6.58	7.99E-03
purine nucleoside triphosphate biosynthetic process (GO:00	83	12	1.82	+	6.58	7.99E-03
nucleoside triphosphate biosynthetic process (GO:0009142)	100	14	2.2	+	6.38	1.42E-03
protein-containing complex disassembly (GO:0032984)	229	32	5.03	+	6.36	1.58E-11
ribonucleoside triphosphate biosynthetic process (GO:0009	88	12	1.93	+	6.21	1.39E-02
nucleoside triphosphate metabolic process (GO:0009141)	259	35	5.69	+	6.15	1.83E-12
electron transport chain (GO:0022900)	185	25	4.06	+	6.15	3.92E-08
ribonucleoside triphosphate metabolic process (GO:000919	238	32	5.23	+	6.12	4.19E-11
purine ribonucleoside triphosphate biosynthetic process (G	82	11	1.8	+	6.11	4.37E-02
purine nucleoside triphosphate metabolic process (GO:0009	239	32	5.25	+	6.1	4.65E-11
purine ribonucleoside triphosphate metabolic process (GO:	232	31	5.09	+	6.09	1.32E-10
ATP metabolic process (GO:0046034)	201	26	4.41	+	5.89	3.56E-08
energy derivation by oxidation of organic compounds (GO:0	238	29	5.23	+	5.55	7.75E-09
translation (GO:0006412)	395	48	8.67	+	5.53	1.97E-16
peptide biosynthetic process (GO:0043043)	418	49	9.18	+	5.34	3.06E-16
ribonucleoprotein complex assembly (GO:0022618)	227	25	4.98	+	5.02	2.10E-06
translational initiation (GO:0006413)	146	16	3.21	+	4.99	3.94E-03
ribonucleoprotein complex subunit organization (GO:00718	241	25	5.29	+	4.72	6.59E-06
generation of precursor metabolites and energy (GO:00060	413	42	9.07	+	4.63	1.69E-11
purine ribonucleotide biosynthetic process (GO:0009152)	187	19	4.11	+	4.63	9.88E-04
peptide metabolic process (GO:0006518)	558	56	12.25	+	4.57	4.20E-16
ribonucleotide biosynthetic process (GO:0009260)	201	20	4.41	+	4.53	6.28E-04
amide biosynthetic process (GO:0043604)	534	52	11.72	+	4.44	3.30E-14
purine nucleotide biosynthetic process (GO:0006164)	196	19	4.3	+	4.42	1.93E-03
ribose phosphate biosynthetic process (GO:0046390)	207	20	4.55	+	4.4	9.76E-04
ribonucleoprotein complex biogenesis (GO:0022613)	442	42	9.7	+	4.33	1.45E-10
ribosome biogenesis (GO:0042254)	289	27	6.35	+	4.26	1.14E-05
purine ribonucleotide metabolic process (GO:0009150)	436	40	9.57	+	4.18	1.80E-09
purine-containing compound biosynthetic process (GO:0072	209	19	4.59	+	4.14	4.79E-03
ribonucleotide metabolic process (GO:0009259)	452	41	9.92	+	4.13	1.25E-09
ribose phosphate metabolic process (GO:0019693)	471	42	10.34	+	4.06	1.05E-09
purine nucleotide metabolic process (GO:0006163)	457	40	10.03	+	3.99	7.19E-09
cellular component disassembly (GO:0022411)	383	33	8.41	+	3.92	1.14E-06
purine-containing compound metabolic process (GO:00725	498	42	10.93	+	3.84	5.83E-09
nucleotide metabolic process (GO:0009117)	594	50	13.04	+	3.83	3.40E-11
nucleoside phosphate metabolic process (GO:0006753)	601	50	13.2	+	3.79	5.21E-11
nucleoside phosphate biosynthetic process (GO:1901293)	289	24	6.35	+	3.78	7.55E-04
cellular amide metabolic process (GO:0043603)	805	65	17.68	+	3.68	1.15E-14
nucleotide biosynthetic process (GO:0009165)	285	23	6.26	+	3.68	2.25E-03
nucleobase-containing small molecule metabolic process (G	688	55	15.11	+	3.64	9.92E-12
drug metabolic process (GO:0017144)	665	45	14.6	+	3.08	8.86E-07
mitochondrion organization (GO:0007005)	444	28	9.75	+	2.87	1.27E-02
cellular protein-containing complex assembly (GO:0034622	825	52	18.11	+	2.87	3.26E-07
organonitrogen compound biosynthetic process (GO:19015	1442	88	31.66	+	2.78	1.01E-13
organophosphate metabolic process (GO:0019637)	1068	65	23.45	+	2.77	3.84E-09
cellular nitrogen compound biosynthetic process (GO:00442	1675	100	36.78	+	2.72	1.48E-15
oxidation-reduction process (GO:0055114)	949	56	20.84	+	2.69	5.90E-07
protein-containing complex subunit organization (GO:0043	1818	107	39.92	+	2.68	1.53E-16
organophosphate biosynthetic process (GO:0090407)	603	35	13.24	+	2.64	3.91E-03
symbiont process (GO:0044403)	767	42	16.84	+	2.49	1.35E-03
carbohydrate derivative metabolic process (GO:1901135)	1125	61	24.7	+	2.47	2.71E-06
viral process (GO:0016032)	690	37	15.15	+	2.44	1.20E-02
protein-containing complex assembly (GO:0065003)	1538	82	33.77	+	2.43	2.85E-09
gene expression (GO:0010467)	2005	106	44.02	+	2.41	6.08E-13
small molecule metabolic process (GO:0044281)	1834	96	40.27	+	2.38	4.44E-11
interspecies interaction between organisms (GO:0044419)	810	42	17.78	+	2.36	5.92E-03
RNA processing (GO:0006396)	864	44	18.97	+	2.32	4.39E-03
carboxylic acid metabolic process (GO:0019752)	899	45	19.74	+	2.28	6.44E-03
cellular nitrogen compound metabolic process (GO:003464	3608	179	79.22	+	2.26	1.48E-23
organic substance biosynthetic process (GO:1901576)	2900	137	63.67	+	2.15	2.42E-14
biosynthetic process (GO:0009058)	2958	139	64.95	+	2.14	2.19E-14
cellular macromolecule biosynthetic process (GO:0034645)	1708	80	37.5	+	2.13	3.21E-06
cellular biosynthetic process (GO:0044249)	2795	130	61.37	+	2.12	9.69E-13
oxoacid metabolic process (GO:0043436)	989	46	21.72	+	2.12	3.72E-02

macromolecule biosynthetic process (GO:0009059)	1761	81	38.67	+	2.09	3.81E-06
organic acid metabolic process (GO:0006082)	1010	46	22.18	+	2.07	4.92E-02
intracellular transport (GO:0046907)	1537	67	33.75	+	1.99	1.22E-03
cellular catabolic process (GO:0044248)	1774	77	38.95	+	1.98	1.61E-04
organic substance catabolic process (GO:1901575)	1728	75	37.94	+	1.98	2.93E-04
catabolic process (GO:0009056)	2012	87	44.18	+	1.97	1.47E-05
nucleobase-containing compound metabolic process (GO:00	2960	126	64.99	+	1.94	2.39E-09
cellular component biogenesis (GO:0044085)	2680	114	58.84	+	1.94	4.83E-08
heterocycle metabolic process (GO:0046483)	3128	129	68.68	+	1.88	9.15E-09
phosphate-containing compound metabolic process (GO:00	2205	90	48.41	+	1.86	1.50E-04
phosphorus metabolic process (GO:0006793)	2232	91	49.01	+	1.86	1.15E-04
cellular aromatic compound metabolic process (GO:000672	3172	128	69.65	+	1.84	5.27E-08
RNA metabolic process (GO:0016070)	1669	66	36.65	+	1.8	4.88E-02
organic cyclic compound metabolic process (GO:1901360)	3391	134	74.45	+	1.8	7.42E-08
establishment of localization in cell (GO:0051649)	1786	70	39.21	+	1.79	2.90E-02
cellular component assembly (GO:0022607)	2459	96	53.99	+	1.78	3.10E-04
organonitrogen compound metabolic process (GO:1901564	5535	201	121.53	+	1.65	3.12E-11
cellular metabolic process (GO:0044237)	7670	276	168.41	+	1.64	9.88E-20
primary metabolic process (GO:0044238)	7725	272	169.61	+	1.6	1.16E-17
nitrogen compound metabolic process (GO:0006807)	7228	254	158.7	+	1.6	3.79E-15
organic substance metabolic process (GO:0071704)	8029	279	176.29	+	1.58	1.35E-17
metabolic process (GO:0008152)	8478	294	186.15	+	1.58	1.46E-19
cellular protein metabolic process (GO:0044267)	3729	129	81.88	+	1.58	9.39E-04
transport (GO:0006810)	4467	143	98.08	+	1.46	1.15E-02
cellular component organization or biogenesis (GO:007184(	5760	184	126.47	+	1.45	9.52E-05
cellular macromolecule metabolic process (GO:0044260)	5085	161	111.65	+	1.44	3.51E-03
protein metabolic process (GO:0019538)	4415	139	96.94	+	1.43	4.38E-02
establishment of localization (GO:0051234)	4588	144	100.74	+	1.43	3.36E-02
macromolecule metabolic process (GO:0043170)	6258	196	137.4	+	1.43	1.16E-04
cellular component organization (GO:0016043)	5580	173	122.52	+	1.41	3.41E-03
cellular process (GO:0009987)	14424	382	316.7	+	1.21	7.63E-08
biological_process (GO:0008150)	17772	424	390.21	+	1.09	3.42E-02
Unclassified (UNCLASSIFIED)	3224	37	70.79	-	0.52	0.00E+00
system process (GO:0003008)	1933	15	42.44	-	0.35	9.10E-03
G protein-coupled receptor signaling pathway (GO:000718(	1318	8	28.94	-	0.28	4.77E-02
sensory perception (GO:0007600)	948	3	20.81	-	0.14	2.55E-02

Table S13d. Geneontology output for genes upregulated in high surviving, younger pediatric ALL patients

Analysis Type:	PANTHER Overrepresentation Test (Released 20181113)						
Annotation Version and Release Date:	GO Ontology database Released 2019-01-01						
Analyzed List:	upload_1 (Homo sapiens)						
Reference List:	Homo sapiens (all genes in database)						
Test Type:	FISHER						
Correction:	BONFERRONI						
Bonferroni count:		8749					
GO biological process complete	Homo sapiens - REFLIST (20996)		upload_1 (881)	upload_1 (expected)	upload_1 (over/under)	upload_1 (fold Enrichment)	upload_1 (P-value)
Unclassified (UNCLASSIFIED)		3224	128	135.28	-	0.95	0.00E+00

Table S14. KM curve median values for pediatric NBL genes. Note, “undefined” indicates survival did not reach less than 50% of population.

	Median survival (days)				
**Gene**	**Oldest Quintile, High Expressors**	**Oldest Quintile, Low Expressors**	**Youngest Quintile, High Expressors**	**Youngest Quintile, Low Expressors**	**p-value**
USP17L5	1235	3691	undefined	undefined	<0.0001
SLC25A5	972	3691	undefined	undefined	<0.0001
POF1B	3691	833	undefined	undefined	<0.0001
RND3	undefined	1235	undefined	undefined	<0.0001
KLC4	3691	1083	undefined	undefined	<0.0001
SLC12A1	undefined	972	undefined	undefined	<0.0001

KM curve median valued for pediatric ALL genes. Note, “undefined” indicates survival did not reach less than 50% of population.

	Median survival (days)				
**Gene**	**Oldest Quintile, High Expressors**	**Oldest Quintile, Low Expressors**	**Youngest Quintile, High Expressors**	**Youngest Quintile, Low Expressors**	**p-value**
THAP4	584	1592	977	undefined	0.0255
ZNHIT2	584	1914	1068	3665	0.0045
SF3B2	504	1688	977	undefined	0.0003
COL5A1	1688	584	3665	977	0.0050
GABBR1	1914	584	3798.5	919	0.0003
HACE1	1688	846	undefined	977	0.0122
RPS6KA5	1914	584	undefined	1344	0.0013
LAMB1	1571	790	undefined	977	0.0281
BMP3	2029	474	undefined	919	<0.0001
MAML3	1914	790	undefined	977	0.0019
SLX4IP	1914	692	3665	1068	0.0022
EPHA7	1688	846	undefined	919	0.0113
OR52H1	1914	584	undefined	1068	0.0032
DDX60L	1688	474	undefined	977	0.0023
SNORA19	1688	790	undefined	977	0.0127
SNORA2A	1688	692	undefined	977	0.0061
ENTHD2	1688	790	undefined	919	0.0016
TRIP11	1688	584	undefined	1068	0.0064
ZNF81	1688	474	undefined	1068	0.0080
ZNF514	1914	584	undefined	1068	0.0006

Fig. S1 (follows).
KM curve panels for all genes in Table S14.

Pediatric NBL, upregulated genes in older, low surviving patients

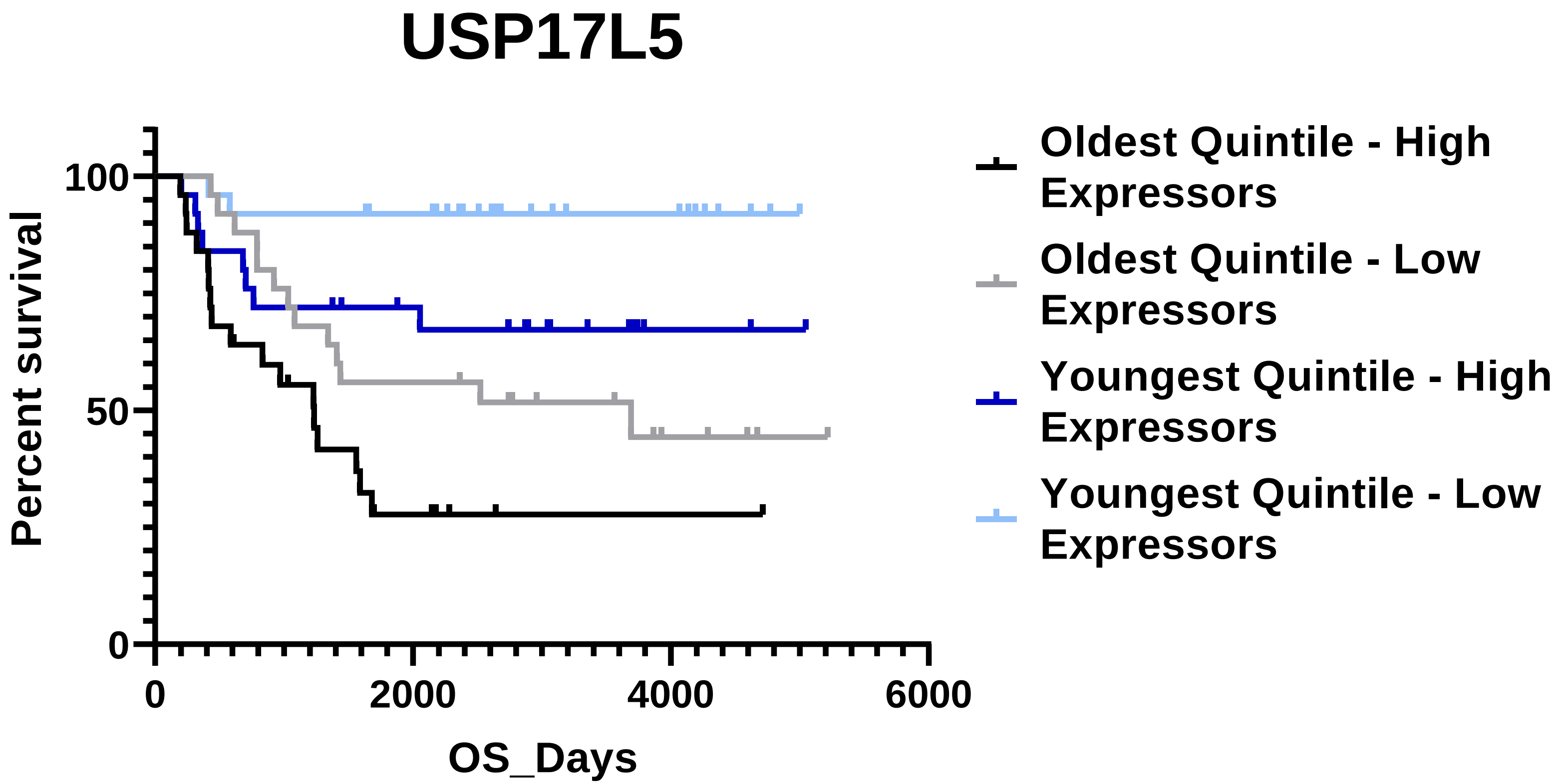
USP17L5
Percent survival
100
50
0
0
2000
4000
6000
OS_Days
Oldest Quintile - High Expressors
Oldest Quintile - Low Expressors
Youngest Quintile - High Expressors
Youngest Quintile - Low Expressors

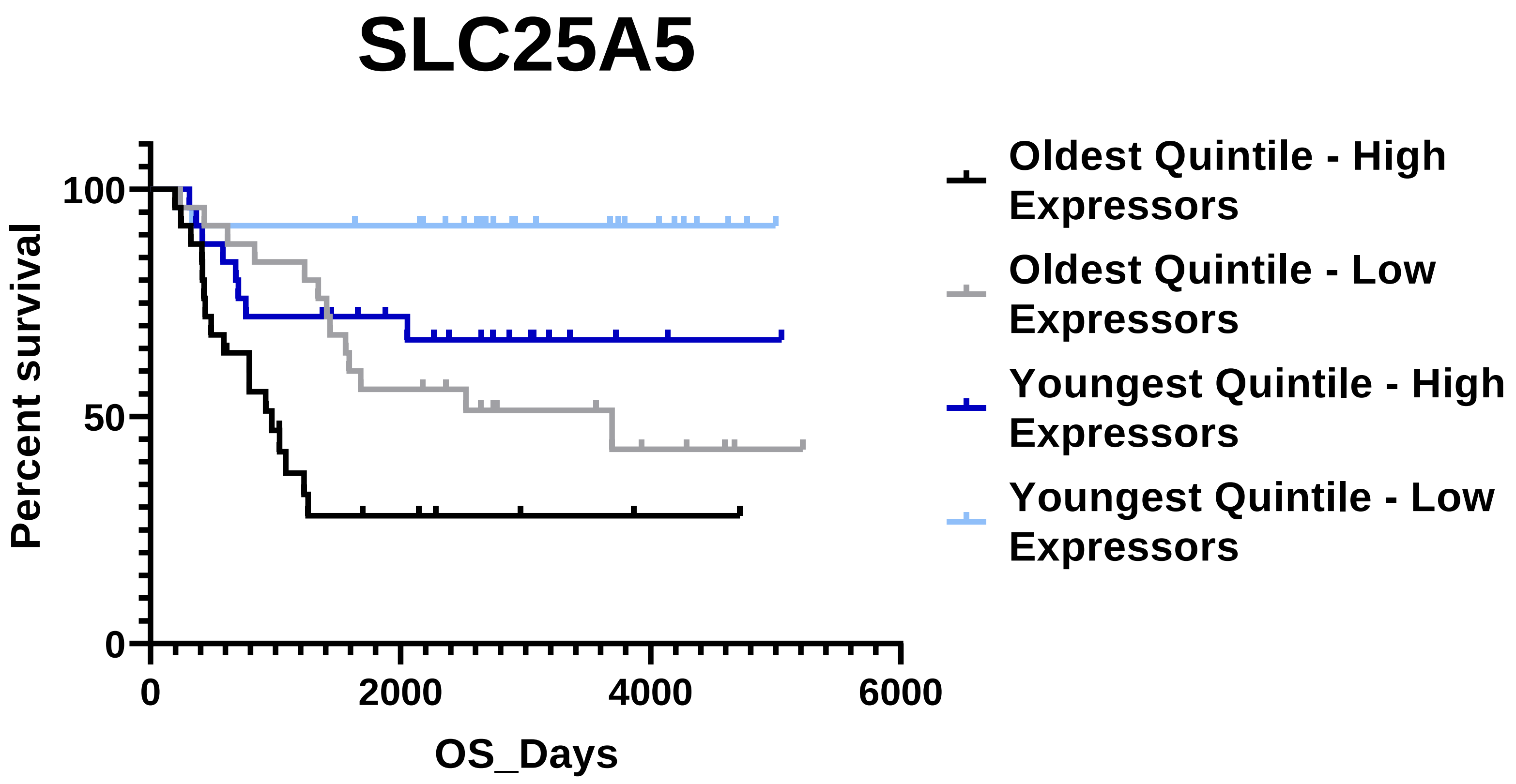
SLC25A5
Percent survival
100
50
0
0
2000
4000
6000
OS_Days
Oldest Quintile - High Expressors
Oldest Quintile - Low Expressors
Youngest Quintile - High Expressors
Youngest Quintile - Low Expressors

Pediatric NBL, upregulated genes in younger, high surviving patients

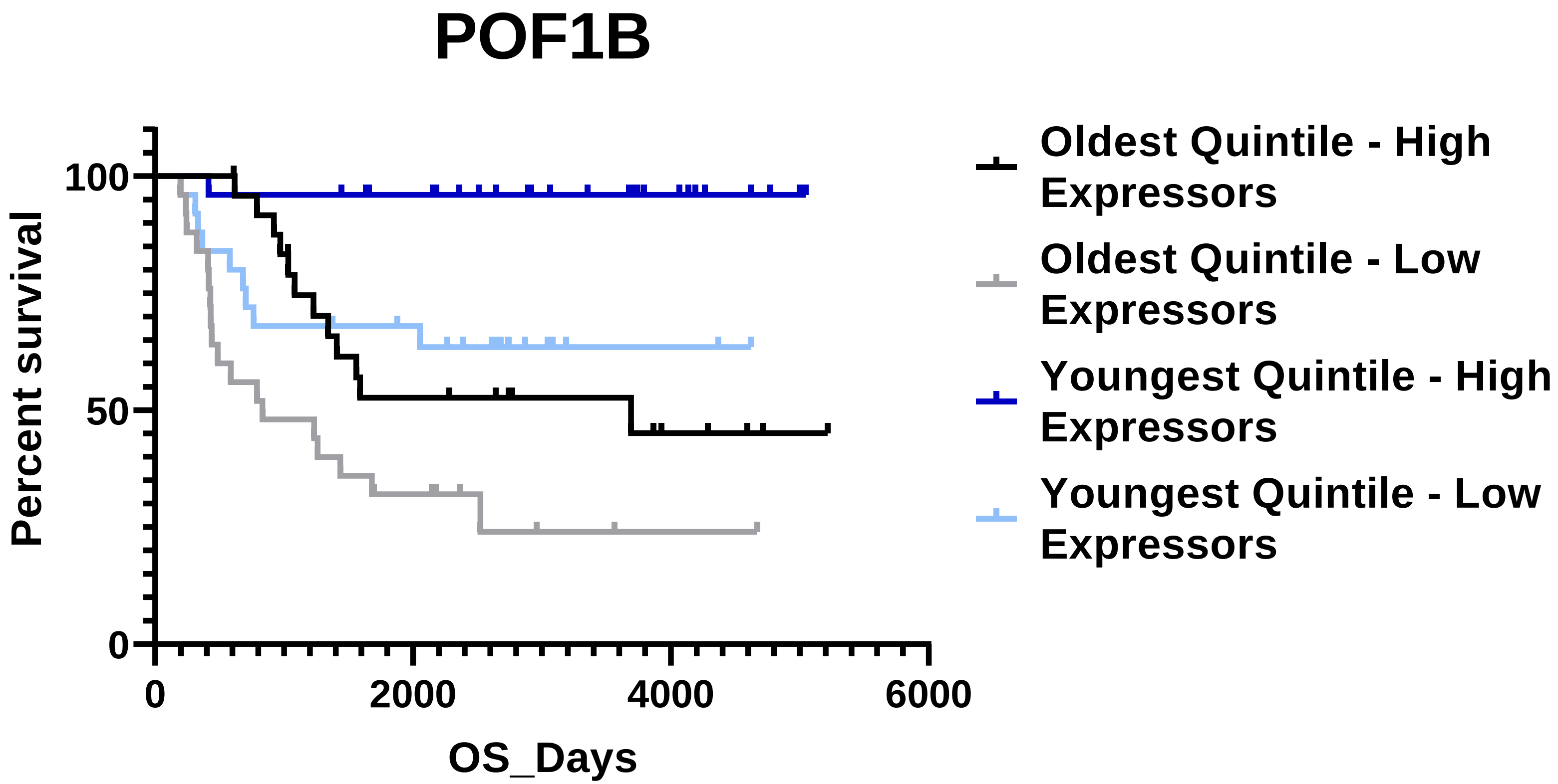
POF1B
Percent survival
100
50
0
0
2000
4000
6000
OS_Days
Oldest Quintile - High Expressors
Oldest Quintile - Low Expressors
Youngest Quintile - High Expressors
Youngest Quintile - Low Expressors

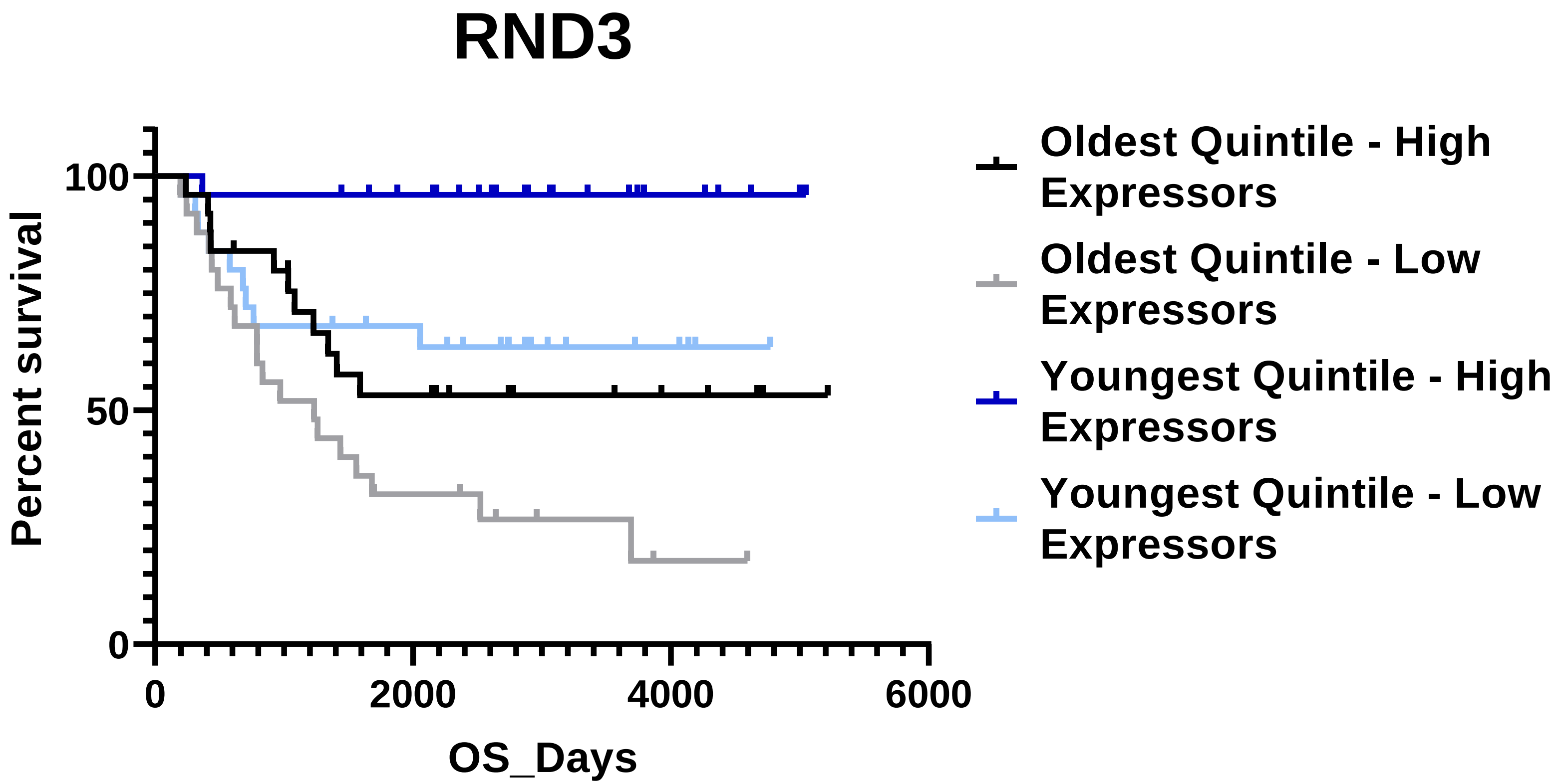
RND3
Percent survival
100
50
0
0
2000
4000
6000
OS_Days
Oldest Quintile - High Expressors
Oldest Quintile - Low Expressors
Youngest Quintile - High Expressors
Youngest Quintile - Low Expressors

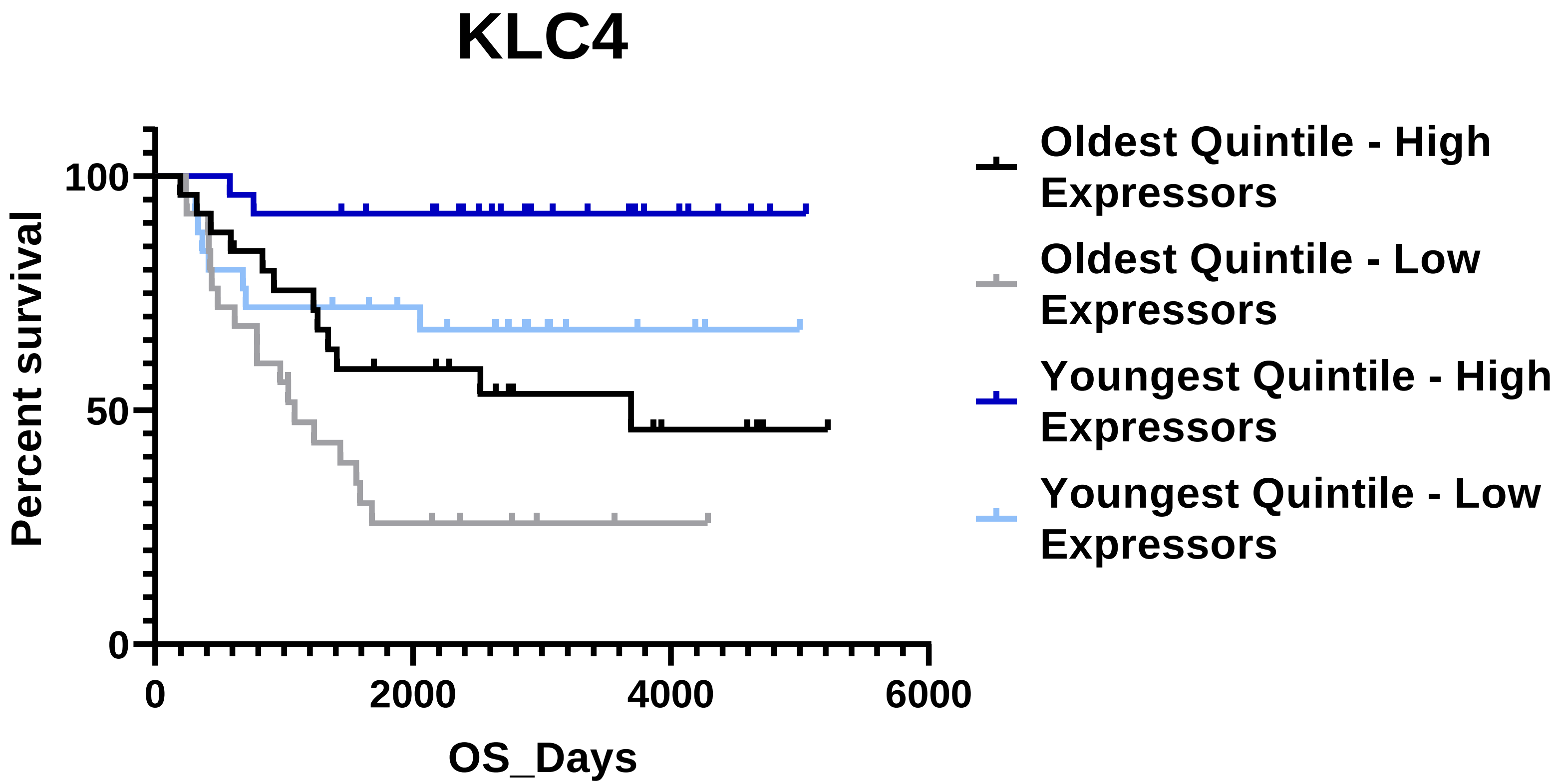
KLC4
Percent survival
100
50
0
0
2000
4000
6000
OS_Days
Oldest Quintile - High Expressors
Oldest Quintile - Low Expressors
Youngest Quintile - High Expressors
Youngest Quintile - Low Expressors

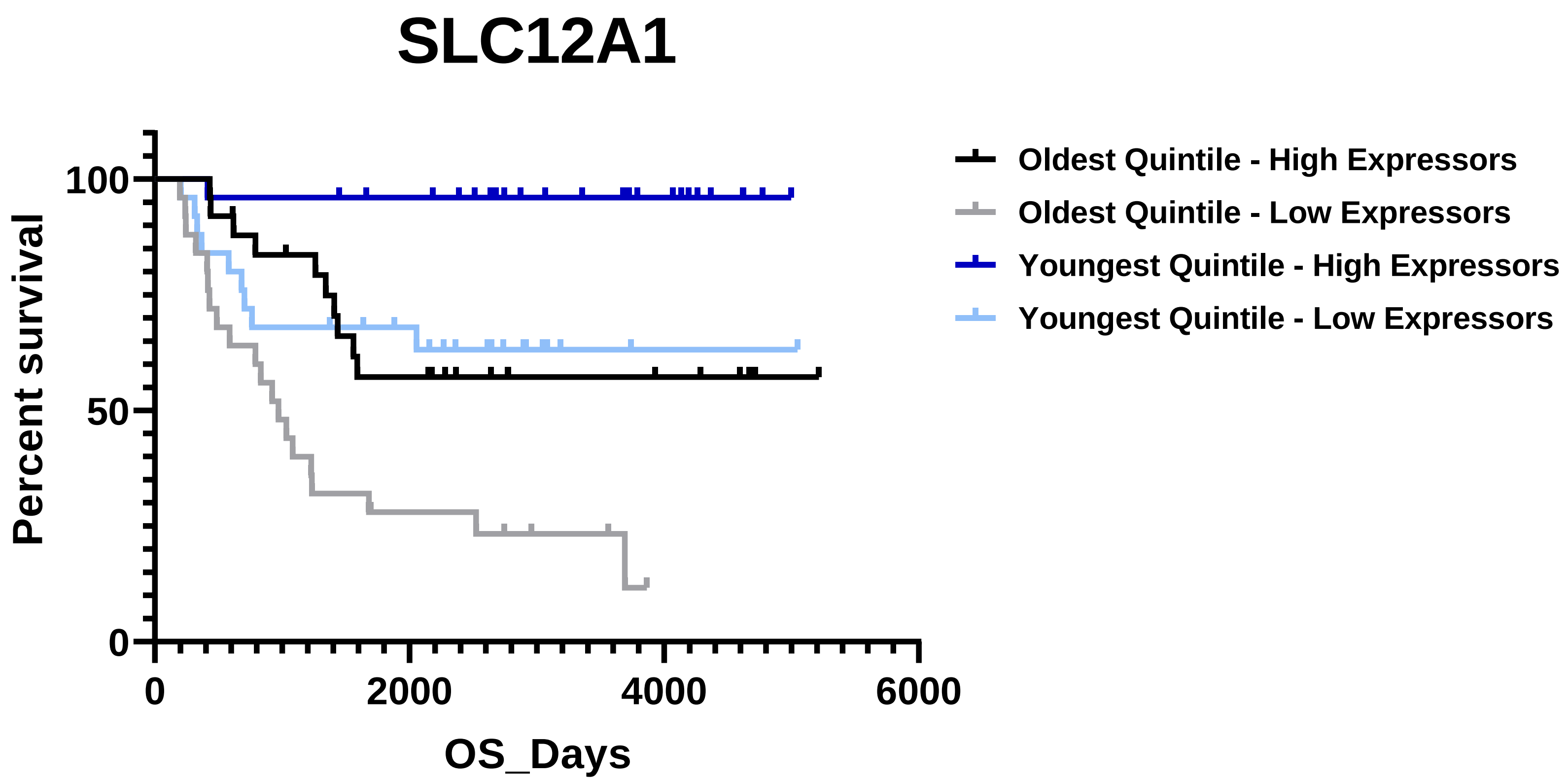
SLC12A1
Percent survival
100
50
0
0
2000
4000
6000
OS_Days
Oldest Quintile - High Expressors
Oldest Quintile - Low Expressors
Youngest Quintile - High Expressors
Youngest Quintile - Low Expressors

Pediatric ALL, upregulated genes in older, low surviving patients

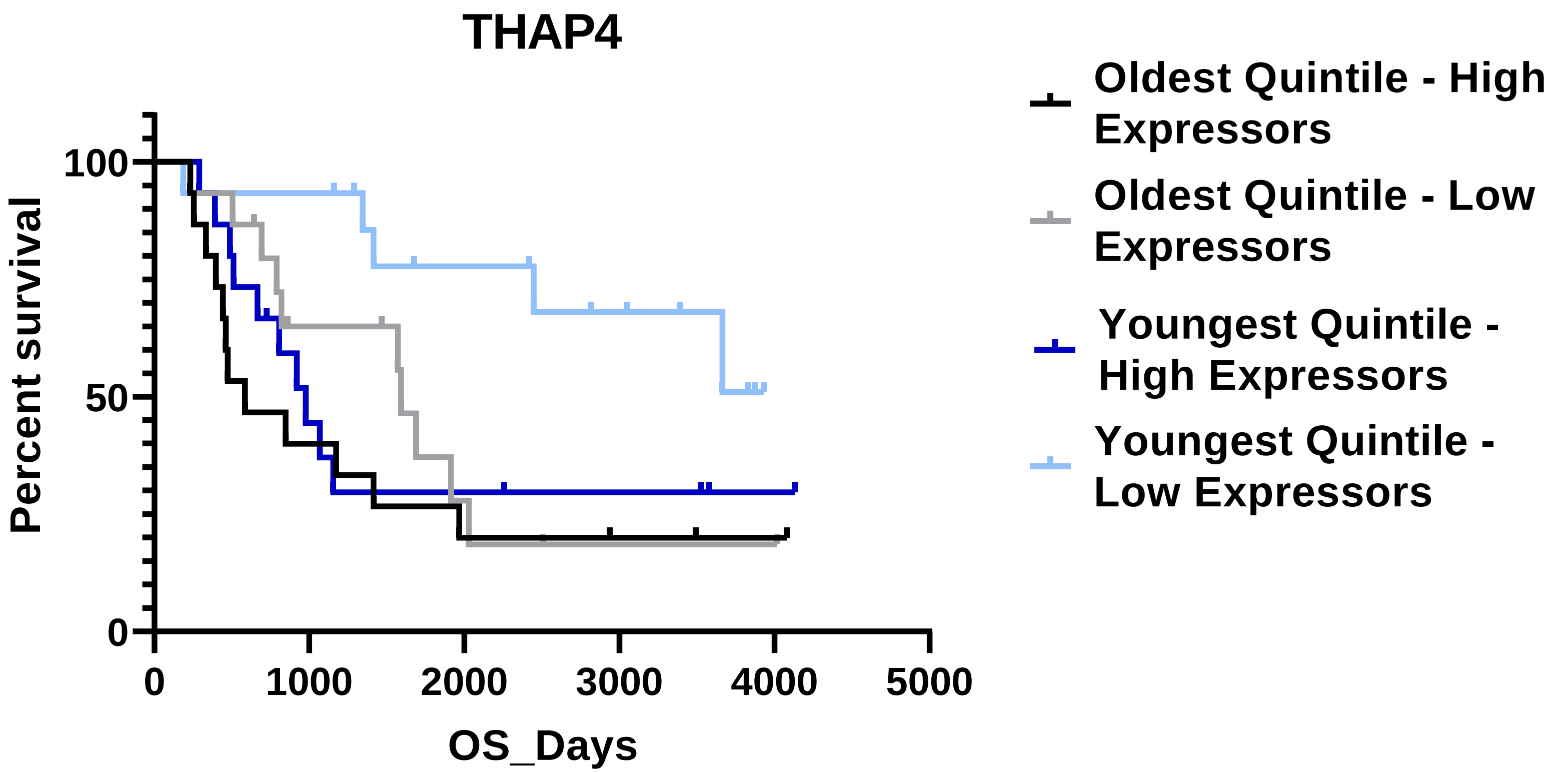
THAP4
Percent survival
100
50
0
0
1000
2000
3000
4000
5000
OS_Days
Oldest Quintile - High Expressors
Oldest Quintile - Low Expressors
Youngest Quintile - High Expressors
Youngest Quintile - Low Expressors

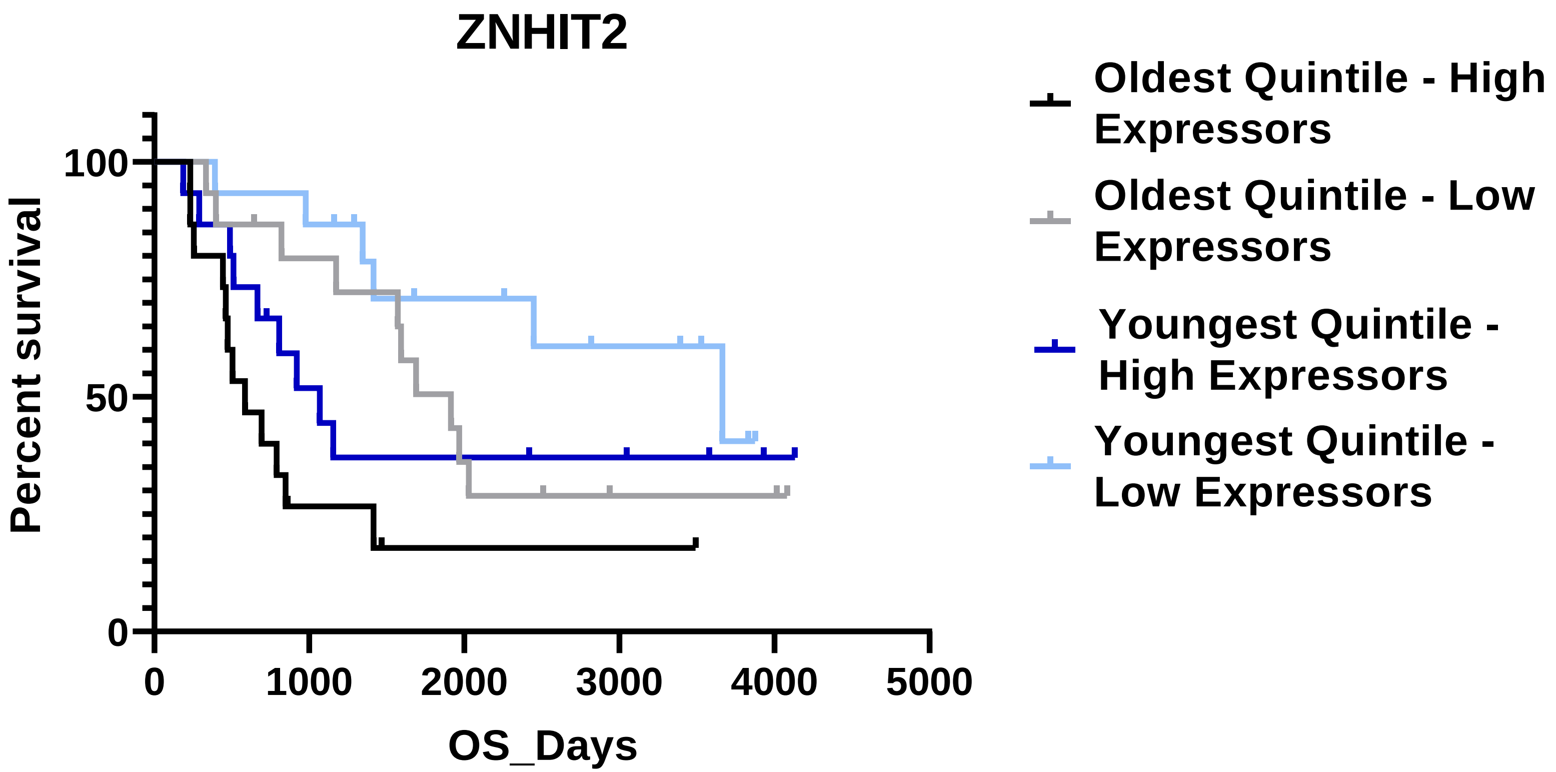
ZNHIT2
Percent survival
100
50
0
0
1000
2000
3000
4000
5000
OS_Days
Oldest Quintile - High Expressors
Oldest Quintile - Low Expressors
Youngest Quintile - High Expressors
Youngest Quintile - Low Expressors

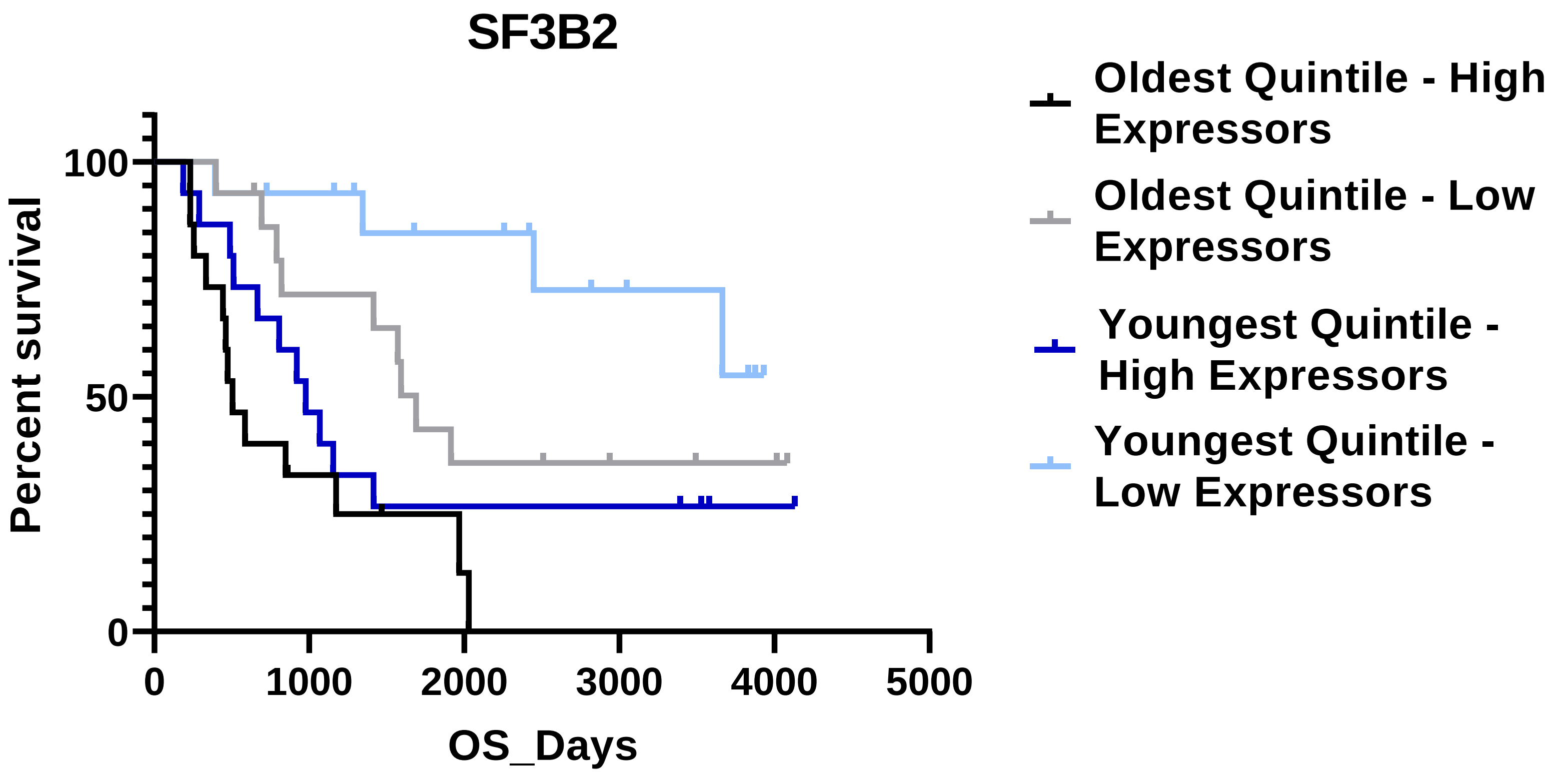
SF3B2
Percent survival
100
50
0
0
1000
2000
3000
4000
5000
OS_Days
Oldest Quintile - High Expressors
Oldest Quintile - Low Expressors
Youngest Quintile - High Expressors
Youngest Quintile - Low Expressors

Pediatric ALL, upregulated genes in younger, high surviving patients

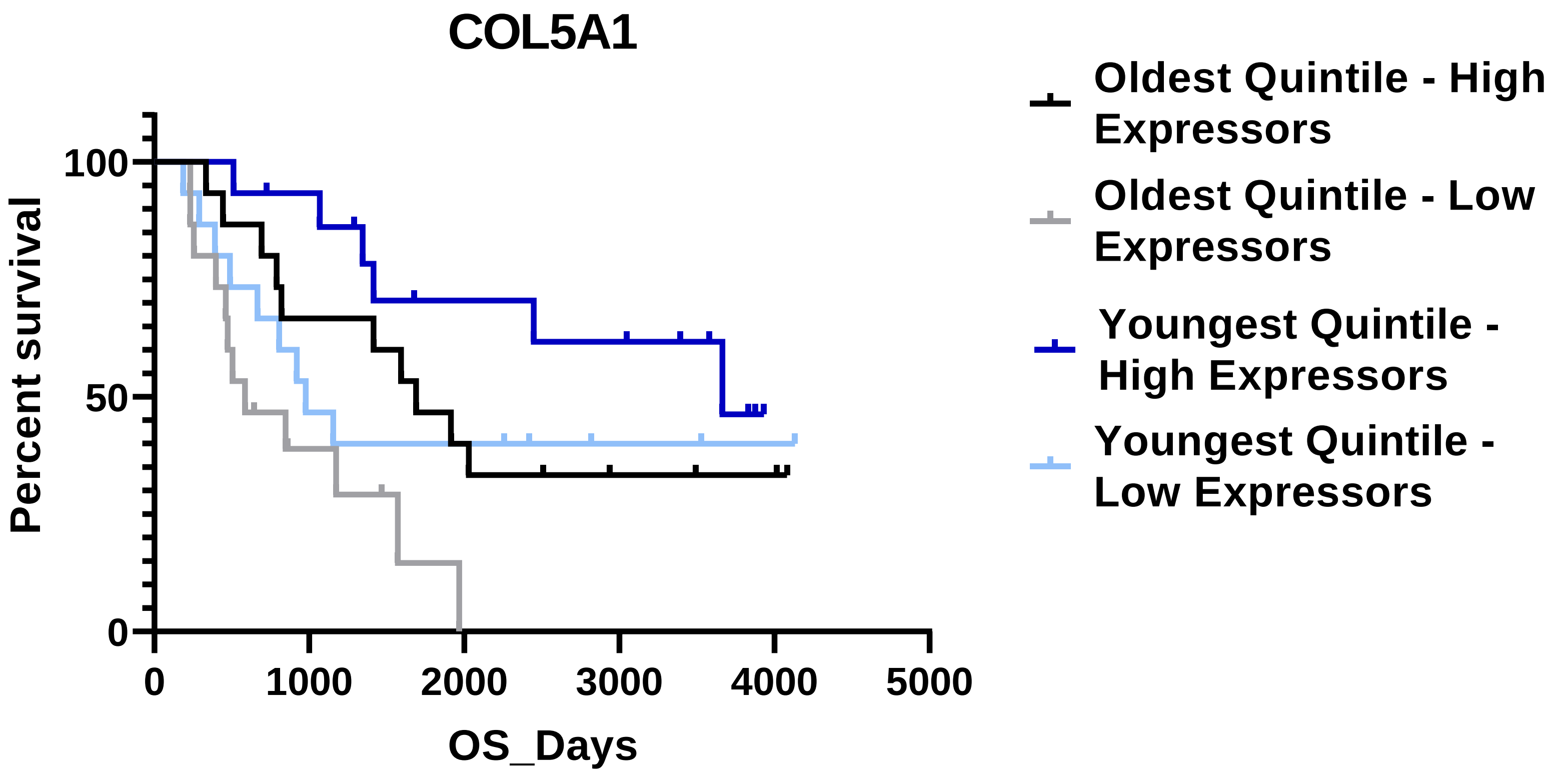

COL5A1
Percent survival
100
50
0
0
1000
2000
3000
4000
5000
OS_Days
Oldest Quintile - High Expressors
Oldest Quintile - Low Expressors
Youngest Quintile - High Expressors
Youngest Quintile - Low Expressors

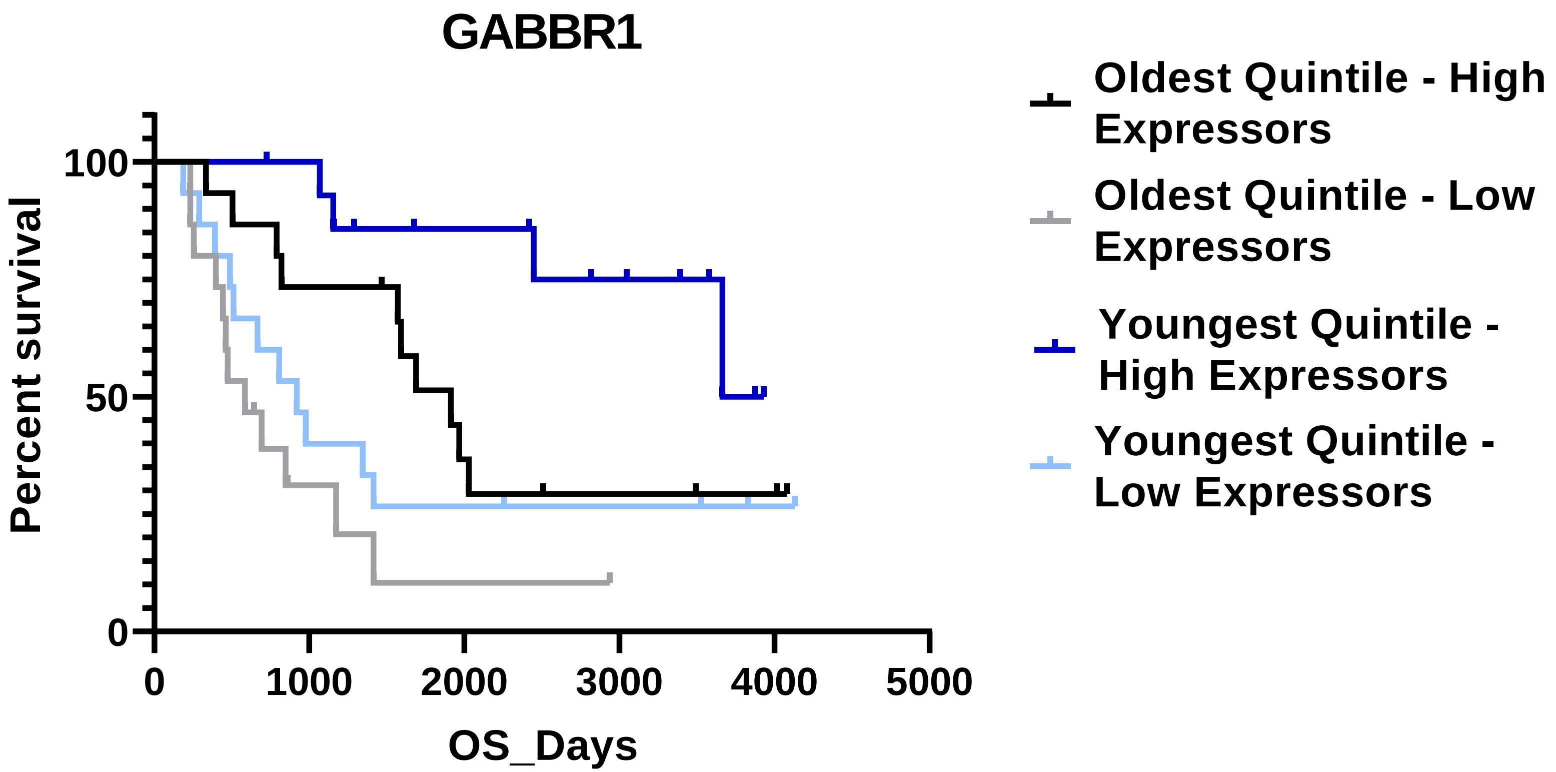
GABBR1
Percent survival
100
50
0
0
1000
2000
3000
4000
5000
OS_Days
Oldest Quintile - High Expressors
Oldest Quintile - Low Expressors
Youngest Quintile - High Expressors
Youngest Quintile - Low Expressors

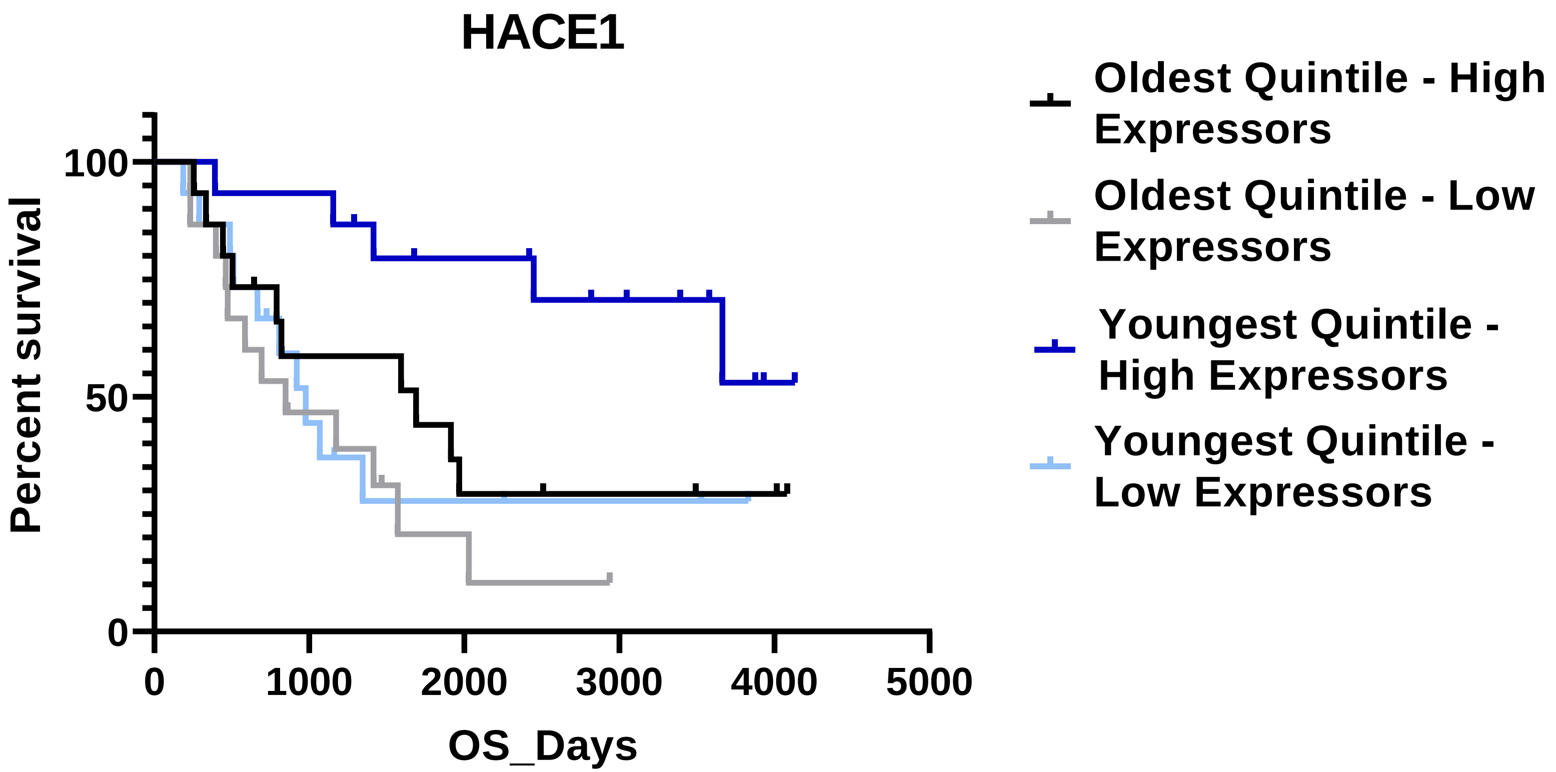
HACE1
Percent survival
100
50
0
0
1000
2000
3000
4000
5000
OS_Days
Oldest Quintile - High Expressors
Oldest Quintile - Low Expressors
Youngest Quintile - High Expressors
Youngest Quintile - Low Expressors

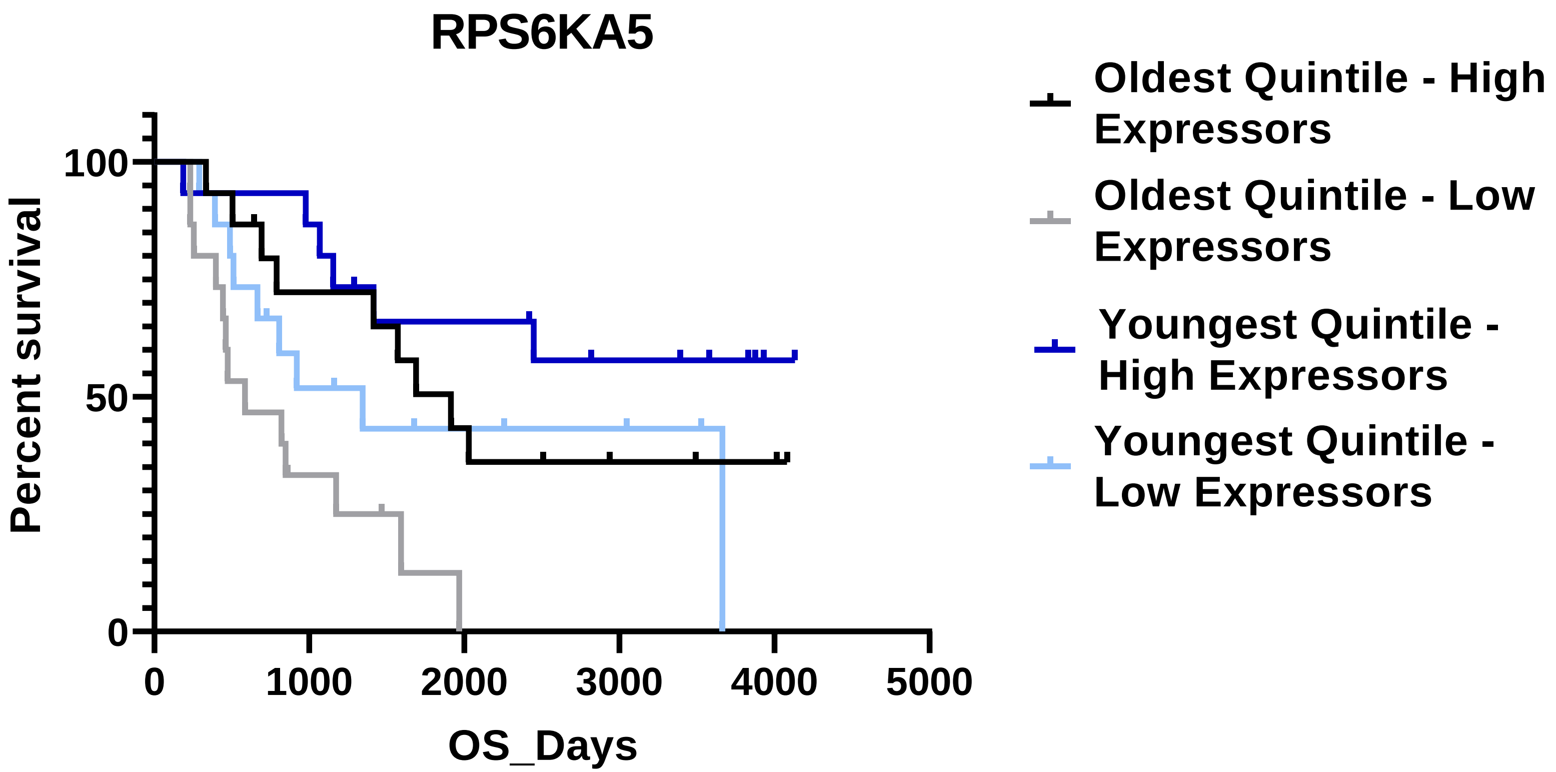

RPS6KA5
Percent survival
100
50
0
0
1000
2000
3000
4000
5000
OS_Days
Oldest Quintile - High Expressors
Oldest Quintile - Low Expressors
Youngest Quintile - High Expressors
Youngest Quintile - Low Expressors

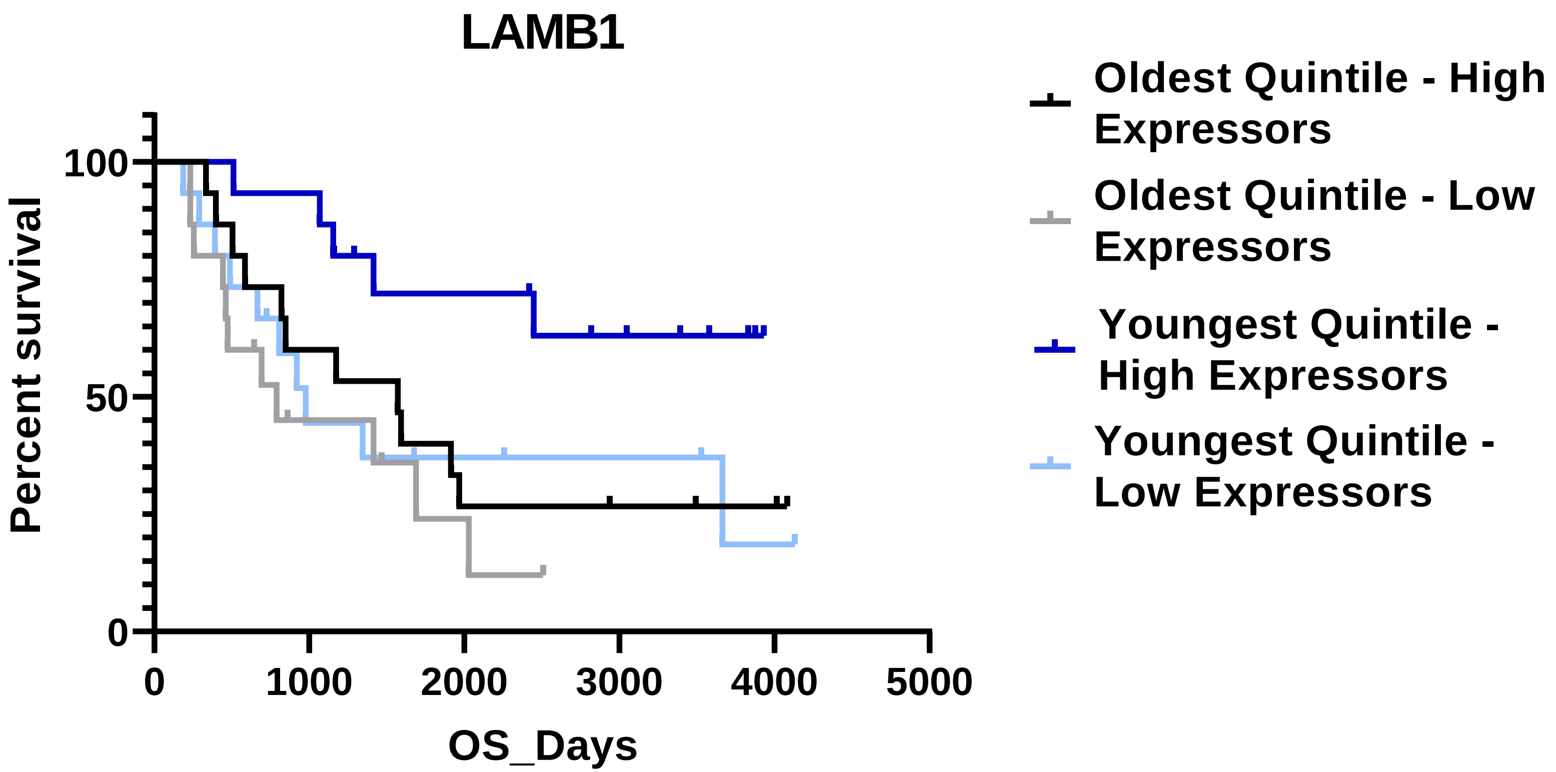

LAMB1
Percent survival
100
50
0
0
1000
2000
3000
4000
5000
OS_Days
Oldest Quintile - High Expressors
Oldest Quintile - Low Expressors
Youngest Quintile - High Expressors
Youngest Quintile - Low Expressors

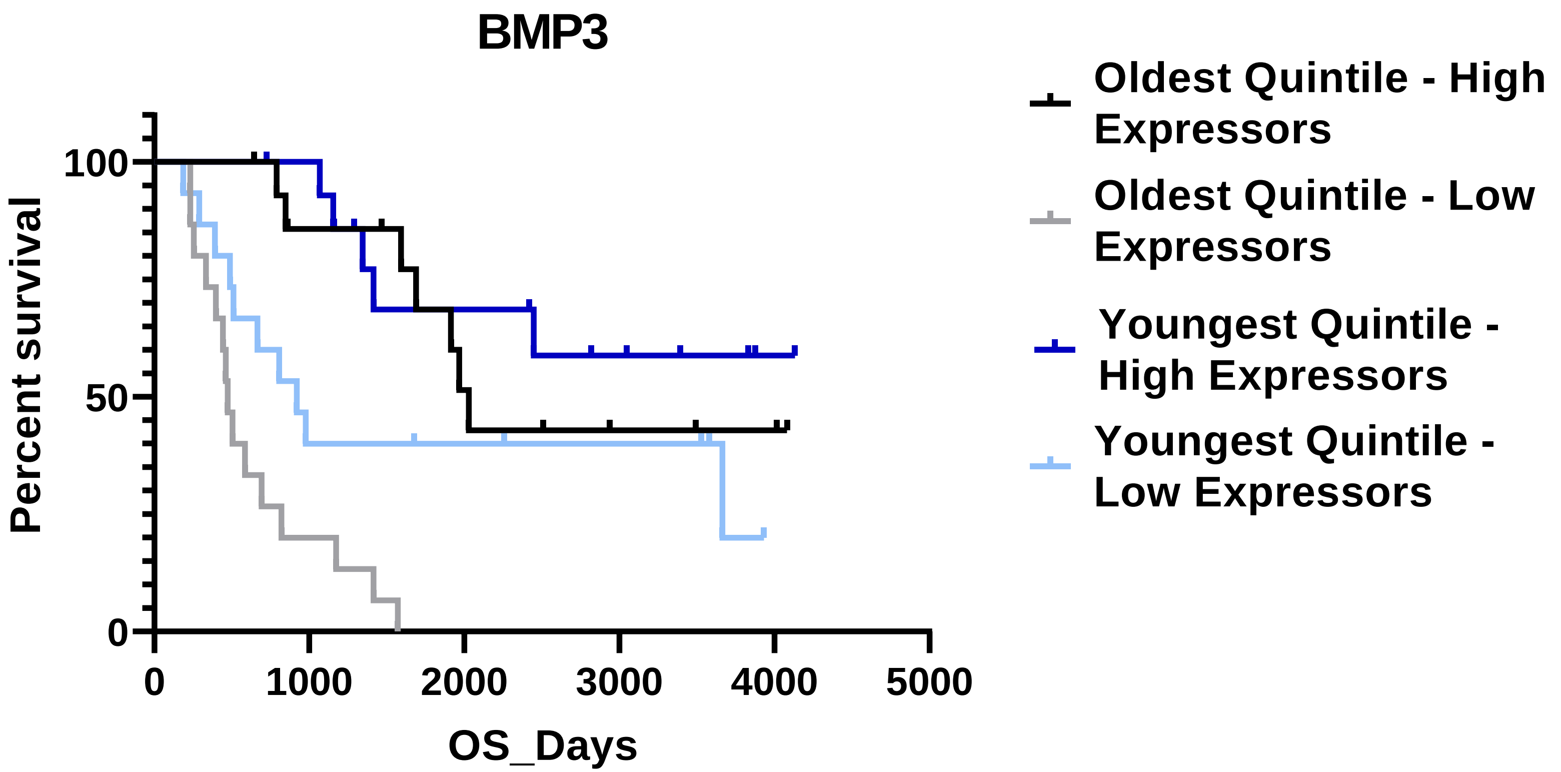
BMP3
Percent survival
100
50
0
0
1000
2000
3000
4000
5000
OS_Days
Oldest Quintile - High Expressors
Oldest Quintile - Low Expressors
Youngest Quintile - High Expressors
Youngest Quintile - Low Expressors

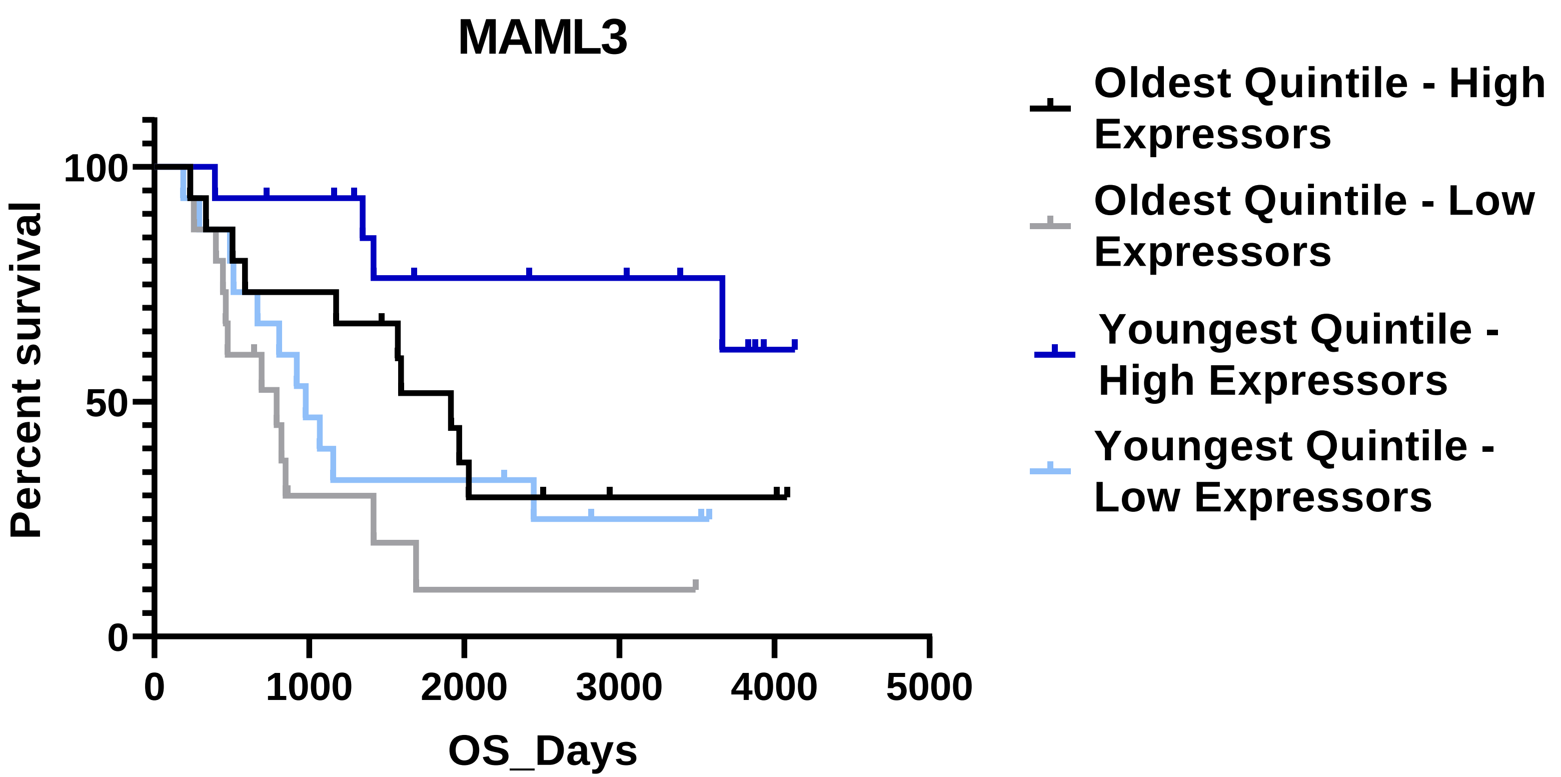

MAML3
Percent survival
100
50
0
0
1000
2000
3000
4000
5000
OS_Days
Oldest Quintile - High Expressors
Oldest Quintile - Low Expressors
Youngest Quintile - High Expressors
Youngest Quintile - Low Expressors

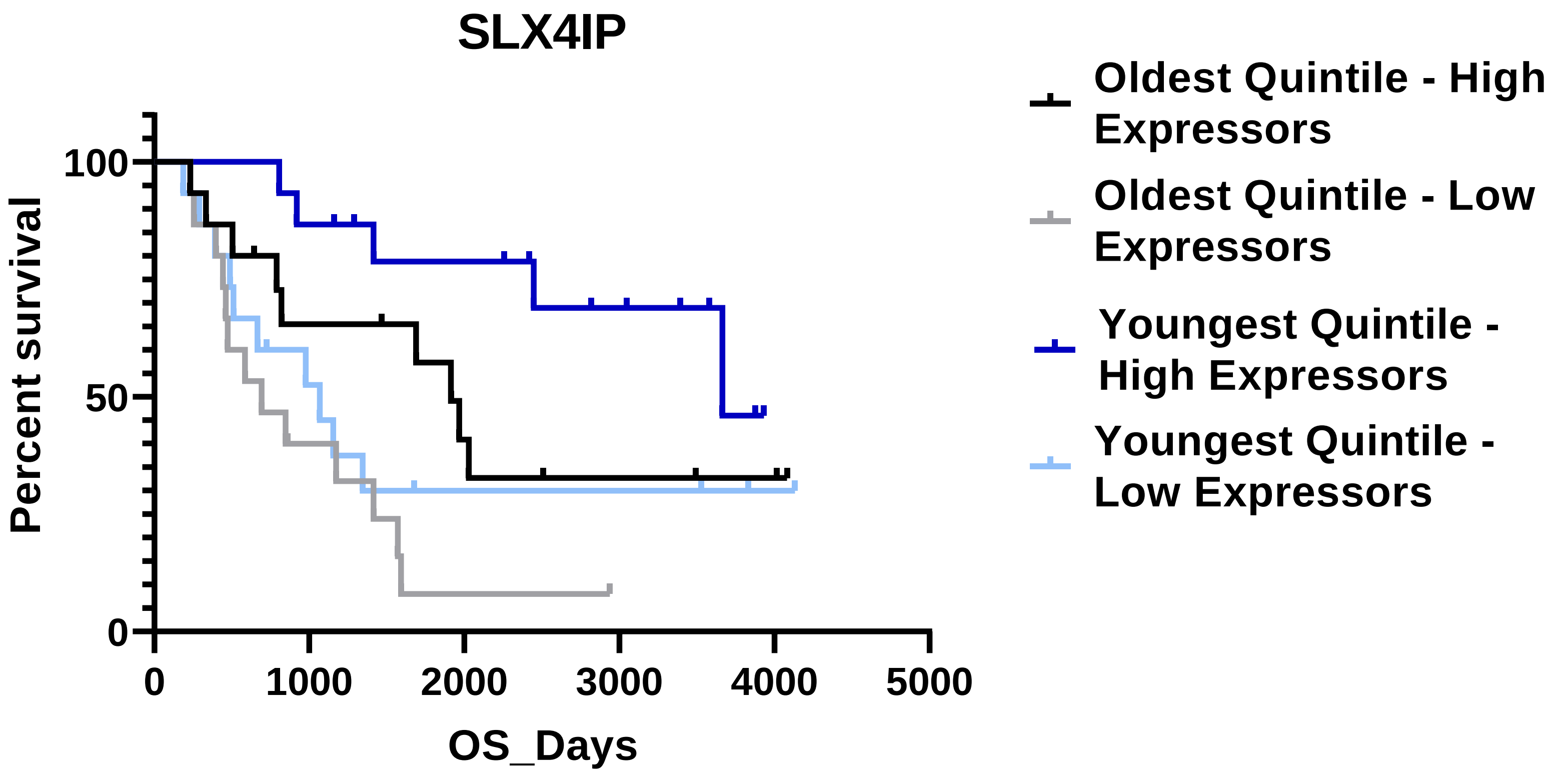
SLX4IP
Percent survival
100
50
0
0
1000
2000
3000
4000
5000
OS_Days
Oldest Quintile - High Expressors
Oldest Quintile - Low Expressors
Youngest Quintile - High Expressors
Youngest Quintile - Low Expressors

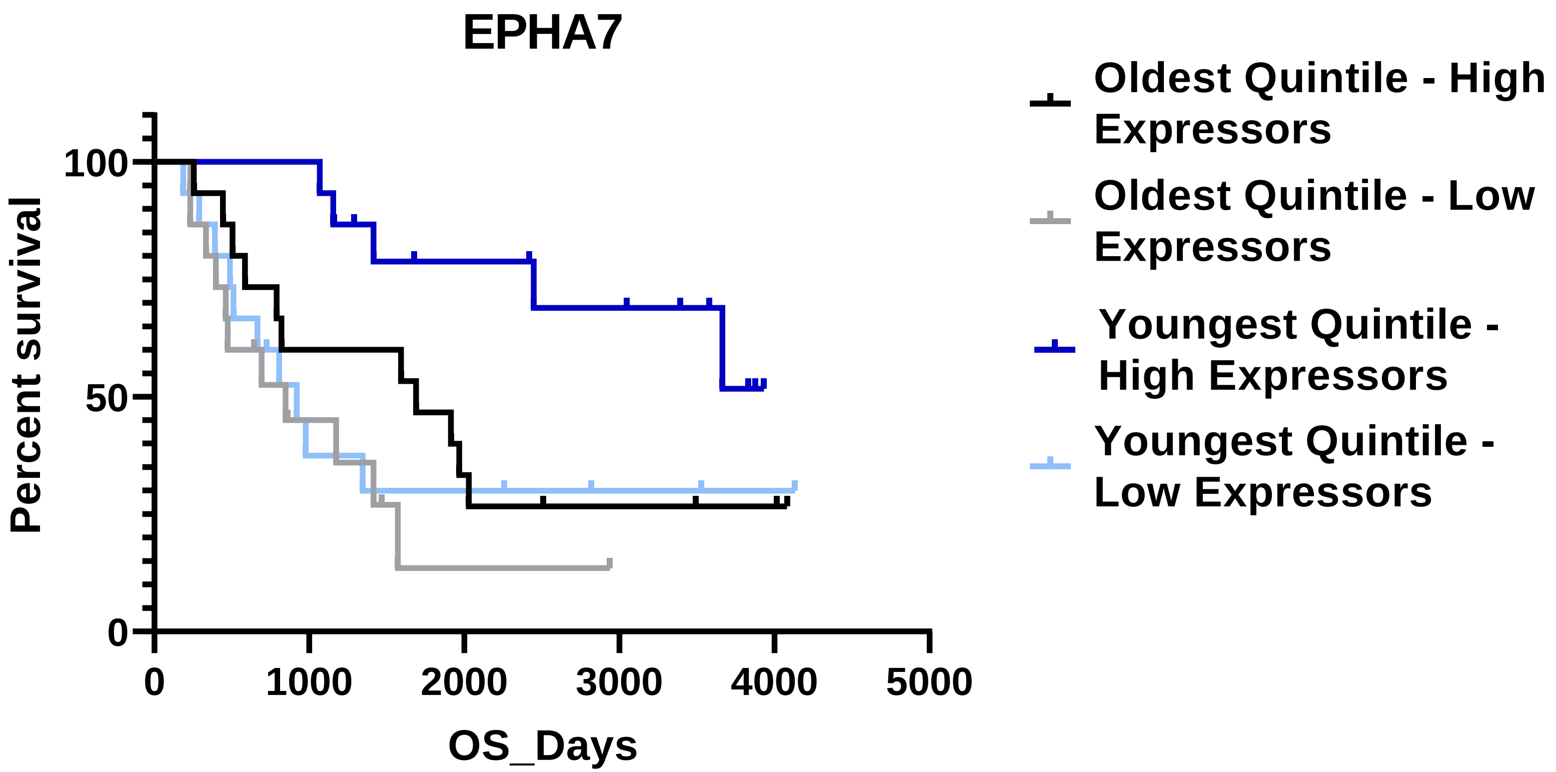
EPHA7
Percent survival
100
50
0
0
1000
2000
3000
4000
5000
OS_Days
Oldest Quintile - High Expressors
Oldest Quintile - Low Expressors
Youngest Quintile - High Expressors
Youngest Quintile - Low Expressors

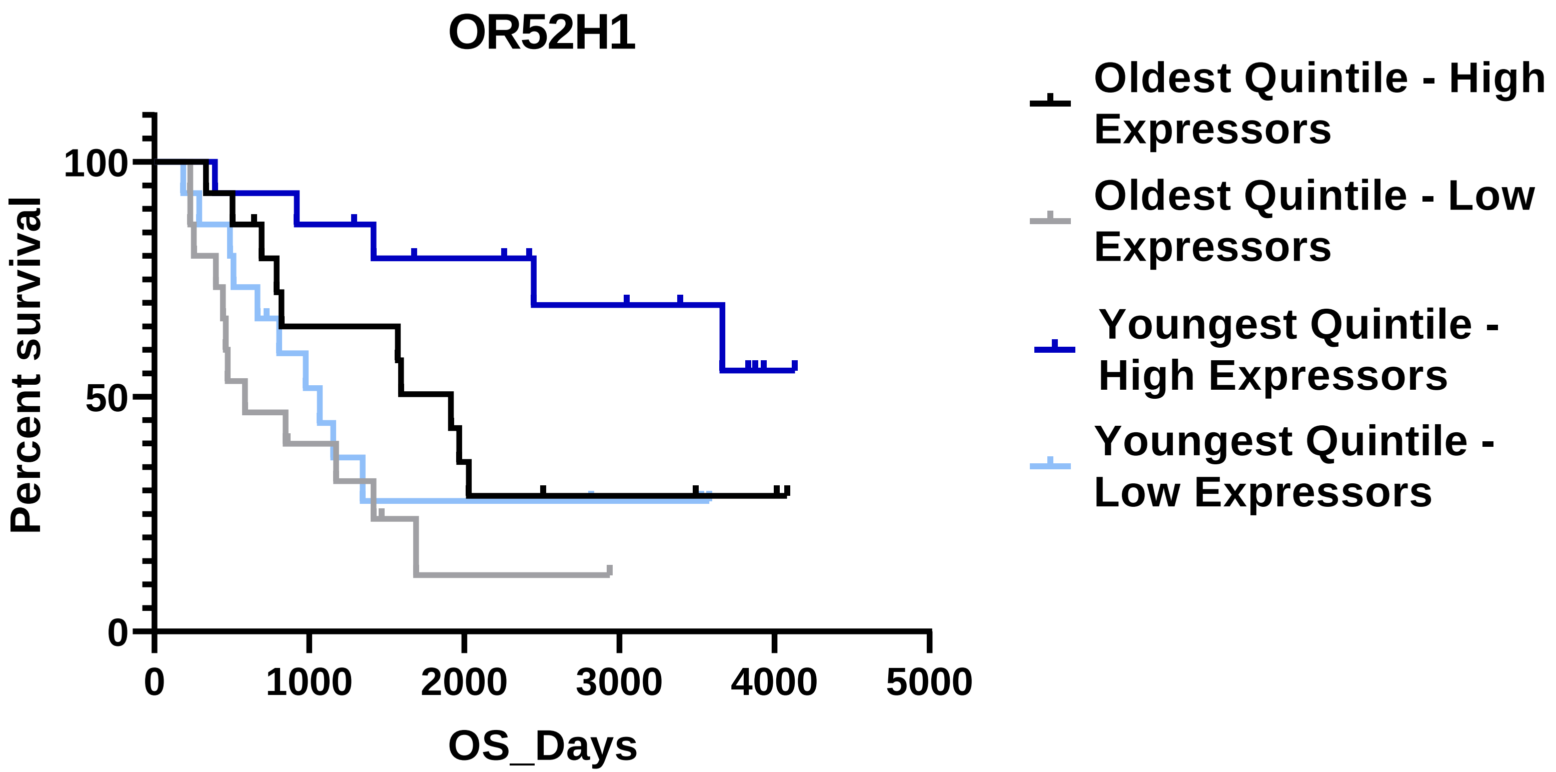
OR52H1
Percent survival
100
50
0
0
1000
2000
3000
4000
5000
OS_Days
Oldest Quintile - High Expressors
Oldest Quintile - Low Expressors
Youngest Quintile - High Expressors
Youngest Quintile - Low Expressors

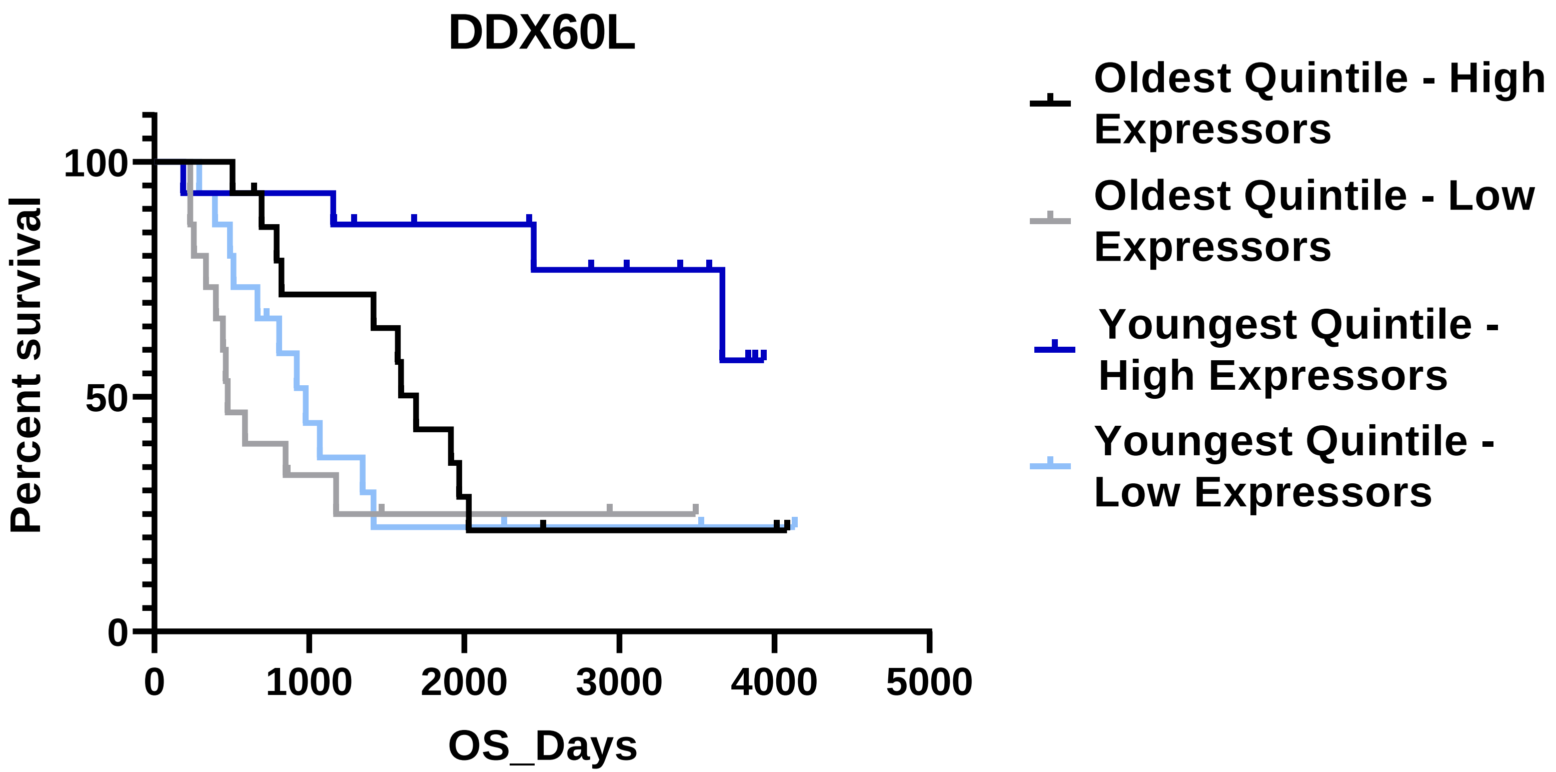
DDX60L
Percent survival
100
50
0
0
1000
2000
3000
4000
5000
OS_Days
Oldest Quintile - High Expressors
Oldest Quintile - Low Expressors
Youngest Quintile - High Expressors
Youngest Quintile - Low Expressors

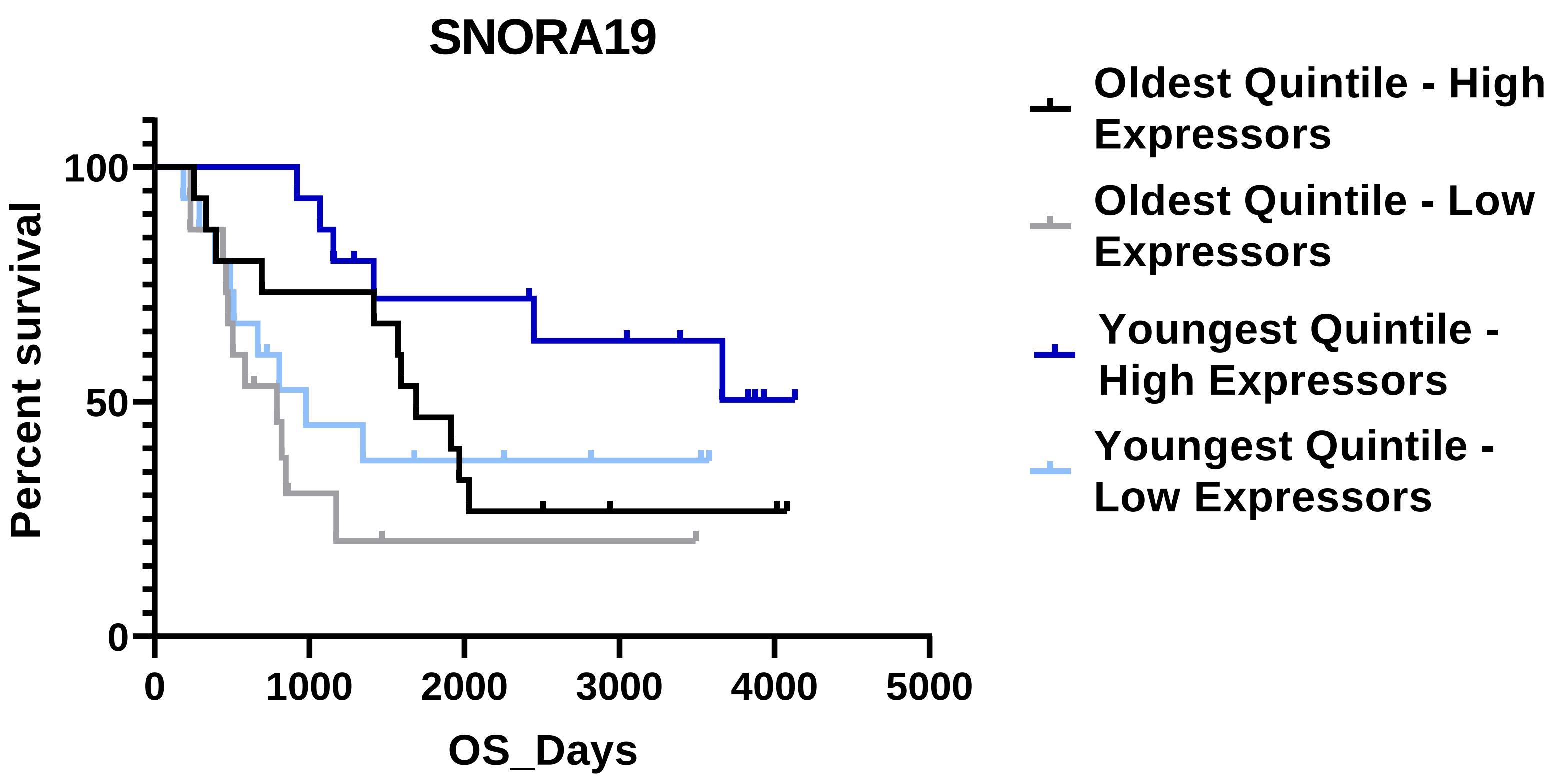

SNORA19
Percent survival
100
50
0
0
1000
2000
3000
4000
5000
OS_Days
Oldest Quintile - High Expressors
Oldest Quintile - Low Expressors
Youngest Quintile - High Expressors
Youngest Quintile - Low Expressors

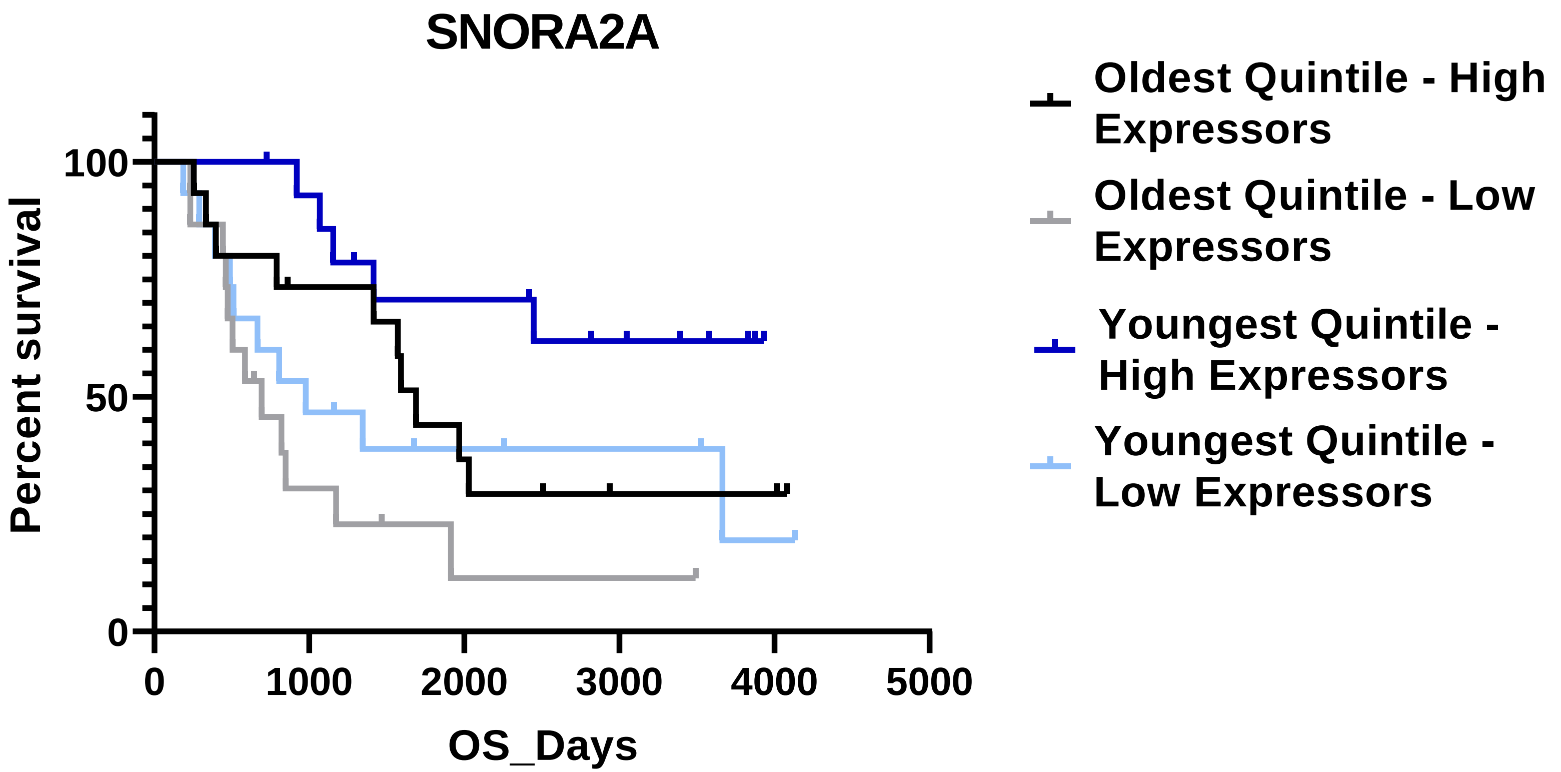
SNORA2A
Percent survival
100
50
0
0
1000
2000
3000
4000
5000
OS_Days
Oldest Quintile - High Expressors
Oldest Quintile - Low Expressors
Youngest Quintile - High Expressors
Youngest Quintile - Low Expressors

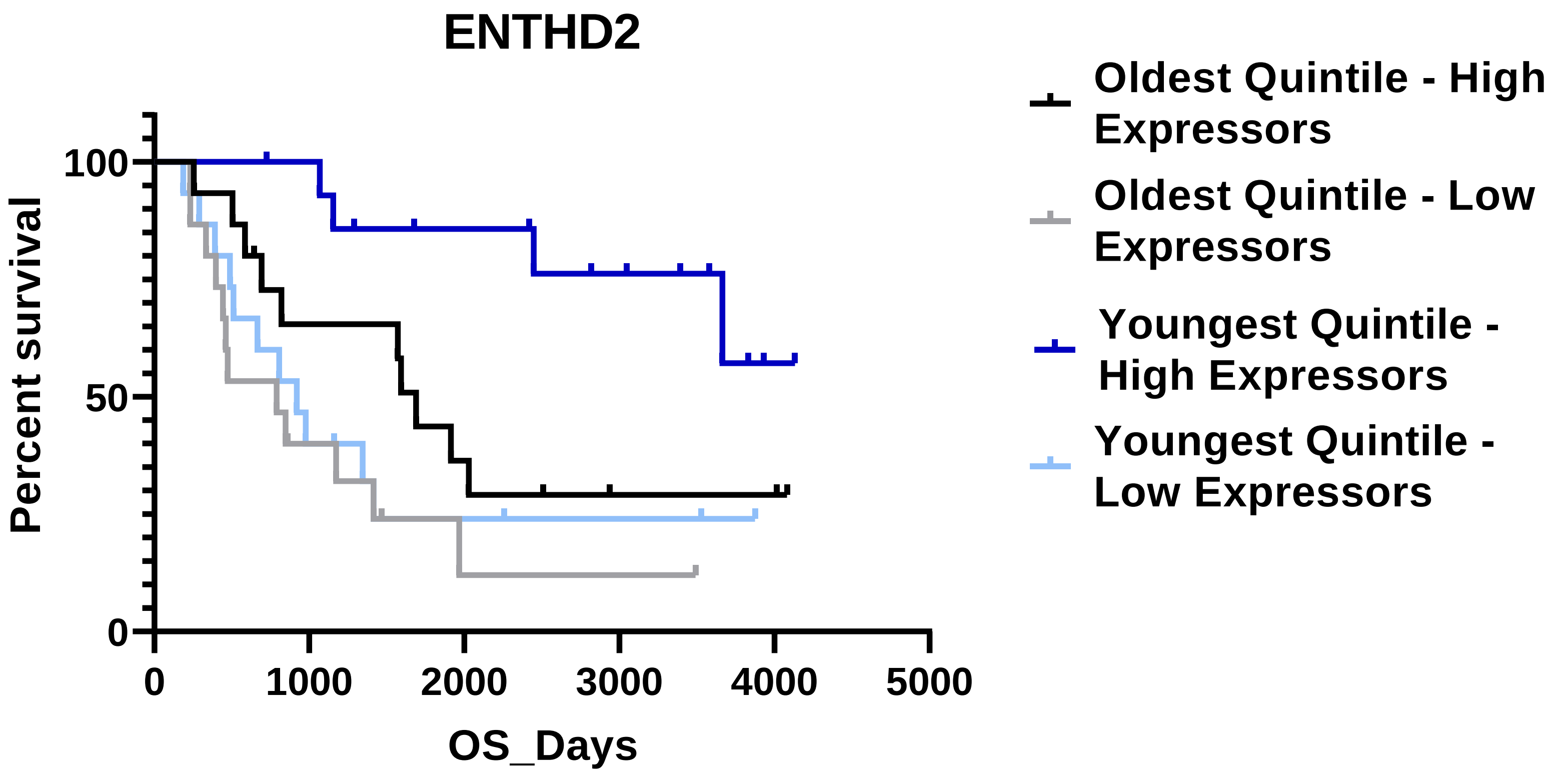
ENTHD2
Percent survival
100
50
0
0
1000
2000
3000
4000
5000
OS_Days
Oldest Quintile - High Expressors
Oldest Quintile - Low Expressors
Youngest Quintile - High Expressors
Youngest Quintile - Low Expressors

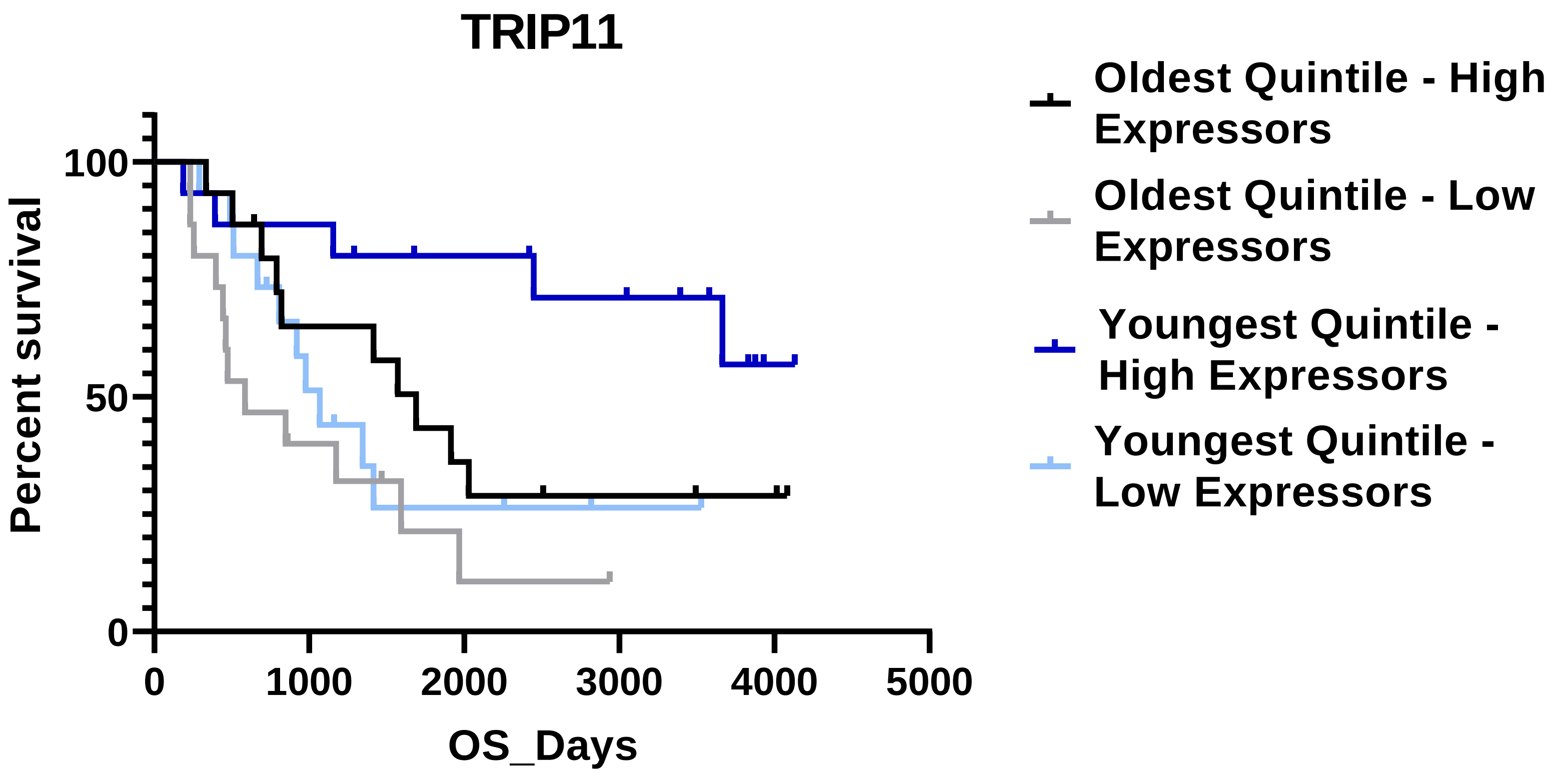

TRIP11
Percent survival
100
50
0
0
1000
2000
3000
4000
5000
OS_Days
Oldest Quintile - High Expressors
Oldest Quintile - Low Expressors
Youngest Quintile - High Expressors
Youngest Quintile - Low Expressors

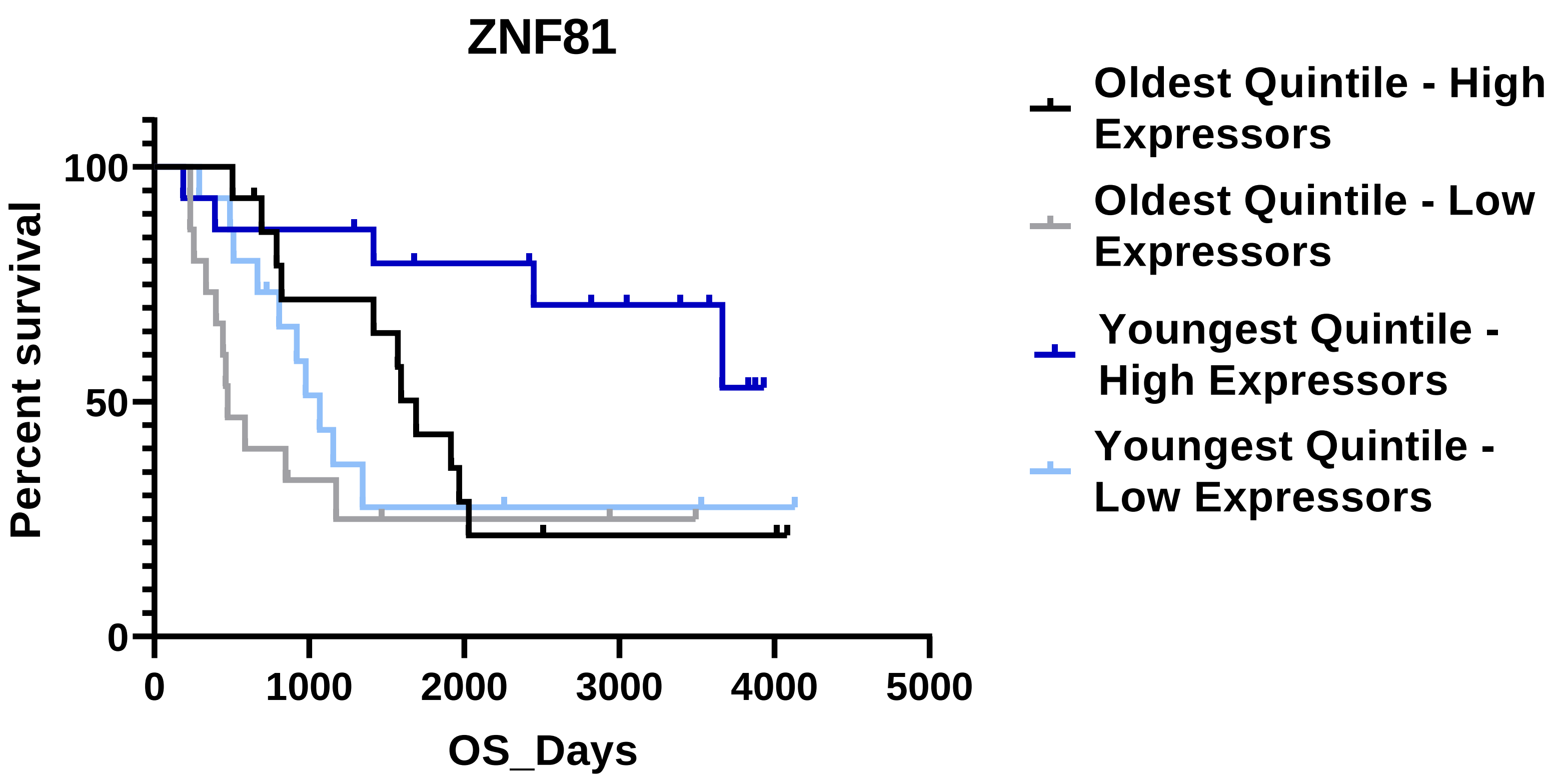
ZNF81
Percent survival
100
50
0
0
1000
2000
3000
4000
5000
OS_Days
Oldest Quintile - High Expressors
Oldest Quintile - Low Expressors
Youngest Quintile - High Expressors
Youngest Quintile - Low Expressors

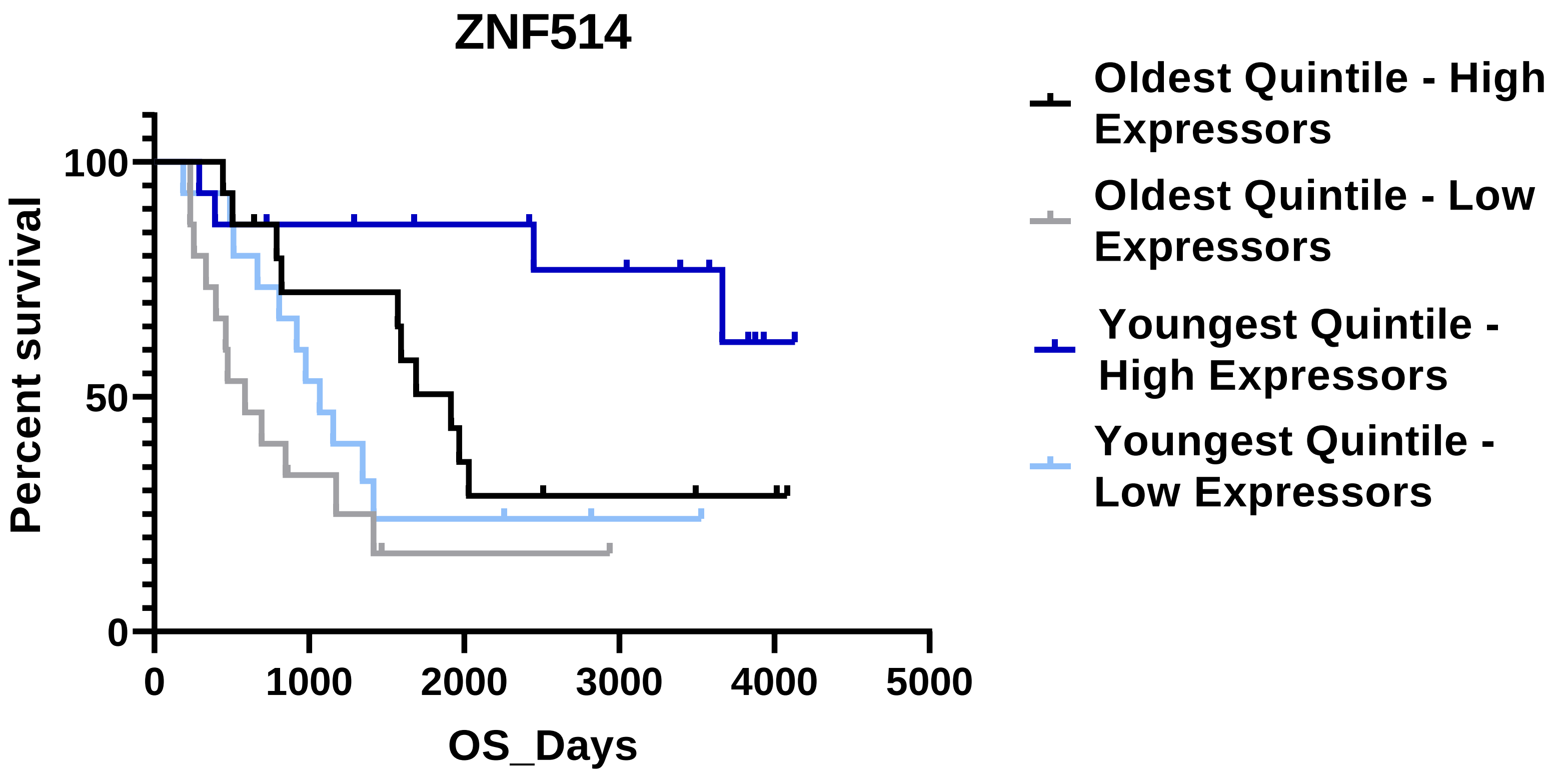

ZNF514
Percent survival
100
50
0
0
1000
2000
3000
4000
5000
OS_Days
Oldest Quintile - High Expressors
Oldest Quintile - Low Expressors
Youngest Quintile - High Expressors
Youngest Quintile - Low Expressors
